# Supplementary material for: Self-reported COVID-19 severity among persons with tuberculosis infection in western Kenya, 2021
Source: PLOS Glob Public Health. 2025 Apr 30;5(4):e0004372. doi: 10.1371/journal.pgph.0004372 (PMC12043119; doi:10.1371/journal.pgph.0004372)
Supplement: S1 File — ”. (PDF) [file pgph.0004372.s001.pdf]

Malaria as a risk factor for COVID-19 in western Kenya and Burkina Faso (MALCOV)

Trial Identifiers

Kenya SERU 348/4097	Kenya RA: KPPB ECCT/20/10/02	LSTM-REC 20-063	CDC-IRB [#####]
Burkina Faso 2020-10-222	Burkina Faso RA 2020-10-222	LSHTM-REC 22599	PACTR: 202009642148520

Chief Investigators

- Prof Feiko ter Kuile, MD, PhD, Department of Clinical Sciences, Liverpool School of Tropical Medicine (LSTM), United Kingdom. Tel: +44 151 705 3287, E-mail: Feiko.terKuile@lstmed.ac.uk
- Prof Chris Drakeley, PhD, Faculty of Infectious and Tropical Diseases, London School of Hygiene & Tropical Medicine (LSHTM), United Kingdom. Tel: +44 207 9272 289, E-mail: Chris.Drakeley@lshtm.ac.uk

Co-Principal Investigators

- Prof Sodiomon B. Sirima, MD, PhD, Groupe de Recherche Action en Santé(GRAS), Ouagadougou, Burkina Faso. Mobile: +226 7020 0444, E-mail: s.sirima@gras.bf
- Dr Simon Kariuki, PhD, Centre for Global Health Research (CGHR), Kenya Medical Research Institute (KEMRI), Kisumu, Kenya. Mobile: + 254 725 389 246; E-mail: SKariuki@kemricdc.org
- Dr Hellen Barsosio, MD, Centre for Global Health Research (CGHR), Kenya Medical Research Institute (KEMRI), Kisumu, Kenya. Mobile: +254 724 464 507, E-mail: Hellen.Barsosio@lstmed.ac.uk

Co-investigators for a full list of investigators and collaborators, see page 11

Funding Agency and Legal Registered Address Bill & Melinda Gates Foundation 1300 I St NW, Washington, DC 20005

Sponsor Names and Legal Registered Addresses

- For the study in Kenya: Liverpool School of Tropical Medicine (LSTM); Pembroke Place, Liverpool L3 5QA, UK, Phone: +44 0151 7053794; Email: lstmgov@lstmed.ac.uk
- For the study in Burkina Faso: London School of Hygiene and Tropical medicine (LSHTM), Keppel St, Bloomsbury, London WC1E 7HT, UK. Phone: +44 207 927 2102; Email: RGIO@lshtm.ac.uk

Revision chronology

Version and date of protocol	Details of Changes	Signature Chief Investigators
v1.2-12Oct20	Original protocol	
v1.3-16Nov20	Revised (see next page)	
v2.0-15Oct20	Amendment (see next page)	

Confidentiality Statement: This document contains confidential information that must not be disclosed to anyone other than the sponsor, the investigator team, host institution, relevant ethics committee and regulatory authorities

DESCRIPTION OF AMENDMENTS

Details of key changes to protocol version v1.3-16Nov20

Page	Paragraph	Details of changes (new text is underlined)
12	2.2.1, Lay summary	Erroneous duplicate mention of artemether-lumefantrine, corrected to pyronaridine-artesunate
15<br>31	2.2.3, Trial registration data<br>6.2.1.1, Inclusion criteria	Agrees not to self-medicate with chloroquine, hydroxychloroquine or other drugs ~~antimalarials~~ with potential anti-SARS-CoV-2 properties without prior discussion with medical study staff
32	6.2.3.2, Exclusion criteria	Received ~~chloroquine~~ any other antimalarials in the last three days
57	11.2.4.1, Binary endpoints	If all models fail to converge, ~~the Mantel Haenszel method~~ log-Poisson regression with a sandwich variance estimator will be used.
59	11.2.6.1, Baseline covariates	The seed for the imputation will be set as ~~128~~ a number with 8 digitals (e.g. the date of the programming).

v2.0-15Oct20

Page	Paragraph	Details of changes (new text is underlined)
1<br><br>11	Cover page, Trial identifiers<br>2, Administrative information	Updated the reference protocol number for the regulator in Burkina Faso
11	2.1.3, Co-investigators	The list of investigators was updated, and two new co-investigators were added
16<br><br>32<br><br>41<br><br><br>42	Key inclusion criteria COVID-19 cohort study<br>6.2.2.1, COVID-19 cohort study, Inclusion criteria<br>9.1.1, Pre-screening with point of contact (POC) SARS-COV-2 antigen tests<br>9.2.1, Pre-Screening (Day 0/1)	Added a positive antigen lateral flow test as part of the inclusion criteria for the COVID-19 cohort study<br><br>Removed ‘not previously diagnosed with Covid-19’ as exclusion criteria as this history cannot be reliably assessed. Instead, the presence of SARS-COV-2 antibodies at enrolment will now be taken into account in the analysis.
16<br><br>32<br>43	2.2.3, Trial registration data, key inclusion criteria<br>6.2.3.1, Inclusion criteria<br>9.2.1.3Nested malaria treatment trial	Updated the inclusion criteria for the nested malaria treatment study to include the HRP2 band on the malaria RDT<br>“Confirmed malaria infection by RDT (HRP2 or pLDH) or microscopy"
19<br><br>63	2.2.4, Schedule of activities;<br>12.7.1.4, Maximum blood volume;<br>Figure 5	The required blood volume in adults and older children above the age of assent was changed from 5 to 15 mL at the 3, 6, and 12 month follow-up visit

19	2.2.4, Schedule of activities;	We updated the range of days around the scheduled days for follow-up visits to occur; e.g. Day 14 (+/- 3)
21<br><br>41<br>45<br>120	2.2.4, Schedule of activities<br>9.1.2, Healthy controls<br>10.2.2, Blood samples<br>16.9.4, Immunological<br>CyTOF B/Tcell assays & and OLINK screen	Added a sub-group of 100 healthy controls consisting of a sub-group of participants who test negative by both SARS-CoV-2 antigen RDT and malaria RDT, and are feeling well. They will be asked to provide a single 5 mL venous blood sample for CyTOF for B and T cell markers (fixed whole blood), Olink immunoassay (plasma) and transcriptional profiling (gene expression). The data is needed for the correct interpretation of the systems immunology component and for transcriptional profiling and will be used as a comparison/control group for the COVID-19-malaria negative and the COVID-19-malaria positive patients.
21	2.2.4, Schedule of activities	Added a full blood count (FBC) (haemogram) 'when clinically indicated' to scheduled FBC taken on days 1 and 28 for patient care.
21	2.2.4, Schedule of activities	Added a repeat QuantiFERON test for screening for TB at the last scheduled follow-up visit at 12 month to exclude incident TB during the 1 year observation period. Added the option to take the first blood sample for QuantiFERON at any timepoint after enrolment and before month 12 if the QuantiFERON test was missed at enrolment.
30<br><br><br><br>33<br>34<br><br><br><br>34<br><br>38<br><br><br>58	5.1.3, Nested malaria treatment trial within the COVID-19 cohort study (N~142)<br>7, Interventions<br>7.1.1.1, Treatment and care of COVID-19 patients who are malaria-negative at enrolment<br>7.1.2, Nested malaria treatment trial<br>7.4.3, Permitted and prohibited concomitant medication and care<br>11.2.5.2, Assessment of efficacy	Removed the text describing that Burkina Faso at the time of protocol writing used chloroquine-azithromycin for the treatment of COVID-19. Burkina Faso dropped this policy before the first patient was recruited and the text is therefore no longer relevant.
31	6.1.1, Study setting, Kenya	Added Rabuor Health Centre as a study hospital
48	10.6, Pharmacokinetics	Added text to clarify that the timing of the last blood sample for pharmacokinetics should be scheduled about 3 to 4 hours after the last dose of the 3-day course has been taken
63<br><br>64	12.7.1.4, Maximum blood volume<br>Figure 5	Increased the blood volume to be taken at 3, 6 and 12 months from 5 to 15 mL to allow for Quantiferon TB test and similar repeat immunological assays as during the first 28 days of follow-up.
125	16.13 Patient information video	Introduction of a patient information video for download and to be shown in the waiting area of study clinics

TABLE OF CONTENTS

1 Abbreviations and terms 9

2 Administrative information 11

2.1 Investigators, collaborators and institutional affiliations 11

2.1.1 Chief Investigators 11

2.1.2 Co-Principal investigators 11

2.1.3 Co-investigators 11

2.1.4 Collaborators 11

2.1.5 Institutions 11

2.2 Protocol summaries and trial registration 12

2.2.1 Lay summary 12

2.2.2 Scientific summary/abstract 13

2.2.3 Trial registration data 14

2.2.4 Schedule of activities 19

3 Introduction 22

3.1 Background information 22

3.2 Problem statement and Justification for the study 23

3.2.1 Is malaria a risk factor for COVID-19 disease severity? 23

3.2.2 Does co-infection with malaria affect the rate of seroconversion and/or seroreversion to SARS-CoV-2 infection? 23

3.2.3 Does the choice of antimalarial treatment affect disease progression or the rate of seroconversion to SARS-CoV-2 infection? 24

3.2.4 Rationale for pyronaridine-artesunate 25

3.3 Null Hypotheses 25

4 Objectives and endpoints 26

4.1 Screening study (cross-sectional survey source population) (N~ 4,720) 26

4.2 Covid-19 cohort (N~708) 26

4.3 Nested malaria treatment trial in the COVID-19 cohort study (N~142) 27

5 Study design 28

5.1 Overview study design 29

5.1.1 Screening study (cross-sectional survey source population) (N~4,720) 29

5.1.2 COVID-19 cohort study (N~708) 30

5.1.3 Nested malaria treatment trial within the COVID-19 cohort study (N~142) 30

5.2 Participant and study completion 30

6 Study setting and population 31

6.1 Study setting 31

6.1.1 Kenya 31

6.1.2 Burkina Faso 31

6.2 Study population 31

6.2.1 Screening study (cross-sectional survey source population) 31

6.2.2 COVID-19 cohort study 32

6.2.3 Nested malaria treatment trial 32

6.2.4 Co-enrolment guidelines 33

7 Interventions ... 33
7.1 Treatments administered ... 33
7.1.1 COVID-19 cohort study ... 33
7.1.2 Nested malaria treatment trial ... 34
7.2 Risks to the participants ... 34
7.2.1 Risks associated administration with artemether-lumefantrine ... 34
7.2.2 Risks associated with pyronaridine-artesunate ... 35
7.3 Method of treatment assignment ... 36
7.4 Blinding ... 36
7.4.1 Preparation/handling/storage/accountability ... 37
7.4.2 Strategies to improve adherence to study protocol monitoring adherence ... 37
7.4.3 Permitted and prohibited concomitant medication and care ... 38
7.4.4 Treatment after the end of the study ... 39
8 Discontinuation/withdrawal criteria ... 39
8.1.1 Discontinuation of study treatment ... 39
8.1.2 Withdrawal from the study ... 39
8.1.3 Lost to Follow-up ... 40
9 Participants timeline and study encounters ... 40
9.1 Screening study (cross-sectional survey source population) ... 40
9.1.1 Pre-screening with point of contact (POC) SARS-COV-2 antigen tests ... 41
9.1.2 Healthy controls ... 41
9.2 COVID-19 cohort study and nested malaria treatment trial ... 42
9.2.1 Pre-Screening (Day 0/1) ... 42
9.2.2 Scheduled follow-up visits day 3, 7, 14, 21, 28 and 42 ... 43
9.2.3 Telephone contact Days 2, 4-6, 8-13, 15-27 ... 44
9.2.4 Close-out visit day 28 Cohort ... 44
9.2.5 Close-out visit day 42 nested malaria treatment trial ... 44
9.2.6 Extended follow-up visits at month 3, 6, and 12 ... 45
9.2.7 Participants who develop malaria during the 42 days follow-up period ... 45
10 Study assessment and procedures ... 45
10.1 Schedule of activities and samples ... 45
10.2 Biological samples ... 45
10.2.1 Mid-nasal swab ... 45
10.2.2 Blood samples ... 45
10.2.3 Other biological samples ... 46
10.2.4 HIV testing and TB testing ... 46
10.3 Assessment of clinical and epidemiological endpoints ... 46
10.3.1 Assessment of disease progression ... 46
10.3.2 Assessment of the antimalarial treatment response ... 47
10.4 Assessment of immunological endpoints ... 47
10.4.1 Cytokine, chemokine, and inflammatory marker profile ... 47
10.4.2 Antibody Testing ... 47
10.4.3 transcriptional profiling and cellular activation ... 47
10.5 Genetic testing ... 47
10.5.1 Sample for host genetic testing ... 47
10.5.2 Host genetic information that will be obtained and genetic testing procedures ... 47

10.6 Pharmacokinetics ... 48
10.7 Sample storage and shipping ... 48
10.7.1 Storage ... 48
10.7.2 Shipping ... 48
10.8 Data collection methods & storage ... 49
10.8.1 Methodologies for data collection / generation ... 49
10.8.2 Data quality and standards ... 49
10.8.3 Managing, storing and curating data ... 49
10.8.4 Metadata standards and data documentation ... 49
10.8.5 Data preservation strategy and standards ... 49
10.9 Data monitoring and trial oversight committees ... 50
10.9.1 Data Safety and Monitoring Board (DSMB) ... 50
10.10 Safety monitoring and reporting ... 50
10.10.1 Definitions ... 50
10.10.2 Identifying, managing adverse events ... 51
10.10.3 Assessment of causality ... 51
10.10.4 Reporting adverse event procedures ... 52
10.11 Biohazard containment ... 53
10.12 Trial monitoring and auditing ... 53
10.12.1 Trial monitoring ... 53
10.12.2 Auditing ... 54
10.12.3 Role of sponsor ... 54
10.13 Other quality control measures ... 54
10.13.1 Safety monitors ... 54
10.13.2 Internal monitoring ... 54
10.13.3 Training ... 54
10.13.4 Quality assurance/control of laboratory tests ... 55
11 Statistical considerations ... 55
11.1 Sample size determination ... 55
11.1.1 Cohort ... 55
11.1.2 Immunological components ... 55
11.1.3 Nested malaria treatment trial ... 56
11.1.4 Screening study (cross-sectional survey source population) ... 56
11.1.5 Interim sample size re-estimations and feasibility of recruitment ... 56
11.2 Statistical analysis ... 56
11.2.1 General principles ... 56
11.2.2 Populations for analysis ... 57
11.2.3 Screening study (cross-sectional survey source population) ... 57
11.2.4 Analytical methods by type of endpoint measure ... 57
11.2.5 Additional statistical consideration for the nested malaria treatment trial ... 58
11.2.6 Missing data ... 59
11.2.7 Multiplicity ... 60
11.2.8 Pharmacokinetic analysis ... 60
12 Ethics and dissemination ... 60
12.1 Declaration of Helsinki ... 60
12.2 Research ethics and regulatory approval ... 60
12.2.1 Review process ... 60

12.2.2 Protocol amendments ... 60
12.2.3 Sanctioning of the protocol by hospitals ... 61
12.2.4 Regulatory approval ... 61
12.3 Informed consent procedures ... 61
12.3.1 Consent procedures ... 61
12.4 Protection of privacy and confidentiality ... 62
12.4.1 Privacy ... 62
12.4.2 Privacy of individual ... 62
12.4.3 Confidentiality of data ... 62
12.5 Declaration of interest ... 62
12.6 Access to source data/documents ... 62
12.7 Risks and benefits ... 62
12.7.1 Risks to Study Participants ... 62
12.7.2 Benefits to study participants or society ... 64
12.7.3 Risk to the population and study staff and biohazard containment ... 65
12.7.4 Other ethical considerations ... 66
12.8 Ancillary and post-trial care ... 66
12.8.1 Health care during the trial ... 66
12.8.2 Trial insurance ... 66
12.8.3 Post-trial care ... 66
12.9 Expenses reimbursement and incentives ... 66
12.10 Dissemination and application of the results ... 67
12.10.1 Result dissemination and publication policy ... 67
12.10.2 Impact ... 67
12.10.3 Authorship and publications ... 67
12.10.4 Data Sharing Statement ... 68

13 Timeframe and duration of the study ... 69
13.1 Timeline ... 69
13.2 Gantt chart ... 69

14 Financial aspects and conflict of interest ... 70
14.1 Funding for the trial ... 70
14.2 Provision of study drugs ... 70
14.3 Budget & budget justification ... 70

15 References ... 71

16 Appendices ... 75
16.1 Appendix I. Role of Investigators and collaborators ... 75
16.1.1 Definition investigator and collaborator ... 75
16.1.2 Protocol development: authors' contributions ... 75
16.1.3 Role Investigators ... 75
16.2 Appendix II. Terms of reference study management group and DSMB ... 76
16.2.1 Study Management Group (TMG) ... 77
16.2.2 Data Safety and Monitoring Board (DSMB) ... 77
16.2.3 Membership ... 77
16.3 Appendix III. Declaration of Helsinki ... 79
16.4 Appendix IV. WHO clinical progression scale ... 82
16.5 Appendix V. Modified Flu-PRO PLUS scale ... 83

16.6 Appendix VI. Personal protection equipment guidelines for study staff by risk category ... 85
16.7 Appendix VII. Product characteristics ... 86
16.7.1 Pyronaridine-artesunate ... 86
16.7.2 Artemether-lumefantrine ... 101
16.8 Appendix VIII. Budget and budget justification ... 116
16.8.1 Budget ... 116
16.8.2 Budget Justification ... 117
16.9 Appendix IX. Description of other clinical and laboratory methods ... 118
16.9.1 Novel diagnostics for COVID-19 ... 118
16.9.2 Kinetics of SARS-CoV-2 and malaria antibodies ... 119
16.9.3 Evaluation of immune biomarkers ... 119
16.9.4 Immunological CyTOF B/Tcell assays & and OLINK screen ... 120
16.9.5 Non-malaria serology and detection of other infection targets ... 121
16.9.6 Host genetics ... 122
16.9.7 Pharmacokinetic studies ... 122
16.10 Appendix X. KEMRI Proposal format checklist ... 124
16.11 Appendix XI. Questionnaires ... 125
16.12 Appendix XII. Participant information sheets and informed consent and assent statements ... 125
16.12.1 Adults ... 125
16.12.2 Parents/guardians ... 125
16.12.3 Minors ... 125
16.13 Patient information video ... 125

1 ABBREVIATIONS AND TERMS

Term	Definition
95% CI	95 percent confidence interval
ACE2	Angiotensin-converting enzyme 2
ACT	Artemisinin-Based Combination Therapy
AE	Adverse event
AIDS	Acquired Immunodeficiency Syndrome
AL	Artemether-lumefantrine
AQ	Amodiaquine
ART	Antiretroviral Therapy
AUC	Area Under the Curve
AZ	Azithromycin
CDC	US Centers for Disease Control and Prevention
CHW	Community Health Worker
Cmax	Maximum Drug Concentration
CTEC	Comité Technique pour les Essais Cliniques, Burkina Faso
COVID-19	Coronavirus disease 2019
CQ	Chloroquine
CRF	Case Report Form
CYP450	Cytochrome P450
DBS	Dried blood spot
DHA	Dihydroartemisinin
DHA-PPQ	Dihydroartemisinin-piperaquine
DSMB	Data and safety monitoring board
EC_{50}	Half-maximal effective concentration
ECG	Electrocardiogram
eCRFs	Electronic case report forms
Eligible	Qualified for enrolment into the study based inclusion/exclusion criteria
FDA	Food and Drug Administration
GCP	Good Clinical Practice
GFR	Glomerular filtration rate
GRAS	Le Groupe de Recherche Action en Santé
Hb	Haemoglobin
HCQ	Hydroxychloroquine
HIPAA	Health Insurance Portability and Accountability Act
HIV	Human Immunodeficiency Virus
hRSV	human Respiratory Syncytial Virus
IATA	International Air Transport Association
ICF	Informed consent form
IPC	Infection Prevention and Control
IRB	Institutional Review Board
ITNs	Insecticide Treated Nets
ITT	Intention to Treat
KEMRI	Kenya Medical Research Institute
LLINS	Long-Lasting Insecticide Treated Nets
LSHTM	London School of Hygiene and Tropical Medicine
LSTM	Liverpool School of Tropical Medicine

MDA	Mass Drug Administration
MERS-CoV	Middle East Respiratory Syndrome coronavirus
MoH	Ministry of Health
NMCP	National Malaria Control Programme
Participant(s)	A term used throughout the protocol to denote the enrolled individual(s)
PA	Pyronaridine-artesunate
PCR	Polymerase chain reaction
PD	Pharmacodynamics
PEP	Post-exposure prophylaxis
PK	Pharmacokinetic
PPQ	Piperaquine
RA	Regulatory authority
RCT	Randomised Controlled Trial
RDT	Rapid Diagnostic Test
REC	Research Ethics Committee
RNA	Ribonucleic acid
SAE	Serious adverse event
SARI	Severe acute respiratory illness
SARS-CoV	Severe acute respiratory syndrome coronavirus
SARS-CoV-2	Severe acute respiratory syndrome coronavirus 2
SmPc	Summary of product characteristics
SoA	Schedule of Activities
SOP	Standard Operating Procedure
SP	Sulphadoxine-Pyrimethamine
T1/2	Plasma Half-Life
QED	Quantitative Engineering Design®
QTc	Rate Corrected Time Qt Interval on Electrocardiogram (ECG)
QTcB	Rate Corrected Time Qt Interval on ECG Using Bazett's Correction
QTcF	Rate Corrected Time Qt Interval on ECG Using Fridericia's Correction
WES	Whole exome sequencing
WHO	World Health Organization

2 ADMINISTRATIVE INFORMATION

2.1 INVESTIGATORS, COLLABORATORS AND INSTITUTIONAL AFFILIATIONS

2.1.1 Chief Investigators

Prof Feiko ter Kuile, MD, PhD[1,3]

Prof Chris Drakeley, PhD[2]

2.1.2 Co-Principal investigators

Dr Hellen Barsosio, MD, PhD (Kenya)[1,3]

Dr Simon Kariuki, PhD (Kenya)[3]

Prof Sodiomon B. Sirima, MD, PhD (Burkina F.)[4]

2.1.3 Co-investigators

Mr Kephas Otieno, MSc[3]

Dr Titus Kwambai, MD, PhD[5]

Dr Clayton Onyango, PhD[5]

Dr Victor Akelo, MD, MPH, MBA[5]

Brian Tangara, MSc[3]

Prof Penelope Phillips-Howard, PhD[1,3]

Dr Alfred B Tiono, MD, PhD[4]

Dr Issa Ouedraogo, PharmD, MSc, PhD[4]

Dr Issiaka Soulama, MSc, PhD[4]

Dr Maia Lesosky, PhD[1]

Dr Aaron Samuels, MD, MHS[1,5,7]

2.1.4 Collaborators

Mr Fredrick Oluoch[9]

Dr Kennedy Oruenjo[9]

Dr Gordon Okomo[9]

Mr Michael Nyachae, Bpharm[9]

Dr Melsa Lutomia[9]

Prof Giancarlo Biagini[1]

Dr Ghaith Aljayyoussi[1]

Prof Andrew Owen[13]

Dr Emily Adams[1]

Prof Tobias Rinke de Wit[11]

Dr Laura Steinhardt[7]

Dr Taraz Samandari[5,7]

Dr Elizabeth Hunsperger[5,7]

Dr Marc Bulterys[5,7]

Dr William Wu[17]

Dr Brice Bicaba, MD, MSc[6]

Dr Joe Fitchett [10]

Prof Amadou A.Sall [12]

Dr Kevin Tetteh[2]

Dr David Allen[2]

Dr John Bradley[2]

Dr Tegwen Marlais[2]

Dr Christian (Chris) Ockenhouse[16]

Dr Richard (Rick) Steketee[15]

Dr Gerlinde Obermoser[14]

Prof Holden Maecker[14]

Dr Scott Miller[18]

Dr Jean-Luc Bodmer[18]

2.1.5 Institutions

1 Liverpool School of Tropical Medicine, Liverpool
2 London School of Hyg and Trop Medicine, London
3 Centre for Global Health Research, Kenya Medical Research Institute, Kisumu, Kenya
4 Groupe de Recherche Action en Santé (GRAS), Ouagadougou, Burkina Faso
5 Centers for Disease Control and Prevention (CDC), Kenya, Kisumu, Kenya
6 CORUS, National Public Health Institute, Ouagadougou, Burkina Faso
7 US CDC, Atlanta, GA, USA
8 Ministry of Health, Burkina Faso
9 Ministry of Health, Kenya
10 Mologic, Ltd, Thurleigh, UK
11 PharmAccess, AIGHD, University of Amsterdam
12 Institute Pasteur, Senegal
13 University of Liverpool
14 Stanford University, CA, USA
15 PMI USAID, Washington DC, USA
16 PATH, Washington DC, USA
17 Quantitative Engineering Design (QED), Poland
18 Bill and Melinda Gates Foundation

2.2 PROTOCOL SUMMARIES AND TRIAL REGISTRATION

2.2.1 Lay summary

We will conduct a clinical study in western Kenya and Burkina-Faso to determine if malaria affects the severity of COVID-19 or its duration. Some patients with COVID-19 also have malaria. We will determine if the treatment of malaria with pyronaridine-artesunate in these patients has an effect on COVID-19 severity or the immune response to COVID-19. Pyronaridine-artesunate is a highly effective malarial treatment that is being rolled out in Africa. In laboratory tests, pyronaridine-artesunate is able to kill viruses, including SARS-CoV-2, the Coronavirus that causes COVID-19. We will follow 708 patients of all ages with COVID-19 for 12 months (the 'COVID-19 cohort study'). They will be enrolled from a population of approximately 4,720 patients who are being screened for COVID-19 ('Screening study'). It is anticipated that about 1 in 5 of the 708 COVID-19 patients will also have malaria (about 142). They will be treated with either artemether-lumefantrine (the current standard malaria treatment) or pyronaridine-artesunate. The assignment will be decided by chance (random). Hospitalisation, self-isolation and home-based care will follow national guidelines. All 708 COVID-19 patients will be seen again during home/clinic visits on days 1, 3, 7, 14, 21, 28, and 42 when swabs for COVID-19 tests and blood samples will be taken. They will then be seen again at 3, 6, and 12 months. The severity and duration of symptoms, the immune response, and the time that patients remain infectious (can spread the virus to others) will be compared between COVID-19 patients with malaria and without malaria, and between patients treated with artemether-lumefantrine and with pyronaridine-artesunate. Strict adherence to personal protection equipment (PPE) will be used to limit the transmission of the Coronavirus. The transport arrangements for patients and staff will follow national COVID-19 guidelines. Written informed consent/assent will be sought. Participants will potentially benefit from close observations. There is a potential benefit to society as the study will help to obtain a better understanding of the impact of COVID-19 on people's health in malaria-endemic areas. The study is anticipated to start in October 2020 and finish approximately 18 months later in March 2022.

2.2.2 Scientific summary/abstract

It is unknown whether malaria or malaria treatment affects COVID-19 severity, immune responses to SARS-CoV-2 virus, or viral loads and/or duration of shedding and therewith the onwards spread of SARS-COV-2. We will conduct an observational cohort study in 708 newly diagnosed COVID-19 patient of all ages in western Kenya and Burkina-Faso. They will be enrolled in hospitals with COVID-19 testing facilities from a source population screened for SARS-CoV-2 (N~4,720). Approximately 142 of the 708 COVID-19 patients are expected to be co-infected with malaria. They will be enrolled in the nested malaria treatment trial and randomized to receive 3-days of artemether-lumefantrine (the current standard of care) or pyronaridine-artesunate, a highly effective antimalarial with known antiviral properties against SARS-CoV-2 in-vitro, that is newly registered and being rolled out in Africa. Disease progression will be assessed and nasal swabs and blood samples taken during home/clinic visits on days 1, 3, 7, 14, 21, 28, and 42 and blood samples at 3, 6, and 12 months. Patients self-isolating will be phoned daily for the first 14 days to assess signs and symptoms. Hospitalisation, self-isolation and home-based care will follow national guidelines. The WHO clinical progression scale and FLU-PRO plus scales will be used to compare disease progression between COVID-19 patients with and without malaria, and by malaria. Other endpoints include seroconversion/reversion rates, chemokine/cytokine responses, T and B cell responses, viral load and duration of viral carriage. Infection prevention and control (IPC), including use of personal protection equipment (PPE), and measures for patient transport will follow national guidelines in each country. Written informed consent/assent will be sought. Participants will potentially benefit from close observations. Society may benefit from improved understanding of the impact of COVID-19 in malaria-endemic areas. The study is anticipated to start in October 2020 and last for approximately 18 months.

2.2.3 Trial registration data

Data Category	Information
Primary registry and trial identifying number	PACTR: 202009642148520
Date of registration in primary registry	[#####]
Secondary identifying numbers	Kenya SERU 348/4097; Kenya RA: KPPB ECCT/20/10/02; LSTM-REC 20-063; CDC-IRB [#####]<br>Burkina Faso 2020-10-222; Burkina Faso RA 2020-10-222; LSHTM-REC 22599
Source(s) of monetary or material support	Bill & Melinda Gates Foundation 1300 I St NW, Washington, DC 20005
Primary sponsor	• For the study in Kenya: Liverpool School of Tropical Medicine (LSTM); Pembroke Place, Liverpool L3 5QA, UK, Phone: +44 0151 7053794; Email: lstmgov@lstmed.ac.uk<br>• For the study in Burkina Faso: London School of Hygiene and Tropical medicine (LSHTM), Keppel St, Bloomsbury, London WC1E 7HT, UK. Phone: +44 207 927 2102; Email:
Secondary sponsor(s)	NA
Contact for public queries	• Prof Feiko ter Kuile, MD, PhD, Department of Clinical Sciences, Liverpool School of Tropical Medicine (LSTM), United Kingdom. Tel: +44 151 705 3287, E-mail: Feiko.terKuile@lstmed.ac.uk<br>• Prof Chris Drakeley, PhD, Faculty of Infectious and Tropical Diseases, London School of Hygiene & Tropical Medicine (LSHTM), United Kingdom. Tel: +44 207 9272 289, E-mail: Chris.Drakeley@lshtm.ac.uk<br>• Prof Sodiomon B. Sirima, MD, PhD, Groupe de Recherche Action en Santé(GRAS), Ouagadougou, Burkina Faso. Mobile: +226 7020 0444, E-mail: s.sirima@gras.bf<br>• Dr Simon Kariuki, PhD, Centre for Global Health Research (CGHR), Kenya Medical Research Institute (KEMRI), Kisumu, Kenya. Mobile: + 254 725 389 246; E-mail: SKariuki@kemricdc.org
Contact for scientific queries	• Prof Feiko ter Kuile, MD, PhD, Department of Clinical Sciences, Liverpool School of Tropical Medicine (LSTM), United Kingdom. Tel: +44 151 705 3287, E-mail: Feiko.terKuile@lstmed.ac.uk<br>• Prof Chris Drakeley, PhD, Faculty of Infectious and Tropical Diseases, London School of Hygiene & Tropical Medicine (LSHTM), United Kingdom. Tel: +44 207 9272 289, E-mail: Chris.Drakeley@lshtm.ac.uk<br>• Prof Sodiomon B. Sirima, MD, PhD, Groupe de Recherche Action en Santé(GRAS), Ouagadougou, Burkina Faso. Mobile: +226 7020 0444, E-mail: s.sirima@gras.bf<br>• Dr Simon Kariuki, PhD, Centre for Global Health Research (CGHR), Kenya Medical Research Institute (KEMRI), Kisumu, Kenya. Mobile: + 254 725 389 246; E-mail: SKariuki@kemricdc.org
Public title	Malaria as a risk factor for COVID-19 in western Kenya and Burkina Faso (MALCOV)
Scientific title	Malaria as a risk factor for COVID-19 in western Kenya and Burkina Faso (MALCOV)
Countries of recruitment	Kenya and Burkina Faso
Health condition(s) or problem(s) studied	Malaria, COVID-19

Intervention(s)	COVID-19 cohort study<br>Malaria-negative participants: Standard of care (SOC)<br>Malaria-positive participants: see nested malaria treatment trial, below<br><br>Nested malaria treatment trial<br>• Artemether-lumefantrine, standard 3-day antimalarial treatment regimen<br>• Pyronaridine-artesunate, standard 3-day antimalaria treatment regimen
Study type	Interventional and observational
	Observational: screening study (cross-sectional survey, source population)Screening study (cross-sectional survey source population)Cross-sectional assessment of a source population consisting of suspected COVID-19 cases and their contacts and high-risk populations.<br>Observational cohort: Participants with COVID-19 with and without malaria will be followed for 28 days, and then at 3, 6, and 12 months.
	Interventional: Covid-19 cases co-infected with malaria will be enrolled in a nested malaria treatment trial. Allocation: randomised; intervention model: parallel assignment; arms: 2; allocation ratio: 1:1; stratified by site (i.e. hospital or clinic) and by the presence of symptoms at enrolment consistent with COVID-19 (asymptomatic vs symptomatic). Masking: blinding of primary outcome assessor (off-site laboratory-based staff)
	Primary purpose: Treatment and prevention
	Phase-III
Date first enrolment	[dd mmm yyyy]
Target sample size	Screening study (cross-sectional survey source population): About 4,720<br>COVID-19 cohort study: About 708<br>Nested malaria treatment trial: About 142
Recruitment status	Not yet recruiting
Primary Objectives	Screening study (cross-sectional survey source population)<br>• To determine if malaria infection is predictive of the prevalence of SARS-CoV-2, adjusted for other risk factors of SARS-CoV-2 infection<br><br>COVID-19 cohort study<br>• To determine if malaria infection affects COVID-19 severity<br><br>Nested malaria treatment trial<br>• To determine if pyronaridine-artesunate compared to artemether-lumefantrine reduces early SARS-CoV-2 viral shedding
Key inclusion criteria	Screening study (cross-sectional survey source population)<br>• Patients attending COVID-19 test centres or other clinics conducting testing for COVID-19<br>• Patient population admitted to the COVID-19 isolation/quarantine facilities<br>• Contacts of confirmed or suspected cases as part of contact tracing<br>• Populations as part of targeted testing of high-risk populations (e.g., frontline health care workers)<br><br>COVID-19 cohort study

	• Laboratory confirmed SARS-CoV-2 infection, with positive molecular test results within the past 72 hours* or a positive rapid diagnostic antigen test<br>• Aged >=6 months **<br>• Resident in the study area<br>• The participant or caretaker is willing and able to give informed consent or assent with parent/guardian informed consent for participation in the study<br>• Agrees not to self-medicate with chloroquine, hydroxychloroquine or other drugs with potential anti-SARS-CoV-2 properties without prior discussion with medical study staff<br>•<br>* We anticipate that most tests results will be available within 24 hours. The maximum of 72 hours is a target and can be increased to 120 hours (5 days) in sites that experience significant delays related to routine testing for SARS-CoV-2 by the local health authorities.<br>** Children < 6 months will be excluded because of the inconvenience of repeated blood sampling and because they have a different clinical presentation of malaria than older children, as they are partially protected from developing clinical malaria due to the protective effect of maternal immunity after birth and through factors that inhibit parasite growth, such haemoglobin F (HbF).<br><br>Nested malaria treatment trial<br>• Fulfils all inclusion criteria for the COVID-19 cohort study<br>• Uncomplicated malaria, defined as able to take oral medication<br>• Bodyweight ≥5kg<br><br>Confirmed malaria infection by RDT (HRP2 or pLDH) or microscopy
Key exclusion criteria	Screening study (cross-sectional survey source population)<br>• Unwilling or unable to provide informed consent/assent<br><br>COVID-19 cohort study<br>• Unwilling or unable to provide informed consent/assent<br>• The participant is judged by the Investigator to be at significant risk of failing to comply with the provisions of the protocol as to cause harm to self or seriously interfere with the validity of the study results<br>• Inability/unlikely to be in the study area for the duration of the 28-day follow-up period<br>Nested malaria treatment trial<br>• Fulfils any of the exclusion criteria for the COVID-19 cohort study<br>• Pregnant or lactating women<br>• Severe disease requiring parenteral treatment<br>• Currently receiving, or recently received (within the last 28 days) pyronaridine-artesunate or artemether-lumefantrine<br>• Received any other antimalarials in the last three days<br>• Inability/unlikely to be in the study area for the duration of the 42-day follow-up period<br>• Known hypersensitivity or specific contraindication to the use of any of the study drugs in the treatment arms

	• Known chronic kidney disease (signs or symptoms of stage IV renal impairment or receiving dialysis)<br>• Known liver cirrhosis (Child-Pugh Class B or greater) or signs or symptoms of severe hepatotoxicity

Objectives and outcomes

Screening study (cross-sectional survey source POPULATION) (N~ 4,720)

Primary objective	Primary endpoint
• To determine if malaria infection is predictive of the prevalence of SARS-CoV-2, adjusted for other risk factors of SARS-CoV-2 infection	• Cross-sectional comparison of the proportion of participants with SARS-CoV-2 in the source population among participants with and without malaria infection expressed as the prevalence ratio for COVID-19 (95% CI)
**Secondary objectives**	**Secondary endpoints**
• To determine the demographic and clinical risk factor for the prevalence of SARS-CoV-2 infection	• Cross-sectional comparison of demographic and clinical risk factors of SARS-CoV-2 in the source population expressed as a prevalence ratio for COVID-19 (95% CI)

Covid-19 cohort (N~708)

Primary objective	Primary endpoint
• To determine if malaria infection affects COVID-19 severity	• Comparison of the severity of COVID-19 by day-28 in cases with and without malaria using the WHO clinical progression scale
**Secondary objectives**	**Secondary endpoints**
• To determine if malaria infection affects COVID-19 disease presentation and duration	• Symptoms and duration of COVID-19 by day-28 in cases with and without malaria
• To determine if malaria infection in COVID-19 patients alters the antibody response to SARS-CoV-2 infection	• Cumulative seroconversion rates* (total antibody, IgG, IgM, IgA) by days 7, 14, 21, 28, and total antibody, IgG, IgM, IgA antibody titres against SARS-CoV-2 by day-28 expressed as the geometric mean, maximum, and change from baseline<br>• Seroreversion rates for IgG, IgM, IgA and geometric mean antibody titres against SARS-CoV-2 by month 3, 6 and 12
• To determine if malaria infection in COVID-19 alters the inflammatory, genomic, and cellular immune responses to SARS-CoV-2 infection	• Multiplex serum cytokine markers; Transcriptional profiling (gene expression) of whole blood; Fixed whole blood for T and B cell markers by day-28
• To determine if malaria infection in COVID-19 alters the viral load or duration of SARS-CoV-2 infection	• Median viral load of SARS-CoV-2 detected from mid-nasal swabs by PCR<br>• Cumulative incidence of SARS-CoV-2 clearance (defined as the proportion of participants with negative nasal swabs) by days 7, 14, 21 and 28

	• Time to clearance of nasal SARS-CoV-2, defined as negative SARS-CoV-2 RNA PCR tests (swabs collected on days 1, 3, 7, 14, 21 and 28)
Nested malaria treatment trial in the COVID-19 cohort study (N~142)	
Primary objective	Primary endpoint
• To determine if pyronaridine-artesunate compared to artemether-lumefantrine reduces early SARS-CoV-2 viral shedding	• Incidence of SARS-CoV-2 clearance (defined as the proportion of participants with a negative nasal swab) on Day 7 after the start of treatment
Secondary objectives	Secondary endpoints
• To determine if pyronaridine-artesunate compared to artemether-lumefantrine alters the viral load and/or the duration of SARS-CoV-2 viral shedding	• Median viral load of SARS-CoV-2 detected from mid-nasal swabs by PCR<br>• Cumulative incidence of SARS-CoV-2 clearance (defined as the proportion of participants with negative nasal swabs) by days 14, 21 and 28<br>• Time to clearance of nasal SARS-CoV-2, defined as negative SARS-CoV-2 RNA PCR tests (swabs collected on days 1, 3, 7, 14, 21 and 28)
• To determine if treatment of malaria infection with pyronaridine-artesunate in COVID-19 patients alters the antibody response to SARS-CoV-2 infection compared to treatment with artemether-lumefantrine	• Cumulative seroconversion rates (IgG, IgM, IgA) by days 7, 14, 21 and 28, and IgG, IgM, IgA antibody titres against SARS-CoV-2 by day-28 expressed as the geometric mean, maximum, and change from baseline
• To determine if treatment of malaria infection with pyronaridine-artesunate in COVID-19 alters the inflammatory, genomic, and cellular immune responses to SARS-CoV-2 infection compared to treatment with artemether-lumefantrine	• Multiplex serum cytokine markers; Transcriptional profiling (gene expression) of whole blood; Fixed whole blood for T and B cell markers by day-28
• To compare the clinical and parasitological efficacy of pyronaridine-artesunate versus AL in COVID-19 patient coinfected with malaria parasites.	• The proportion with early treatment failure, late clinical failure, late parasitological failure or an adequate clinical and parasitological response. Recrudescence will be differentiated from new infection by genotyping of malaria parasites by day 42
• To determine if pyronaridine-artesunate compared to artemether-lumefantrine alters COVID-19 disease progression and severity	• COVID-19 symptoms, duration and severity as defined by a severity index score by day 28<br>• The proportion of days with a fever after randomization<br>• The proportion of days with respiratory symptoms after randomization
• To test the safety of pyronaridine-artesunate compared to artemether-lumefantrine in COVID-19 patient coinfected with malaria parasites	• The cumulative proportion of treatment-related adverse events and serious adverse events and adverse events resulting in treatment discontinuation by day 7

2.2.4 Schedule of activities

**Procedure**	**Screening suspected cases**	**Cohort confirmed COVID-19 cases:**														
		**Self-isolation/home-based care or hospitalisation as per national guidelines in each country**							**Extended hospital stay if clinically indicated otherwise as per national guidelines in each country**								
		**Treatment Period**			**Post-treatment Period**							**Close-out visit**		**Extended serological follow-up**		
	**Day 0[a]**	**Day 1**	**Day 2[b]**	**Day 3 (±1)**	**Days 4 to 6[b]**	**Day 7 (-2, +3)**	**Days 8 to 13[b]**	**Day 14 (±3)**	**Days 15 to 20[b]**	**Day 21 (±3)[c]**	**Days 22 to 27[b]**	**Day 28 (-3, +14)**	**Days 42[b] (±7) [c]**	**3 months (±0.5)**	**6 months (±1)**	**12 months (±4)**
**Screening study (cross-sectional survey source population) (screening for SARS-CoV-2)**																
Informed consent/assent	X[a]															
Demography	X															
Past and current medical conditions	X															
COVID-19 exposure history	X															
Malaria RDT or microscopy and dried blood spot	X															
Nasopharyngeal / oropharyngeal swab [d]	X															
Blood sample [f]	X															
Saliva [g]	X															
SARS-CoV-2 test results available		X[e]														
**Confirmed COVID-19 cases (COVID-19 cohort** study**)**																
**Recruitment**																
Pre-screening eligibility		X														
Prior consent discussion		X														
**Enrolment**																
Eligibility screen: Inclusion and exclusion criteria		X														
Informed consent cohort		X														
Cohort study code issued		X														

	Day 0[a]	Day 1	Day 2[b]	Day 3	Days 4 to 6[b]	Day 7 (±1)	Days 8 to 13[b]	Day 14 (±3)	Days 15 to 20[b]	Day 21 (±3)[c]	Days 22 to 27[b]	Day 28 (±3)	Days 42[b] (±5)[c]	3 months (±0.5)	6 months (±1)	12 months (±2)
**Assessments (demographic and clinical measures)**																
Demography, including contact telephone numbers		X														
Past and current medical conditions [h]		X		X		X		X		X [c]		X	X[c]			
Copy clinical/lab data from the patient card/clinic registers		X														
Physical examination		X		X		X		X		X[c]		X	X[c]			
Concomitant medications, and signs and symptoms review		X		X		X		X [i]		X[c,i]		X	X [ci]			
Daily telephone survey in between scheduled clinic visits (including dosing adherence, concomitant medications, and symptoms review)			X		X		X		X [i]							
Close-out survey (including concomitant medications, symptoms, etc.)												X [c]				
Unscheduled sick-patient clinic visits			X		X		X		X							
**Biological samples**		X														
Mid-nasal swab [j]		X		X		X		X		X [c]		X				
Blood sample [k]		X		X		X		X		X [c]		X	X[c,l]	X[k]	X[k]	X[k]
Saliva sample [g]		X		X		X		X		X [c]		X	X[c]	X	X	X
Stool sample [m]		X		X		X		X		X [c]		X				
**Nested malaria treatment trial within the COVID-19 cohort** study **(activities additional to those listed above under the COVID-19 cohort** study)																
Informed consent cohort & nested malaria treatment trial	X[n]	X														
Randomisation & allocation [n]	X[n]	X[n]														
Study therapy (AL or PA) [o]	[n]	##	##	##												
Close-out nested malaria treatment trial													X			
Dosing adherence, concomitant medications, and signs and symptoms review		X		X		X		X		X [c]		X	X [c]			
Safety assessment for (serious) adverse events		X	X	X	X	X	X	X	X	X [c]		X	X [c]			
Pharmacokinetic samples [p]				X		X		X		X [c]		X	X [c]			
SARS-CoV-2=severe acute respiratory syndrome coronavirus 2, C19=COVID-19, RDT=rapid diagnostic test, CRF=Case Record Form, PA= pyronaridine-artesunate, AL=artemether-lumefantrine																

[a] Screening for Covid-19 as part of a screening study (cross-sectional survey, source population). Screening and Day 1 evaluations may occur on the same day if the point of care antigen SARS-CoV-2 tests result is positive. This depends on the malaria status of the patient (i.e. malaria positive: Invite eligible participants to enrol in the nested malaria treatment trial on Day-0; malaria-negative: Provide the participant with the option to enrol in the COVID-19 cohort study either the same day (Day-0) or wait until the confirmatory PCR results are available on Day-1 (see [e], below)). A sub-group of participants who test negative by both SARS-CoV-2 antigen RDT and malaria RDT, and are feeling well, will be invited to participate as healthy controls.

[b] These evaluations will be conducted by telephone and/or text messaging

[c] These evaluations will be conducted among participant enrolled in the nested malaria treatment trial only

[d] Any default diagnostic swab performed by local health authorities is acceptable. Additional swabs will be taken for a SARS-COV-2 point of care antigen test.

[e] The number of days for the SARS-CoV-2 test results to become available can be longer than 1 day and may vary by site.

[f] Blood sample for SARS-CoV-2, malaria diagnostics and malaria and non-malaria serology in source population (fingerpick, 250 µL dried blood spot and malaria RDT). Consenting healthy control participants will provide a single 5 mL venous blood sample for CyTOF for B and T cell markers (fixed whole blood), Olink immunoassay (plasma) and transcriptional profiling (gene expression).

[g] Saliva sample (~1mL) for SARS-CoV-2 RNA and antigen detection and SARS-CoV-2 and malaria serology

[h] Includes questions for the WHO Ordinal Scale for Clinical Improvement and/or modified Flu-PRO PLUS

[i] Daily during the first 14 days, or until the participant is asymptomatic for two consecutive days, whichever comes last, and then on days 21, 28 and 42

[j] Mid-nasal swabs for research specific molecular detection and viral load and sequencing of SARS-CoV-2 (for later batch analysis, not for patient care)

[k] Blood sample (15mL on day-1 & 28, month 3, 6, and 12; 10mL on days 3, 7, 14, 21, 42) for anti-SARS-CoV-2 antibody titre (serum), cytokine/chemokine, inflammation and disease severity biomarker panel (serum), CyTOF for B and T cell markers (fixed whole blood), transcriptomics (gene expression), malaria and non-malaria serology (serum), malaria microscopy, malaria and non-malarial pathogen detection by molecular (e.g. PCR & TaqMAN, RDT), and (in malaria positive participants) genotyping to differentiate between recrudescence or reinfection (whole blood), biochemistry (day-1 and day-28 only), full blood count for haematology (day-1 & 28 only, or when clinically indicated), host genetics (blood pellet/dried blood spots) and QuantiFERON-TB Gold In-Tube (whole blood, at enrolment or otherwise at any other timepoint after enrolment if the QuantiFERON was missed at enrolment; a repeat QuantiFERON sample will be taken at 12m for all). In children, the total volume of blood will be 2.5mL in children weighing <10kg and 5ml in children weighing 10-<15kg.

[l] Blood sample for serology and for malaria microscopy and RDT. The day-21 & 42 visits are in the nested malaria treatment trial only.

[m] Stool sample for detection of helminth infection, SARS-CoV-2 detection and culture.

[n] Informed Consent procedures, randomization and initiation of malaria treatment may also occur on day 0 if participants test positive for malaria during the cross-sectional screening for SARS-CoV-2 by routine staff and by a SARS-CoV-2 antigen test (see [d, g], above). If the SARS-CoV-2 test results turn out to be negative, the patient will complete the 3-day treatment course as assigned but will be considered a screening failure and not followed-up any further.

[o] Study drug: ## denotes either artemether-lumefantrine (AL) twice daily, or pyronaridine-artesunate (PA) once daily (depending on group assignment). The first dose will be administered in the study clinic as directly observed therapy. All subsequent doses will be taken at home by the study participant if they are asked to self-isolate for home-based care, or by study/hospital staff for participants admitted to isolation wards.

[p] Pharmacokinetic samples: Blood (5mL) and saliva (0.5mL) sample for pyronaridine concentrations. The pharmacokinetic blood sample will be taken from the same blood draw as under 'k' above, but the total volume increased to 20mL (15mL for the above-mentioned assays under 'k' and another 5mL for pharmacokinetics) on Day-1 and Day-28, and 15mL (10mL + 5mL) on days 3, 7, 14, 21, and 42. Of the 5mL for the pharmacokinetics, 2.5mL will be stored as whole blood, and the remaining 2.5ml will be processed and stored as plasma. Children under the age of assent (<12 years in Burkina Faso and <13 years in Kenya) will be excluded from pharmacokinetic sampling.

3 INTRODUCTION

3.1 BACKGROUND INFORMATION

The novel coronavirus SARS-CoV-2, first reported in late 2019, is currently the cause of a global pandemic of the coronavirus disease 2019 (COVID-19). In Africa, COVID-19 has the potential to cripple the continent's fragile healthcare systems and be devastating economically. As of writing, there is no proven effective chemoprophylaxis, no proven anti-viral treatment, and no vaccine to mitigate or prevent infection with SARS-CoV-2. While the risk groups for severe COVID-19 identified in resource-rich countries are male, older adults, obesity, diabetes, hypertension, and people of any age with serious underlying medical conditions,[1, 2, 3, 4, 5] little is known about risk factors in malaria-endemic countries. Africa has a relatively young population, but a high prevalence of people living with compromised immune systems, such as those with poorly controlled HIV infection, tuberculosis, or acute and chronic malnutrition. African populations are also more frequently exposed to infectious diseases, such as malaria, that may exacerbate or dampen the inflammatory responses associated with COVID-19.

Malaria is endemic in most of sub-Saharan Africa. In sub-Saharan Africa, young children often have acute (symptomatic) malaria when infected with *P. falciparum* parasites. Older children and adults living in areas of moderate to high malaria transmission often have asymptomatic circulating blood-stage *P. falciparum* parasites. It is unknown whether immune-modulation associated with symptomatic or chronic malaria infections are risk factors for the severity of COVID-19, or high viral load and longer duration of virus shedding, both potential contributors of onwards spread of SARS-CoV-2.[6] Malaria is also one of the main causes of moderate and severe anaemia affecting the body's oxygen-carrying capacity and placing young children at risk of tissue hypoxia and severe COVID-19, and a contributor to malnutrition,[7] potentially further compromising a child's ability to mount an efficient immune response to SARS-CoV-2.[6]

Chronic malaria could also be potentially protective against severe COVID-19. Although asymptomatic infections have been recognised to induce a level of immunologic hypo-responsiveness that may impede the development of protective immune responses to other infections,[8, 9] including to SARS-CoV-2, a hyporesponsive immune system may also provide partial protection from severe COVID-19 if it reduces the risk cytokine storm syndrome resulting from hyper-inflammation.[6] This has been observed with other respiratory virus infection. For example, malaria-induced immunomodulation has been shown to be protective against respiratory distress associated with influenza, in hospitalized children in Kenya.[6, 10] There is also some suggestion from murine models that malaria infection can lead to reduced clinical symptoms by reducing the recruitment of cellular inflammatory components to the lungs of mice infected with pneumovirus (to model infections with the human respiratory syncytial virus (hRSV). However, viral control was also impaired, leading to increased viral dissemination.[6, 11]

Malaria could also potentially enhance the coagulopathy seen with SARS-CoV-2 infection,[6] which can manifest as venous thromboembolism and arterial thrombotic complications (including pulmonary embolism and stroke), disseminated intravascular coagulation (DIC) with pulmonary haemorrhage and thrombosis.[12, 13, 14] Thrombocytopenia is another potential feature of COVID-19 and is associated with worse outcomes.[12] Malaria is also associated with a pro-coagulant state, with activation of the coagulation cascade, mediated by TNF-alpha and IL-6, proportional to disease severity.[15, 16] Thrombocytopenia develops in 60–80% of malaria cases,[16] and lysis of activated platelets, along with

tissue factor released from damaged vascular endothelial cells, promotes the pro-coagulant state,[15] similar to the proposed mechanism in COVID-19.[6]

Some of the existing antimalarial drugs, including the 4-aminoquinoline antimalarials chloroquine, hydroxychloroquine, amodiaquine (AQ), its main metabolite desethylamodiaquine, and pyronaridine, another quinoline-related compound structurally related to chloroquine, exhibit significant antiviral activity against SARS-CoV-2 in some cell lines in-vitro (Tim Wells, Medicines for Malaria Venture (MMV), personal communications).[17, 18] This has generated a widespread interest that they may provide clinical benefits in COVID-19 patients. However, the evidence base is growing rapidly, and the small number of trials that have evaluated chloroquine or hydroxychloroquine in confirmed COVID-19 patients have yielded mixed results.[18, 19] Recent unpublished data from Epithelix using primary nasal epithelial cell lines also suggests that the anti-viral effects on SARS-CoV-2 of these quinoline antimalarials, unlike Remdesivir, may depend on the cell lines tested in-vitro (Tim Wells, personal communication at the WHO-Workstream 1 meeting (May 19, 2020). More cell culture data are expected to become available in the coming months.

3.2 Problem statement and Justification for the study

3.2.1 Is malaria a risk factor for COVID-19 disease severity?

The rationale for this study is three-fold. First, it is critical to determine whether malaria infection is found to be a positive or negative risk factor for severe COVID-19 or its transmission. Even small changes in the risk of severe outcomes due to coinfections could result in substantial changes in the impact and epidemiology of COVID-19 in LMICs.[6] The resulting morbidity and mortality from the pandemic may expand beyond predominantly older adults and those with underlying risk factors (heart and lung disease, diabetes, obesity, immuno-compromised) to a broader age range that includes infants, children, and younger adults. Furthermore, it would suggest that successfully controlling malaria would be needed to control the COVID-19 epidemic as it may have a positive, mitigating effect on COVID-19 severity and transmission. It would reinforce the importance, and potential expansion of malaria control programs to avoid similar scenarios as during the Ebola outbreak and diversion of resources and antimalarials for COVID-19 treatment. By contrast, if partial immunosuppression from chronic malaria is found to be protective against severe COVID-19 resulting from hyper-inflammation, then aggressive malaria control such as mass drug administration may paradoxically increase severe COVID-19 in a subgroup of cases. We will, therefore, follow a cohort of COVID-19 patients with and without malaria with a primary objective, to understand better how co-infection of SARS-CoV-2 and *Plasmodium falciparum* impacts disease progression compared to COVID-19 patients without malaria.

3.2.2 Does co-infection with malaria affect the rate of seroconversion and/or seroreversion to SARS-CoV-2 infection?

Second, it is also unknown if malaria affects the type (IgG, IgA, IgM) or avidity of antibody response to SARS-CoV-2 infection and time to seroconversion and rate of loss of antibodies or seroreversion and quality (avidity) of antibody responses. Similar considerations will apply to immune responses to future COVID-19 vaccines and whether vaccine efficacy will differ in the presence of malaria parasitaemia and the subsequent clearance of these malaria infections by appropriate antimalarial treatment.

3.2.3 Does the choice of antimalarial treatment affect disease progression or the rate of seroconversion to SARS-CoV-2 infection?

Third, it is also important to know if the choice of antimalarial to treat malaria in co-infected patients affects the rate of seroconversion or disease progression. Most countries in sub-Saharan Africa, including Kenya and Burkina Faso, use artemether-lumefantrine as the first-line antimalarial for the case-management of uncomplicated malaria. Lumefantrine belongs to the group of aryl-amino alcohols like quinine, mefloquine, and halofantrine and does not have any anti-viral properties in-vitro (Tim Wells, MMV personal communication). Pyronaridine, as the artemisinin-based combination therapy (ACT) pyronaridine-artesunate (PA), is increasingly being rolled-out in sub-Saharan Africa as second-line treatment, or as an alternative to artemether-lumefantrine as part of a rotational or mosaic first-line treatment strategy to delay the onset of the development of drug resistance. It remains to be determined if antimalarial treatment with pyronaridine-artesunate provides any additional clinical benefit compared to treatment with artemether-lumefantrine in COVID-19 patients who are co-infected with malaria and require antimalarial treatment.

In humans, pyronaridine is slowly eliminated and has extensive tissue binding, including in the lung. PK/PD modelling suggests that pyronaridine when provided as a standard 3-day treatment course of pyronaridine-artesunate, could potentially provide 28 days of anti-viral cover based on their predicted pulmonary exposure (Dr Ghaith Aljayyoussi and Prof Giancarlo Biagini, personal communications, LSTM).

Figure 1 Predicted Pyronaridine exposure in plasma (red line) and lung (blue line). Plasma exposure based on reported pk parameters in the literature and lung exposure predicted as a ratio to plasma exposure based on PBPK modelling.

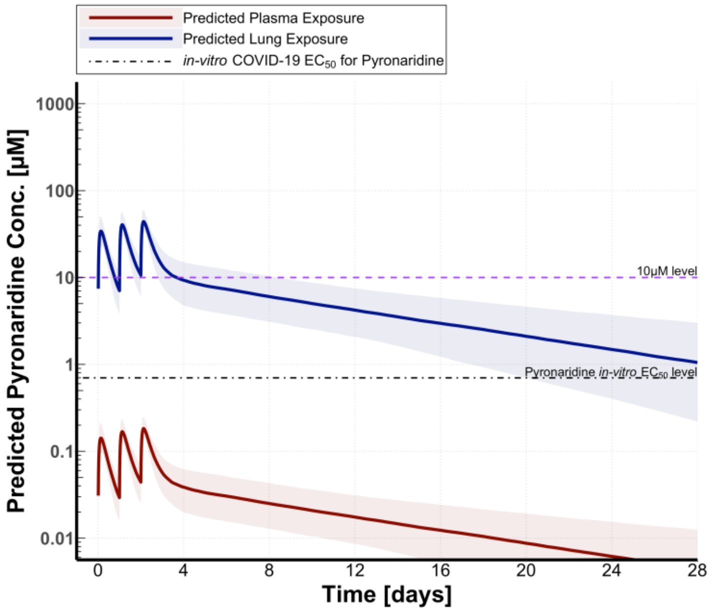

Assumptions of the study include that the in vitro effects of pyronaridine against SARS-CoV-2 will translate to an in vivo effect and a benefit in human participants and that pyronaridine reaches concentrations in human tissues, including the lungs, that have a viral suppressive effect. However, the exact distribution of pyronaridine within the respiratory tract, and whether these in vitro findings will translate into clinical benefit, is unknown.

Quinoline antimalarials may also have unintended consequences in reducing the seroconversion rate to the SARS-CoV-2 virus. Importantly, chloroquine has been shown to markedly decrease the seroconversion rate in persons immunised with the rabies vaccine resulting in 70% lower antibody titres[20] through its potent anti-inflammatory effects by downregulating the cell-mediated immune response through its interaction with Toll-like receptors[21] and affecting lysosomal degradation.[22, 23]

This raises a question whether similar effects could occur with other quinoline antimalarials like pyronaridine when used for the treatment of malaria of COVID-19 patients. We aim, therefore, to determine if malaria is a direct or indirect risk factor for mounting an appropriate immune response to SARS-CoV-2 and whether this is affected by the choice of treatment.

3.2.4 Rationale for pyronaridine-artesunate

Pyronaridine has some similarity in structure to chloroquine, and similar antiviral activity in-vitro against SARS-CoV-2 compared to chloroquine and hydroxychloroquine.[24] Pyronaridine is active against SARS-CoV-2 in Vero76 cell assays in vitro with plasma/blood EC50 0.7 µ (Tim Wells, MMV personal communication). Moreover, Physiologically Based Pharmacokinetic (PBPK) modelling suggests that pyronaridine has excellent lung penetration, with a Cmax lung/blood ratio of 44, supporting the exploration of pyronaridine as a potential antiviral drug for COVID-19. There is very little information about the antiviral activity against SARS-CoV-2 of artesunate[25], but because the artemisinin derivates are eliminated from the body within hours, artesunate is unlikely to have a clinically relevant impact on viral replication. The inclusion of artesunate is driven by the availability of the approved fixed-dose combinations of pyronaridine-artesunate with established safety, tolerability and efficacy in malaria. Pyronaridine has also shown promise in protecting mice from Ebola virus infection and has potent immunomodulatory properties in-vivo in mice.[26]

Shin Poong Pharm. Co., Ltd, the manufacturer of pyronaridine-artesunate (Pyramax®), will evaluate pyronaridine-artesunate for the treatment of SARS-CoV-2 infection in symptomatic outpatients with mild disease in South Africa (http://www.koreabiomed.com/news/articleView.html?idxno=8200). This is an exploratory, randomized, single-centre, open-label study of four different experimental treatment arms versus standard of care and scheduled to start later in 2020. Artesunate-amodiaquine (AS-AQ) is an alternative candidate, but discussions with the respective MoH's in Kenya and Burkina suggest a preference for pyronaridine-artesunate because of tolerance, availability in each country and intended roll-out as first or second-line treatment in parts of the country in 2020-2021. Furthermore, in Burkina Faso, the use of amodiaquine is restricted to Seasonal Malaria Chemoprevention (SMC) (in combination with SP). Lastly, pyronaridine is the only quinoline antimalarial that is not associated with QT prolongation,[27] which is a potential advantage over chloroquine and amodiaquine, particularly when used for the treatment of COVID-19 in combination with other QT-prolonging drugs like azithromycin.

3.3 Null Hypotheses

Our null hypotheses are:

1. Malaria is not a direct or indirect risk factor affecting COVID-19 severity, duration of disease, and viral shedding
2. Malaria is not a direct or indirect risk factor for mounting an appropriate immune response to SARS-CoV-2
3. Pyronaridine-artesunate does not affect the seroconversion rate to SARS-COV-2 or ameliorate disease severity and accelerate recovery from COVID-19 in malaria co-infected individuals, relative to the standard first-line therapy of malaria with artemether-lumefantrine.

4 OBJECTIVES AND ENDPOINTS

4.1 SCREENING STUDY (CROSS-SECTIONAL SURVEY SOURCE POPULATION) (N~ 4,720)

**Primary objective**	**Primary endpoint**
• To determine if malaria infection is predictive of the prevalence of SARS-CoV-2, adjusted for other risk factors of SARS-CoV-2 infection	• Cross-sectional comparison of the proportion of participants with SARS-CoV-2 in the source population among participants with and without malaria infection expressed as the prevalence ratio for COVID-19 (95% CI)
**Secondary objectives**	**Secondary endpoints**
• To determine the demographic and clinical risk factor for the prevalence of SARS-CoV-2 infection	• Cross-sectional comparison of demographic and clinical risk factors of SARS-CoV-2 in the source population expressed as a prevalence ratio for COVID-19 (95% CI)

4.2 COVID-19 COHORT (N~708)

**Primary objective**	**Primary endpoint**
• To determine if malaria infection affects COVID-19 severity	• Comparison of the severity of COVID-19 by day-28 in cases with and without malaria using the WHO clinical progression scale
**Secondary objectives**	**Secondary endpoints**
• To determine if malaria infection affects COVID-19 disease presentation and duration	• Symptoms and duration of COVID-19 by day-28 in cases with and without malaria
• To determine if malaria infection in COVID-19 patients alters the antibody response to SARS-CoV-2 infection	• Cumulative seroconversion rates* (total antibody, IgG, IgM, IgA) by days 7, 14, 21, 28, and total antibody, IgG, IgM, IgA antibody titres against SARS-CoV-2 by day-28 expressed as the geometric mean, maximum, and change from baseline<br>• Seroreversion rates for IgG, IgM, IgA and geometric mean antibody titres against SARS-CoV-2 by month 3, 6 and 12
• To determine if malaria infection in COVID-19 alters the inflammatory, genomic, and cellular immune responses to SARS-CoV-2 infection	• Multiplex serum cytokine markers; Transcriptional profiling (gene expression) of whole blood; Fixed whole blood for T and B cell markers by day-28
• To determine if malaria infection in COVID-19 alters the viral load or duration of SARS-CoV-2 infection	• Median viral load of SARS-CoV-2 detected from mid-nasal swabs by PCR<br>• Cumulative incidence of SARS-CoV-2 clearance (defined as the proportion of participants with negative nasal swabs) by days 7, 14, 21 and 28<br>• Time to clearance of nasal SARS-CoV-2, defined as negative SARS-CoV-2 RNA PCR tests (swabs collected on days 1, 3, 7, 14, 21 and 28)

4.3 Nested malaria treatment trial in the COVID-19 cohort study (N~142)

**Primary objective**	**Primary endpoint**
• To determine if pyronaridine-artesunate compared to artemether-lumefantrine reduces early SARS-CoV-2 viral shedding	• Incidence of SARS-CoV-2 clearance (defined as the proportion of participants with a negative nasal swab) on Day 7 after the start of treatment
**Secondary objectives**	**Secondary endpoints**
• To determine if pyronaridine-artesunate compared to artemether-lumefantrine alters the viral load and/or the duration of SARS-CoV-2 viral shedding	• Median viral load of SARS-CoV-2 detected from mid-nasal swabs by PCR<br>• Cumulative incidence of SARS-CoV-2 clearance (defined as the proportion of participants with negative nasal swabs) by days 14, 21 and 28<br>• Time to clearance of nasal SARS-CoV-2, defined as negative SARS-CoV-2 RNA PCR tests (swabs collected on days 1, 3, 7, 14, 21 and 28)
• To determine if treatment of malaria infection with pyronaridine-artesunate in COVID-19 patients alters the antibody response to SARS-CoV-2 infection compared to treatment with artemether-lumefantrine	• Cumulative seroconversion rates (IgG, IgM, IgA) by days 7, 14, 21 and 28, and IgG, IgM, IgA antibody titres against SARS-CoV-2 by day-28 expressed as the geometric mean, maximum, and change from baseline
• To determine if treatment of malaria infection with pyronaridine-artesunate in COVID-19 alters the inflammatory, genomic, and cellular immune responses to SARS-CoV-2 infection compared to treatment with artemether-lumefantrine	• Multiplex serum cytokine markers; Transcriptional profiling (gene expression) of whole blood; Fixed whole blood for T and B cell markers by day-28
• To compare the clinical and parasitological efficacy of pyronaridine-artesunate versus AL in COVID-19 patient coinfected with malaria parasites.	• The proportion with early treatment failure, late clinical failure, late parasitological failure or an adequate clinical and parasitological response. Recrudescence will be differentiated from new infection by genotyping of malaria parasites by day 42
• To determine if pyronaridine-artesunate compared to artemether-lumefantrine alters COVID-19 disease progression and severity	• COVID-19 symptoms, duration and severity as defined by a severity index score by day 28<br>• The proportion of days with a fever after randomization<br>• The proportion of days with respiratory symptoms after randomization
• To test the safety of pyronaridine-artesunate compared to artemether-lumefantrine in COVID-19 patient coinfected with malaria parasites	• The cumulative proportion of treatment-related adverse events and serious adverse events and adverse events resulting in treatment discontinuation by day 7
* The cumulative seroconversion rate is defined as the proportion of participants with a positive antibody test per period among those with a negative antibody test on enrolment.	

5 STUDY DESIGN

Figure 2: Design overview

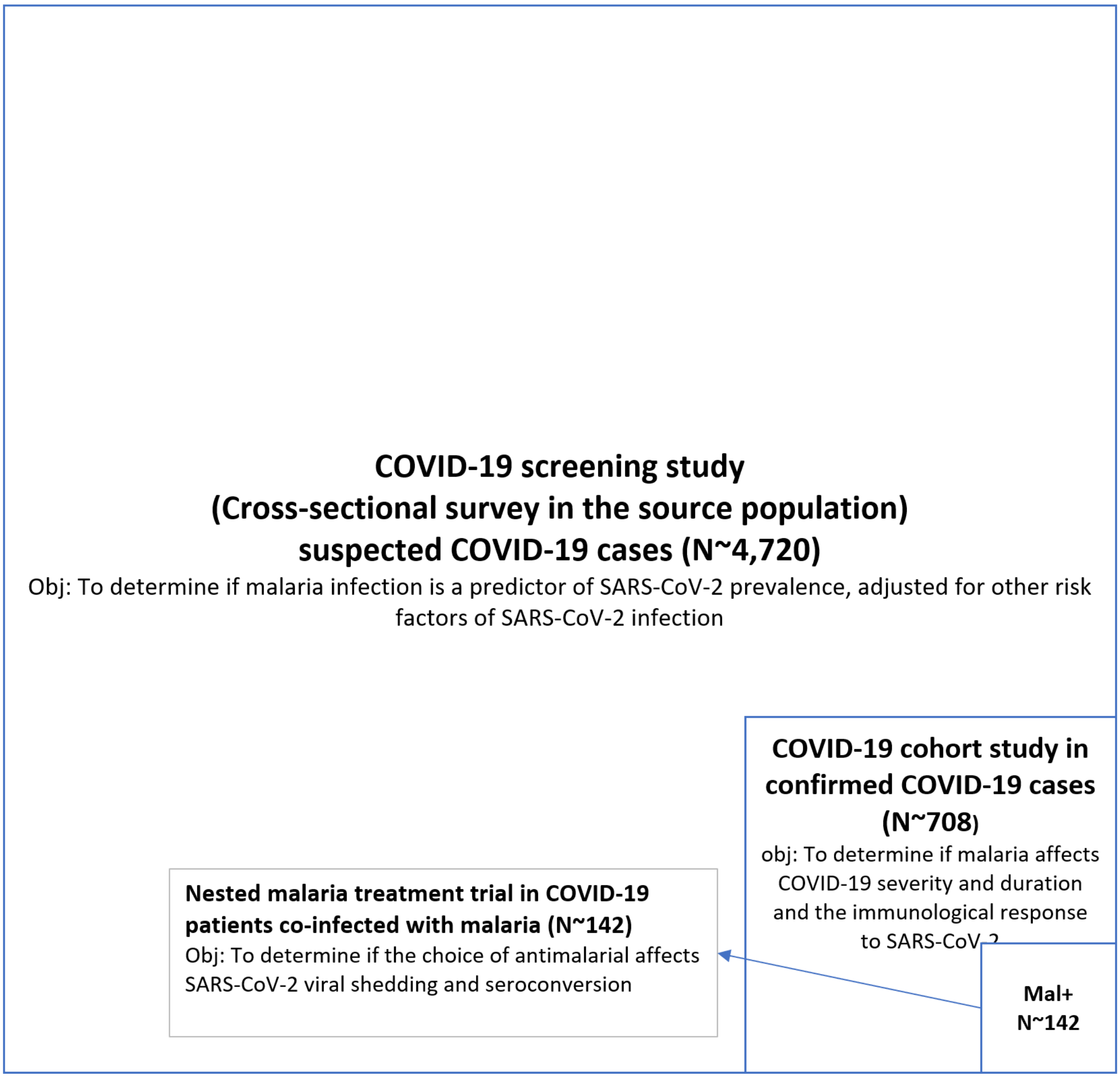

COVID-19=Coronavirus disease 2019, SARS-CoV-2=Severe acute respiratory syndrome coronavirus 2, Mal=malaria infection detected by a rapid diagnostic test or microscopy

Figure 3: Design overview

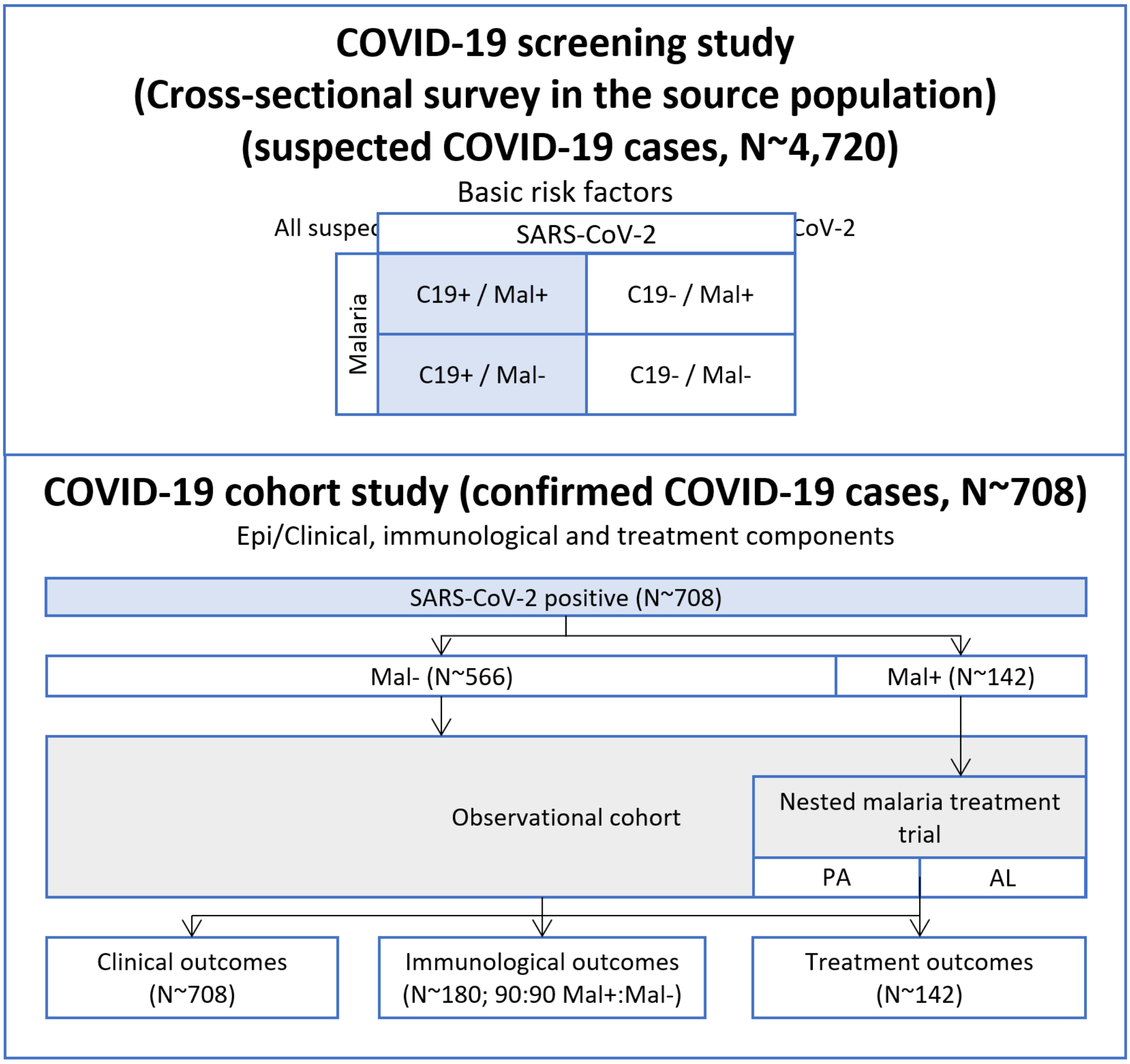

COVID-19=Coronavirus disease 2019, SARS-CoV-2=Severe acute respiratory syndrome coronavirus 2, Mal=malaria infection detected by a rapid diagnostic test or microscopy, PA=pyronaridine-artesunate, AL=artemether-lumefantrine

5.1 OVERVIEW STUDY DESIGN

The overarching goal of this study is to assess if malaria alters the severity and duration of COVID-19 among a predominantly outpatient population with SARS-CoV-2 infection to inform public health control strategies. We propose to enrol a prospective COVID-19 cohort study among *confirmed* COVID-19 cases selected from a source population consisting of all *suspected* cases tested for SARS-CoV-2. We will conduct a nested malaria treatment trial among the COVID-19 cohort study participants who are co-infected with malaria at enrolment.

5.1.1 Screening study (cross-sectional survey source population) (N~4,720)

The screening study (cross-sectional survey, source population) from which a cohort of all ages will be enrolled will consist of all *suspected* COVID-19 cases tested for SARS-CoV-2 in quarantine wards/centres, COVID-19 test centres, other clinics, or as part of contact tracing or targeted testing in western Kenya and Burkina Faso. It will thus include both SARS-CoV-2 positive and negative participants with and without malaria. The data will allow for a cross-sectional description of basic

demographic and clinical data using a relatively large sample size and to determine if malaria is a risk factor for the prevalence and severity of COVID-19.

5.1.2 COVID-19 cohort study (N~708)

All SARS-CoV-2 positive participant from the source population will be eligible for enrolment in the COVID-19 cohort study of *confirmed* COVID-19 cases regardless of their malaria status. The COVID-19 cohort study has a clinical disease progression component and an immunological component. It also has a nested malaria treatment trial in a subgroup of participants that are co-infected with malaria (see 5.1.3, Nested malaria treatment trial within the COVID-19 cohort study, below). The primary aim is to determine if symptomatic or asymptomatic malaria infection affects the clinical course of COVID-19. Some elements of the immunological component involve all participants in the COVID-19 cohort study (e.g. antibody responses), and some involve a subset of approximately 180 participants (e.g. cellular responses) from whom more detailed immunologic profiling of serial samples of serum and whole blood will be analysed and compared between participants with and without malaria. All cohort participants will be followed on days 0, 3, 7, 14, and 28 to monitor disease progression, cumulative seroconversion rates, viral load, and viral carriage. Additional samples will be taken at 3, 6, and 12 months to determine the duration of the protective antibody responses to the SARS-CoV-2 virus.

All COVID-19 patients without malaria will be treated with the recommended standard of care (SOC) at the time of enrolment. The SOC will follow the national guidelines in each country and are subject to change (see 7.1.1, Interventions, COVID-19 cohort study, page 33). All COVID-19 patients with malaria will be enrolled in the nested malaria treatment trial and have additional samples taken at day 21 and day 42, as described in section 5.1.3, below.

5.1.3 Nested malaria treatment trial within the COVID-19 cohort study (N~142)

In addition, we will conduct a nested exploratory, 6-week, open-label, 2-arm, randomised controlled nested malaria treatment trial in the COVID-19 patients co-infected with malaria with mild disease who are able to take oral medication. We will compare pyronaridine-artesunate and artemether-lumefantrine to assess whether, in COVID-19 patients with malaria, treatment with quinoline antimalarials (pyronaridine) affects the seroconversion rate or time to seroconversion to SARS-CoV-2 or reduce viral load and duration of shedding relative compared to patients receiving AL. The patients enrolled in this nested malaria treatment trial will have an additional follow-up visit at days 21 and 42.

The follow-up schedule in the treatment study will be identical to that in COVID-19 patients without malaria to allow for comparison between disease progression between COVID-19 with and without malaria.

5.2 PARTICIPANT AND STUDY COMPLETION

A participant is considered to have completed the study if he/she has completed all phases of the study, including the last scheduled procedure shown in the Schedule of Activities (SoA).

This study will be considered completed when a sufficient number of participants complete the study to enable appropriate evaluation of the primary endpoint.

6 STUDY SETTING AND POPULATION

6.1 STUDY SETTING

6.1.1 Kenya

As of writing (September 10, 2020), there are 35,460 COVID-19 cases reported cumulatively in Kenya. The fieldwork will be conducted as part of the ongoing collaboration between KEMRI, CDC, and LSTM in the following hospitals in western Kenya which are located in areas with intense malaria transmission and local transmission of COVID-19: Kisumu County: Primary Hospital(s): Jaramogi Oginga Odinga Teaching and Referral Hospital (JOORTH), Kisumu County Referral Hospital. Secondary Expansion Hospitals: Lumumba Health Centre, Ahero County Hospital, KMET Cockran Clinic, St Elizabeth Mission Hospital, St Monica's Mission Hospital, Rabuor Health Centre; Busia County: Primary Hospital(s): Alupe sub-County Hospital, Secondary Expansion Hospitals: Busia County Teaching and Referral Hospital, Busia GK Prison Dispensary; Siaya County: Primary Hospital(s): Bondo sub-County Hospital, Secondary Expansion Hospitals: Siaya County Teaching And Referral Hospital, Ambira sub-County Hospital; Migori County: Macalder (Nyatike) sub-County Hospital, Migori County Teaching and Referral Hospital; Secondary Expansion Hospitals: Kehancha sub-County Hospital, Migori GK Prison Dispensary, Isebania sub-County Hospital. Other hospitals in the previous Nyanza and western Provinces may be considered if local outbreaks of Covid-19 start to occur in their catchment areas.

6.1.2 Burkina Faso

As of writing (September 10, 2020), there are 1,476 COVID-19 cases reported cumulatively in Burkina Faso. In Burkina, malaria transmission is seasonal, reaching a peak in October/ November of 30% RDT positivity in all ages. The study will be conducted in hospitals designated for care and treatment of COVID-19 cases in Ouagadougou (currently three hospitals) and, if sample size requirements dictate, Bobo Dioulasso. The work will be coordinated by the Groupe de Recherche Action en Santé, Ouagadougou.

6.2 STUDY POPULATION

6.2.1 Screening study (cross-sectional survey source population)

6.2.1.1 Inclusion criteria

- Patients attending COVID-19 test centres or other clinics conducting testing for COVID-19
- Patient population admitted to the COVID-19 isolation/quarantine facilities
- Contacts of confirmed or suspected cases as part of contact tracing
- Populations as part of targeted testing of high-risk populations (e.g., frontline health care workers)

6.2.1.2 Exclusion criteria

- Unwilling or unable to provide informed consent/assent

Participants may be re-screened if they are found not to have COVID-19 at the time of the initial screening, but develop a further, and separate acute illness suggestive of COVID-19 at a later stage again or as part of further contact tracing.

6.2.2 COVID-19 cohort study

6.2.2.1 Inclusion criteria

- Laboratory confirmed SARS-CoV-2 infection, with positive molecular test results within the past 72 hours* or a positive rapid diagnostic antigen test
- Aged >=6 months **
- Resident in the study area
- The participant or caretaker is willing and able to give informed consent or assent with parent/guardian informed consent for participation in the study
- Agrees not to self-medicate with chloroquine, hydroxychloroquine or other drugs with potential anti-SARS-CoV-2 properties without prior discussion with medical study staff
- Contactable by phone for follow-up permitting real-time, reliable information

6.2.2.2 Exclusion criteria

- Unwilling or unable to provide informed consent/assent
- The participant is judged by the Investigator to be at significant risk of failing to comply with the provisions of the protocol as to cause harm to self or seriously interfere with the validity of the study results
- Inability/unlikely to be in the study area for the duration of the 28-day follow-up period

* We anticipate that most tests results will be available within 24 hours. The maximum of 72 hours is a target and can be increased to 120 hours (5 days) in sites that experience significant delays related to routine testing for SARS-CoV-2 by the local health authorities.
** Children < 6 months will be excluded because of the inconvenience of repeated blood sampling and because they have a different clinical presentation of malaria than older children, as they are partially protected from developing clinical malaria due to the protective effect of maternal immunity after birth and through factors that inhibit parasite growth, such haemoglobin F (HbF).[28]

6.2.3 Nested malaria treatment trial

6.2.3.1 Inclusion criteria

- Fulfils all inclusion criteria for the COVID-19 cohort study
- Uncomplicated malaria, defined as able to take oral medication
- Bodyweight ≥5kg
- Confirmed malaria infection by RDT (HRP2 or pLDH) or microscopy

6.2.3.2 Exclusion criteria

- Fulfils any of the exclusion criteria for the COVID-19 cohort study
- Pregnant or lactating women
- Severe disease requiring parenteral treatment
- Currently receiving, or recently received (within the last 28 days) pyronaridine-artesunate or artemether-lumefantrine
- Received any other antimalarials in the last three days
- Inability/unlikely to be in the study area for the duration of the 42-day follow-up period
- Known hypersensitivity or specific contraindication to the use of any of the study drugs in the treatment arms
- Known chronic kidney disease (signs or symptoms of stage IV renal impairment or receiving dialysis)
- Known liver cirrhosis (Child-Pugh Class B or greater) or signs or symptoms of severe hepatotoxicity

HIV and TB are not exclusion criteria, and details of any anti-retroviral and anti-TB medication will be documented on the CRFs (see 10.2.4, HIV testing and TB testing, page 46).

6.2.4 Co-enrolment guidelines

Participants may be co-enrolled in other research studies if these are observational studies only. Any other exception requires the approval of the Principal Investigators; if a participant clinically worsens, such as requiring hospitalisation, it is expected that an exception will be automatically granted and participation in other treatment studies permitted. The study team should be consulted for co-enrolment in studies that do not meet this guidance or if there are questions about eligibility for co-enrolment. For any co-enrolled study, the total volume of blood samples collected across the two studies should not exceed current Red Cross phlebotomy guidance.

7 INTERVENTIONS

If cohort participants are co-infected with malaria, they will be enrolled in the nested malaria treatment trial if they fulfil the eligibility criteria. In each country, participants will be enrolled in one of two treatment arms (see below) and followed weekly for six weeks (42 days). The quarantine and isolation practices and contact tracing will follow the local practices and guidelines in each country.

COVID-19 patient co-infected with malaria will be randomised to receive either a weight-based standard 3-day course of artemether-lumefantrine or a weight-based standard 3-day treatment course of pyronaridine-artesunate. Dosing will follow the standard WHO dosing guidelines.

7.1 TREATMENTS ADMINISTERED

7.1.1 COVID-19 cohort study

7.1.1.1 Treatment and care of COVID-19 patients who are malaria-negative at enrolment

All COVID-19 patients without malaria will be treated with the recommended standard of care (SOC) at the time of enrolment. The SOC will follow the national guidelines in each country and are subject to change.

In Kenya, this consists of isolation and supportive treatment, depending on the severity of the disease. This will follow the local guidelines. The most recent guidelines from June 2020 stipulate home-based isolation and home-based care for asymptomatic or paucisymptomatic patients with COVID-19 if they fulfil certain criteria (e.g. low-risk patient category, availability of a dedicated room and a caretaker in the house, absence of any high-risk person in the household). If they don't meet the self-isolation and home-based care criteria, they are admitted for mandatory isolation. Confirmed symptomatic cases are admitted to isolation wards for supportive management. It is anticipated that Government quarantine and isolation guidelines may be updated during the course of the pandemic and by geographical region (i.e. depending on the patient load and local capacity of the health system). We will follow any updates to these national and local (county) guidelines.

Mild cases are provided with symptomatic treatment such as antipyretics for fever, e.g. paracetamol 500mg -1gm six hourly for adults and for children 10-15mg/kg 6 hourly taken orally in addition to counselling about signs and symptoms of complications and to seek urgent care through national referral systems upon onset of severe disease. Severe cases, i.e. those presenting with severe acute respiratory illness (SARI) are admitted and provided with supportive care such as supplemental oxygen, fluid, empiric antimicrobials such as amoxicillin, erythromycin, azithromycin and/or

intensive care. In non-routine cases, corticosteroids may be indicated as in the treatment of viral pneumonia.

In Burkina Faso, this currently consists of isolation at home for potential cases of COVID-19 until the test results are known, unless they have symptoms of severe diseases requiring hospitalisation. Asymptomatic or paucisymptomatic confirmed cases are requested to self-isolate, where cases with moderate to severe COVID-19 are admitted.

7.1.1.2 Treatment and care of COVID-19 patients who are malaria-positive at enrolment

The COVID-19 patients with malaria who are eligible for enrolment in the nested malaria treatment trial will receive the same care as for malaria-negative COVID-19 patients described in section 7.1.1.1, Treatment and care of COVID-19 patients who are malaria-negative at enrolment, above. In addition, they will receive treatment for malaria as described in section 7.1.2, Nested malaria treatment trial, below.

7.1.2 Nested malaria treatment trial

Table 1 Dosing schedule nested treatment study

**Study Treatment**	Artemether-lumefantrine (AL)	Pyronaridine-artesunate (PA)
**Dosage Formulation**	5 to <15kg: Dispersible tablets 20/120 mg A/L,<br><br>>=15 kg: standard tablets of 40/240mg A/L	5 to<20kg: granules for oral suspension of 60/20 mg P/A #<br><br>>=20 kg: Standard tablets of 180/60 mg of P/A
**Route of Administration**	oral	oral
**Dosing Instructions**		
**Manufacturer**	*Coartem*® from Novartis or another WHO-prequalified produce of artemether-lumefantrine	*Pyramax*® from Shin Poong Pharm. Co., Ltd, Korea

For children weighing between 5 kg to under 20 kg, a granule formulation will be used. Each sachet of *Pyramax*® granules for oral suspension contains 60 mg pyronaridine tetraphosphate and 20 mg artesunate.

7.2 RISKS TO THE PARTICIPANTS

7.2.1 Risks associated administration with artemether-lumefantrine

Artemether-lumefantrine is the standard of care for malaria in both Kenya and Burkina Faso and is generally very well tolerated.

The WHO's Malaria Treatment Guidelines, third edition report the following under its safety section for artemether-lumefantrine on page 216:[29]

7.2.1.1 Adverse events

Artemether-lumefantrine has a wide therapeutic index and is generally well-tolerated, with reported side-effect such as nausea, dizziness and headache that are not easily distinguishable from symptoms of acute malaria. Artemether-lumefantrine does not significantly prolong the QTC interval.[29]

7.2.1.2 Contraindications

Artemether-lumefantrine should not be administered to patients with known hypersensitivity to either artemether or lumefantrine, or severe hepatic or renal impairment.[29]

7.2.1.3 Cautions

Artemether-lumefantrine has not been studied extensively in patients >65 years or children weighing < 5kg, so these patients should be monitored closely taking this medication.[29]

The manufacturer advises against the administration to patients with congenital or clinical conditions resulting in QTc prolongation, a family history of congenital long QT syndrome or sudden death or those with electrolyte abnormalities such as hypokalaemia and hypomagnesaemia, which may affect cardiac conductively, although there is no evidence for iatrogenic toxicity in these groups.[29]

7.2.2 Risks associated with pyronaridine-artesunate

Pyronaridine-artesunate was approved as *Pyramax®* by the European regulator EMA in 2012, and the granules for the treatment of children in 2015. Pyronaridine-artesunate was also approved by WHO for the treatment of malaria in October 2019.[31]

The following information is extracted from the summary of product characteristics for *Pyramax®*.

7.2.2.1 Contraindications

- Known hypersensitivity to pyronaridine or artesunate or any component of the formulation
- Patients with clinical signs or symptoms of hepatic injury (such as nausea and/or abdominal pain associated with jaundice) or known severe liver disease (i.e. decompensated cirrhosis, Child-Pugh stage B or C). There is no information on dosing in patients with hepatic impairment. Due to its potential liver toxicity Pyramax is contraindicated in patients with signs of hepatic impairment or known significant liver function test abnormalities.
- Severe renal impairment. There is no information on dosing patients with severe renal impairment. Although excretion via faeces was the main route of elimination of pyronaridine-related material in a human mass balance study, significant urinary excretion was also observed. Pyramax is, therefore, contraindicated in the case of severe renal impairment.

7.2.2.2 Cautions

7.2.2.2.1 hepatoxicity

Pyramax has been associated, in some patients, with transient increases in liver enzymes without clinical signs. Pyramax is therefore contra-indicated in the case of underlying hepatic injury, clinical signs or symptoms of hepatic injury or known severe liver disease (see section 7.2.1.2, Contraindications, above). If a patient is already known to have elevated transaminases, the use of Pyramax is not recommended.

Patients should be advised of the clinical signs and symptoms of hepatotoxicity in order to monitor closely if such signs or symptoms occur, especially in the first two weeks after Pyramax intake. It is recommended that, in patients who exhibit symptoms of hepatotoxicity following treatment with Pyramax, the liver function tests be monitored if possible, until normalisation.

7.2.2.2.2 Co-infections

No data are available in patients with co-infections (HBV, HCV, HIV); those receiving co-administration of drugs known to be associated with mitochondrial toxicity (i.e. valproate,

antiretroviral drugs), use of herbal medicines, patients with malnutrition or patients with other hepatic underlying conditions (i.e. ethanol intoxication, hepatic steatosis). Particular caution is advised in these patients regarding the risk of liver toxicity since these risk factors, also including co-administration of paracetamol, might produce a cumulative effect on the liver. Enhanced surveillance is warranted in young children in case of malnutrition.

7.2.2.2.3 HIV

The safety and effectiveness of pyronaridine-artesunate for the treatment of malaria in patients with HIV/AIDS has been studied in fifteen patients, with no apparent differences in efficacy or safety compared with HIV negative patients (Shin Poong and Stephane Duparc, MMV, personal communications, unpublished observations).

7.2.2.2.4 Severe diarrhoea and vomiting

In patients with acute malaria who present with severe diarrhoea and vomiting, alternative therapy should be considered. If Pyramax is used in these patients, the parasite load should be closely monitored.

7.2.2.2.5 Allergic reactions

Pyramax® contains tartrazine and sunset yellow as colouring agents which may cause allergic reactions which may manifest as flushing, the appearance of wheals/urticarial, breathlessness, faintness and/or fall in blood pressure.

7.2.2.2.6 Pregnancy and lactation

The safety of pyronaridine tetraphosphate and artesunate when administered concurrently for use in human pregnancy has not been established, and the potential risk is unknown.

Studies in rats have shown that pyronaridine is excreted into breast milk. The benefits of breastfeeding to mother and infant should be weighed against potential risk from infant exposure to pyronaridine through breast milk.

7.3 METHOD OF TREATMENT ASSIGNMENT

Balanced randomisation will be used using permuted block randomisation methods stratified by site (i.e. hospital or clinic) and by the presence of symptoms at enrolment consistent with COVID-19 (asymptomatic vs symptomatic). A statistician in the UK will generate the randomisation lists with the allocation sequence, which will be forwarded to the trial pharmacists based in Kenya and Burkina Faso who will then prepare sequentially numbered, sealed, opaque boxes or envelopes for each participant with the randomisation assignments. Contained within each of these envelopes or boxes will be the pre-packed investigational product. The opaque boxes/envelopes will be opened sequentially upon enrolment of a study participant in each site. Stratification by the presence of symptoms at enrolment is needed to ensure balanced distribution among the two study arms as the presence of symptoms is likely to be an important (known) predictor of the COVID-19 disease progression (the primary endpoint). Currently at least half of the COVID-19 cases identified in Kenya are asymptomatic, reflecting targeted screening of contacts and high-risk populations. Minimisation or distance randomisation using central randomisation services by internet is not feasible because of the unreliable internet connections and telephone services in some of the proposed sites.

7.4 BLINDING

This is an open-label study using the standard of care as the control arm. A placebo-controlled study is not feasible because of the differences in weight-based dosing categories and dosing frequency between AL (dosed twice daily) and pyronaridine-artesunate (dose once daily) (see Table 1 Dosing

schedule nested treatment study, page 34). Thus, the study pharmacy staff will be unblinded, as they will prepare the study medication, and the study statistician will be unblinded for analysis purposes, as well as the participant and study staff involved with the initial follow-up to assess adherence and tolerance will be unblinded. However, all the laboratory assays, including the viral detection and load assays and immunological assays, including viral shedding (primary endpoint), will be blinded, as laboratory staff will not be informed of randomised assignment. It should be noted that the time seroconversion, viral carriage, and viral load, are objective assessments, unlikely altered by unmasking, should it occur.

7.4.1 Preparation/handling/storage/accountability

Drugs should be stored at room temperature (<30°C) protected from direct sunlight, as per package insert. Records must be maintained that document receipt, release for dosing, disposal, or return to the Sponsor.

7.4.2 Strategies to improve adherence to study protocol monitoring adherence

7.4.2.1 Adherence to study protocol and medication

Where feasible, study participants will be reminded about any follow-up visits through mobile phone contact. All information will be recorded on the appropriate sections of the CRF. Subjects judged to be non-compliant may continue in the study but will be counselled on the importance of taking their study medication as prescribed or following the study's follow-up schedule. Participant initiated consultation via text messaging, or mobile phone calls will be available to provide support to the participant to complete study procedures.

7.4.2.2 Strategies for retention

During screening and consent procedures, potential participants will be asked whether they live in the catchment area, are willing to adhere to the study protocol. Specifically, potential participants who will be eligible to self-isolate at home according to the local Government's guidelines in each country will be asked whether they will be willing and able to comply with the frequent follow-up schedule. The 'study catchment area' will be defined for each study site before the start of the study. Dedicated study transport will be arranged for follow-up visits. All participants who make their own travel arrangements, e.g. for unscheduled/unannounced sick visits, will be reimbursed for transportation costs to and from the clinic.

GPS position and otherwise, detailed directions to participants' homes as well as contact information, including mobile phone information, will be recorded at enrolment. The study team will call participants daily in between scheduled visits. For scheduled follow-up visits study transport will be arranged. If participants cannot be reached by mobile phone, study staff may visit their house to help arrange transport to the clinic if the participants are willing to come to the clinic, or, alternately, a study staff member may go to their home for clinical evaluation and assess if they still wish to participate in the study. All information will be recorded on the appropriate sections of the CRF. Subjects judged to be non-compliant may continue in the study but should be counselled on the importance of taking their study medication as prescribed.

7.4.2.3 Adherence to study drug

If participants are eligible to self-isolate at home, the first dose of each 3-day course will be given under the supervision of study staff and the remaining doses for day-1 (AL), days 2 (AL or PA) and 3 (AL or PA) will be taken at home by the study participants. All participants in this nested malaria treatment trial will be contacted by mobile phone to take the assigned tablets or sachets. If

participants are admitted to isolation wards, the subsequent doses will be given as directly observed therapy by study staff or ward nurses. Details of the time of the dose and adherence monitoring will be recorded on CRFs. If adherence to the prescribed antimalarial treatment regimen is in doubt and/or the correct number of tablets or sachets of the study drug was not taken, an explanation of the reason why the treatment was not followed will be recorded on the case report form where possible.

7.4.3 Permitted and prohibited concomitant medication and care

7.4.3.1 Routine care

Care and management of confirmed COVID-19 patients will be done in line with the COVID-19 National Guidelines which in Kenya[32] and Burkina Faso currently includes isolation for treatment for those testing positive at designated isolation centres approved by the MoH, or self-isolation and home-based care, as well linkage to the contact tracing teams at the MoH offices to facilitate testing of primary contacts. This includes analgesic for fever and other symptoms and haematinics for participants with evidence of anaemia.

7.4.3.2 Concomitant therapy documentation

Participants will be asked about concomitant medications at the screening/baseline evaluation visit. Participants will be counselled to avoid concomitant medications not prescribed by the study clinic, specifically antimalarial drugs. During the study, participants will be asked to complete surveys (Daily Survey and Exit Contact Survey) that include information regarding any medication or vaccine (including over-the-counter or prescription medicines, vitamins, and/or herbal supplements) that the participant receives during the study. At each contact, the Investigator will also question the participant about any medication taken. All concomitant medications taken during the study will be recorded in the appropriate sections of the CRF with indication, dose information, and dates of administration.

All medication used for the management of COVID-19 is permitted, with the exceptions listed under section 7.4.3.3, Prohibited medication, page 38. For COVID-19 participants with malaria who are enrolled in the nested malaria treatment trial, permitted medication includes the use of azithromycin. Other permitted medication includes artesunate or quinine for severe malaria, standard treatments for co-infections (e.g. soil-transmitted helminths, TB, HIV, schistosomiasis, etc.), antibiotics for suspected bacterial infections, haematinics for anaemia, antipyretics/analgesics (e.g. paracetamol, ibuprofen) and treatment for other symptoms as appropriate.

7.4.3.3 Prohibited medication

Prohibited medication for participants enrolled in the treatment study, includes other antimalarial drugs not prescribed within the trial protocol (e.g. chloroquine, amodiaquine), or local remedies that have antimalarial activity, such as herbal remedies containing extracts from the Artemisia annua plant (sweet wormwood) used in a tonic against COVID-19 in Madagascar, Tanzania, Chad, and Togo.[33] Randomised participants who take prohibited medications resulting in the premature cessation of the study intervention, will remain in the trial and will be included in the primary, intention-to-treat analysis, but excluded from the per-protocol analysis.

7.4.3.4 Precautionary medications

Use of medications classified as precautionary is not considered as prohibited but will be discussed with the study clinician and the participant.

7.4.3.4.1 Artemether-lumefantrine

Precautionary medications include drugs that are known to prolong the QT interval. Drugs that have a mixed effect (inhibitors and inducers) on CYP3A4, especially antiretroviral drugs such as HIV protease inhibitors and non-nucleoside reverse transcriptase inhibitors, and those that have an effect on the QT interval should be used with caution in patients taking artemether-lumefantrine. Artemether-lumefantrine may reduce the effectiveness of hormonal contraceptives. Therefore, patients using contraceptives should be advised to use an additional non-hormonal method of birth control.

7.4.3.4.2 Pyronaridine-artesunate

Pyronaridine-artesunate has been associated in some patients with transient increases in liver enzymes without clinical signs. Pyronaridine-artesunate is contraindicated in the case of underlying hepatic injury, clinical signs or symptoms of hepatic injury or known severe liver disease. It is recommended that, in patients who exhibit symptoms of jaundice following treatment with pyronaridine-artesunate, the liver function tests be monitored if possible, until normalisation. The safety and effectiveness of pyronaridine-artesunate for the treatment of malaria in patients with HIV/AIDS has been studied in fifteen patients, with no apparent differences in efficacy or safety compared with HIV negative patients (Shin Poong and Stephane Duparc, MMV, personal communications, unpublished observations).

7.4.4 Treatment after the end of the study

No additional treatment will be provided at the end of the study.

8 DISCONTINUATION/WITHDRAWAL CRITERIA

8.1.1 Discontinuation of study treatment

Study treatment for those enrolled in the nested malaria treatment trial will be discontinued for the following reasons:

- Hospitalisation (at the discretion of the inpatient provider, hospitalised participants may continue to receive study treatment if maintained on the originally randomised treatment regimen)
- The requirement for prohibited concomitant medications or other contraindication to study product
- The occurrence of an AE requiring discontinuation of study product, even if not addressed in Section 7.2, page 34
- Request by a participant to terminate study treatment

Participants who stop study product should continue the study participation off study product with continued evaluations as per the Schedule of Activities. The reason for study product discontinuation should be recorded.

Hospitalised participants will be followed through hospitalisation.

8.1.2 Withdrawal from the study

- A participant may withdraw from the study at any time at his/her own request or may be withdrawn at any time for the following reasons:

- At the request of the primary care provider if he/she thinks the study is no longer in the best interest of the participant
- A participant is judged by the Investigator to be at significant risk of failing to comply with the provisions of the protocol as to cause harm to self or seriously interfere with the validity of the study results
- At the discretion of the Institutional Review Board/Ethics Committee or government agencies as part of their duties, Investigator, or industry supporter

- If the participant withdraws consent for disclosure of future information, the Sponsor may retain and continue to use any data collected before such withdrawal of consent.
- If a participant withdraws from the study, he/she may request destruction of any samples taken and not tested, and the Investigator must document this in the site study records. If the request is received after the dataset has been anonymised, the stored sample can no longer be withdrawn.
- See for data to be collected at the time of study discontinuation and follow-up and for any further evaluations that need to be completed.

8.1.3 Lost to Follow-up

A participant will be considered lost to follow-up if he/she is unable to be contacted by the study site while taking the following actions into account:

- The site must attempt to contact the participant as soon as possible and counsel the participant on the importance of maintaining the assigned procedure schedule and ascertain whether or not the participant wishes to and/or should continue in the study.
- Before a participant is deemed lost to follow-up, the Investigator or designee must make every effort to regain contact with the participant (where possible, three telephone calls and, if necessary, a home visit to the participant's last known place of residence). These contact attempts should be documented in the participant's case record forms.
- Should the participant continue to be unreachable, he/she will be considered to have withdrawn from the study with a primary reason recorded as 'lost to follow-up'.

9 Participants Timeline and Study Encounters

The current COVID-19 pandemic has placed a significant burden on the healthcare system. For this study, specimen and data collection will be conducted to minimise the impact of asymptomatic or paucisymptomatic participants within the healthcare system. If the participant is assessed as eligible, direct contact between study participants and study personnel will be minimised, and we will make use of mobile phone and text message as much as possible during the follow-up period on days that biological sampling is not indicated.

Participants will be instructed to contact the study staff by phone as soon as possible should they manifest signs or symptoms of LRTI or if their clinical situation worsens. They will also be instructed to notify any non-study health care personnel about trial participation if they seek care from non-study clinics.

9.1 Screening Study (Cross-Sectional Survey Source Population)

Most of the source population data and procedures will be collected and conducted by routine staff as part of the local response to the COVID-19 epidemic. Basic demographic, medical and travel histories will be taken, the approximate location of residence determined, and a basic physical

examination performed. Any relevant demographic or health-related information will be copied from existing hospital and clinic records. The contact telephone numbers and location of residence will be recorded for all participants.

As of writing (July 2020), most suspected cases, including those with mild or no symptoms, are admitted for at least one day on the quarantine/isolation wards of the local referral hospitals until the results of the COVID-19 test are known. It is expected that this may change during the course of the pandemic (and thus the study) when hospital isolation wards reach capacity when it is anticipated that Government guidelines in each country will allow or encourage self-quarantine or self-isolation at home and home-based care while waiting for the COVID-19 test results, which are typically available within 18 to 48 hours, although this may vary during the course of the pandemic.

9.1.1 Pre-screening with point of contact (POC) SARS-COV-2 antigen tests

The screening study (cross-sectional survey, source population) includes pre-screening with an experimental POC SARS-COV-2 antigen tests and Government approved antigen tests. At the time of writing (September 2020), there are no approved SARS-CoV-2 antigen tests yet in Kenya or Burkina, but this may change during the course of the study. We will follow the national guidelines in each country. In Kenya GeneXpert, antigen-RDT and PCR results are considered for definitive diagnosis of COVID-19. If a participant tests COVID positive for any rapid antigen tests, GeneXpert, or PCR, the participant shall be enrolled.

Eligible patients with both a positive SARS-COV-2 test and a positive malaria test on Day-0 require malaria treatment that same day and will be invited to enrol in the COVID-19 cohort study and the nested malaria treatment trial on Day-0; i.e. prior to knowing the SARS-COV-2 PCR results (see 9.2.1.3, Nested malaria treatment trial, page 43). Patients with a positive antigen test but negative for malaria will be invited to participate in the COVID-19 cohort study and can choose whether to enrol that same day (Day-0) or wait until the confirmatory PCR results become available (typically on Day-1).

9.1.2 Healthy controls

In addition to enrolling those with COVID19 into the cohort, approximately 100 healthy control participants (~ 50 per country) will be invited to provide a venous blood sample (5 mL) at a single timepoint. These will be individuals identified through the screening study (cross-sectional survey, source population) and will be SARS-CoV-2 antigen RDT negative (nasal swab) and Plasmodium antigen-negative (finger prick) and will have no symptoms at the time. Where such individuals have been identified retrospectively within the previously screened population, they will be contacted and invited to repeat the rapid tests to confirm their COVID-19 and malaria-free status before providing a venous blood sample. If they have received a COVID-19 vaccine, this should be at least four weeks before the samples are taken.

Venous blood (5 mL) from those who consent will be taken into an EDTA tube and a PAXgene tube to correspond with samples from COVID-19 cohort participants.

Venous blood from healthy control participants will be used for the systems immunology component investigating T and B cell markers and for transcriptional profiling as a comparison/control group against the COVID-19-malaria negative and the COVID-19-malaria positive patients.

9.2 COVID-19 COHORT STUDY AND NESTED MALARIA TREATMENT TRIAL

9.2.1 Pre-Screening (Day 0/1)

Newly diagnosed COVID-19 cases will be eligible for enrolment in the COVID-19 cohort study regardless of malaria status. They will be pre-screened for study eligibility, based on a selection of the inclusion criteria that can be determined without informed consent

9.2.1.1 Pre-screening criteria

1. Confirmed SARS-CoV-2 infection by PCR or antigen test, with positive test results within the past 72 hours
2. Resident in the study area
3. Aged >=6 months

If they fulfil the pre-screening criteria, they will be approached for prior consent discussions to determine if they would potentially be willing and able to attend informed consent procedures.

9.2.1.2 Screening and enrolment

If they are potentially interested in joining the study and fulfilling all other inclusion and exclusion criteria the informed consent procedures will be conducted by dedicated staff trained to GCP or equivalent level (see also 2.2.4, Schedule of activities, page 19), the COVID-19 cohort study code issued and the following baseline information collected

- Further demographic information
- Socioeconomic and educational history
- Details of the location of residence
- Tel contact number(s)
- Past and current medical conditions
- Concomitant medication information
- COVID-19 exposure history, including any known exposure to any other, confirmed or suspected COVID-19 cases
- Travel history

A basic physical examination will be performed. Any relevant demographic or health-related information will be copied from existing hospital and clinic records. A venous whole blood sample will be taken (15mL) for further malaria rapid diagnostic test for patient care, and the remaining sample transported within two hours to the laboratories at KEMRI-CGHR in Kisumu and the laboratories of the Groupe de Recherche Action en Santé(GRAS) in Burkina Faso for further processing and storage of whole blood, pellets and serum/plasma (for details, see 2.2.4, Schedule of activities, page 19 and section 10.2, Biological samples, page 45). Some samples will be stored for further distribution to the testing laboratories. In addition, a confirmatory nasal swab will be taken for research purposes and later testing (not for patient care).

Participants will receive a monitoring kit, which will include a digital thermometer and a portable SpO2 pulsometer. They will be instructed on how to self-assess their body temperature, oxygen saturation and respiratory rate in between the scheduled visits (see 9.2.2, Scheduled follow-up visits day 3, 7, 14, 21, 28 and 42, below) and record results daily on a dedicated form. Participants will be instructed that they will be contacted daily by study staff by mobile phone to check on their health status through a brief telephone survey. Participants who don't have a working mobile phone will be issued with a temporary mobile phone from the study, which needs to be returned after study

closeout. Participants will also be instructed how to contact the study team if their symptoms worsen or if they need to contact the research team for other reasons.

9.2.1.3 Nested malaria treatment trial

Participants with a positive rapid diagnostic test (HRP2 or pLDH band) or positive microscopy for malaria requiring antimalarial treatment will be potentially eligible for enrolment in the nested malaria treatment trial if they provide informed consent and have mild disease and are able to take oral medication (i.e. have uncomplicated malaria). Patients with RDTs that are positive by HRP2 band only will only be followed if subsequent microscopy confirms the presence of asexual parasitaemia.

Participants will be counselled about the preliminary in vitro data on the activity of the planned treatment arms against malaria and SARS-CoV-2 and equipoise regarding efficacy in humans.

They will then be randomised and receive a box or envelop with their study medication. The first dose on day-0 will be provided as directly observed therapy in the clinic.

Participants will be given some clean water for swallowing the tablets. Participants will then be observed for thirty minutes. In the event of vomiting within thirty minutes of administration after the first dose, a repeat dose should be given. If the repeat dose is vomited, the patient should be given the alternative antimalarial drug from the other arm.

If a dose is missed, it should be taken as soon as realised, and then the recommended regimen continued until the full course of treatment has been completed.

The MOH takes malaria RDTs or malaria microscopy at the same time as the COVID-19 swabbing in symptomatic patients only. Asymptomatic patients are not routinely tested for malaria, and this will, therefore, be conducted as part of the screening study on day 0 or if that was not feasible, later when the results of the COVID-19 test become available and patient return for their results on Day-1. Because the malaria test results are typically known within 20 minutes to 2 hours, treatment will need to be initiated that same day. Eligible patients with both a positive SARS-COV-2 antigen test and a positive malaria test on Day-0 will therefore be invited to enrol in the COVID-19 cohort study and the nested malaria treatment trial that same day (Day-0).

9.2.2 Scheduled follow-up visits day 3, 7, 14, 21, 28 and 42

We will follow local MoH guidelines in each country to determine if a participant requires hospitalisation and for how long, or if a participant will be invited to self-isolate at home.

Participants who are self-isolating will be asked to return to the study clinics for scheduled follow-up visits on days 3, 7, 14, 21, 28 and 42. Of these visits, the follow-up visits on day 21 and 42 apply to the participants enrolled in the nested malaria treatment trial only. During each of these scheduled follow-up visits, a similar symptom questionnaire will be filled out, concomitant medication will be reviewed, and brief physical examination performed, and the respiratory rate and pulse, body temperature and O2 saturation taken by study staff. The participant will be asked to bring the form with the self-assessed values for body temperature and O2 saturation values that were taken by the participant at home for verification of the data and the technique used by the participant. A photo will be taken of the self-assessment form for subsequent data entry and verification. Where relevant, any relevant demographic or health-related information will again be copied from existing hospital and clinic records. Similar venous blood samples (ml), swab samples will be taken as on enrolment.

Participants who will be admitted on isolation wards will be monitored daily for signs and symptoms of severe COVID-19, and have daily respiratory rate, body temperature and O2 saturation measured. Similar blood and swab samples will be taken for participants who are self-isolating.

If participants are enrolled in the nested malaria treatment trial will have an additional 5mL of whole blood taken for antimalarial drug levels from the same blood draw as the overall sample (i.e. the total blood volume per blood draw on days 3, 7, 14, 21, 28 and 42 will be 20mL (15mL for the above-mentioned assays under 'k' in the section 2.2.4, Schedule of activities page 19 and 5mL for pharmacokinetics). The volume samples in children below the age of assent will be less and based on bodyweight (total 2.5 mL <10kg and 5 mL 10-<15kg per sample) (see section 12.7.1.4, Maximum blood volume, page 63) and will be excluded from the pharmacokinetic sampling.

The data collected on these days will also be used for the assessment of the antimalarial treatment response as described in section 10.3.2, Assessment of the antimalarial treatment response, page 47.

9.2.2.1 Home visits, clinic visits and transport arrangements

If patients are asked to self-isolate at home, the scheduled follow-up visits during the acute (infectious) phase can take place either in the clinic or at home (home visits are currently standard practice for follow-up of COVID-19 patients in Burkina Faso). The choice will depend on local guidelines in each country and feasibility of transport arrangements and the clinical status of the patient. The overall aim is to minimise the risk of further transmission of COV-SARS-2 and the burden to sick patients self-isolating at home. If patients are requested and able to return to the clinic, special transport arrangement by dedicated vehicles and trained drivers and study staff will be made during the acute infectious phase. In all these circumstances (clinic visits, home visits, transport for staff or participants), strict adherence to personal protection equipment (PPE) and other infection prevention and control (IPC) measures will be followed according to local guidelines in each country to minimize the risk of transmission of SARS-CoV-2 (see also 12.7.3.1, Biohazard containment to limit risks of SARS-CoV-2 transmission, page 65).

9.2.3 Telephone contact Days 2, 4-6, 8-13, 15-27

Participants who are self-isolating, or have been discharged, will be contacted via mobile phone on the days in between the scheduled visits. Participants will be clinically assessed for signs and symptoms of respiratory distress and will have an in-person assessment of AEs. Support will also be provided with the self-assessment and recording of the daily body temperature, oxygen saturation, pulse and heart rate. As needed, additional contact with the study clinician or staff will be conducted at the request of the participant (e.g., if developing concerning symptoms or an adverse event) or if needed to clarify study procedures or follow-up symptoms.

9.2.4 Close-out visit day 28 Cohort

In addition to the measurements taken at the other scheduled follow-up visits, a close-out questionnaire will be filled out on day 28 with a brief summary whether the participant completed the study, was lost to follow-up, was (re-)admitted /discharged from/to hospital and when, and survival status.

9.2.5 Close-out visit day 42 nested malaria treatment trial

In additional malaria-specific close-out visit will take place on day 42 in the patients enrolled in the nested malaria treatment trial to assess the therapeutic response to the antimalarials. The rationale for this additional day-42 visit is based on the standard 6-week duration of follow-up recommended by WHO for treatment efficacy studies of antimalarials.

9.2.6 Extended follow-up visits at month 3, 6, and 12

Funding permitting, participants will be asked to return to the clinic for extended follow-up visits 3, 6, and 12 months after enrolment to assess the longevity of the antibody responses to SARS-CoV-2. A sample for malaria RDT, microscopy and PCR will be taken to continue to determine any associations between malaria co-infection and persistence of SARS-COV-2 immune responses.

9.2.7 Participants who develop malaria during the 42 days follow-up period

Participants enrolled in the nested malaria treatment trial who have recurrent malaria detected by microscopy on days 7 to 42 inclusive will be re-treated with the antimalarial from the other arm. Participants without malaria at enrolment who develop clinical malaria during follow-up will be treated with artemether-lumefantrine.

10 STUDY ASSESSMENT AND PROCEDURES

10.1 SCHEDULE OF ACTIVITIES AND SAMPLES

See section 2.2.4, Schedule of activities, page 19 for an overview of all activities and biological samples.

10.2 BIOLOGICAL SAMPLES

10.2.1 Mid-nasal swab

We will take two mid-nasal swab sample for research specific molecular detection of SARS-CoV-2 (for later batch analysis, not for patient care). Mid-nasal cavity are less intrusive then nasopharyngeal swabs with less potential for the patient to sneeze, cough, or gag and provide a more comfortable patient experience. Mid-nasal swabs were shown to have a sensitivity of 94% compared to nasopharyngeal swabs.[34]

The nasal swabs will be placed in the plastic tube with transport media, which will be placed into a labelled specimen bag, pre-packaged with an absorbent sheet. One swab will be placed in viral transport media (e.g. DNA/RNA Shield), the other in 0.9% saline, or phosphate-buffered saline, which have been shown to be equivalent for storage of SARS-CoV-2 samples based on validated assays. Previous testing has demonstrated that respiratory viral ribonucleic acid (RNA) is stable in room temperature for up to 1 week. Samples will be stored at -80°C prior to use.

Samples eluted from swabs will be subjected to RNA amplification and tested for SARS-CoV-2 and used for viral invasion assays.

Viral RNA loads will be estimated using quantitative real-time reverse-transcriptase polymerase chain reaction (qRT-PCR) assays which target multiple sites in the SARS-CoV-2 genome, including at least one position each in the Orf1ab and N regions. Validated and standardised quantified plasmid and in vitro transcript RNA controls will be made available to all partner laboratories for use in viral RNA load estimation.

10.2.2 Blood samples

During the screening study (cross-sectional survey, source population) a fingerprick blood sample (about 250 uL) will be taken for malaria diagnosis by malaria RDT, malaria microscopy and a dried blood spot for malaria PCR, and for SARS-CoV-2, malaria and non-malaria serology. Approximately 100 healthy control participants (~ 50 per country) who test negative by SARS-CoV-2 antigen RDT during screening and are malaria RDT negative will have a 5 mL venous blood sample taken.

In the participants enrolled in the COVID-19 cohort study, whole blood will be stored in PAXgene Blood RNA Tubes, which inactivates and stabilises the virus, for human transcriptomics changes related to SARS-CoV-2 related and as dried blood spots (DBS, which also inactivates the virus) for Mass cytometry (CyTOF) for B and T cell markers, and parasite and host genetics related to susceptibility to respiratory illness and COVID-19 infection. The serum will be used for SARS-CoV-2, related coronaviruses and malaria serology (IgG, IgM and IgA), and cytokine and chemokine panel assays. Full blood haematology and biochemistry will be performed on day 0 and day 28. Human transcriptomics, CyTOF and cytokine and chemokine assays will be carried out on the blood samples from healthy control participants. For more details about the laboratory assays, please see sections 16.9, Appendix IX. Description of other clinical and laboratory methods, page 118.

Should funds be available, the serological assays will be extended to include antibody responses to other potential coinfections, including hepatitis, respiratory pathogens such as respiratory syncytial virus, parainfluenza, *Klebsiella pneumoniae* and parasitic infections such as schistosomiasis, and soil-transmitted helminths. Additionally, whole blood will be run for molecular diagnoses on a TaqMan array card panel[35] to evaluate for potential viral, bacterial and parasitic coinfections and for tuberculosis Quantiferon assay (see 10.2.4, HIV testing and TB testing, below).

10.2.3 Other biological samples

Saliva samples will be taken for the measurement of antibodies (all cohort patients), viral RNA/antigen, and pyronaridine drug concentrations (among those enrolled in the pyronaridine-artesunate arm, and in addition to the assessment of pyronaridine concentrations in blood described in section 10.2.2, Blood samples, above) (see also 2.2.4, Schedule of activities, page 19).

Stool samples will be requested from cohort participants on day 0 or 1 and days 3, 7, 14 and 28 and stored in saline at -20degrees. Stools will undergo molecular testing for soil-transmitted helminths and viral RNA/DNA and detection of SARS-CoV-2.

It will not be considered a protocol deviation if saliva or stool samples are not collected from all participants at all time points.

10.2.4 HIV testing and TB testing

HIV testing will not be done as part of this research study, but any relevant information (i.e. known HIV status) will be copied from existing health records and any anti-viral medication noted in the case report forms. We will work in close liaison with existing HIV counselling and testing services to refer any participants for counselling and testing.

The blood-based Quantiferon assay will be used to screen for tuberculosis. Individuals who are Quantiferon positive will be referred for further testing to differentiate between active disease and latent infection as per national guidelines in each country. They will be run in batches, and the anticipated turnaround time for a Quantiferon test result coming from the field is approximately seven days.

10.3 Assessment of clinical and epidemiological endpoints

10.3.1 Assessment of disease progression

Disease severity, will be compared between the groups daily over the first 14 days and then at days 21 and 28. We will use the updated WHO Clinical Progression Scale (see 16.4, Appendix IV. WHO clinical progression scale, page 82).

Alternately, we will consider the inFLUenza Patient-Reported Outcome instrument (Flu PRO) PLUS – Modified for SARS-CoV-2. FLU-PRO is a well-established instrument for the collection of respiratory symptoms (see 16.5, Appendix V. Modified Flu-PRO PLUS scale, page 83).[36, 37, 38, 39]

10.3.2 Assessment of the antimalarial treatment response

The parasitological and clinical antimalarial treatment response will be assessed using standard methods for therapeutic efficacy studies (TES) and the data collected on days 0, 3, 7, 14, 21, 28 and 42.[40] Recrudescence will be differentiated from new infection by genotyping of malaria parasites by day 42.[41]

10.4 Assessment of immunological endpoints

10.4.1 Cytokine, chemokine, and inflammatory marker profile

Serum samples collected at days 0, 1, 3, 7, 14 and 28 will be tested for cytokine and chemokines associated with cytokine storm phenomena (interleukin (IL)-2, IL-7, granulocyte colony-stimulating factor, interferon-γ inducible protein 10, monocyte chemoattractant protein 1, macrophage inflammatory protein 1-α, and tumour necrosis factor-α) and inflammatory markers (CRP, angiotensin 1 & 2 procalcitonin, chitinase, MxA, TREM-1). Other markers of disease severity such as D-dimer, ferritin, lactate dehydrogenase will also be considered.

10.4.2 Antibody Testing

Serum samples for serology collected at days 0, 1, 3, 7, 14 and 28, 42, and at 3, 6, and 12 months will be tested for IgA, IgM, and IgG antibodies to SARS-CoV-2 and malaria-specific antigens and other infections. Avidity of SARS-CoV-2 antibodies will also be measured, and a sub-set of serum samples will be used for neutralising assays in order to relate antibody levels to viral neutralising activity.

10.4.3 transcriptional profiling and cellular activation

Whole blood collected in EDTA will be mixed with a stabiliser (e.g. PROT1 Proteomic Stabilizer, Fisher #501351691), vortexed and frozen -80C. Serum samples will be stored at -80 for later proteomic screening for transcriptional biomarkers.

10.5 Genetic testing

10.5.1 Sample for host genetic testing

Genetic markers may be plausibly associated with SARS-CoV-2 acquisition and severity. Recent studies indicate that angiotensin-converting enzyme 2 (ACE2) and transmembrane serine protease 2 (TRPMSS2) are the host receptor for SARS-CoV-2. Variants in these receptors could reduce the association between ACE2 or TRPMSS2 and SARS-CoV-2. Therefore, the expression of human ACE2 and TRPMSS2 might be important for the susceptibility, symptoms, and outcome of SARS-CoV-2 infection.[42] Other variants such as HbS, HbC, ABO blood group and immune-related genes, including those for chemokine receptors and monocyte and T-cell markers have also been implicated. The association between plausible genetic markers and SARS-CoV-2 infection and disease severity will be assessed in this study. Since genetic investigations underlying COVID-19 is an active area of research and any new genetic variants affecting susceptibility or severity of disease will also be included in this study. Whole blood pellet and dried blood spot samples collected in the study are viable for genetic studies. During the consent process, participants can opt-out of genetic testing.

10.5.2 Host genetic information that will be obtained and genetic testing procedures

Anonymised stored samples will be shipped to laboratories in the United Kingdom to investigate genes that may play a role in susceptibility to SARS-CoV-2 infection or other respiratory viruses

and/or severity of COVID-19 and malaria. The samples will not be used to study genes related to diseases other than SARS-CoV-2 or other respiratory viruses and malaria. Samples will be analysed for the presence of single or combinations of genetic markers, which the investigators believe, on the basis of available medical literature, to possibly be associated with susceptibility to SARS-CoV-2 infection and/or the severity of COVID-19 or malaria. Genetic variants will be analysed as single or haplotypic responses for associations with clinical and immunological outcomes.

Nested multiplex PCR will be developed for all SNP's of interest and a bead-based detection assay for the resulting oligonucleotides will be used to assess their prevalence. In addition, we will use exome sequencing, also known as whole-exome sequencing (WES) to investigate the presence of rare disease variants associated with SARS-CoV-2 severity, including in a sub-sample of the most severe patients. Genome-wide screening is not planned. The assays will be conducted in the United Kingdom to ensure comparability between both countries. These tests will be run centrally in batches on anonymized samples, and the test results cannot be reported back to participants. For more details, see 16.9.6, Host genetics, page 122.

10.6 Pharmacokinetics

The exposure-response relationship of pyronaridine for SARS-CoV-2 has not been established. Population PK analyses can be used to further inform dose selection in other populations and support concentration-response investigations with efficacy and safety endpoints.

To accomplish this, sparse PK sampling techniques with the collection of whole blood and plasma at days 3, 7, 14, 21, 28 and 42.

The basic requirements for PK sampling are as follows:

- An accurate record of the time of the dose prior to the blood sampling (dd:mm:yy; hh:mm)
- Timing of the Tmax sample will take place approximately 3-4 hours after drug intake of the third dose based on the history of drug intake by the patient.
- Accurate recording of the time of blood sampling (dd:mm:yy; hh:mm) for each blood sampling
- Whole blood will be obtained by venipuncture
- Whole blood is collected into two tubes, one preserved as whole blood and the other to obtain plasma using centrifugation. Both plasma and whole blood are required to explore plasma vs blood partitioning which is important for drugs like pyronaridine with high blood to plasma ratio (>10 fold)
- Samples are later analysed using Liquid chromatography-mass spectrometry (LC/MS-MS).

10.7 Sample storage and shipping

10.7.1 Storage

Swab specimens can be stored at 2-8°C for up to 72 hours after collection. If a delay in testing or shipping is anticipated, specimens will be stored at ≤ -70°C.

10.7.2 Shipping

All specimens will be packaged, shipped, and transported according to the current edition of the International Air Transport Association (IATA) Dangerous Goods Regulations as at the time of shipping.

At the time of writing whilst the WHO advises the transport of specimens to laboratories for analysis is generally working well, there have been some delays caused by confusion over the classification of specimens in transit, and whether this falls within UN 3373, Biological substance (i.e. Category B), or as

UN 2814 (i.e. Category A). Reflecting on international experience shipping specimens of SARS-CoV-1, Influenza A/H1N1/2009 and MERS-CoV international bodies (including WHO and US CDC) are advising to ship specimens as UN 3373, Biological substance, Category B. As such, all specimens will be transported in accordance with UN 3373 and packed in accordance with PI 650.

10.8 Data collection methods & storage

10.8.1 Methodologies for data collection / generation

Data will be captured using a combination of paper-based and electronic record forms. For the questionnaires that are administered in paper format, the study will utilise ScanForm (QED®) software to design the paper-based CRFs for semi-automated transcribing into an electronic database by taking images with android-based ScanForm App and Optical Character Recognition, intelligent document recognition and data validation and human verification of information against source documents. Once validated, the data will be transferred to the target database along with a PDF of the original image of the CRFs, such that there is an electronic copy of all paper-based documents. For the electronic-only data capture, we will use tablets with integrated sim cards to transfer encrypted data to the ODK servers. This has worked well in previous studies.

10.8.2 Data quality and standards

The quality of questionnaire data collection and data entry will be maximised through training of field staff in the standardised questionnaire administration methodology. Field staff will be trained in the methodology for collecting data and will be expected to demonstrate competence before conducting fieldwork. All electronic CRFs and data validations processes for data captured through Teleform will incorporate range and consistency checks.

10.8.3 Managing, storing and curating data

Verified and validated data from both countries will be stored on a secure, highly fault-tolerant, storage area network servers. Locally, data will be backed-up on a continuous basis on a secure off-site server and on encrypted standalone hard drives.

Once the data validation phase is completed by the central data manager, the database will be locked and transferred to a statistical programmer who will do further syntax-driven consistency checks and syntax-driven data cleaning. The statistical programmer will have access to the PDF copies of the source data. He/she will then prepare the database for data analysis by the statistician by creating the final variables for data analysis, such as the creation of the composite endpoints. The final cleaned database will be available in Stata and other formats, with a corresponding data dictionary.

10.8.4 Metadata standards and data documentation

The full study protocol, supporting documents including the data management Standard Operating Procedures (SOPs) and the full individual participant level database will be made publicly available once the study findings have been published. The data manager and statistical programmer will produce a document summarising the methods used to generate the data with a full description of all procedures, analyses, data capture tools, coding and description of variables.

10.8.5 Data preservation strategy and standards

The majority of the data collected will be captured using scannable paper-based forms. The scanned PDF copies will be used for archiving, and the original paper questionnaires kept in a dedicated lockable data storage room in-country and then destroyed after a minimum period subject to the prevailing laws in each country. The country-specific paper-based CRFs, the scanned PDF copies of

the paper-based CRFs, and the databases will be stored and archived at KEMRI's Centre for Global Health Research (CHGR) in western Kenya (for the data collected in Kenya) and in the for the Groupe de Recherche Action en Santé (GRAS) Institute in Ouagadougou, Burkina Faso (for the data collected in Burkina Faso). The research data will be stored in the long-term in the original electronic format, in a large unified database and a public database that contains all research data other than identifiable participant data. The public database will be updated when needed if the software becomes obsolescent to achieve long-term preservation. The data will be preserved in this way for ten years or longer if still being accessed at that stage.

10.9 Data monitoring and trial oversight committees

10.9.1 Data Safety and Monitoring Board (DSMB)

Since the study contains a nested clinical trial, an independent Data Safety and Monitoring Board (DSMB) will be set up. The DSMB will be critical to ensure that the subjects are protected from harm, while also ensuring that the study integrity is not compromised. The DSMB will consist of 3 to 4 independent topic experts. They will meet regularly (e.g. twice yearly or more frequently if so required) during data collection period to provide a review of blinded (and if requested unblinded) data to ensure the safety, rights and well-being of trial participants. In addition, regular review of the quality of the study data will be conducted at each meeting of the DSMB (for more details, see section 16.2.2, Data Safety and Monitoring Board (DSMB), page 77).

10.10 Safety monitoring and reporting

The principles of ICH GCP require that both investigators and sponsors follow specific procedures when notifying and reporting adverse events or reactions in clinical trials. Participants will be asked to complete daily surveys on CRFS and/or by telephone interview that include information on any symptoms that they are experiencing. In addition, AE review by a staff member (via telephone or text messaging) will be performed.

10.10.1 Definitions

The following definitions apply to this protocol:

10.10.1.1 Adverse Event (AE)

Any untoward medical occurrence in a patient or clinical trial subject administered a medicinal product and which does not necessarily have a causal relationship with this treatment.

10.10.1.2 Adverse Reaction (AR)

Any untoward and unintended response to an investigational medicinal product related to any dose administered.

Comment: All adverse events judged by either the reporting investigator or the sponsor as having a reasonable causal relationship to a medicinal product would qualify as adverse reactions. The expression 'reasonable causal relationship' means to convey, in general, that there is evidence or argument to suggest a causal relationship.

10.10.1.3 Serious Adverse Event (SAE) or Serious Adverse Reaction (SAR)

Any adverse event or adverse reaction that results in death, is life-threatening*, requires hospitalisation or prolongation of existing hospitalisation, results in persistent or significant disability or incapacity, or is a congenital anomaly or birth defect.

Comment: Medical judgement should be exercised in deciding whether an adverse event/reaction should be classified as serious in other situations. Important adverse events/reactions that are not immediately life-threatening or do not result in death or hospitalisation, but may jeopardise the subject or may require intervention to prevent one of the other endpoints listed in the definition above, should also be considered serious.

*Life-threatening in the definition of a serious adverse event or serious adverse reaction refers to an event in which the subject was at risk of death at the time of the event. It does not refer to an event which hypothetically might have caused death if it were more severe.

10.10.1.4 Suspected Unexpected Serious Adverse Reaction (SUSAR)

An adverse reaction that is both unexpected (not consistent with the applicable product information) and also meets the definition of a Serious Adverse Event/Reaction.

10.10.1.5 Intensity

The intensity of each AE recorded in the case report form should be assigned to a grade (1-5), which will be determined following the definitions set forth in the Common Terminology Criteria for Adverse Events v5.0 (CTCAE) (Cancer Therapy Evaluation Program, 2006). Use of these standardized guidelines will allow for uniform reporting. The grades are defined as follows:

- Grade 1: Mild AE
- Grade 2: Moderate AE
- Grade 3: Severe AE
- Grade 4: Life-threatening or disabling AE
- Grade 5: Death related to AE

10.10.2 Identifying, managing adverse events

Participants who develop adverse events as a consequence of the study interventions or other treatments will be identified at follow-up visits and referred to the designated hospital for evaluation and treatment according to local guidelines in each country. Mild adverse events will be noted in the participant's case report form; no further action will be taken by study staff except in the case of vomiting, in which case the study medication may need to be re-administered. In the case of any SAE, subjects will be referred to the hospital for management. Transportation to the hospital will be provided where feasible or transport reimbursed. All participants with SAEs will undergo a record review to identify potential adverse consequences of study participation.

10.10.3 Assessment of causality

The investigator is obligated to assess the relationship between the investigational product and the occurrence of each AE/SAE. The investigator will use clinical judgment to determine the relationship, which will be scored using four categories (related, possibly related, probably related, not related). Alternative causes, such as the natural history of the underlying diseases, concomitant therapy, other risk factors, and the temporal relationship of the event to the investigational product will be considered and investigated. The investigator will also consult the drug information and the DSMB as needed in the determination of his/her assessment.

There may be situations when an SAE has occurred, and the investigator has minimal information to include in the initial report. However, it is very important that the investigator always assess causality for every event prior to transmission of the SAE report. The investigator may change his/her opinion of causality in light of follow-up information, amending the SAE case report form accordingly.

10.10.4 Reporting adverse event procedures

All SAEs and any pregnancies will be reported to the in principal country investigator or an assigned representative within 24 hours of the staff becoming aware of it, using an SAE form, which should be completed and sent electronically. The SAE form asks for nature of the event, date of onset, severity, any corrective therapies given, outcome and causality (i.e. unrelated, unlikely, possible, probably, definitely). The responsible study clinician should assign the causality of the event.

10.10.4.1 Expedited reporting

SAEs that are unexpected and are at least ‘possibly related’ to the study drug require expedited reporting within 24 hours of the country principal investigator or assigned representative becoming aware of it (e-mail notification); i.e. this will be a maximum of 48 hours after the event occurred or the study team were made aware of the event (including the 24 hours required for the field staff to report to the principal investigator/representative). Additional information will be sent within 14 additional days (full SAE report) if the reaction had not resolved at the time of e-mail notification.

10.10.4.2 Scheduled reporting

Other SAEs and AEs will be reported annually (or more frequently if required by the DSMB or ethics committees) in an aggregated report. AEs that will not be reported include common illnesses that do not result in hospitalization, including but not limited to clinical malaria, respiratory, gastrointestinal, and skin diseases unless they are considered at least possibly related to the intervention.

10.10.4.3 Recipients of reports

The study will comply with local regulations pertaining to reporting of SAEs to their local Research Ethics Committee and/or Research & regulatory offices. In addition to the primary ethics committees, we will report safety data to the DSMB, and to the sponsor. In addition, and if requested by the manufacturer, the sponsor will forward the reports to the manufacturer of the investigational products. A copy of the final study report will be provided to all study hospitals, ethics committees, DSMB, to local regulators, and, if so requested, to the manufacturers of artemether-lumefantrine and pyronaridine-artesunate.

Figure 4: Safety reporting assessment flowchart[43]

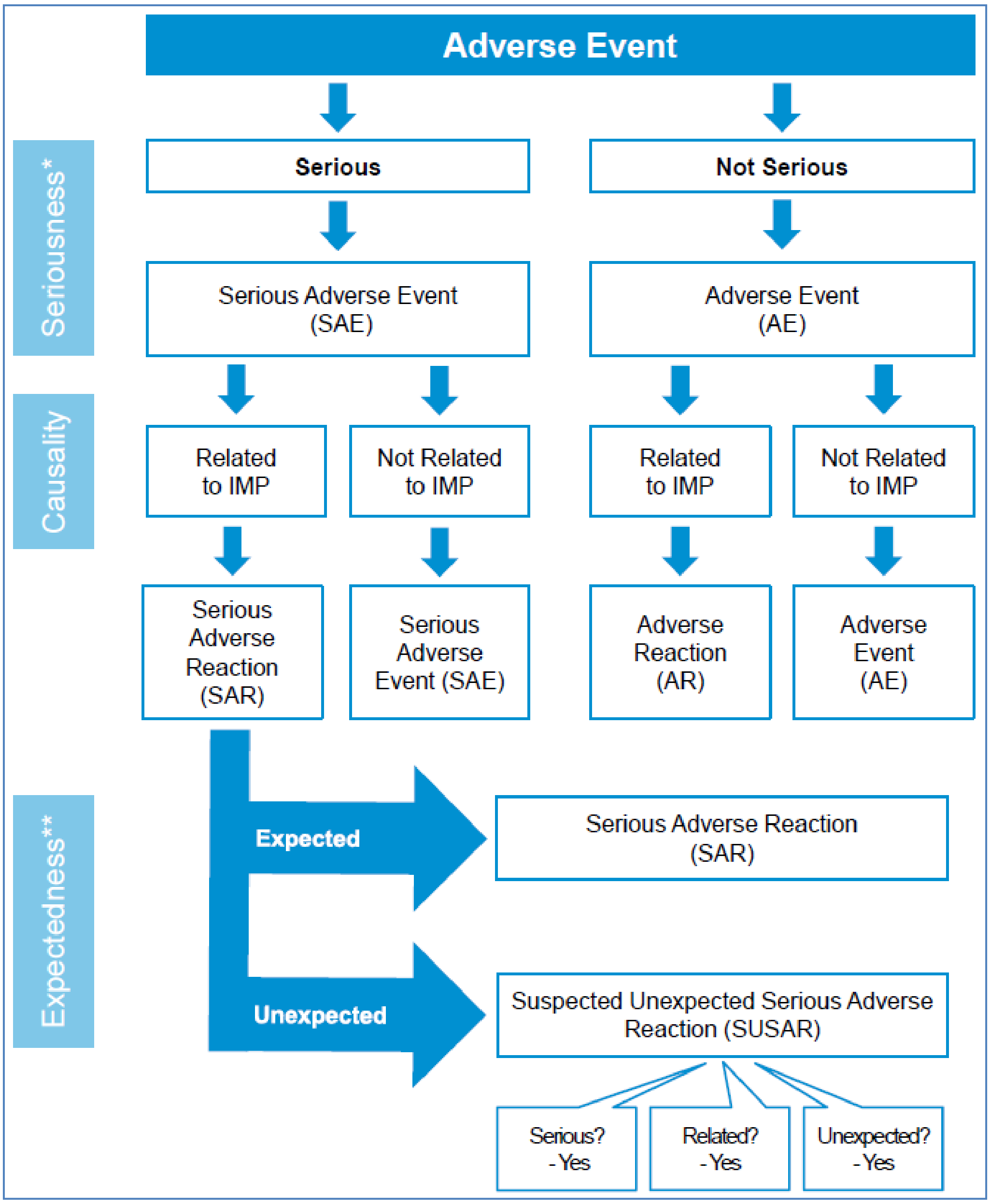

Adapted from ct.toolkit.ac.uk. IMP: Investigational Medicinal Product
*See definition of SAE in section 10.10.1
**Assessed in line with the summary of product characteristics (SmPc) (see section 16.7, Appendix VII. Product characteristics, page 86)

10.11 BIOHAZARD CONTAINMENT

See section 12.7.3, Risk to the population and study staff and biohazard containment, page 65 about details of procedures to reduce the risk of infection with SARS-CoV-2 to study staff.

10.12 TRIAL MONITORING AND AUDITING

10.12.1 Trial monitoring

Study conduct will be monitored by independent monitors. External clinical trial monitoring visits are provided by the sponsor at trial initiation, and then after approximately 10-20% of patients have been recruited, and then regularly (at least yearly) thereafter and at trial closeout, or more frequently if so required; e.g. if the fieldwork is about 18 months, this means that each site is visited approximately four times by external monitors. The results from each monitoring visit will help inform whether more frequent or earlier repeat visits are required.

Because of the existing international travel restrictions, the independent monitors will likely be recruited from local academic or commercial Contract Research Organisations (CROs) in-country. This will take place by visiting the participating clinical research sites. However, this can also take place remotely if on-site monitoring is not possible due to local social distancing restriction on travel and movement of people are in place, in which it will take place by teleconference calls and provision of electronic and photographic evidence of documentation and infrastructure.

Monitors will review the individual participant records, including consent forms, case report forms, supporting data, laboratory specimen records, and endpoints through laboratory and medical records (physicians' progress notes, nurses' notes, individuals' hospital charts), to ensure the protection of study participants, compliance with the protocol, and accuracy and completeness of records. The monitors also will inspect the sites' trial site and master files to ensure that requirements are being followed and the sites' pharmacies to review product storage and management.

10.12.2 Auditing

The independent clinical monitoring process will be audited by a study staff from the sponsor's research office at LSTM in Liverpool, and LSHTM in London, UK.

10.12.3 Role of sponsor

The sponsor reserves the right to suspend temporarily or prematurely discontinue this study at any time for reasons including, but not limited to, safety or ethical issues or severe non-compliance. If the sponsor determines such action is needed, it will discuss this with the investigator. When feasible, the sponsor will provide advance notification to the investigator of the impending action prior to it taking effect. The sponsor will promptly inform the ethics committees and provide the reason for the suspension or termination.

10.13 Other quality control measures

10.13.1 Safety monitors

A local paediatrician and physician will act as trial safety monitors, one per country.

10.13.2 Internal monitoring

Each of the partner's institutions has their own internal quality control teams who will conduct internal monitoring on a regular basis, and help prepare for external monitoring visits.

10.13.3 Training

The country Principal Investigators are responsible for the conduct of the study at the study sites, including delegation of specified study responsibilities, and training of study staff. The training will include instructions on the use of PPE and other infection prevention and control (IPC) measures (see section 12.7.3.1, Biohazard containment to limit risks of SARS-CoV-2 transmission, page 65). All staff involved with the consent procedures will have been trained to GCP or equivalent level.

Each site will maintain a record of all individuals involved in the study (medical, nursing and other staff) and will ensure that all persons assisting with the trial receive the appropriate training about the protocol, the investigational product(s) and their trial-related duties and functions, including formal certified GCP training. During the study, the regular spot checks will be conducted to assess the performance of the study site staff members and re-training provided where necessary.

10.13.4 Quality assurance/control of laboratory tests

Regular audits of laboratory performance will be completed by experienced supervisors according to standard operating procedures already in place in the respective laboratories.

11 STATISTICAL CONSIDERATIONS

11.1 SAMPLE SIZE DETERMINATION

Sample size calculations were conducted with PASS v20.

11.1.1 Cohort

The primary endpoint used for the sample size calculations is the cumulative proportion of patients that progresses at least one category in disease severity from enrolment to day-28 based on the WHO clinical progression scale (see 16.4, Appendix IV. WHO clinical progression scale, page 82). At least one category increase in disease severity is defined as progressing from either asymptomatic or mild disease at enrolment to at least moderate disease requiring hospital admission (excluding hospitalisations for isolation only), or from moderate disease at enrolment to severe disease, or from severe disease at enrolment to death. For definitions of each disease severity category, see 16.4, Appendix IV. WHO clinical progression scale, page 82. The sample size calculations to determine the crude impact (i.e., unadjusted for potential confounders) of malaria on COVID-19 disease regression suggests that a pooled sample size of 708 (673 completers) of whom approximately 142 are malaria positive and 566 are malaria negative (135 and 538 completers) would provide 90% power to detect a 50% difference in the primary endpoint from 30% in the malaria-negative group to 45% in the malaria positive group (alpha=0.05, 5% loss to follow-up by day 28). The true power is likely to be lower when the confounding effect of baseline differences between malaria and non-malaria patients (such as age is considered). However, the inflation in the sample size required is difficult to estimate a priori. Similar crude sample size calculations using 80% power suggest 522 participants are required. Thus overall, a sample size of 708 participants would provide 80% power to detect a 50% difference in the primary endpoint allowing for a 35.6% inflation in sample size (708/522=1.356) to take adjustment for confounding into account in multivariate or stratified analysis. The sample size calculation for the COVID-19 cohort study assumes that each country will recruit 354 COVID-19 participants and that approximately 20% of them are coinfected with malaria (RDT positive), representing a weighted average of 15% of 354 COVID-19 cases in Kenya and 25% of 354 cases in Burkina Faso.

11.1.2 Immunological components

Serological assays will involve all cohort participants. Detailed immunological assays such as the chemo/cytokine panel and T and B cell response will be conducted from the stored samples of approximately 45 malaria cases and a similar number of matched malaria negatives per country, i.e., approximately 90 per country or 180 overall. The sample size may increase if more funding becomes available.

11.1.3 Nested malaria treatment trial
Approximately 54 participants in Kenya are expected to have malaria (15% of 354) and will be enrolled in the nested treatment study comparing AL vs pyronaridine-artesunate (1:1). The corresponding number in Burkina Faso is 88 (25% of 354). Thus 142 pooled across the two countries. A study of 142 participants has 80% power the detect a 22.2% absolute increase in viral clearance by day-7 from 20% in the artemether-lumefantrine arm to 42.2% in the pyronaridine-artesunate arm (RR=2.11), assuming a 5% loss to follow-up by day 7 (134 completers) (two-sided alpha=0.05). The 20% viral clearance rate by day=7 in the control arm (artemether-lumefantrine) is based on a previous trial.[44]

Similarly, a sample of 142 participants has 80% power to detect a 50% relative decrease (RR=0.50) or increase (RR=1.50) in the cumulative seroconversion rate by day 14 from 50% in the artemether-lumefantrine arm (control arm) to either 25% or 75% in the pyronaridine-artesunate arm, assuming a 10% loss to follow-up (128 completers). The seroconversion rate of 50% by day 14 is based on previous observational COVID-19 studies in China.[45]

Although the nested malaria treatment trial will be exploratory in nature, the study design includes the option to conduct interim analyses as part of the emergency response to the pandemic to ensure that promising findings can be shared early (see 11.2.5.3, Interim analysis and early stopping rule for efficacy, page 59).

11.1.4 Screening study (cross-sectional survey source population)
A source population of 4,720 suspected cases (2,360 in each country) is required to recruit a cohort of 708 participants (354 per country). This assumes a SARS-CoV-2 test positivity rate of 20% across the two countries and eligibility of 75% among those testing positive (0.2 x 0.75 x 4,720 = 708). If the average malaria RDT test positivity in the source population is 20% across both countries pooled, then 4,720 suspected cases would consist of 234, 710, 710 and 3066 MAL+/C19+, Mal+/C19-, MAL-/C19-, and MAL-/C19- participants. Such a sample size with a ratio of 4:1 malaria-negative: malaria-positive participants would provide 80% power to detect a 25% difference from 18.6% to 23.3%, allowing for a 30% sample size inflation relative to a crude analysis that does not adjust for confounding (effective sample size of 3,630), or a 26% difference from 18.6% to 23.4% allowing for 35% sample size inflation (effective sample size 3,495) (two-sided alpha = 0.05).

11.1.5 Interim sample size re-estimations and feasibility of recruitment
This study is designed at a time when there are many uncertainties about the parameters needed for sample size calculations. A two-stage approach will be used that includes an 'internal pilot study' in approximately the first 20% of cases. This is then followed by a formal blinded interim sample size re-estimation to assess the test positivity for malaria and SARS-CoV-2 in the source population and the observed event and drop-out rate in the COVID-19 cohort study. The progression of the epidemic, malaria season and health system, and study site capacity will also be taken into account during the sample size re-estimation to determine the feasibility of the total sample size and if any potential expansion to more sites is required.

11.2 Statistical analysis

11.2.1 General principles
All statistical analyses will be described in detail in the Statistical Analysis Plan (SAP). All tests will be two-sided and unless otherwise defined and *P*-values <0.05 will be used to define statistical significance for analysis.

11.2.2 Populations for analysis

11.2.2.1 Screening study (cross-sectional survey source population) and COVID-19 cohort study

All participants in the screening study (cross-sectional survey, source population) and COVID-19 cohort study will contribute to the analysis of the associations with malaria and COVID-19 prevalence and severity.

11.2.2.2 Malaria treatment study

For the nested malaria treatment trial, the primary analyses will be based on the Intention-to-treat (ITT) population and secondary supportive analyses will be done on the Per-protocol (PP) population. The safety analysis will be performed on the safety population. These analytical populations are defined as follows: 1) The ITT population consists of all randomised subjects with valid informed consent; 2) The PP population is a subset of the ITT population, where subjects with major protocol deviations will be excluded. Major protocol deviations will be defined in the Statistical Analysis Plan (SAP). 3) The safety population is a subset of the ITT population, consisting of all randomised subjects who receive at least one dose or a partial dose of study drug.

11.2.3 Screening study (cross-sectional survey source population)

The primary analysis of the cross-sectional data is the estimation of malaria infection as a potential risk factor for COVID-19, adjusted for other predictors of COVID-19 prevalence. We will use log-binomial regression to obtain prevalence ratios (95% CI) of malaria among patients who test positive vs negative for SARS-CoV-2. The analysis will also be stratified by age groups and the presence of symptoms on enrolment. Similar approaches will be used for secondary analyses of the evaluation of other potential risk factors for COVID-19 and severity.

11.2.4 Analytical methods by type of endpoint measure

11.2.4.1 Binary endpoints

For single measurements of binary endpoints, including for the binary primary endpoints of the screening study (cross-sectional survey, source population), COVID-19 cohort study and the nested malaria treatment trial, a generalised linear model (GLM) with the log-link function and binomial distribution (log-binomial regression) will be used to compare endpoints between exposure groups (e.g. by malaria status at enrolment). Crude GLM models will have the predictor of interest (e.g. malaria status at enrolment) as the only predictor and ‘study site’ as covariates. Results will be expressed as the risk ratio (RR) with associated 95% CI. If the log-binomial regression models do not converge, the ‘binreg’ or ‘difficult’ options (in that order) will be specified in Stata. If all models fail to converge, log-Poisson regression with a sandwich variance estimator will be used. Because the effect of malaria on COVID-19 may be biased by predictors that are not equally distributed by other predictors of COVID-19 severity, such as age, we will also conduct adjusted analyses to determine whether the effect of malaria on COVID-19 is affected with the inclusion of additional covariables. For repeated measurements of binary endpoints, a generalised linear mixed model (GLMMIX) with the log-link function and binomial distribution (log-binomial regression) will be used.

11.2.4.2 Continuous endpoints

Continuous endpoints will be summarised using the number of subjects (n), mean, standard deviation (SD), geometric mean (95% CI), minimum, median (50th percentile), 25th and 75th percentiles and maximum by treatment group. For continuous endpoints with single measurements, a GLM model with a normal distribution and identity link function will be used, with the stratification factors ‘site’ and ‘presence of symptoms’ as covariates in the crude models and with additional co-

variates in adjusted models. Results will be expressed as the mean differences with their two-sided 95% CIs will be derived from the GLM model. For continuous endpoints with repeated measurements, a GLMMIX with the identity function and normal distribution will be used.

Non-normally distributed data will be transformed to obtain an approximately normal distribution. For example, antibody titres to COVID-19 will be summarised by geometric mean (the Geometric Mean Titre or GMT) (95% CI) by group, and groups compared using the Geometric Mean (Titre) Ratio (GMTR, or GMR) and associated 95% CIs (see also 11.2.4.4, Immunological responses, below).

If variables do not attain a normal distribution after transformation, results will be expressed and the median and 25% and 75% percentile by group and compared using the Wilcoxon rank-sum test.

For the analysis of the disease progression and clinical scales including the WHO clinical progression scale and the FLU-PRO scales, we will use change scores (95% CI) defined as the mean difference between the score at enrolment and the score by defined timepoints, or the maximum score achieved.

11.2.4.3 Analysis of count outcomes

The count outcome will be summarised using the number of events (e.g. days with fever, days with respiratory symptoms, days with low O2 saturation, etc.) and incidence rate by group and will be analysed by a GLM model with the predictor as exposure variable and with Poisson distribution and log link function, and the stratification factors 'study site' as covariates. Incidence rate ratio with their two-sided 95% CIs will be derived from the GLM model.

11.2.4.4 Immunological responses

Antibody titres to COVID-19 and malaria and other pathogens will be summarised by mean fluorescence index (MFI) and geometric mean titre (GMT) (95% CI) by group at different time points. The analysis will consider: C_{max}, maximum antibody signal allowing for Δ_C the maximum change in antibody signal (i.e. including baseline measure); t_{max}, time to maximum antibody signal which equates to seroconversion; $t_{1/2}$, the half-life of antibody signal (in days) after a maximum signal has been reached; and t_{neg}, modelled time until the signal is reduced to below the level of detection of the assay.

11.2.5 Additional statistical consideration for the nested malaria treatment trial

11.2.5.1 Baseline characteristics

The number of participants, their baseline demographics and characteristics, including age, height, weight, sex, race, and country etc., will be summarized by treatment group using descriptive statistics (mean, standard deviation, median, minimum and maximum for continuous and rates for categorical).

11.2.5.2 Assessment of efficacy

Similar approaches as for the COVID-19 cohort study will be used to assess treatment efficacy for binary, count and continuous data, using treatment arm as the main exposure variable of interest, and the stratification factors study site and presence of symptoms as co-variates in the crude analysis. Secondary analyses include covariate-adjusted analysis to determine whether the estimate of treatment-effect is affected with the inclusion of additional covariables, and per-protocol analysis, and sub-group analysis. Imputation for baseline missing covariates will be carried out before categorising. Assessment of the homogeneity of treatment effect by a subgroup variable will be conducted by a GLM with the treatment, subgroup variable, and their interaction term as predictors, and the P-value presented for the interaction term.

11.2.5.3 Interim analysis and early stopping rule for efficacy
Formal interim analyses will be conducted at the discretion of the Data Safety and Monitoring Board (DSMB), or at any other time point recommended by the DSMB.

The interim analysis will be performed by an unblinded statistician, who will prepare a closed report for DSMB review and recommendations. Analyses will be presented with blinded codes for treatment arms to protect against the possibility that the DSMB report may fall into the wrong hands.[46] The DSMB will have access to the allocation code and will discuss or report the results of the interim analysis with the Trial Management Group (TMG), e.g. in a joint meeting. The DSMB, together with the Chief and Principal Investigators, decide on the continuation of the study.

The Lan-DeMets spending function with Haybittle-Peto type boundaries will be employed as a guide for the DSMB. Under the Haybittle-Peto rule, the recruitment can be stopped during any of the interim analyses with a type I error probability of 0.001 for the primary endpoint while preserving the overall type I error rate for efficacy at the α=0.05 level at the final analysis.

Statistics will not be the sole basis for the decision to stop or continue, and the DSMB can advise to continue recruiting in the trial, or stop recruiting but continue to complete the intervention as per randomization in the remaining active participants, even if statistically the stopping boundary is crossed, e.g. in order to continue collecting more safety or immunological information or data for further sub-group analyses etc. The trial will not be stopped in case of futility unless the DSMB during the course of safety monitoring advises otherwise.

A detailed plan for interim analysis, the provisional stopping rules and how the stopping rules will be applied, will be drawn up prior to the start of the interim analysis and documented in the study statistical analysis plan.

In addition, the DSMB will review the quality of the trial data and the safety data at every scheduled meeting.

11.2.5.4 Safety analysis
For the safety analysis, data from all subjects from the safety analysis population will be included. All analyses will be descriptive. Data will be summarized by treatment group. The percentage of subjects with at least one AE during the follow-up period will be tabulated with exact 95% CI (two-sided). No multiplicity adjustment will be implemented in analysis. The percentage of subjects with at least one report of unsolicited AE and reported up to 28 days after enrolment will be tabulated with exact 95% CI.

The occurrence of SAEs will be determined on the safety analysis population. The proportion of subjects with an SAE, classified by the MedDRA preferred term level, reported from study start until study conclusion will be tabulated with exact 95% CI. Comparisons between groups will be made using Fisher's Exact Test for each preferred term. Serious adverse events (SAEs) occurring at any point during the trial will be summarised, and relatedness to treatment will be assessed.

11.2.6 Missing data

11.2.6.1 Baseline covariates
Missing baseline covariates in the overall COVID-19 cohort study and nested malaria treatment trial will be imputed using simple imputation methods in the covariate-adjusted analysis based on the covariate distributions, should the missing values for a particular covariate be less than 5%. For a continuous variable, missing values will be imputed from random values from a normal distribution

with mean and SD calculated from the available sample by age group and study site and malaria status. For a categorical variable, missing values will be imputed from random values from a uniform distribution with probabilities P_1, P_2, …, and P_k from the sample. The seed for the imputation will be set as a number with 8 digitals (e.g. the date of the programming).

If the missing values for a covariate are ≥5%, then they will be imputed using Markov chain Monte Carlo (MCMC) methods.

11.2.6.2 Efficacy outcomes

For the primary analysis, a complete case analysis will be performed, and no imputation for missing efficacy outcome values will be performed.

11.2.7 Multiplicity

Issues of multiplicity will not be considered in this exploratory study.

11.2.8 Pharmacokinetic analysis

Compartmental Population Pharmacokinetic Modelling will be performed using Monolix 2018R2 to establish the pharmacokinetic profile throughout the population and relate to covariates, including patient sex, weight, BMI and malaria/COVID-19 disease status. The pharmacokinetic analysis will also be performed on quantified drug concentrations within saliva samples to establish drug distribution to this compartment and related to predicted lung concentrations using Physiologically Based Pharmacokinetic (PBPK).

Pharmacokinetic-Pharmacodynamic modelling will be similarly performed using compartmental analyses Monolix by linking drug exposure to drug effects quantified through SARS-CoV-2 expression from nasal swabs collected at similar time points to blood samples. Other covariates relating to patient symptoms and recovery status and time will be studied in parallel and separate analyses to investigate any potential PKPD interactions.

12 ETHICS AND DISSEMINATION

12.1 DECLARATION OF HELSINKI

The trial will be conducted in compliance with the principles of the Declaration of Helsinki (1996) (See section 16.3, Appendix III. Declaration of Helsinki, page 79), the principles of GCP, and in accordance with all applicable regulatory requirements in Kenya and Burkina Faso.

12.2 RESEARCH ETHICS AND REGULATORY APPROVAL

12.2.1 Review process

This protocol, the informed consent document, patient information sheets will be reviewed in Kenya, by KEMRI's Scientific and Ethics Review Unit and the Kenyan Pharmacy and Poisons Board and in Burkina Faso, by the National Ethical Committee and the Technical Committee for Clinical Trials (*Comité Technique pour les Essais Cliniques [CTEC])* of the Ministry of Health. In addition, the protocol will require approval from the Research and Ethics Committee of Liverpool School of Tropical Medicine, Liverpool (LSTM) and the London School of Hygiene and Tropical Medicine (LSHTM).

12.2.2 Protocol amendments

If it is necessary for the protocol to be amended, the protocol amendment will be submitted to the research ethics committees at LSTM and LSHTM (sponsors) and the primary ethics committees in

each country for approval before implementation in that country. Any change to the informed consent form, except for layout, spelling errors and formatting, must also be approved by the sponsor and the primary ethics committee in each country, before the revised form is used.

No change will be made to the approved protocol without the agreement of the sponsor. The Chief Investigators, or a delegated person, will distribute amendments on behalf of the sponsor to each principal investigator, who in turn is responsible for the distribution of these documents to the staff at his/her study site.

12.2.3 Sanctioning of the protocol by hospitals

Where this is a requirement of participating hospitals, the approved protocol will subsequently be submitted to the participating hospital's review committees or representatives for subsequent sanctioning of the protocol and procedures by the hospital.

12.2.4 Regulatory approval

Regulatory approval for the nested malaria treatment trial will be sought from the national regulators in Kenya and Burkina Faso, respectively. Both artemether-lumefantrine and pyronaridine-artesunate are currently approved under various brand names in each of the countries.

12.3 INFORMED CONSENT PROCEDURES

Informed consent and assent will be obtained before participants are enrolled in the screening study (cross-sectional survey, source population) and again when they are eligible for the COVID-19 cohort study. Assent will be obtained for children age 12-19 years in Burkina Faso and 13-17 years (inclusive) in Kenya. See section 16.12, Appendix XII. Participant information sheets and informed consent and assent statements, page 125, for further details.

12.3.1 Consent procedures

Written informed consent and assent will be obtained in the local vernacular language (Kenya: Kiswahili, Dholuo, Luhya, Igikuria, Iteso; Burkina Faso: French). The consent process will be initiated at the time of enrolment into the study and will continue throughout the participant’s participation. Potential participants will be made aware of the existence of the study by routine staff or by study staff working alongside the routine staff. If the participant meets the study enrolment criteria, the full consent process will follow, with a written consent/assent form provided. The consenting/assenting procedures will be conducted by trained staff who will answer any questions the participants may have. A copy of the informed consent/assent document will be given to the participant for their records unless they state that they do not wish to have a copy.

For illiterate participants, an independent witness will be present during the informed consent process and will sign the consent form as a witness, while the participant will be asked to indicate consent by use of thumbprint. The participant may withdraw consent at any time throughout the course of the study, and this will be made clear in the informed consent/assent process. A copy of the informed consent /assent document will be given to the participant or their caregiver for their records unless they state that they do not wish to have a copy.

All individuals will be informed that there is no requirement to join the study and that standard medical care will remain the same regardless of study enrolment. If the participant or their caregiver chooses not to be enrolled in the study, they will be allowed to continue with routine care provided by the health facility without any consequences to the expected level of care provided.

12.4 Protection of privacy and confidentiality

12.4.1 Privacy

Personal and medical information relating to research participants will be treated as confidential. The risk of disclosure will be minimized by secure storage of documents and use of linked data by replacing personal identifiers with a unique study code to conceal the identity of the participant.

12.4.2 Privacy of individual

Individual data such as PCR results for SARS-CoV-2, RDT tests for malaria and anaemia will be reported to the participant at point of care, to relevant study staff and where appropriate will be recorded in the participants' medical record book in addition to CRFs.

12.4.3 Confidentiality of data

All information regarding the participants will remain confidential to the extent allowed by law. Unique numerical identifiers will be used for data entry. All screening forms and case report forms will be kept in a secured location with access limited to authorized study staff. Unique numerical identifiers will be used for the computer-based data entry and blood samples. Publications will contain only aggregated data. No identifying information will be included to ensure individual patient anonymization of all data and results made public.

12.5 Declaration of interest

None of the chief or principal investigators has paid consultancies with the pharmaceutical companies involved in the trial, or other competing interest for the overall trial or in each study site.

12.6 Access to source data/documents

In addition to the clinical monitors, authorised representatives of the sponsor/CRO, an IEC/IRB or regulatory authority may visit the study site to perform audits or inspections, including source data verification. The investigator agrees to allow the sponsor and CRO representatives, including the monitor and study safety monitor, the DSMB, the IRB/IEC and regulatory authority direct access to source data and other relevant documents.

12.7 Risks and benefits

12.7.1 Risks to Study Participants

12.7.1.1 Safety of the study drugs

See section 7.2.1, Risks associated administration with artemether-lumefantrine, page 34 and section 7.2.2, Risks associated with pyronaridine-artesunate, page 35.

12.7.1.2 Procedures for participants developing acute respiratory symptoms during the trial

The study will be conducted in an environment where national guidelines in each country may change during the course of the study that determines which patients are admitted in hospital or kept in isolation wards versus which patients with no or mild symptoms are asked to self-isolate at home. If after enrolment initially asymptomatic patients develop symptoms, or if symptoms worsen consistent with severe COVID-19, the participant will be asked to alert the study team by phone,

such that transport arrangements can be made for the participant to visit the study clinic and/or hospital or for a home visit.

12.7.1.3 Blood and tissue sampling

The additional protective measure used for sampling from COVID-19 patients is discussed in more detail in section 12.7.3.1, Biohazard containment to limit risks of SARS-CoV-2 transmission, page 65.

All examinations undertaken as part of this study will be non-invasive, with the exception of blood sampling and swabs. Blood sampling may be inconvenient to the participants and may cause minor discomfort and bruising. In some aspects of the trial, blood sampling has the potential to directly benefit the participants or their babies, as any malaria infection or anaemia detected as a result of the sampling will be treated. In other aspects of the trial, such as the immunological assays, will not be of direct benefit to the individual participants, but the potential knowledge gained will eventually be used to benefit all populations in areas where malaria and COVID-19 co-exist.

12.7.1.4 Maximum blood volume

In adults and older children above the minimum age of assent, a blood sample of no more than 20mL per sample will be taken at days 1 (enrolment) and day-28 (close-out) and 15mL on each of the five scheduled visits up to day 42 inclusive (day 3, 7, 14, 21 and 42) and during the extended follow at 3, 6, and 12 months. Thus the total cumulative volume of blood collected from each participant will be small (130.25mL in the first three months [0.25 mL at screening, 2 further blood draws of 20mL and 5 blood draws of 15mL in the first 42 days plus one 15mL blood draw at three months) and 160.25 mL in total over 12 months [two additional 15mL blood draws at 6 and 12 months) and well within the accepted limit of 500mL over three months as stipulated by the KEMRI–Wellcome Trust Research Programme, Kilifi, Kenya guidelines for the maximum volume per participant[47] and other international guidelines.[48, 49] Where possible routine care and study-specific samples will be combined in a single blood draw.

Table 2 Maximum blood volumes in children per bodyweight category

Weight in Kg in weight category	5.5[b] – <10 kg	10 – <15 kg	>=15kg – <12/13 yrs	>=12/13 years (~>=27.7kg)[a]
Maximum weight in Kg in weight category	<10	<15	<22.2	
Screening study volume (mL)	0.25	0.25	0.25	0.25
Cohort study Days 1, 3, 7, 14, 21, 28, 42 (mL)	7x2.5=17.5	7x5=35	7x7.5=52.5	(2x20) + (5x15) = 115
3 month follow-up visit (mL)	2.5	5	5	15
Cumulative volume by 3 m (mL)	20.25	40.25	57.75	130.25
Cumulative volume by 3 m, safety limit (mL)[c]	27.5	50	75	138.5
6 & 12 month follow-up visits (mL)	2x2.5=5	2x5=10	2x5=10	2x15=30
Cumulative volume by 12 m (mL)	25.25	50.25	67.75	160.25

[a] This is the minimum (1st percentile) expected bodyweight expected for 12 or 13-year-old girls (who are lighter than boys), the age of assent in Burkina Faso and Kenya respectively. Based on the 1st percentile for weight-for-age of 10 year olds[50] and an average weight gain of 25% in girls between 10 and 12 years of age.[51]

[b] This is the minimum expected bodyweight for a six months old child (the lower age threshold for inclusion in the study) based on the 1st percentile of the WHO weight-for-age growth reference[50]

[c] Cumulative volume of blood that is considered safe in children for repeated blood draws taken for research purposes over a three month period (in addition to the blood taken for routine clinical care), based on a maximum of 5ml/kg body weight[47]

Younger children below the age of assent (Burkina Faso 12 years, Kenya 13 years) will be excluded from the pharmacokinetic sampling to minimize the blood volume in younger children as the pharmacokinetic sample requires 5mL. Children below 10kg bodyweight will have 2.5mL instead of 20mL or 15mL blood draws taken during follow-up in addition to the finger/heel prick on Day-0 (250 uL) (a cumulative total of 20.25mL by over three months and 25.25 mL over 12 months). This ensures that even the lightest six months old children (the minimum age at enrolment) remain below the safety limit of 5mL/kg[47] over three months (corresponding to a bodyweight of 4.1 kg for eight blood draws of 2.5 mL per sample) because the first percentile at the age of six months of the WHO weight-for-age reference is 5.5kg in girls.[50] Similarly, the volume for children weighing 10-<15kg will be 5mL per draw, and 7.5mL for children weighing 15kg or more until the age of assent. At the age of assent (12 years in Burkina Faso and 13 years in Kenya), they are eligible for the nested malaria treatment trial and 20mL (Days 1 and 28) or 15mL (days 3, 7, 14, 21, 42) per sample will be taken, or 120.25mL over three months and within the 3-month safety limit of 138.5mL (based on the 1st percentile for weight-for-age of 10 year old[50] and an average weight gain of 25% in girls between 10 and 12 years of age.[51]). The cumulative volume by 12 months will be 130.25mL (see Table 2, above).

Figure 5: Maximum blood volume per bodyweight in children for research in addition to blood taken for routine clinical care

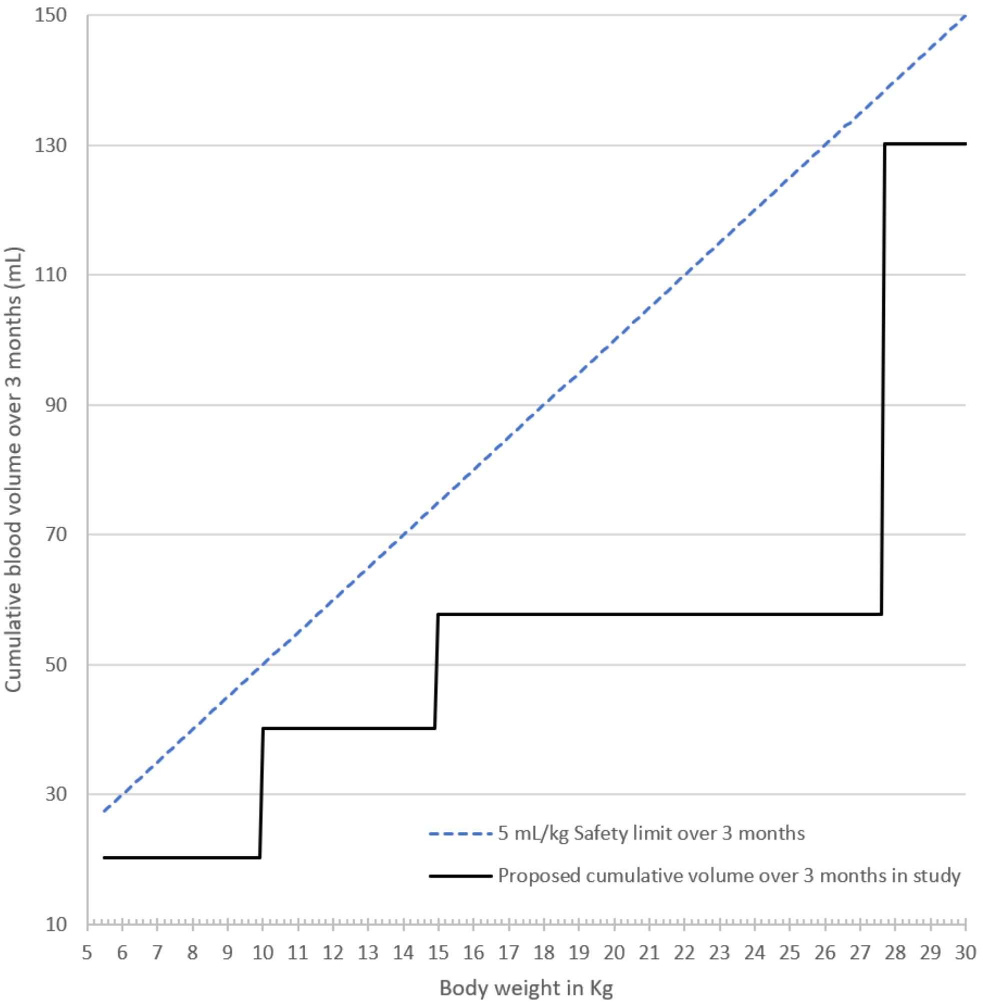

12.7.2 Benefits to study participants or society

By taking part in this study, participants will potentially benefit from close observations. Participants who do not attend scheduled appointments will receive reminders and active follow-up. Participants

experiencing illness between visits will be seen and treated free of charge as part of the study, although this is already routine practice in both countries.

There is a potential benefit to society as participation in the study will help investigators to obtain a better understanding of the impact of COVID-19 on people's health in malaria-endemic areas.

12.7.3 Risk to the population and study staff and biohazard containment

12.7.3.1 Biohazard containment to limit risks of SARS-CoV-2 transmission

The design is based on practical considerations that both Kenya and Burkina Faso are implementing social distancing and stay at home strategies which restrict free movement and make home visits more challenging. The procedures for identifying a case and the subsequent isolation and management will follow local and national guidelines in each country, and the study will interfere as little as possible in the usual local investigation and management of COVID-19 cases unless this is deemed necessary to comply with the research team's responsibilities to provide adequate care to research participants.

As the transmission of SARS-CoV-2 and other respiratory droplet pathogens can occur through contact with respiratory droplets and contaminated surfaces, precautions will be employed by all personnel in the handling of all specimens for this study. Strict adherence to personal protection equipment (PPE) and other infection prevention and control (IPC) measures will be followed according to local guidelines in each country to minimize the risk of transmission of SARS-CoV-2. The recommended types of PPE per activity will follow national guidelines in Kenya and Burkina Faso. These are currently based on the WHO guidelines for 'Recommended type of personal protective equipment (PPE) to be used in the context of COVID-19 disease, according to the setting, personnel and type of activity'[52] (see 16.6, Appendix VI. Personal protection equipment guidelines for study staff by risk category, page 85). All swabs and other biological samples will be taken by a trained study team member as per national standard operating procedures with strict adherence to these PPE guidelines. Also, to limit exposure in clinic waiting rooms, dedicated appointments will be made to collect clinical specimens.

Staff will be trained to remember to observe hand hygiene and standard precautions, including adherence to hand hygiene by washing with soap and water or using alcohol-based hand sanitizer immediately after removing gloves and other equipment and after any contact with respiratory secretions. Full personal equipment should be worn comprising of PPE suit, N95 mask, goggles or face shield, hair protection, while obtaining nasopharyngeal swabs for suspected cases and undertaking aerosol-generating procedures (e.g., bronchoscopy, elective intubation, suctioning, administering nebulized medications, dental procedures). The study-specific procedure will be undertaken in well-ventilated separate rooms or areas, such as dedicated tents that are away from the routine clinical areas.

For the safe transport of biological substances, see section 10.7.2, Shipping, page 48. For the local, within country, transport of dangerous goods and materials, including diagnostic specimens and infectious substances, we will follow by local Governmental Regulations.[32]

12.7.3.2 Community sensitisation and public engagement for home visits

Continuous community sensitisation and public engagement will be used, as per the national guidelines in each country to mitigate the risk to the staff and participants to allow home visits. Similar strategies are used by national Governments in each country for contract tracing.

12.7.3.3 Transport arrangements

Safe transportation can also include a car with three or fewer occupants where all the occupants, including the sick person, use facial masks. Public transport (matatus, buses) should not be used to transport sick individuals to the nearest isolation facilities as these are likely to propagate the spread of infection. If safe transport is not available, then a dedicated study vehicle with trained drivers and appropriate personnel and PPE will be dispatched to pick the patient. Trained drivers will be provided with face masks and other PPE and disinfectant to clean the cars after each trip. The study team is working in close collaboration with the Ministry of Health to arrange dedicated COVID-19 ambulances with oxygen supply and ambulance staff in full PPE for the transport of any patients requiring urgent hospitalisation.

12.7.4 Other ethical considerations

Long term samples storage for future studies and shipment of samples to external laboratories

With respect to applicable country regulations, written informed consent/assent will be sought from participants for long term storage of their samples for future research, which permits long-term storage of samples for 25 years in Kenya and 10 years in Burkina Faso. When consent for long-term storage for future research is not granted by the participant, the surplus sample volume will be destroyed following the completion of this study.

Informed consent will also be sought for the shipping of their samples to external laboratories overseas for relevant analyses. It will be clarified that the results of these assays will not be used for patient care.

12.8 Ancillary and post-trial care

12.8.1 Health care during the trial

All care directly related to the proper and safe conduct of the trial, and the treatment of immediate adverse events related to trial procedures will be provided free of charge by the study in the study hospitals. The provision of ancillary care beyond that immediately required for the conduct of the trial will not be covered by the trial.

12.8.2 Trial insurance

The Liverpool School of Tropical Medicine and the London School of Hygiene & Tropical Medicine hold Public Liability ("negligent harm") and Clinical Trial ("non-negligent harm") insurance policies which apply to this study. This will include cover for additional health care, compensation or damages whether awarded voluntarily by the Sponsor, or by claims pursued through the courts. The liability of the manufacturer of the trial drugs is limited to those claims arising from faulty manufacturing of the commercial product and not to any aspects of the conduct of the study.

12.8.3 Post-trial care

The study budget is not in a position to fund post-study care.

12.9 Expenses reimbursement and incentives

The study will provide payment for all study drugs, study procedures, study-related visits and reasonable medical expenses that are incurred in study clinics or hospitals as a result of the study, including expenses for transport for any study-related visits including unscheduled visits in between scheduled visits to study clinics. The study will not cover the costs of any non-study related events, including scheduled or unscheduled surgery or trauma-related events (e.g. accidents, burns etc.) if this is not deemed to be related to the study by the principal investigators or their representative.

Table 3: Reimbursement of expenses and incentives provided by the study

To Whom	What	Approximate Amount
Hospital	Improvement of infrastructure where required	up to ~$10,000/hospital
	Training of routine staff adult ward	~ $3,000/hospital
	Study procedure costs and study drugs and admission fees for inpatients	~ $100,000/hospital
Participant	Travel expenses for the participant and for one accompanying person/caregiver.	Up to ~$10 per person per round trip*
	Average compensation for loss of daily wages for each day they are scheduled to come to the hospital or clinic	~$ 5 per day (excluding any travel expenses)**

* A fixed rate of 5,000 XOF/CFA or 950 KES (~ $9 USD) will be given per visit for the combined compensation for the participant's or caretaker's time and travel expenses. In Kenya, the average travel expenses are about KES 400 (~$4) per visit, however, in exceptional cases, higher amounts (>$10) of travel expenses can be reimbursed if distance or transport modes requires this. This would need to be decided on a case by case basis, courtesy of the site PI/coordinator. In addition, they will receive ~$5 USD per day for loss of daily wages for each day they are scheduled to come to the hospital or clinic (i.e. a total of about $9 USD for travel and loss of daily wages).

12.10 Dissemination and application of the results

12.10.1 Result dissemination and publication policy

Our research findings will be communicated to country-level stakeholders including the study participants, reproductive health and malaria programmes/national Ministries of Health, and the research partner institutions in Kenya and Burkina Faso, and to other national and international research partners, NGOs, technical agencies and implementing partners, donor organizations, WHO, UNICEF, Medicines for Malaria Venture (MMV) and Shin Poong Pharm. Co., Ltd, the licence holders of the FLU-PRO questionnaire (NIH) and the general public. We will use multiple communication strategies to reach each target audience, as outlined below. Specifically, we will

- Share results early with National policymakers and implementers (MOH Kenya and MOH Burkina Faso)
- Share results early with WHO's Global Malaria Programme (GMP) (prior to publication)
- Share results early with the Imperial College modelling group and any other COVID-19 modelling groups
- Share results early with international policy implementers and funders of malaria control programmes such as the President's Malaria Initiative (PMI)
- Present results at scientific planning meetings and international conferences
- Peer-reviewed publication with early access via pre-print servers for health sciences https://www.medrxiv.org/

12.10.2 Impact

See section 3.2, Problem statement and Justification for the study, page 23, for potential impact.

12.10.3 Authorship and publications

Potential authors include all professionals that have participated in the study for a minimum of four of the 18 months. Authorship of any presentations or publications arising from this study will also be governed by the principles for authorship criteria of the International Committee of Medical Journal

Editors has designed.[53] Disputes regarding authorship will be settled by the publications committee, with further involvement of the independent chair of the DSMB if so required. The manufacturer of the study medication will be provided with a draft of the manuscript but will have no role in the review, data interpretation, or writing of the article.

12.10.4 Data Sharing Statement

We will encourage data sharing to ensure that the scientific potential of this study is maximized.

12.10.4.1 Types of data

We anticipate that five different datasets will be generated by this project:

- An epidemiological data set containing cross-sectional data from the source population, i.e. including SARS-CoV-2 positive and negative patients, with and without malaria co-infection. This is estimated to include data from approximately 4000 to 5000 participants depending on the test positivity rate for SARS-CoV-2.
- A clinical and serological cohort dataset that includes approximately 708 COVID-19 patients with and without malaria
- An immunological cohort data set with all immunological parameters from a subset of approximately 180 participants
- An antimalarial treatment response data set involving the participants enrolled in the nested malaria treatment trial, including the corresponding safety data.
- A PK/PD data set on the sub-group of participants included in the pyronaridine-artesunate treatment component

12.10.4.2 Discovery by potential users of the research data

The public datasets, study protocol and statistical analysis plan, will be shared through the World-Wide Antimalarial Resistance Network (WWARN) platform (http://www.wwarn.org/working-together/sharing-data/data-available-research-community) with a URL link provided in the data sharing section of publications, any study-specific website and WWARN's website. Our policy to data sharing, process and timelines will be provided in manuscripts and on the study website and the WWARN website. The immunological data will also be made publicly available on the IMMPORT database of NIH/NIAID. The results and anonymised data set of the FLU-PRO questionnaire will be shared with the Licence holders of FLU-PRO (NIH).

12.10.4.3 Governance of access

Biological samples will be shared using material transfer agreements with the collaborating institutions (see 2.1.4 Collaborators, page 11) to minimise the risk of unauthorised analysis beyond the scope of the agreed parameters.

The full anonymized research database will be made publicly available as soon as the full study findings have been published or based on any data requests that may occur during the study or analysis is still ongoing. For the databases, we will use a controlled access approach. The data sharing policy will be provided in publications and on the respective websites. This will state that we encourage data requests. Data access will be provided to researchers after a proposal has been approved by an independent review committee identified for this purpose. An agreement on how to collaborate will be reached based on any overlap between the proposal and any ongoing efforts. Proposals can be directed to email addresses provided in the publications and websites. To gain access, data requesters will need to sign a data-sharing agreement.

The only limits to data sharing will be to safeguard research participants' confidentiality. The proposed procedures for data sharing will be set out clearly in the participant information sheet with

current and potential future risks associated with this explained to the research participants as part of the informed consent process. External users will be bound by data-sharing agreements in line with the Data Sharing Policy from the respective Sponsors and the Gates Foundation to ensure that the privacy of individuals is protected. The agreement will prohibit any attempt to (a) identify study participants from the data or otherwise breach confidentiality, (b) make unapproved contact with study participants.

13 Timeframe and duration of the study

13.1 Timeline

The total duration of the project is projected to take 18 months, including 3 months for the preparation and approximately 12 months of patient recruitment and 3m for laboratory analysis and write up (see 13.2, Gantt chart, page 69).

13.2 Gantt chart

	2020			2021			
	Q2	Q3	Q4	Q1	Q2	Q3	Q4
**Activity**							
Protocol development	■						
IRB approval / Annual Review	■	■			■		
Regulatory approval/ Annual Review	■	■			■		
CRF and SOP development	■						
Drug and placebo preparation	■						
Drug shipment	■						
Enrolment Kenya		■	■	■	■	■	■
Enrolment Burkina Faso		■	■			■	■
Study		■	■	■	■	■	■ .
Closure							■
Immunological assays			■		■		■
Interim sample size re-estimation			■		■		■
Observational cohort analysis			■		■		■
Manuscript and results dissemination							■

14 FINANCIAL ASPECTS AND CONFLICT OF INTEREST

14.1 FUNDING FOR THE TRIAL

Funding to conduct the trial is provided by the Bill and Melinda Gates Foundation. In Kenya, in-kind co-funding is provided by the US Centers for Disease Control and Prevention (CDC).

14.2 PROVISION OF STUDY DRUGS

Pyronaridine-artesunate will be provided free of charge by Shinpoong Pharm.co., Ltd, South Korea. Artemether-lumefantrine will be purchased. When so requested by the manufacturer, the study will provide copies of safety reports of SAEs and AEs to the manufacturers (expedited where required). The manufacturer will not be involved in the design of the trial.

14.3 BUDGET & BUDGET JUSTIFICATION

See section 16.8, Appendix VIII. Budget and budget justification, page 116

15 REFERENCES

1. CDC COVID-19 Response Team, 2020. Preliminary Estimates of the Prevalence of Selected Underlying Health Conditions Among Patients with Coronavirus Disease 2019 — United States, February 12–March 28, 2020. . MMWR Morb Mortal Wkly Rep 2020;69:382–386.: 382–386.
2. Lighter J, Phillips M, Hochman S, Sterling S, Johnson D, Francois F, Stachel A, 2020. Obesity in patients younger than 60 years is a risk factor for Covid-19 hospital admission. Clin Infect Dis.
3. Zhou F, Yu T, Du R, Fan G, Liu Y, Liu Z, Xiang J, Wang Y, Song B, Gu X, Guan L, Wei Y, Li H, Wu X, Xu J, Tu S, Zhang Y, Chen H, Cao B, 2020. Clinical course and risk factors for mortality of adult inpatients with COVID-19 in Wuhan, China: a retrospective cohort study. Lancet 395: 1054-1062.
4. Onder G, Rezza G, Brusaferro S, 2020. Case-Fatality Rate and Characteristics of Patients Dying in Relation to COVID-19 in Italy. JAMA.
5. Wu Z, McGoogan JM, 2020. Characteristics of and Important Lessons From the Coronavirus Disease 2019 (COVID-19) Outbreak in China: Summary of a Report of 72314 Cases From the Chinese Center for Disease Control and Prevention. JAMA.
6. Gutman JR, Lucchi NW, Cantey PT, Steinhardt LC, Samuels AM, Kamb ML, Kapella BK, McElroy PD, Udhayakumar V, Lindblade KA, 2020. Malaria and Parasitic Neglected Tropical Diseases: Potential Syndemics with COVID-19? Am J Trop Med Hyg.
7. Das D, Grais RF, Okiro EA, Stepniewska K, Mansoor R, van der Kam S, Terlouw DJ, Tarning J, Barnes KI, Guerin PJ, 2018. Complex interactions between malaria and malnutrition: a systematic literature review. BMC Med 16: 186.
8. Mooney JP, Butler BP, Lokken KL, Xavier MN, Chau JY, Schaltenberg N, Dandekar S, George MD, Santos RL, Luckhart S, Tsolis RM, 2014. The mucosal inflammatory response to non-typhoidal Salmonella in the intestine is blunted by IL-10 during concurrent malaria parasite infection. Mucosal Immunol 7: 1302-11.
9. Lokken KL, Stull-Lane AR, Poels K, Tsolis RM, 2018. Malaria Parasite-Mediated Alteration of Macrophage Function and Increased Iron Availability Predispose to Disseminated Nontyphoidal Salmonella Infection. Infect Immun 86.
10. Thompson MG, Breiman RF, Hamel MJ, Desai M, Emukule G, Khagayi S, Shay DK, Morales K, Kariuki S, Bigogo GM, Njenga MK, Burton DC, Odhiambo F, Feikin DR, Laserson KF, Katz MA, 2012. Influenza and malaria coinfection among young children in western Kenya, 2009-2011. J Infect Dis 206: 1674-84.
11. Edwards CL, Zhang V, Werder RB, Best SE, Sebina I, James KR, Faleiro RJ, de Labastida Rivera F, Amante FH, Engwerda CR, Phipps S, Haque A, 2015. Coinfection with Blood-Stage Plasmodium Promotes Systemic Type I Interferon Production during Pneumovirus Infection but Impairs Inflammation and Viral Control in the Lung. Clin Vaccine Immunol 22: 477-83.
12. Giannis D, Ziogas IA, Gianni P, 2020. Coagulation disorders in coronavirus infected patients: COVID-19, SARS-CoV-1, MERS-CoV and lessons from the past. J Clin Virol 127: 104362.
13. Oxley TJ, Mocco J, Majidi S, Kellner CP, Shoirah H, Singh IP, De Leacy RA, Shigematsu T, Ladner TR, Yaeger KA, Skliut M, Weinberger J, Dangayach NS, Bederson JB, Tuhrim S, Fifi JT, 2020. Large-Vessel Stroke as a Presenting Feature of Covid-19 in the Young. N Engl J Med 382: e60.
14. Klok FA, Kruip M, van der Meer NJM, Arbous MS, Gommers D, Kant KM, Kaptein FHJ, van Paassen J, Stals MAM, Huisman MV, Endeman H, 2020. Confirmation of the high cumulative incidence of thrombotic complications in critically ill ICU patients with COVID-19: An updated analysis. Thromb Res 191: 148-150.
15. Srichaikul T, 1993. Hemostatic alterations in malaria. Southeast Asian J Trop Med Public Health 24 Suppl 1: 86-91.
16. Angchaisuksiri P, 2014. Coagulopathy in malaria. Thromb Res 133: 5-9.
17. Sarma P, Kaur H, Kumar H, Mahendru D, Avti P, Bhattacharyya A, Prajapat M, Shekhar N, Kumar S, Singh R, Singh A, Dhibar DP, Prakash A, Medhi B, 2020. Virological and Clinical Cure in Covid-19 Patients Treated with Hydroxychloroquine: A Systematic Review and Meta-Analysis. J Med Virol.
18. Pastick KA, Okafor EC, Wang F, Lofgren SM, Skipper CP, Nicol MR, Pullen MF, Rajasingham R, McDonald EG, Lee TC, Schwartz IS, Kelly LE, Lother SA, Mitjà O, Letang E, Abassi M, Boulware DR, 2020. Review: Hydroxychloroquine and Chloroquine for Treatment of SARS-CoV-2 (COVID-19). Open Forum Infectious Diseases.
19. Taccone FS, Gorham J, Vincent JL, 2020. Hydroxychloroquine in the management of critically ill patients with COVID-19: the need for an evidence base. Lancet Respir Med.

20. Taylor DN, Wasi C, Bernard K, 1984. Chloroquine prophylaxis associated with a poor antibody response to human diploid cell rabies vaccine. Lancet 1: 1405.
21. Shippey EA, Wagler VD, Collamer AN, 2018. Hydroxychloroquine: An old drug with new relevance. Cleve Clin J Med 85: 459-467.
22. Endy TP, Keiser PB, Cibula D, Abbott M, Ware L, Thomas SJ, Polhemus ME, 2020. Effect of Antimalarial Drugs on the Immune Response to Intramuscular Rabies Vaccination Using a Postexposure Prophylaxis Regimen. J Infect Dis 221: 927-933.
23. Townsend KN, Hughson LR, Schlie K, Poon VI, Westerback A, Lum JJ, 2012. Autophagy inhibition in cancer therapy: metabolic considerations for antitumor immunity. Immunol Rev 249: 176-94.
24. Otta DA, de Araujo FF, de Rezende VB, Souza-Fagundes EM, Eloi-Santos SM, Costa-Silva MF, Santos RA, Costa HA, Siqueira-Neto JL, Martins-Filho OA, Teixeira-Carvalho A, 2018. Identification of Anti-Trypanosoma cruzi Lead Compounds with Putative Immunomodulatory Activity. Antimicrob Agents Chemother 62.
25. Uzun T, Toptas O, 2020. Artesunate: could be an alternative drug to chloroquine in COVID-19 treatment? Chinese Medicine 15: 54.
26. Lane TR, Massey C, Comer JE, Anantpadma M, Freundlich JS, Davey RA, Madrid PB, Ekins S, 2019. Repurposing the antimalarial pyronaridine tetraphosphate to protect against Ebola virus infection. PLoS Negl Trop Dis 13: e0007890.
27. Funck-Brentano C, Ouologuem N, Duparc S, Felices M, Sirima SB, Sagara I, Soulama I, Ouedraogo JB, Beavogui AH, Borghini-Fuhrer I, Khan Y, Djimde AA, Voiriot P, 2019. Evaluation of the effects on the QT-interval of 4 artemisinin-based combination therapies with a correction-free and heart rate-free method. Sci Rep 9: 883.
28. D'Alessandro U, Ubben D, Hamed K, Ceesay SJ, Okebe J, Taal M, Lama EK, Keita M, Koivogui L, Nahum A, Bojang K, Sonko AA, Lalya HF, Brabin B, 2012. Malaria in infants aged less than six months - is it an area of unmet medical need? Malar J 11: 400.
29. World Health Organization, 2015. Guidelines for the treatment of malaria, third edition: World Health Organization,.
30. Croft SL, Duparc S, Arbe-Barnes SJ, Craft JC, Shin CS, Fleckenstein L, Borghini-Fuhrer I, Rim HJ, 2012. Review of pyronaridine anti-malarial properties and product characteristics. Malar J 11: 270.
31. World Health Organisation, 2019. Information note: The use of artesunate-pyronaridine for the treatment of uncomplicated malaria, October 2019. Geneva: World Health Organisation,.
32. Ministry of Health Kenya, 2020. Interim guidelines on management of COVID-19 in Kenya. COVID-19, Infection Prevention and Control (IPC) and Case Management. Nairobi.
33. Welle D, 2020. COVID-19: Tests for 'miracle cure' herb Artemisia begin. Available at: https://www.dw.com/en/covid-19-tests-for-miracle-cure-herb-artemisia-begin/a-53442366. Accessed July 05, 2020.
34. Tu YP, Jennings R, Hart B, Cangelosi GA, Wood RC, Wehber K, Verma P, Vojta D, Berke EM, 2020. Swabs Collected by Patients or Health Care Workers for SARS-CoV-2 Testing. N Engl J Med.
35. Diaz MH, Waller JL, Theodore MJ, Patel N, Wolff BJ, Benitez AJ, Morris T, Raghunathan PL, Breiman RF, Whitney CG, Blau DM, Winchell JM, 2019. Development and Implementation of Multiplex TaqMan Array Cards for Specimen Testing at Child Health and Mortality Prevention Surveillance Site Laboratories. Clin Infect Dis 69: S311-S321.
36. Han A, Poon JL, Powers JH, 3rd, Leidy NK, Yu R, Memoli MJ, 2018. Using the Influenza Patient-reported Outcome (FLU-PRO) diary to evaluate symptoms of influenza viral infection in a healthy human challenge model. BMC Infect Dis 18: 353.
37. Powers JH, 3rd, Bacci ED, Leidy NK, Poon JL, Stringer S, Memoli MJ, Han A, Fairchok MP, Coles C, Owens J, Chen WJ, Arnold JC, Danaher PJ, Lalani T, Burgess TH, Millar EV, Ridore M, Hernandez A, Rodriguez-Zulueta P, Ortega-Gallegos H, Galindo-Fraga A, Ruiz-Palacios GM, Pett S, Fischer W, Gillor D, Moreno Macias L, DuVal A, Rothman R, Dugas A, Guerrero ML, 2018. Performance of the inFLUenza Patient-Reported Outcome (FLU-PRO) diary in patients with influenza-like illness (ILI). PLoS One 13: e0194180.
38. Powers JH, 3rd, Bacci ED, Guerrero ML, Leidy NK, Stringer S, Kim K, Memoli MJ, Han A, Fairchok MP, Chen WJ, Arnold JC, Danaher PJ, Lalani T, Ridore M, Burgess TH, Millar EV, Hernandez A, Rodriguez-Zulueta P, Smolskis MC, Ortega-Gallegos H, Pett S, Fischer W, Gillor D, Macias LM, DuVal A, Rothman R, Dugas A, Ruiz-Palacios GM, 2018. Reliability, Validity, and Responsiveness of InFLUenza Patient-Reported Outcome (FLU-PRO(c)) Scores in Influenza-Positive Patients. Value Health 21: 210-218.

39. Powers JH, Guerrero ML, Leidy NK, Fairchok MP, Rosenberg A, Hernandez A, Stringer S, Schofield C, Rodriguez-Zulueta P, Kim K, Danaher PJ, Ortega-Gallegos H, Bacci ED, Stepp N, Galindo-Fraga A, St Clair K, Rajnik M, McDonough EA, Ridore M, Arnold JC, Millar EV, Ruiz-Palacios GM, 2016. Development of the Flu-PRO: a patient-reported outcome (PRO) instrument to evaluate symptoms of influenza. BMC Infect Dis 16: 1.
40. World Health Organization., 2009. Methods for surveillance of antimalarial drug efficacy. Geneva: World Health Organization.
41. World Health Organisation, 2008. Methods and techniques for clinical trials on antimalarial drug efficacy: genotyping to identify parasite populations. Informal consultation organized by the Medicines for Malaria Venture and cosponsored by the World Health Organization. 29–31 May 2007, Amsterdam, The Netherlands. Organisation WH, ed.
42. Cao Y, Li L, Feng Z, Wan S, Huang P, Sun X, Wen F, Huang X, Ning G, Wang W, 2020. Comparative genetic analysis of the novel coronavirus (2019-nCoV/SARS-CoV-2) receptor ACE2 in different populations. Cell Discov 6: 11.
43. Research NIfH, 2014. CT-toolokit. Available at: http://www.ct-toolkit.ac.uk/glossary/suspected-unexpected-serious-adverse-reactions-susar. Accessed 02 July 2016, 2016.
44. Cai Q, Yang M, Liu D, Chen J, Shu D, Xia J, Liao X, Gu Y, Cai Q, Yang Y, Shen C, Li X, Peng L, Huang D, Zhang J, Zhang S, Wang F, Liu J, Chen L, Chen S, Wang Z, Zhang Z, Cao R, Zhong W, Liu Y, Liu L, 2020. Experimental Treatment with Favipiravir for COVID-19: An Open-Label Control Study. Engineering (Beijing).
45. Lou B, Li TD, Zheng SF, Su YY, Li ZY, Liu W, Yu F, Ge SX, Zou QD, Yuan Q, Lin S, Hong CM, Yao XY, Zhang XJ, Wu DH, Zhou GL, Hou WH, Li TT, Zhang YL, Zhang SY, Fan J, Zhang J, Xia NS, Chen Y, 2020. Serology characteristics of SARS-CoV-2 infection since exposure and post symptom onset. Eur Respir J.
46. Beigel JH, Tomashek KM, Dodd LE, Mehta AK, Zingman BS, Kalil AC, Hohmann E, Chu HY, Luetkemeyer A, Kline S, Lopez de Castilla D, Finberg RW, Dierberg K, Tapson V, Hsieh L, Patterson TF, Paredes R, Sweeney DA, Short WR, Touloumi G, Lye DC, Ohmagari N, Oh MD, Ruiz-Palacios GM, Benfield T, Fatkenheuer G, Kortepeter MG, Atmar RL, Creech CB, Lundgren J, Babiker AG, Pett S, Neaton JD, Burgess TH, Bonnett T, Green M, Makowski M, Osinusi A, Nayak S, Lane HC, Members A-SG, 2020. Remdesivir for the Treatment of Covid-19 - Preliminary Report. N Engl J Med.
47. Brent A, Berkley J, Chilengi R, KEMRI-Wellcome Trust, 2008. KEMRI CGMR-C: Guidelines for the conduct of Clinical Research. Prepared for the Clinical Research Group & Scientific Coordinating Committee.
48. Peplow C, Assfalg R, Beyerlein A, Hasford J, Bonifacio E, Ziegler AG, 2019. Blood draws up to 3% of blood volume in clinical trials are safe in children. Acta Paediatr 108: 940-944.
49. Howie SR, 2011. Blood sample volumes in child health research: review of safe limits. Bull World Health Organ 89: 46-53.
50. de Onis M, Onyango AW, Borghi E, Siyam A, Nishida C, Siekmann J, 2007. Development of a WHO growth reference for school-aged children and adolescents. Bull World Health Organ 85: 660-7.
51. Centers for Disease Control and Prevention (CDC), 2000. Weight-for-age Growth Chart 2 to 20 years: Girls Stature Weight-for-age percentiles. Developed by the National Center for Health Statistics in collaboration with the National Center for Chronic Disease Prevention and Health Promotion (2000). http://www.cdc.gov/growthcharts. Available at: https://www.cdc.gov/growthcharts/clinical_charts.htm. Accessed.
52. World Health Organisation, 2020. Rational use of personal protective equipment for coronavirus disease 2019 (COVID-19). Interim guidance, 27 February 2020. Geneva: World Health Organisation.
53. 2010. Uniform requirements for manuscripts submitted to biomedical journals: Writing and editing for biomedical publication. Journal of Pharmacology & Pharmacotherapeutics 1: 42-58.
54. Wu L, Hall T, Ssewanyana I, Oulton T, Patterson C, Vasileva H, Singh S, Affara M, Mwesigwa J, Correa S, Bah M, D'Alessandro U, Sepulveda N, Drakeley C, Tetteh KKA, 2019. Optimisation and standardisation of a multiplex immunoassay of diverse Plasmodium falciparum antigens to assess changes in malaria transmission using sero-epidemiology. Wellcome Open Res 4: 26.
55. Wang W, Su B, Pang L, Qiao L, Feng Y, Ouyang Y, Guo X, Shi H, Wei F, Su X, Yin J, Jin R, Chen D, 2020. High-dimensional immune profiling by mass cytometry revealed immunosuppression and dysfunction of immunity in COVID-19 patients. Cell Mol Immunol 17: 650-652.
56. Sundling C, Rönnberg C, Yman V, Asghar M, Jahnmatz P, Lakshmikanth T, Chen Y, Mikes J, Forsell MN, Sondén K, Achour A, Brodin P, Persson KE, Färnert A, 2019. B cell profiling in malaria reveals expansion and remodelling of CD11c+ B cell subsets. JCI insight 5: e126492.

57. van de Veerdonk FL, Janssen NAF, Grondman I, de Nooijer AH, Koeken VACM, Matzaraki V, Boahen CK, Kumar V, Kox M, Koenen HJPM, Smeets RL, Joosten I, Br, amp, uumlggemann RJM, Kouijzer IJE, van der Hoeven HG, Schouten JA, Frenzel T, Reijers M, Hoefsloot W, Dofferhoff ASM, Kerckhoffs A, amp, P.M. e, Blaauw MJT, Veerman K, Maas C, Schoneveld AH, Hoefer IE, Derde LPG, Willems L, Toonen E, van Deuren M, van der Meer JWM, van Crevel R, Giamarellos-Bourboulis EJ, Joosten LAB, van den Heuvel MM, Hoogerwerf J, de Mast Q, Pickkers P, Netea MG, 2020. A systems approach to inflammation identifies therapeutic targets in SARS-CoV-2 infection. medRxiv: 2020.05.23.20110916.

16 APPENDICES

16.1 APPENDIX I. ROLE OF INVESTIGATORS AND COLLABORATORS

16.1.1 Definition investigator and collaborator

16.1.1.1 Investigators

Investigators are directly engaged with the field research and live and work in Kenya and Burkina Faso, except for the trial statisticians and Chief Investigators.

16.1.1.2 Collaborators

Collaborators are not directly engaged with any of the fieldwork on-site and have an advisory and supporting role either as PhD supervisors living overseas or support the study with the laboratory assays in their overseas laboratories. Each home institutions will sign MOUs in the form of MTA and/or DTAs with the overseas collaborators before samples or data can be shipped overseas.

16.1.2 Protocol development: authors' contributions

The Chief Investigators wrote the first draft of the protocol. All investigators contributed to the refinement of the study protocol and approved the final versions.

16.1.3 Role Investigators

16.1.3.1 Chief Investigators

Prof Feiko ter Kuile (MD, PhD), is a Professor of Tropical Epidemiology from the Liverpool School of Tropical Medicine (LSTM) based at KEMRI in Kisumu. He is the Chief Investigator and grant holder of the study component in Kenya and will carry overall responsibility for the coordination of the trial and for the linkages with the sponsor, funders and with international partners involved.

Prof Chris Drakeley (PhD), is a Professor of Infection & Immunity at the London School of Hygiene & Tropical Medicine with over 30 years' experience of working on malaria. He is the Chief Investigator and grant holder of the study component in Burkina Faso and will carry overall responsibility for the coordination of the immunological studies and for the linkages with the sponsor, funders and with international partners involved.

16.1.3.2 Co-principal investigators

Dr Hellen Barsosio (MD, MSc), is a research medical officer with KEMRI/CDC/LSTM collaboration, based in Kisumu, Kenya. She will be the co-Principal Investigator for Kenya. She will also liaise with the local hospitals, the ethics committees, the regulators and the MoH in the country. She will be supervised locally by Dr Simon Kariuki, the KEMRI-CDC Malaria Branch Chief and internationally by Prof Feiko ter Kuile.

Dr Simon Kariuki (PhD), is the KEMRI/CDC Malaria Branch Chief and will be the co-Principal Investigator for Kenya. Dr Kariuki, the KEMRI-CDC Malaria Branch Chief and oversees all research studies that fall under the Malaria Branch. He will supervise Dr Hellen Barsosio in addition to providing local support with regulators and the MoH in the country.

Prof Sodiomon B. Sirima (MD, PhD), is the Principal Investigator in Burkina Faso and heads the Groupe de Recherche Action en Santé (GRAS). He will have the overall responsibility for the study in

Burkina Faso and liaise with the local hospitals, the ethics committees, the regulators and the MoH in the country.

16.1.3.3 Co-investigators

Dr Alfred B Tiono, (MD, PhD), is part of the Groupe de Recherche Action en Santé (GRAS). Together with Prof Sodiomon B. Sirima, he will have the overall responsibility for the study in Burkina Faso and liaise with the local hospitals, the ethics committees, the regulators and the MoH in the country.

Dr Issa Ouedraogo, MD, MSc from GRAS in Burkina Faso is a biologist. He will oversee all the laboratory activities related to immunological sample collection and processing in Burkina Faso.

Dr Issiaka Soulama, PharmD, MSc and PhD from GRAS in Burkina Faso is a molecular biologist. He will conduct the study activities in relations with molecular processing of samples in Burkina Faso.

Dr Titus Kwambai, MD, PhD, from the Centre of Global Health Research (CGHR), KEMRI and CDC in western Kenya will support the clinical and epidemiological aspects of the study and act as the clinical liaison with Siaya County in western Kenya.

Mr Kephas Otieno, BSc from the Centre of Global Health Research (CGHR), KEMRI, Kisumu western Kenya heads the malaria laboratories and will oversee the laboratory activities related to the research samples in Kenya.

Dr Victor Okelo, MD, MPH, MBA, from KEMRI-CGHR and CDC in western Kenya will coordinate the collaboration between KEMRI, CDC and LSTM on-site in western Kenya and act as the clinical liaison with the Kisumu County health authorities.

Dr Clayton Onyango, PhD, from KEMRI-CGHR and CDC in western Kenya, will coordinate the viral testing for SARS-CoV-2 and act as the testing liaison between KEMRI, CDC, LSTM and the County health authorities in western Kenya.

Dr Aaron Samuels (MD, MHS) is a medical epidemiologist from the Centers for Disease Control and Prevention (CDC) and an adult physician and infectious disease specialist. He is the Malaria Program Director and Senior Technical Advisor, CDC-Kenya, based at KEMRI-CGHR in western Kenya. In Kenya, he represents the Malaria Branch of the Division of Parasitic Diseases and Malaria, Center for Global Health, CDC. He has over ten years' experience in clinical research and will provide scientific co-leadership for the study site in Kenya and act as a liaison between KEMRI, LSTM and with CDC's malaria and COVID-19 related activities in the country.

Prof Penelope Phillips-Howard (PhD) is a public health epidemiologist with 30 years' research experience who will provide support with protocol writing and conduct of the study in western Kenya.

Prof Duolao Wang, PhD is Professor of Medical Statistics at the Liverpool School of Tropical Medicine and will be the statistician of the nested malaria treatment trial.

James Dodd, MSc at the Liverpool School of Tropical Medicine is a statistician who will be involved with the data merging and data analysis under supervision of Prof Duolao Wang.

16.2 APPENDIX II. TERMS OF REFERENCE STUDY MANAGEMENT GROUP AND DSMB

The study will have the following committees and governance structure. All committees will act in accordance with the International Clinical Harmonization Guidelines for Good Clinical Practice (GCP) Principles.

16.2.1 Study Management Group (TMG)

16.2.1.1 Membership

The Chief Investigators (2x, ad hoc) and Country Co-Principal Investigators (4x) or representatives, the country study coordinator(s), lead data manager, lead administrators. Others involved in the day to day running of the trial are invited ad hoc.

16.2.1.2 Responsibilities

The TMG is responsible for the administrative management and day to day running of the study. Their activities will include study planning, organising TMG and DSMB meetings, providing reports to the DSMB, regulators, and ethics committees, SUSAR [Serious unexpected suspected adverse events] reporting, maintaining the study/trial master file, budget administration and contractual issues, the organisation of central data management and sample collection. The TMG will also monitor the conduct of the study by reviewing monthly reports prepared by the data managers and statisticians of accrual and baseline characteristics, data completeness, specimen collection, and adverse events (AEs). The study team will also review individual participant-level safety data frequently to assess the relation of all reported AEs to study treatment.

16.2.2 Data Safety and Monitoring Board (DSMB)

16.2.3 Membership

An independent data and safety monitoring board (DSMB) will be convened for this study. The DSMB will consist of four independent members with expertise in coronavirus disease (COVID-19) or respiratory viruses, antiviral therapies and shedding, malaria, and a biostatistician. If so required, in addition to the core members, a topic expert can be invited by the chair of the DSMB on an ad hoc basis to attend. An unblinded biostatistician may also be invited to attend part of the closed sessions to present the most current data from the trial or provide the allocation code for certain participants.

16.2.3.1 Operational

The DSMB meetings will be conducted via teleconference and consist of an open session, followed by a closed session. The open sessions can be attended by representatives of the investigators, including the Chief Investigators, Country Principal Investigators, and study statistician. Other investigators can also attend open session on an ad hoc basis. The funder can attend open DSMB sessions as observers. The Chair and at least one of the three other independent members together will constitute a quorum.

The DSMB will have a start-up meeting to discuss its mandate and discuss the protocol. If possible, a second meeting will take place shortly after the commencement of the study, e.g. when approximately 10% of participants have been recruited. After that, the frequency of the meetings will be determined by the Chair of the DSMB in consultation with the Chief Investigators. It is anticipated that the DSMB will generally meet twice per year during the life span of the study and have one meeting at the closure of the study.

During the first meeting, the DSMB members will review the timeline set out in the protocol for participant recruitment, informed consent documents and plans for data safety monitoring. Prior to subsequent meetings, open reports containing accrual and retention rates, participant characteristics, and serious adverse events will be sent to the DSMB members the week prior to any DSMB meeting. Only the DSMB members and the unblinded biostatistician will receive password-

protected closed reports of SARS-CoV-2 endpoints by randomisation arm. A full confidential report should be submitted in writing to the Chief Investigator at the end of each DSMB meeting

16.2.3.2 Role of the DSMB

They are independent and look at the study overall and the nested malaria treatment trial from an ethical point of view of the participant safety, future participants, and society in general. It is their responsibility to prevent participants from being exposed to any excess risks by making a recommendation to the Investigators for the study or trial suspension or early termination if the safety or efficacy results are sufficiently convincing. For the nested trial component, the DSMB can choose whether to review the data blinded or to see unblinded data and analyses by study arm. A DSMB charter will be developed for this study that will supersede this section of the protocol. Any changes in the role of the DSMB relative to this protocol text will not be considered a protocol deviation and does require an amendment. Briefly, some of the suggested roles of the DSMB will include:

- To evaluate the progress of the study/trial in relation to the timeliness, data quality and other factors that can affect the overall objectives of the trial
- To ensure participant rights and safety are adhered to and that the protocol demands freely given informed consent
- To conduct any interim reviews if specified) when adequate data have been accrued of blinded or unblinded safety data and report back to the Chief Investigators with a recommendation whether the study or nested trial should continue, the protocol be modified, or the trial or trial arm be stopped.
- Review and any major deviations from the study protocol
- To review relevant information from other sources
- To review and approve modifications to the overall enrolment target based on the event rate.
- To inform the Chief Investigators and TMG of the need to make changes to the trial protocol
- To consider the blinded or unblinded interim data from the study and nested trial and relevant information from other sources.
- To determine how frequently any interim analysis of study or trial data should be undertaken.
- To consider any requests for unblinding and release of interim trial data and to recommend on the importance of this.
- To ensure that the study results are disseminated appropriately, and consideration be given to the implementation of the results into policy

16.3 Appendix III. Declaration of Helsinki

WORLD MEDICAL ASSOCIATION DECLARATION OF HELSINKI

Recommendations guiding physicians in biomedical research involving human subjects. Adopted by the 18th World Medical Assembly, Helsinki, Finland, June 1964. Amended by the 29th World Medical Assembly, Tokyo, Japan, October 1975, 35th World Medical Assembly, Venice, Italy, October 1983, 41st World Medical Assembly Hong Kong, September 1989 and the 48th General Assembly, Somerset West, Republic of South Africa, October 1996

INTRODUCTION

It is the mission of the physician to safeguard the health of the people. His or her knowledge and conscience are dedicated to the fulfilment of this mission.

The Declaration of Geneva of the World Medical Association binds the physician with the words, "The health of my patient will be my first consideration", and the International Code of Medical Ethics declares that, "A physician shall act only in the patient's interest when providing medical care which might have the effect of weakening the physical and mental condition of the patient."

The purpose of biomedical research involving human subjects must be to improve diagnostic, therapeutic and prophylactic procedures and the understanding of the aetiology and pathogenesis of disease.

In current medical practice, most diagnostic, therapeutic or prophylactic procedures involve hazards. This applies especially to biomedical research.

Medical progress is based on research which ultimately must rest in part on experimentation involving human subjects.

In the field of biomedical research, a fundamental distinction must be recognized between medical research in which the aim is essentially diagnostic or therapeutic for a patient, and medical research, the essential object of which is purely scientific and without implying direct diagnostic or therapeutic value to the person subjected to the research.

Special caution must be exercised in the conduct of research which may affect the environment, and the welfare of animals used for research must be respected.

Because it is essential that the results of laboratory experiments be applied to human beings to further scientific knowledge and to help suffering humanity, the World Medical Association has prepared the following recommendations as a guide to every physician in biomedical research involving human subjects. They should be kept under review in the future. It must be stressed that the standards as drafted are only a guide to physicians all over the world. Physicians are not relieved from criminal, civil and ethical responsibilities under the laws of their own countries.

Basic principles

- Biomedical research involving human subjects must conform to generally accepted scientific principles and should be based on adequately performed laboratory and animal experimentation and on a thorough knowledge of the scientific literature.
- The design and performance of each experimental procedure involving human subjects should be clearly formulated in an experimental protocol which should be transmitted for consideration, comment and guidance to a specially appointed committee independent of the investigator and

the sponsor provided that this independent committee is in conformity with the laws and regulations of the country in which the research experiment is performed.

- Biomedical research involving human subjects should be conducted only by scientifically qualified persons and under the supervision of a clinically competent medical person. The responsibility for the human subject must always rest with a medically qualified person and never rest on the subject of the research, even though the subject has given his or her consent.
- Biomedical research involving human subjects cannot legitimately be carried out unless the importance of the objective is in proportion to the inherent risk to the subject.
- Every biomedical research project involving human subjects should be preceded with careful assessment of predictable risks in comparison with foreseeable benefits to the subject or to others. Concern for the interests of the subject must always prevail over the interests of science and society.
- The right of the research subject to safeguard his or her integrity must always be respected. Every precaution should be taken to respect the privacy of the subject and to minimize the impact of the study on the subject's physical and mental integrity and on the personality of the subject.
- Physicians should abstain from engaging in research projects involving human subjects unless they are satisfied that the hazards involved are believed to be predictable. Physicians should cease any investigation if the hazards are found to outweigh the potential benefits.
- In publication of the results of his or her research, the physician is obliged to preserve the accuracy of the results. Reports of experimentation not in accordance with the principles laid down in this Declaration should not be accepted for publication.
- In any research on human beings, each potential subject must be adequately informed of the aims, methods, anticipated benefits and potential hazards of the study and the discomfort it may entail. He or she should be informed that he or she is at liberty to abstain from participation in the study and that he or she is free to withdraw his or her consent to participation at any time. The physician should then obtain the subject's freely-given informed consent, preferably in writing.
- When obtaining informed consent for the research project, the physician should be particularly cautious if the subject is in a dependent relationship to him or her or may consent under duress. In that case the informed consent should be obtained by a physician who is not engaged in the investigation and who is completely independent of this official relationship.
- In case of legal incompetence, informed consent should be obtained from the legal guardian in accordance with national legislation. Where physical or mental incapacity makes it impossible to obtain informed consent, or when the subject is a minor, permission from the responsible relative replaces that of the subject in accordance with national legislation. Whenever the minor child is in fact able to give a consent, the minor's consent must be obtained in addition to the consent of the minor's legal guardian.
- The research protocol should always contain a statement of the ethical considerations involved and should indicate that the principles enunciated in the present Declaration are complied with.

Medical research combined with professional care (Clinical Research)

- In the treatment of the sick person, the physician must be free to use a new diagnostic and therapeutic measure, if in his or her judgement it offers hope of saving life, re-establishing health or alleviating suffering.
- The potential benefits, hazards and discomfort of a new method should be weighed against the advantages of the best current diagnostic and therapeutic methods.

- In any medical study, every patient - including those of a control group, if any - should be assured of the best proven diagnostic and therapeutic method. This does not exclude the use of inert placebo in studies where no proven diagnostic or therapeutic method exists.
- The refusal of the patient to participate in a study must never interfere with the physician-patient relationship.
- If the physician considers it essential not to obtain informed consent, the specific reasons for this proposal should be stated in the experimental protocol for transmission to the independent committee (I,2).
- The physician can combine medical research with professional care, the objective being the acquisition of new medical knowledge, only to the extent that medical research is justified by its potential diagnostic or therapeutic value for the patient.

Non-therapeutic biomedical research involving human subjects (Non-clinical biomedical research)

- In the purely scientific application of medical research carried out on a human being, it is the duty of the physician to remain the protector of the life and health of that person on whom biomedical research is being carried out.
- The subjects should be volunteers -- either healthy persons or patients for whom the experimental design is not related to the patient's illness.
- The investigator or the investigating team should discontinue the research if in his/her or their judgement it may, if continued, be harmful to the individual.
- In research on man, the interest of science and society should never take precedence over considerations related to the well-being of the subject.

16.4 Appendix IV. WHO clinical progression scale

Figure 6: WHO clinical progression scale

Patient State	Descriptor	Score
Uninfected	Uninfected; no viral RNA detected	0
Ambulatory mild disease	Asymptomatic; viral RNA detected	1
	Symptomatic; independent	2
	Symptomatic; assistance needed	3
Hospitalised: moderate disease	Hospitalised; no oxygen therapy*	4
	Hospitalised; oxygen by mask or nasal prongs	5
Hospitalised: severe diseases	Hospitalised; oxygen by NIV or high flow	6
	Intubation and mechanical ventilation, pO_2/FiO_2 ≥150 or SpO_2/FiO_2 ≥200	7
	Mechanical ventilation pO_2/FIO_2 <150 (SpO_2/FiO_2 <200) or vasopressors	8
	Mechanical ventilation pO_2/FiO_2 <150 and vasopressors, dialysis, or ECMO	9
Dead	Dead	10

ECMO=extracorporeal membrane oxygenation. Fi02=fraction of inspired oxygen. NIV=non-invasive ventilation. p02=partial pressure of oxygen. Sp02=oxygen saturation. *If hospitalised for isolation only, record status as for ambulatory patient.

16.5 APPENDIX V. MODIFIED FLU-PRO PLUS SCALE

Flu-PRO scale
DAILY SURVEY (D1-14, D21, D28)
inFLUenza patient-reported outcome (Flu-PRO): 32 items validated, can score across domains. The FLU-PRO plus version differs from the below FLU-PRO version in that it says “viral respiratory infections” instead of “Flu” and has the two additional questions (loss of taste/loss of smell).

***FYI: This takes about 5 minutes to complete

How are you feeling today?

FLU-PRO	Not at all	A little bit	Somewhat	Quite a bit	Very much
Nose					
Runny or dripping					
Congestion or stuffy					
Sneezing					
Sinus pressure					
Lack of smell					
Throat					
Sore throat					
Scratchy or itchy throat					
Difficulty swallowing					
Lack of taste					
Eyes					
Teary or watery eyes					
Sore or painful eyes					
Eyes sensitive to light					
Chest/Respiratory					
Trouble breathing					
Chest congestion					
Chest tightness					
Dry or hacking cough					
Wet or loose cough					
Sputum (coughing up sputum or phlegm)					
Wheezing					
Gastrointestinal					
Felt nauseous					
Stomach ache					
Vomit					
Diarrhea					
Body/Systemic					
Felt Dizzy					
Head congestion					
Headache					
Lack of appetite					
Sleeping more than usual					
Body aches or pains					
Weak or tired					
Chills of shivering					
Felt cold					
Felt hot					

Sweating

How are you feeling today?

0 no symptoms	1 Mild	2 Moderate	3 Severe	4 Very severe

Please rate interference in daily activities due to illness:

1 Not at all	2 A little bit	3 Somewhat	4 Quite a bit	5 Very much

How is your general health?

1 Poor	2 Fair	3 Good	4 Very good	5 Excellent

Have you returned to your usual health today? Yes/no
Have you returned to your usual activities today? Yes/no

TAKE MEDS, TAKE SWAB (Copy post-exposure prophylaxis)
Take your vitals:
Oxygen level
Pulse
Temperature
Respiratory rate
Electrocardiogram monitor

PM Time:
Take meds
Take vitals
Oxygen level
Pulse
Temperature
Respiratory rate

Anxious mood	Not at all	mild	moderate	severe	very severe

Yu J, Powers JH, Vallo D, et al. Evaluation of efficacy outcomes for a Phase IIb study of a respiratory syncytial virus vaccine in older adults using patient-reported outcomes with laboratory confirmation. Value Health. 2020 Feb;23(2):227-235.

16.6 Appendix VI. Personal protection equipment guidelines for study staff by risk category

Risk category	Characteristic of exposure of staff	Examples of staff	Surgical masks	Gloves	Gown	Particulate respirators (e.g. N95 masks)	Eye protection (e.g. Goggles/ face shield)
1	Health care workers who manage patients clinically and have close contact (<1 meter) with known/suspected COVID-19 patients or their infectious material	E.g. Doctors, nurses who work in the fever clinics	√	√	√	√	√
2	Non-health care worker staff who have close contact (<1 meter) with known/suspected COVID-19 patients or their infectious material	E.g. Security personnel, receptionist, cleaning staff who work in the fever clinic	√	√	√		
3	Staff with close contact (<1 meter) with persons of "unknown" COVID-19 status	E.g., Essential duty travellers	√				
4	Staff infected with COVID-19 influenza	E.g. Patients in the fever clinic	√				
5	Staff with no known close contact (<1 meter) with known/ suspected COVID- 19 patients or their infectious material	E.g. Critical staff "quarantined" in a workspace, and not working in the fever clinic					

16.7 Appendix VII. Product characteristics

16.7.1 Pyronaridine-artesunate

See next pages

ANNEX I

SUMMARY OF PRODUCT CHARACTERISTICS

1. NAME OF THE MEDICINAL PRODUCT

Pyramax 180 mg/60 mg Film-coated tablet

2. QUALITATIVE AND QUANTITATIVE COMPOSITION

Each Pyramax tablet contains 180 mg Pyronaridine tetraphosphate and 60 mg Artesunate.

Excipients with known effect: each tablet contains 0.11 mg Sunset yellow FCF (E110) and 0.58 mg Tartrazine (E102).
For a full list of excipients, see section 6.1.

3. PHARMACEUTICAL FORM

Film-coated tablet
Round, biconvex, orange coloured tablet

4. CLINICAL PARTICULARS

4.1 Therapeutic indications

Pyramax tablets are indicated in the treatment of acute, uncomplicated malaria infection caused by *Plasmodium falciparum* or by *Plasmodium vivax* in adults and children weighing 20 kg or more.

Consideration should be given to official guidance on the appropriate use of antimalarial agents (see section 4.4)

4.2 Posology and method of administration

Mode of administration
The dose should be taken orally once a day for three days with or without food.

<u>*Posology*</u>

Dosage in adults and children
Pyramax tablets should be taken orally as a single daily dose for three consecutive days.

Body weight	Number of tablets	Regimen
20 - < 24 kg	1 tablet	Daily for 3 days
24 - <45 kg	2 tablets	Daily for 3 days
45 - < 65 kg	3 tablets	Daily for 3 days
≥ 65 kg	4 tablets	Daily for 3 days

A granule formulation is available for children weighing between 5 kg to under 20 kg.

In the event of vomiting within 30 minutes of administration after the first dose, a repeat dose should be given. If the repeat dose is vomited, the patient should be given an alternative antimalarial drug. In the event of non-severe diarrhoea normal dosing should be continued.

If a dose is missed, it should be taken as soon as realised and then the recommended regimen continued until the full course of treatment has been completed.

Dosage in paediatrics population
Pyramax is dosed according to body weight. The safety and efficacy of Pyramax tablets has not been established in children below 20 kg body weight. The clinical studies conducted in *Plasmodium vivax* malaria, included only 13 patients below 12 years old (see section 5.1.)

Elderly
Clinical studies did not include patients aged 65 years and over. No dosing adjustments are necessary based on present knowledge and the short 3 day course of treatment. However, considering the possibility of age-associated decrease in hepatic and renal function, caution should be exercised when administering the product to the elderly.

Dosage in hepatic and renal impairment

There is no information on dosing in patients with hepatic impairment. Due to its potential liver toxicity Pyramax is contraindicated in patients with signs of hepatic impairment or known significant liver function test abnormalities.

There is no information on dosing patients with severe renal impairment. Although excretion via faeces was the main route of elimination of pyronaridine-related material in a human mass balance study, significant urinary excretion was also observed. Pyramax is, therefore, contraindicated in the case of severe renal impairment and caution should be exercised when treating patients with mild or moderate renal impairment.

4.3 Contraindications

- Known hypersensitivity to pyronaridine or artesunate or any component of the formulation.

- Patients with clinical signs or symptoms of hepatic injury (such as nausea and/or abdominal pain associated with jaundice) or known severe liver disease (i.e. decompensated cirrhosis, Child-Pugh stage B or C).

- Severe renal impairment

4.4 Special warnings and precautions for use

Pyramax tablets should not be used as a prophylactic treatment of malaria.

Pyramax has been associated, in some patients, with transient increases in liver enzymes without clinical signs (see section 4.8). Pyramax is contra-indicated in the case of underlying hepatic injury, clinical signs or symptoms of hepatic injury or known severe liver disease (see section 4.3). If a patient is already known to have elevated transaminases the use of Pyramax is not recommended.

Patients should be advised of the clinical signs and symptoms of hepatotoxicity in order to monitor closely if such signs or symptoms occur, especially in the first two weeks after Pyramax intake. It is recommended that, in patients who exhibit symptoms of hepatotoxicity following treatment with Pyramax, the liver function tests be monitored if possible, until normalisation.

No data are available in patients with co-infections (HBV, HCV, HIV); those receiving co-administration of drugs known to be associated with mitochondrial toxicity (i.e. valproate, antiretroviral drugs), use of herbal medicines, patients with malnutrition or patients with other hepatic underlying conditions (i.e. ethanol intoxication, hepatic steatosis). Particular caution is advised in these patients regarding the risk of liver toxicity since these risk factors, also including co-administration of paracetamol, might produce a cumulative effect on the liver. Enhanced surveillance is warranted in young children in case of malnutrition.

No specific QT/QTc study has been performed to specifically assess the cardiac safety of Pyramax. Based on the available comparative clinical studies, this risk does not appear to be higher with single or repeat administration of Pyramax as compared to the other available antimalarial drugs used in these trials (artesunate + mefloquine, chloroquine, artemether-lumefantrine). However, patients with known history or evidence of clinically significant cardiovascular disorders (including arrhythmia, QTc interval ≥450 milliseconds) were excluded from these clinical studies. Therefore, caution should be exercised in at risk patients i.e. those:

- with congenital prolongation of QTc interval, hypokalaemia, dehydration, cardiac arrhythmia, heart failure, etc.
- treated concomitantly with other drugs that can block potassium channels, such as antiarrhythmics, neuroleptics, certain antimicrobial agents (e.g. macrolides, fluoroquinolones, imidazole and triazole antifungals, pentamidine, saquinavir) and non-sedating antihistamines, cisapride, domperidone or methadone,
- recently treated with medicinal products with long elimination half-life and known to prolong the QTc interval that may still be circulating at the time Pyramax treatment course is commenced (see section 4.8. and 5.1.).

A fall in haemoglobin may occur during treatment. There is very little information on the effect of this in patients with initial haemoglobin levels of less than 8 g/dl. Caution should be exercised in treating patients with a low haemoglobin.

Pyramax should not be used for the treatment of severe malaria, cerebral malaria or other severe manifestations of complicated malaria, including hyperparasitaemia, pulmonary oedema, severe anaemia, renal or hepatic failure. Patients with severe malaria are not candidates for oral therapy.

In patients with acute malaria who present with severe diarrhoea and vomiting, alternative therapy should be considered. If Pyramax is used in these patients, the parasite load should be closely monitored.

Pyramax is a blood schizonticide and for the treatment of *P. vivax* malaria, a radical cure (to destroy the parasite in the liver and thus prevent relapse) is required with a hypnozoitocidal drug such as primaquine.

In the event of proven or suspected recrudescent malaria infections after treatment with Pyramax, patients should be treated with a different blood schizonticide.

Artemisinin compounds should not be used for treatment of malaria in the first trimester of pregnancy if other suitable and effective antimalarials are available (See Section 4.6).

There is no experience in the treatment of mixed *P. vivax* and *P. falciparum* infections. No data are available with Pyramax in the treatment of malaria due to *Plasmodium malariae* or *Plasmodium ovale*.

The safety and effectiveness of Pyramax for the treatment of malaria in patients with HIV/AIDS has not been established. If Pyramax is used in these patients, the parasite load should be closely monitored.

This medicine contains tartrazine (E102) and sunset yellow (E110) as colouring agents which may cause allergic reactions which may manifest as flushing, the appearance of wheals/urticarial, breathlessness, faintness and/or fall in blood pressure.

4.5 Interaction with other medicinal products and other forms of interaction

Particular caution is advised in case of co-administration of drugs known to be associated with mitochondrial toxicity (i.e. valproate, antiretroviral drugs), use of herbal medicines, and also co-administration of paracetamol (see section 4.4).

Pyronaridine shows *in vitro* CYP2D6 inhibitory potential that is confirmed *in vivo* using metoprolol as CYP2D6 probe. The study shows an increase of metoprolol Cmax around 50% but the overall exposure increases to a lesser extent. Caution is therefore advised when co-administering Pyramax with metoprolol given in cardiac failure, notably during the titration phase, and a possible dose adjustment may be required. This also applies to flecainide and propafenone, two antiarrhythmics exclusively metabolised by CYP2D6.

As pyronaridine shows *in vitro* P-gp inhibitory potential, substrates for P-gp such as digoxin and dabigatran may also require additional monitoring of blood levels and possible dose adjustment.

The combination of Pyramax and primaquine has shown neither clinically relevant pharmacokinetic variations nor any impaired tolerance. If needed, the two antimalarial drugs may be co-administered.

Dihydroartemisinin (DHA) administration may result in a slight decrease in CYP1A2 activity. Caution is therefore, advised when Pyramax is administered concomitantly with medicinal products metabolised by this enzyme that have a narrow therapeutic index, such as theophylline. Any effects are unlikely to persist beyond 24 hours after the last intake of DHA.

Enzyme inducing medicinal products such as rifampicin, carbamazepine, phenytoin, phenobarbital, St. John's wort (Hypericum perforatum) may lead to reduced DHA plasma concentrations.

4.6 Fertility, pregnancy and lactation

Fertility
In animal studies, no effects on fertility and reproductive performance were observed. In these studies, the exposure to artesunate was below the human exposure; the maximum exposure to pyronaridine was 3-fold higher than the proposed human exposure.

Pregnancy
The safety of pyronaridine tetraphosphate and artesunate when administered concurrently for use in human pregnancy has not been established and the potential risk is unknown.

A component of Pyramax is artesunate, a recognized *in vivo* embryotoxic and teratogenic compound in animal models, including primate (see section 5.3). There is a limited amount of data from the use of artesunate during the first trimester of pregnancy.

Pyronaridine did not show any teratogenic effects in animal studies. There are no data from the use of pyronaridine in pregnant women.

Artemisinin compounds cannot be recommended for treatment of malaria in the first trimester of pregnancy. However, they should not be withheld if treatment is considered lifesaving for the mother and other antimalarials are considered unsuitable. Because of the limited safety data, artemisinin compounds should only be used in the second and third trimesters of pregnancy when other treatments are considered unsuitable (see sections 4.4 and 5.3).

Pregnancy register
A pregnancy register to monitor all pregnancies and their outcomes has been set up by the supplier. In the event that a patient is found to be pregnant whilst receiving Pyramax or becomes pregnant within two months of treatment this must be reported to the supplier immediately.

5

Lactation
Studies in rats have shown that pyronaridine is excreted into breast milk. The benefits of breastfeeding to mother and infant should be weighed against potential risk from infant exposure to pyronaridine through breast milk.

4.7 Effects on ability to drive and use machines

No studies on the effects on the ability to drive and use machines have been performed.

Dizziness, fatigue, asthenia and somnolence have been reported uncommonly or rarely following treatment with Pyramax. Patients should be warned not to drive or use machines if they feel tired or dizzy.

4.8 Undesirable effects

The safety of pyronaridine tetraphosphate and artesunate for treatment of malaria has been evaluated in clinical trials of more than 4000 patients.

Summary of the safety profile
The most commonly reported (≥1/100 to <1/10) adverse events were headache, eosinophilia, neutropenia, anaemia, increased platelet count, vomiting, abdominal pain, bradycardia, transaminase increases and hypoglycaemia.

Tabulated list of adverse reactions
The following table provides a summary of adverse reactions reported with Pyramax in clinical trial reports. Adverse reactions are ranked under headings of frequency using the MedDRA frequency convention: very common (≥1/10); common (≥1/100 to <1/10); uncommon (≥1/1000 to <1/100); rare (<1/1000).

System Organ Class	Common	Uncommon	Rare
**Blood and lymphatic system disorders**	Anaemia, eosinophilia, neutropenia, increased platelet count*	Basophilia, leukocytosis, leukopenia, lymphocytosis, monocytosis, splenomegaly, thrombocytopenia	Lymphopenia, pancytopenia
**Cardiac disorders**	Bradycardia	Palpitations, ventricular extrasystoles	Arrhythmia, atrioventricular block first degree, sinus arrhythmia
**Ear and labyrinth disorders**		Vertigo	Ear pain, hearing impaired, tinnitus
**Eye disorders**			Conjunctivitis
**Gastrointestinal disorders**	Abdominal pain, Vomiting	Constipation, diarrhoea dyspepsia, gastritis, nausea	Abdominal tenderness, aphthous stomatitis, stomach discomfort, tongue ulceration
**General disorders and administration site conditions**		Asthenia, fatigue	Chest pain, chills, hypothermia, pyrexia
**Hepatobiliary disorders**		Hepatomegaly	Hepatosplenomegaly, liver tenderness

6

System Organ Class	Common	Uncommon	Rare
**Immune system disorders**			Hypersensitivity
**Infections and infestations**		Gastroenteritis, malaria, oral herpes, respiratory tract infection, tinea capitis, upper respiratory tract infection, urinary tract infection	Bronchitis, bronchopneumonia, infection parasitic, pharyngitis, pharyngotonsillitis, *Plasmodium falciparum* infection, pneumonia, rhinitis, subcutaneous abscess, tracheobronchitis
**Investigations**	Transaminases increased (See section 4.4)	Blood albumin decreased, blood alkaline phosphatase increased, blood creatine phosphokinase increased, blood creatinine decreased, blood sodium increased, electrocardiogram abnormal, electrocardiogram QT prolonged (see section 4.4), liver function test abnormal	Blood albumin increased, blood bilirubin decreased, blood bilirubin increased, blood creatinine increased, blood potassium decreased, haematocrit increased, red blood cell count increased, white blood cells urine
**Metabolism and nutrition disorders**	Hypoglycaemia	Anorexia, hyperkalaemia	Decreased appetite, hyperglycaemia
**Musculoskeletal and connective tissue disorders**		Myalgia	Arthralgia, back pain
**Nervous system disorders**	Headache	Dizziness, dysgeusia, paraesthesia	Somnolence
**Pregnancy, puerperium and perinatal conditions**			Abortion complete
**Psychiatric disorders**		Insomnia	Sleep talking
**Renal and urinary disorders**		Haematuria, proteinuria	Ketonuria
**Reproductive system and breast disorders**			Vulvovaginal pruritus
**Respiratory, thoracic and mediastinal disorders**		Cough	Asthma, epistaxis, haemoptysis, rhinorrhoea
**Skin and subcutaneous tissue disorders**		Hyperhidrosis, pruritus, rash	Blister, dermatitis, urticaria papular

System Organ Class	Common	Uncommon	Rare
**Vascular disorders**			Hypertension, hypotension

* A rise in platelets generally from a low to normal level was commonly reported (≥1/100 to <1/10)

Description of selected adverse reactions
Changes in haematology parameters were generally of similar magnitude in all treatment groups and are expected consequences of malaria infection and treatment. Overall, white cell counts remained constant throughout treatment with falls in neutrophils and compensatory rises in lymphocytes and eosinophils.

Treatment with Pyramax, in keeping with other antimalarials, has caused reductions in haemoglobin of up to 2 g/dL and sometimes more. These generally reached a nadir by Day 3, recovering by Day 28.

In Phase II/III clinical trials that evaluated one single 3-days treatment course, Pyramax treatment was associated with mostly transient ALT elevations, with elevations of >3x upper limit of normal (ULN) and uncommonly, >10x ULN, with early onset peaking between Day 3 and 7 and normalising by Day 28.

In the Phase IIIb longitudinal study Pyramax was administered to patients experiencing single and repeated episodes of malaria and was shown to be similarly well tolerated on repeat administration as for first administration with repeat administration intervals as short as 28 days. The comparator arms of the study, artemether-lumefantrine or artesunate-amodiaquine, or a second study arm of DHA-piperaquine, also showed similar tolerability between initial and repeat administration. Where transient ALT elevations occurred, the adverse event profile was slightly higher with repeat administration for both adults and children.

In Episode 1, the percentage of patients with ALT > 1.5xULN and ≤ 3xULN for the 4 treatment arms was Pyramax 3.9%, artemether-lumefantrine 2.2%, artesunate-amodiaquine 1.2% and DHA-piperaquine 1.6%.

In Episode 2+, the percentage of patients with ALT >1.5xULN and ≤ 3xULN for the 4 treatment arms was Pyramax 5.1%, artemether-lumefantrine 2.2%, artesunate-amodiaquine 2.4% and DHA-piperaquine 2.2%.

Cases of syncope and isolated prolonged QTc were uncommonly reported in the available clinical trials for Pyramax. Mean decreases in heart rate were observed in all treatment groups and corresponded to reduction in the fever associated with the malaria infection (see section 4.4. and 5.1.).

In the Phase IIIb longitudinal study, Pyramax compared favourably to the other treatment arms in terms of QTc (Bazett and Fridericia) both on initial or any repeat dose (measured centrally).

In Episode 1, the percentage of patients with QTc (Bazett) >450 msec for the 4 treatment arms was Pyramax 4.0%, artemether-lumefantrine 10.3%, artesunate-amodiaquine 24.2% and DHA-piperaquine 34.1% and QTc (Fridericia), 0%, 0%, 5% and 7.2% respectively. No Pyramax patients had a QTc >480 msec.

In Episode 2+, the percentage of patients with QTc (Bazett) >450 msec for the 4 treatment arms was Pyramax 7.2%, artemether-lumefantrine 10%, artesunate-amodiaquine 41.1% and DHA-piperaquine 48.5% and QTc (Fridericia), 1.6%%, 2.9%, 9.55% and 17.6% respectively. No Pyramax patients had a QTc >500 msec.

Paediatric population

The frequency, type and severity of adverse reactions in children over 20 kg in body weight are expected to be similar to adults. Repeat dosing did not demonstrate a significant increase in adverse events versus one-time treatment with Pyramax tablets including in liver function changes.

Other specific populations

Except for findings regarding significant transient transaminase rises in Caucasian healthy volunteers, which may be linked to differences of pharmacokinetics due to non-infected state of healthy volunteers rather than to the potential difference of metabolic pathways between ethnic origins, no unexpected or clinically significant differences were observed in the analysis of adverse events and laboratory values by intrinsic factors (age group, gender, race, weight), extrinsic factors (region, study drug dose) or disease severity factors (previous malaria episode, number of previous malaria episodes in the last 12 months, baseline parasitaemia). In particular, patients with higher parasite loads (≥80.000/μL) were not at greater risk of adverse events, laboratory changes or electrocardiogram and cardiac events than the main population as a whole.

4.9 Overdose

No case of overdosage with Pyramax has been reported. In cases of suspected overdosage symptomatic and supportive therapy should be given as appropriate, transaminases (AST and ALT) should be monitored. If there are significant rises then serial total and direct bilirubin values should also be obtained to determine whether there is any change in liver function.

5. PHARMACOLOGICAL PROPERTIES

5.1 Pharmacodynamic properties

Pharmacotherapeutic group: pyronaridine in combination with artesunate, *an artemisinin derivative*, ATC Code: P01BF06

Mechanism of action

Pyronaridine inhibits the formation of β-haematin thus, preventing the malarial parasite from neutralizing haem, which is toxic to the parasite. Additionally, by forming a drug-haematin complex pyronaridine inhibits glutathione-dependent degradation of haematin and enhances haematin-induced lysis of red blood cells. Both these actions lead to parasite death.

Several mechanisms of action have been proposed to account for the activity of artemisinins; the generation of free radicals inside the parasite food vacuole and inhibition of the parasite's sarcoplasmic endoplasmic reticulum calcium-ATPase are widely accepted.

Pharmacodynamic effects

Whilst the outcome of *in vitro* studies using combinations of pyronaridine and artemisinin have reported mixed results, efficacy studies in rodent models of malaria using sensitive and resistant parasite strains have shown enhanced therapeutic effects using a combination of both compounds in a 3:1 ratio respectively.

Pyronaridine has potent *in vitro* activity against *P. falciparum* and *P. vivax* strains and clinical isolates including those resistant to other antimalarials. Against erythrocytic *P. falciparum* activity is greatest for the ring-form stage (ED_{50}; 8.3 nM), followed by schizonts ($ED_{50;}$ 11.6 nM) then trophozoites ($ED_{50;}$ 14.0 nM). Pyronaridine retains high activity against chloroquine resistant strains. *In vivo* efficacy of pyronaridine has been reported in mouse and non-human primate models of malaria.

Artesunate and its active principal metabolite dihydroartemisinin (DHA) show potent *in vitro* activity against multiple strains of *P. falciparum* and *P. vivax*, as well as against clinical isolates, including those resistant to other antimalarials. Reported IC_{50}s for inhibition of parasite multiplication are usually <19 nM. *In vivo* efficacy of artesunate has been reported in mouse, rat and non-human primate models of malaria.

Cross resistance

In vitro data from 181 clinical isolates showed that pyronaridine and artesunate were active against *P. falciparum* strains and isolates that were resistant to chloroquine, quinine, monodesethylamodiaquine, mefloquine or pyrimethamine and IC50 of both pyronaridine and artesunate were not affected by an increase in IC50 of chloroquine, monodesethylamodiaquine, mefloquine or pyrimethamine. In another *in vitro* study conducted against 104 multidrug resistant *P. falciparum* isolates from Southern Papua, pyronaridine demonstrated potent activity against isolates resistant to chloroquine, amodiaquine (another quinoline-type Mannich base) and piperaquine.

Cross resistance to other antimalarials cannot be ruled out.

Resistance to artemisinin has been reported in clinical isolates *in vitro* and genetically stable resistance has been observed in animal models. Resistance has been reported as labile and difficult to induce experimentally in animals; however, those data cannot be extrapolated to humans *in vivo*. The threshold for resistance of *P. falciparum* to artesunate remains indeterminate however, prolonged parasite clearance times in patients with apparent artemisinin resistance have recently been described in Western Cambodia.

Clinical efficacy

Plasmodium falciparum malaria:
Pyramax was demonstrated in Phase III clinical studies in both the Per Protocol (PP) and Intent to Treat (ITT) populations to be non-inferior to artemether-lumefantrine and mefloquine + artesunate in the treatment of acute uncomplicated *P. falciparum* in 2280 children and adults for the primary endpoint of polymerase chain reaction (PCR)-adjusted adequate clinical and parasitological response (ACPR) at 28 days. In addition, Pyramax was also found to be non-inferior to the comparator agents for the secondary endpoints of parasite PCR-adjusted ACPR at 42 days. Pyramax was rapidly effective, with more than 90% of subjects clearing parasites and fever within 48 hours. Parasite count (*P. falciparum* asexual forms) decreased rapidly (during the first 16 hours) in both the Pyramax and comparator groups. Time to parasite clearance was statistically significantly shorter in the Pyramax group compared with artemether-lumefantrine group based on the log-rank test. In the integrated analysis of all Phase III studies with *P. falciparum*, no clinically important differences in PCR-adjusted ACPR were observed by region, age, gender, race, weight, previous malaria episode, baseline parasitaemia, or formulation. Crude cure rate results were also similar. The median time to fever clearance was 15.5 hours.

In all studies conducted in *P. falciparum* malaria, there was a marginal but consistently longer gametocytes clearance time in the Pyramax groups as compared to mefloquine plus artesunate or artemether-lumefantrine groups. Further trials are awaited to address the mosquito infectivity.

In an analysis of a repeat-dose longitudinal study of 1342 patients treated with Pyramax tablets and granules for oral suspension, examining safety and efficacy of repeat dosing; of the 770 patients weighing ≥ 20 kg, 434 (56.4%) received at least one further treatment and 31.6% had a second or more re-treatment. Reasons for non-inclusion into the study or non re-treatment were complicated malaria or hyperparasitaemia or significantly raised liver enzymes as well as comorbidities such as HIV, hepatitis, or severe malnutrition. Efficacy findings were similar to those in pivotal trials and were maintained with repeated treatment episodes. Patients previously excluded or poorly represented in the clinical studies will be included in a pharmacovigilance study being conducted in endemic areas.

The PCR-adjusted ACPR at Day 28 for the initial malaria episode for all four treatment arms (all patients) were, respectively in ITT and PP population, 95.7% / 99.8% for Pyramax, 90.0% / 99.0% for artemether-lumefantrine, 94.1% / 99.8% for artesunate-amodiaquine as direct comparators, while it was 96.3% / 99.9% for DHA-piperaquine. At Day 42, these values were 85.6% / 99.6%, 73.5% / 99.0%, 80.5% / 99.4% and 92.0% / 99.9% respectively. Subsequent episodes in the ITT/PP population are shown to demonstrate similar outcomes.

For patients weighing ≥20 kg, in the ITT population, the PCR-adjusted ACPR at Day 28 for the four treatment arms were 97.1%, 93.7%, 95.2%, 97.3% respectively and at Day 42 were 91.8%, 79.1%, 82.8%, 94.2% respectively.

Plasmodium vivax malaria:
In the studies in subjects with *P. vivax* malaria, non-inferiority of Pyramax compared with chloroquine was demonstrated with respect to the crude cure rate on Day 14 in the efficacy evaluable population (in children and adults), which was the primary end point in that study. Results were maintained in the intent-to-treat population. A high crude cure rate (95.5%) was still observed at Day 42. Times to fever and parasite clearance were significantly shorter for Pyramax than chloroquine in this study. Only 13 patients less than 12 years old (no patient less than 7 years) were treated with Pyramax for *P. vivax* malaria. At the time the study was conducted, the areas where the studies were performed had low chloroquine resistance to *P. vivax*.

5.2 Pharmacokinetic properties

There is no pharmacokinetic interaction between pyronaridine tetraphosphate and artesunate at the recommended dose.

In clinical trials trough levels of pyronaridine and artesunate in children were generally within the range observed in adults.

Absorption

Following administration of Pyramax tablets to healthy volunteers and patients with malaria, peak plasma concentrations are generally reached between 0.5 and 1.0 hours post-dose for artesunate, between 1 and 2 hours post-dose for DHA and between 2 and 8 hours post-dose for pyronaridine. Exposure to artesunate and pyronaridine was increased by 34% and 20% respectively when Pyramax was administered with a high fat meal, however these effects were not judged clinically significant and patients can take Pyramax tablets without regard to meals (see section 4.2).

Distribution

Pyronaridine and its metabolites are extensively distributed into tissues, with highest concentrations achieved in the liver, spleen, adrenal gland, kidney and thyroid gland in the rat. There is evidence that pyronaridine binds to melanin in the eye. In the dog, approximately 6% of a single dose of pyronaridine remained in the liver 24 months after administration. The potential extrapolation to human is not elucidated but the very slow elimination of pyronaridine-related material from the body means that accumulation, with possible hepatotoxicity, cannot be ruled out if pyronaridine is readministered too early.

Pyronaridine preferentially associates with blood cells, exhibiting a whole blood/plasma concentration ratio of approximately 1.5:1. Pyronaridine is highly bound to human serum proteins *in vitro* (92 to 95%). Pyronaridine displays two-compartment pharmacokinetic characteristics with a blood level profile that has a distinct distribution phase.

Artesunate and its metabolites are primarily associated in the rat with tissues involved in absorption and excretion and high levels were also found in the spleen.

Plasma protein binding of artesunate and DHA is moderate (62 to 93%) and albumin is the principal binding protein for DHA in human plasma.

Biotransformation

Pyronaridine appears to have a large number of potential metabolites, with no clear major metabolic route. Human *in vivo* metabolic profiling was conducted in blood, urine, and faecal samples from six healthy male volunteers in a microdose radioactivity mass balance study. Pyronaridine (unchanged) and a total of thirteen metabolites were identified in one or more sample matrices. Proposed metabolic pathways include: *N*-dearylation, oxidation, de-methylation, glucuronidation, cysteine conjugation, acetylation and reduction.

Artesunate is very rapidly metabolized by esterases to the active metabolite dihydroartemisinin (DHA). DHA is subsequently conjugated with glucuronic acid via UGT1A9 and UGT2B7.

Elimination

Pyronaridine is eliminated slowly from blood, with an elimination half-life in adults of between 14 and 18 days for parent compound, and a mean of 33.5 days for total drug-related material. Urinary excretion of unchanged pyronaridine is <2% in healthy human subjects. Data from the mass balance study with pyronaridine in healthy volunteers indicates that faeces excretion is the main route of elimination of drug-related material. In this study, pyronaridine-related material was excreted both via faeces (47.8%) and urine (23.7%) after oral dosing of pyronaridine to healthy human subjects. Elimination occurred very slowly, the mean recovery of 71.5% (range 60.3%-82.2%) was achieved by 86 days after dosing.

In patients with uncomplicated malaria, artesunate and DHA are cleared from plasma with an elimination half-life of about 0.5 and 0.8 hours, respectively. No urinary excretion data are available for humans.

Hepatic and Renal Impairment

Pyramax has not been studied for efficacy and safety in patients with severe hepatic and/or severe renal impairment (see section 4.2).

Elderly Patients

No specific pharmacokinetic studies have been performed in patients older than 65 years of age.

5.3 Preclinical safety data

Repeat-dose toxicity studies with pyronaridine tetraphosphate:artesunate (3:1) in rats and dogs produced similar effects to those seen with each component individually.

The predominant feature in animals receiving repeated higher doses of pyronaridine tetraphosphate:artesunate (3:1) was related to the accumulation of pyronaridine.

Microscopically, after repeated dosing, this was seen as a widespread accumulation of basophilic material in many tissues and organs, sometimes present without associated inflammatory change (as for bone marrow and eye) but more often associated with dose-related inflammatory changes (as for liver, lung, spleen, gall bladder and kidney). It should be noted that, following a single 3-day cycle of treatment in dog, inflammatory changes were confined to liver and brain.

These inflammatory changes are considered secondary to the body’s attempt to clear the accumulated material, and an increase in white blood cell count, predominantly in neutrophils and monocytes, is

also considered a sequela of these changes. In more reactive tissues, notably rat liver, inflammatory and degenerate changes worsened over time in response to the prolonged presence of material, and this was correlated with increasing transaminase levels. This increase in severity was not evident following a single cycle of treatment.

Minimal to mild perivasculitis of the brain was noted in all repeat dose dog studies, including the single cycle study. This finding occurred with dose-related incidence, was not associated with relevant neurobehavioural changes and was not fully reversible.

Thymus atrophy was observed after administration of pyronaridine and artesunate to rats and dogs.

HERG studies were performed with pyronaridine, artesunate and dihydroartemisinin (DHA). Those studies showed that artesunate seldom had an effect on hERG tail current up to 300 µM (115.3 µg/mL) and that DHA and pyronaridine both inhibited hERG tail current with IC50s of 282.7 µM and 0.65 µM, respectively.

Pyronaridine was clastogenic in *in vitro* chromosome aberration tests and mouse lymphoma assays. The positive findings *in vitro* with mammalian cells are consistent likely related to the potential of pyronaridine for topoisomerase II inhibition. Pyronaridine was negative in the *in vivo* mouse bone marrow micronucleus test and rat liver *in vivo/in vitro* Unscheduled DNA Synthesis assay. In the rat liver comet assay negative results were obtained at liver concentrations 45-fold higher than the estimated liver concentrations reached in humans. Overall, the genotoxic risk associated with the proposed treatment cycle using pyronaridine should be no greater than that associated with other current therapies. Artesunate was not genotoxic in a standard package of genotoxicity assays. Carcinogenicity studies were not conducted since the treatment is limited to 3 days.

Neither pyronaridine tetraphosphate nor artesunate have effects on rat fertility. Artesunate is embryolethal at varying maternal dose levels and dosing regimens, depending on the nonclinical species. In fact, together with most other artemisinins (dihydroartemisinin, arteether, artemether) artesunate acts by depleting embryonic erythroblasts leading to severe anaemia. In cynomolgus monkeys, embryo lethality was observed in monkeys treated with artesunate for 30 days during the period of organogenesis, whereas no embryo lethality was observed in monkeys treated for a 3- or 7-day period during organogenesis, at comparable dose (which is 3 times the human dose based on mg/kg). In rats and rabbits, artesunate also caused embryolethality and foetotoxicity (decreased foetal body weight and increased skeletal and visceral variations).

Pyronaridine was shown to cross the placenta in rats. At maternally toxic doses, it caused early resorptions and abortions in rabbits, and decreased foetal body weight in rats and rabbits. There was no evidence of teratogenicity in both species.

6. PHARMACEUTICAL PARTICULARS

6.1 List of excipients

Tablet core
Microcrystalline cellulose
Crospovidone
Mannitol (E421)
Magnesium stearate
Talc
Hypromellose
Macrogol 6000

Film coating
Hypromellose

Titanium dioxide
Tartrazine (E102)
Macrogol 6000
Sunset yellow FCF (E110)

6.2 Incompatibilities

Not applicable.

6.3 Shelf life

2 years.

6.4 Special precautions for storage

Do not store above 30°C.
Store in the original package.

6.5 Nature and contents of container

Aluminium/PVC/Aluminium-oPA foil blisters containing 9 tablets.
The blisters are packed into cartons containing one or 10 blisters.

6.6 Special precautions for disposal

No special requirements.

Patients should be advised not to throw away any medicines via wastewater or household waste and ask their health provider how to dispose of unused medication.

7. SUPPLIER

Shin Poong Pharmaceutical Co., Ltd
161, Yeoksam-ro
Gangnam-gu
Seoul
South Korea

8. MARKETING AUTHORISATION NUMBER(S)

Not applicable.

9. DATE OF FIRST AUTHORISATION/RENEWAL OF THE AUTHORISATION

Not applicable.

10. DATE OF REVISION OF THE TEXT

Detailed information on this product is available on the website of the European Medicines Agency (EMA) http://www.ema.europa.eu

1. NAME OF THE MEDICINAL PRODUCT

Pyramax 60 mg/20 mg Granules for oral suspension

2. QUALITATIVE AND QUANTITATIVE COMPOSITION

Each sachet of Pyramax Granules for oral suspension contains 60 mg Pyronaridine tetraphosphate and 20 mg Artesunate.

Excipients with known effect: each sachet contains 0.05 mg Sunset yellow FCF (E110) and 0.15 mg Tartrazine (E102).
For a full list of excipients, see section 6.1.

3. PHARMACEUTICAL FORM

Pyramax granules for oral suspension
Orange coloured granules

4. CLINICAL PARTICULARS

4.1 Therapeutic indications

Pyramax Granules for oral suspension are indicated in the treatment of acute, uncomplicated malaria infection caused by *Plasmodium falciparum* or by *Plasmodium vivax* in children and infants weighing 5 kg to under 20 kg.

Consideration should be given to official guidance on the appropriate use of antimalarial agents (see section 4.4).

4.2 Posology and method of administration

Mode of administration
The dose should be taken orally once a day for three days with or without food.

Posology

Dosage for Granules for oral suspension in children and infants
Pyramax Granules for oral suspension should be taken orally as a single daily dose for three consecutive days.

Body weight	Number of granules sachets	Regimen
5 - < 8 kg	1 sachet	Daily for 3 days
8 - < 15 kg	2 sachets	Daily for 3 days
15 - < 20 kg	3 sachets	Daily for 3 days

A tablet formulation is available for children weighing 20 kg and over.

Administration of Pyramax Granules for oral suspension:

Add a small amount of water (approximately 10 ml i.e. 2 teaspoons) into a small cup. Put the contents of the required number of sachets (based on the weight of the child) into the cup and stir gently until the granules are suspended evenly. The granules will not dissolve. The patient should swallow the suspension immediately. Add a small amount of water (approximately 10 ml i.e. 2 teaspoons) to the cup to mix any remaining granules and the suspension should then be immediately swallowed by the

patient. It is recommended to repeat this step until the patient has swallowed all the granules and no granules remain in the cup.

Only drinking water should be used for preparation of the oral suspension. Administration with feeding tubes has not been studied. Caution should be exercised to avoid the risk of aspiration in very young children.

In the event of vomiting within 30 minutes of administration after the first dose, a repeat dose should be given. If the repeat dose is vomited, the patient should be given an alternative antimalarial drug. In the event of non-severe diarrhoea normal dosing should be continued.

If a dose is missed, it should be taken as soon as possible and then the recommended regimen continued until the full course of treatment has been completed.

Dosage in paediatrics population
Pyramax is dosed according to body weight. Safety and efficacy of Pyramax granules for oral suspension has been established in infants and children weighing 5 kg to below 20 kg, but not in children less than 5 kg. The clinical studies conducted in *Plasmodium vivax* malaria, included only 13 patients below 12 years old (see section 5.1.)

Elderly
Not applicable. Pyramax Granules for Oral Suspension are intended for children and infants weighing 5 kg to under 20 kg.

Dosage in hepatic and renal impairment

There is no information on dosing in patients with hepatic impairment. Due to its potential liver toxicity Pyramax is contraindicated in patients with signs of hepatic impairment or known significant liver function test abnormalities.

There is no information on dosing patients with severe renal impairment. Although excretion via faeces was the main route of elimination of pyronaridine-related material in a human mass balance study, significant urinary excretion was also observed. Pyramax is, therefore, contraindicated in the case of severe renal impairment and caution should be exercised when treating patients with mild or moderate renal impairment.

4.3 Contraindications

- Known hypersensitivity to pyronaridine or artesunate or any component of the formulation.

- Patients with clinical signs or symptoms of hepatic injury (such as nausea and/or abdominal pain associated with jaundice) or known severe liver disease (i.e. decompensated cirrhosis, Child-Pugh stage B or C).

- Severe renal impairment.

4.4 Special warnings and precautions for use

Pyramax should not be used as a prophylactic treatment of malaria.

Pyramax has been associated, in some patients, with transient increases in liver enzymes without clinical signs (see section 4.8). Data are very limited for infants weighing less than 8 kg (see section 5.1). Pyramax is contra-indicated in the case of underlying hepatic injury, clinical signs or symptoms of hepatic injury or known severe liver disease (see section 4.3). If a patient is already known to have elevated transaminases the use of Pyramax is not recommended.

Patients or their parent/guardian should be advised of the clinical signs and symptoms of hepatotoxicity in order to monitor closely if such signs or symptoms occur, especially in the first two weeks after Pyramax intake. It is recommended that, in patients who exhibit symptoms of hepatotoxicity following treatment with Pyramax, the liver function tests be monitored if possible, until normalisation.

No data are available in patients with co-infections (HBV, HCV, HIV); those receiving co-administration of drugs known to be associated with mitochondrial toxicity (i.e. valproate, antiretroviral drugs), use of herbal medicines, patients with malnutrition or patients with other hepatic underlying conditions (i.e. ethanol intoxication, hepatic steatosis). Particular caution is advised in these patients regarding the risk of liver toxicity since these risk factors, also including co-administration of paracetamol, might produce a cumulative effect on the liver. Enhanced surveillance is warranted in young children in case of malnutrition.

No specific QT/QTc study has been performed to specifically assess the cardiac safety of Pyramax. Based on the available comparative clinical studies, this risk does not appear to be higher with single or repeat administration of Pyramax as compared to the other available antimalarial drugs used in these trials (artesunate + mefloquine, chloroquine, artemether-lumefantrine). However, patients with known history or evidence of clinically significant cardiovascular disorders (including arrhythmia, QTc interval ≥ 450 milliseconds) were excluded from these clinical studies. Therefore, caution should be exercised in at risk patients i.e. those:

- with congenital prolongation of QTc interval, hypokalaemia, dehydration, cardiac arrhythmia, heart failure, etc.
- treated concomitantly with other drugs that can block potassium channels, such as antiarrhythmics, neuroleptics, certain antimicrobial agents (e.g. macrolides, fluoroquinolones, imidazole and triazole antifungals, pentamidine, saquinavir) and non-sedating antihistamines, cisapride, domperidone or methadone
- recently treated with medicinal products with long elimination half-life and known to prolong the QTc interval that may still be circulating at the time Pyramax treatment course is commenced (see section 4.8. and 5.1.).

A fall in haemoglobin may occur during treatment. There is very little information on the clinical effect of this in patients with initial haemoglobin levels of less than 8 g/dl. Caution should be exercised in treating patients with a low haemoglobin.

Pyramax should not be used for the treatment of severe malaria, cerebral malaria or other severe manifestations of complicated malaria, including hyperparasitaemia, pulmonary oedema, severe anaemia, renal or hepatic failure. Patients with severe malaria are not candidates for oral therapy.

In patients with acute malaria who present with severe diarrhoea and vomiting, alternative therapy should be considered. If Pyramax is used in these patients, the parasite load should be closely monitored.

Pyramax is a blood schizonticide and for the treatment of *P. vivax* malaria, a radical cure (to destroy the parasite in the liver and thus prevent relapse) is required with a hypnozoitocidal drug such as primaquine.

In the event of proven or suspected recrudescent malaria infections after treatment with Pyramax, patients should be treated with a different blood schizonticide.

Artemisinin compounds should not be used for treatment of malaria in the first trimester of pregnancy if other suitable and effective antimalarials are available (See Section 4.6).

There is no experience in the treatment of mixed *P. vivax* and *P. falciparum* infections. No data are available with Pyramax in the treatment of malaria due to *Plasmodium malariae* or *Plasmodium ovale*.

The safety and effectiveness of Pyramax for the treatment of malaria in patients with HIV/AIDS has not been established. If Pyramax is used in these patients, the parasite load should be closely monitored.

This medicine contains tartrazine (E102) and sunset yellow (E110) as colouring agents which may cause allergic reactions which may manifest as flushing, the appearance of wheals/urticarial, breathlessness, faintness and/or fall in blood pressure.

4.5 Interaction with other medicinal products and other forms of interaction

Particular caution is advised in case of co-administration of drugs known to be associated with mitochondrial toxicity (i.e. valproate, antiretroviral drugs), use of herbal medicines, and also co-administration of paracetamol (see section 4.4).

Pyronaridine shows *in vitro* CYP2D6 inhibitory potential that was confirmed *in vivo* using metoprolol as CYP2D6 probe. The study showed an increase of metoprolol Cmax of around 50% but the overall exposure increased to a lesser extent. Caution is therefore advised when co-administering Pyramax with metoprolol given in cardiac failure, notably during the titration phase and a possible dose adjustment may be required. This applies to flecainide and propafenone as well, two antiarrhythmics exclusively metabolised by CYP2D6.

As pyronaridine shows *in vitro* P-gp inhibitory potential, substrates for P-gp such as digoxin and dabigatran may require additional monitoring of blood levels and possible dose adjustment as well.

The combination of Pyramax and primaquine has shown neither clinically relevant pharmacokinetic variations nor any impaired tolerance. If needed, the two antimalarial drugs may be co-administered.

Dihydroartemisinin (DHA) administration may result in a slight decrease in CYP1A2 activity. Caution is therefore, advised when Pyramax is administered concomitantly with medicinal products metabolised by this enzyme that have a narrow therapeutic index, such as theophylline. Any effects are unlikely to persist beyond 24 hours after the last intake of DHA.

Enzyme inducing medicinal products such as rifampicin, carbamazepine, phenytoin, phenobarbital, St. John's wort (*Hypericum perforatum*) may lead to reduced DHA plasma concentrations.

4.6 Fertility, pregnancy and lactation

Fertility
In animal studies, no effects on fertility and reproductive performance were observed. In these studies, the exposure to artesunate was below the human exposure; the maximum exposure to pyronaridine was 3-fold higher than the proposed human exposure.

Pregnancy
Not applicable. Pyramax Granules for Oral Suspension are intended for children and infants weighing 5 kg to under 20 kg.

Pregnancy register
Not applicable. Pyramax Granules for Oral Suspension are intended for children and infants weighing 5 kg to under 20 kg.

Lactation
Not applicable. Pyramax Granules for Oral Suspension are intended for children and infants weighing 5 kg to under 20 kg.

4.7 Effects on ability to drive and use machines

Not applicable. Pyramax Granules for Oral Suspension are intended for children and infants weighing 5 kg to under 20 kg.

4.8 Undesirable effects

The safety of pyronaridine tetraphosphate and artesunate for treatment of malaria has been evaluated in clinical trials of more than 4000 patients.

Summary of the safety profile
The most commonly reported (≥1/100 to <1/10) adverse event were headache, eosinophilia, neutropenia, anaemia, increased platelet count, vomiting, abdominal pain, bradycardia, transaminase increases and hypoglycaemia.

Tabulated list of adverse reactions
The following table provides a summary of adverse reactions reported with Pyramax for both tablets and granules in clinical trial reports. Adverse reactions are ranked under headings of frequency using the MedDRA frequency convention: very common (≥1/10); common (≥1/100 to <1/10); uncommon (≥1/1000 to <1/100); rare (<1/1000).

System Organ Class	Common	Uncommon	Rare
**Blood and lymphatic system disorders**	Anaemia, eosinophilia, neutropenia, increased platelet count*	Basophilia, leukocytosis, leukopenia, lymphocytosis, monocytosis, splenomegaly, thrombocytopenia	Lymphopenia, pancytopenia
**Cardiac disorders**	Bradycardia	Palpitations, ventricular extrasystoles	Arrhythmia, atrioventricular block first degree, sinus arrhythmia
**Ear and labyrinth disorders**		Vertigo	Ear pain, hearing impaired, tinnitus
**Eye disorders**			Conjunctivitis
**Gastrointestinal disorders**	Abdominal Pain, Vomiting	Constipation, diarrhoea dyspepsia, gastritis, nausea	Abdominal tenderness, aphthous stomatitis, stomach discomfort, tongue ulceration
**General disorders and administration site conditions**		Asthenia, fatigue	Chest pain, chills, hypothermia, pyrexia
**Hepatobiliary disorders**		Hepatomegaly	Hepatosplenomegaly, liver tenderness
**Immune system disorders**			Hypersensitivity
**infections and infestations**		Gastroenteritis, malaria, oral herpes, respiratory tract infection, tinea capitis, upper respiratory tract infection, urinary tract infection	Bronchitis, bronchopneumonia, infection parasitic, pharyngitis, pharyngotonsillitis, *Plasmodium falciparum* infection, pneumonia, rhinitis, subcutaneous abscess, tracheobronchitis
**Investigations**	Transaminases increased (See section 4.4)	Blood albumin decreased, blood alkaline phosphatase increased, blood creatine phosphokinase increased, blood creatinine decreased, blood sodium increased, electrocardiogram abnormal, electrocardiogram QT prolonged (see section 4.4), liver function test abnormal	Blood albumin increased, blood bilirubin decreased, blood bilirubin increased, blood creatinine increased, blood potassium decreased, haematocrit increased, red blood cell count increased, white blood cells urine
**Metabolism and nutrition disorders**	Hypoglycaemia	Anorexia, hyperkalaemia	Decreased appetite, hyperglycaemia
**Musculoskeletal and connective tissue disorders**		Myalgia	Arthralgia, back pain
**Nervous system disorders**	Headache	Dizziness, dysgeusia, paraesthesia	Somnolence
**Pregnancy, puerperium and perinatal conditions**			Abortion complete
**Psychiatric disorders**		Insomnia	Sleep talking
**Renal and urinary disorders**		Haematuria, proteinuria	Ketonuria
**Reproductive system and breast disorders**			Vulvovaginal pruritus
**Respiratory, thoracic and mediastinal disorders**		Cough	Asthma, epistaxis, haemoptysis, rhinorrhoea
**Skin and subcutaneous tissue disorders**		Hyperhidrosis, pruritus, rash	Blister, dermatitis, urticaria papular
**Vascular disorders**			Hypertension, hypotension

* A rise in platelets generally from a low to normal level was commonly reported (≥ 1/100 to < 1/10)

19

20

Description of selected adverse reactions
Changes in haematology parameters were generally of similar magnitude in all treatment groups and are expected consequences of malaria infection and treatment. Overall, white cell counts remained constant throughout treatment with falls in neutrophils and compensatory rises in lymphocytes and eosinophils.

Treatment with Pyramax, in keeping with other antimalarials, has caused reductions in haemoglobin of up to 2 g/dL and sometimes more. These generally reached a nadir by Day 3 recovering by Day 28.

In Phase II/III clinical trials evaluating one single 3-day treatment course, Pyramax treatment was associated with mostly transient ALT elevations, with elevations of > 3x upper limit of normal (ULN) and uncommonly, > 10x ULN with early onset peaking between Day 3 and 7 and normalising by Day 28.

In the Phase IIIb longitudinal study Pyramax was administered to patients experiencing single and repeated episodes of malaria and was shown to be similarly well tolerated on repeat administration, as for first administration with repeat administration intervals as short as 28 days. The comparator arms of the study, artemether-lumefantrine or artesunate-amodiaquine, or a second study arm of DHA-piperaquine, also showed similar tolerability between initial and repeat administration. Where transient ALT elevations occurred, the adverse event profile was similar with repeat administration for both adults and children based on data associated with the treatment of Episode 1 and any repeat treatment (Episodes 2+) for all treatment arms in terms of liver enzyme classifications for the highest post Day 0 values.

A sub analysis of liver function tests in the granules population of the longitudinal study was performed and the incidence of ALT values relative to the normal range, by body weight <10 kg or ≥10 kg for the three treatment arms using the highest post-Day 0 value were similar across the treatment groups and weight categories.

One potential Hy's law case on Pyramax were seen in the <10 kg body weight group occurring in Episode 3. There was one case of potential Hy's law case on Pyramax were seen in the ≥ 10 kg body weight group occurring in Episode 1 and, in this case, the patient was subsequently retreated with no recurrence of the hepatotoxicity. There were two Hy's law cases reported in the artemether-lumefantrine group in the ≥ 10 kg body weight group; one case occurring in Episode 1 and the other on re-treatment.

Cases of syncope and isolated prolonged QTc were uncommonly reported in the available clinical trials. Mean decreases in heart rate were observed in all treatment groups and corresponded to reduction in the fever associated with the malaria infection (see section 4.4. and 5.1.).

In the Phase IIIb longitudinal study, Pyramax compared favourably to the other treatment arms in terms of QTc (Bazett and Fridericia) both on initial or any repeat dose (measured centrally).

In Episode 1, the percentage of patients with QTc (Bazett) >450 msec for the 4 treatment arms was Pyramax 4.0%, artemether-lumefantrine 10.3%, artesunate-amodiaquine 24.2% and DHA-piperaquine 34.1% and QTc (Fridericia), 0%, 0%, 5% and 7.2% respectively. No Pyramax patients had a QTc >480 msec.

In Episode 2+, the percentage of patients with QTc (Bazett) >450 msec for the 4 treatment arms was Pyramax 7.2%, artemether-lumefantrine 10%, artesunate-amodiaquine 41.1% and DHA-piperaquine 48.5% and QTc (Fridericia), 1.6%%, 2.9%, 9.55% and 17.6% respectively. No Pyramax patients had a QTc >500 msec.

There were no post dose QTc (Bazett) values >500 msec and no post dose QTc (Fridericia) values >450 msec nor any increases of >60 msec from baseline in the granules population including patients less than 10 kg.

Paediatric population
The frequency, type and severity of adverse reactions in children 5 kg and over in body weight are similar to adults, however, to date, very few patients weighing less than 8 kg have been treated with Pyramax. Repeat dosing in the 128 children re-dosed at least once with Pyramax granules did not demonstrate a significant increase in adverse events versus one-time treatment with Pyramax including in liver function changes.

Other specific populations
Except for findings regarding significant transient transaminase rises in Caucasian healthy volunteers - which may be linked to differences of pharmacokinetics due to non-infected state of healthy volunteers rather than to the potential difference of metabolic pathways between ethnic origins - no unexpected or clinically significant differences were observed in the analysis of adverse events and laboratory values by intrinsic factors (age group, gender, weight), extrinsic factors (region, study drug dose) or disease severity factors (previous malaria episode, number of previous malaria episodes in the last 12 months, baseline parasitaemia) and in particular, patients with higher parasite loads (≥80.000/µL) were not at greater risk of adverse events, laboratory changes or electrocardiogram and cardiac events than the main population as a whole.

4.9 Overdose

No case of overdosage with Pyramax has been reported. In cases of suspected overdosage symptomatic and supportive therapy should be given as appropriate, transaminases (AST and ALT) should be monitored. If there are significant rises then serial total and direct bilirubin values should also be obtained to determine whether there is any change in liver function.

5. PHARMACOLOGICAL PROPERTIES

5.1 Pharmacodynamic properties

Pharmacotherapeutic group: pyronaridine in combination with artesunate, *an artemisinin derivative*, ATC Code: P01BF06

Mechanism of action

Pyronaridine inhibits the formation of β-haematin thus, preventing the malarial parasite from neutralizing haem, which is toxic to the parasite. Additionally, by forming a drug-haematin complex pyronaridine inhibits glutathione-dependent degradation of haematin and enhances haematin-induced lysis of red blood cells. Both these actions lead to parasite death.

Several mechanisms of action have been proposed to account for the activity of artemisinins; the generation of free radicals inside the parasite food vacuole and inhibition of the parasite's sarcoplasmic endoplasmic reticulum calcium-ATPase are widely accepted.

Pharmacodynamic effects

Whilst the outcome of *in vitro* studies using combinations of pyronaridine and artemisinin have reported mixed results, efficacy studies in rodent models of malaria using sensitive and resistant parasite strains have shown enhanced therapeutic effects using a combination of both compounds in a 3:1 ratio respectively.

Pyronaridine has potent *in vitro* activity against *P. falciparum* and *P. vivax* strains and clinical isolates including those resistant to other antimalarials. Against erythrocytic *P. falciparum* activity is greatest for the ring-form stage (ED_{50}; 8.3 nM), followed by schizonts ($ED_{50;}$ 11.6 nM) then trophozoites ($ED_{50;}$ 14.0 nM). Pyronaridine retains high activity against chloroquine resistant strains. *In vivo* efficacy of pyronaridine has been reported in mouse and non-human primate models of malaria.

Artesunate and its active principal metabolite dihydroartemisinin (DHA) show potent *in vitro* activity against multiple strains of *P. falciparum* and *P. vivax*, as well as against clinical isolates, including those resistant to other antimalarials. Reported IC_{50}s for inhibition of parasite multiplication are usually <19 nM. *In vivo* efficacy of artesunate has been reported in mouse, rat and non-human primate models of malaria.

Cross resistance

In vitro data from 181 clinical isolates showed that pyronaridine and artesunate were active against *P. falciparum* strains and isolates that were resistant to chloroquine, quinine, monodesethylamodiaquine, mefloquine or pyrimethamine and IC50 of both pyronaridine and artesunate were not affected by an increase in IC50 of chloroquine, monodesethylamodiaquine, mefloquine or pyrimethamine. In another *in vitro* study conducted against 104 multidrug resistant *P. falciparum* isolates from Southern Papua, pyronaridine demonstrated potent activity against isolates resistant to chloroquine, amodiaquine (another quinoline-type Mannich base) and piperaquine.

Cross resistance to other antimalarials cannot be ruled out.

Resistance to artemisinin has been reported in clinical isolates *in vitro* and genetically stable resistance has been observed in animal models. Resistance has been reported as labile and difficult to induce experimentally in animals; however those data cannot be extrapolated to humans *in vivo*. The threshold for resistance of *P. falciparum* to artesunate remains indeterminate however, prolonged parasite clearance times in patients with apparent artemisinin resistance have recently been described in Western Cambodia.

Clinical efficacy

Plasmodium falciparum malaria:
Pyramax was demonstrated in Phase III clinical studies in both the Per Protocol (PP) and Intent to Treat (ITT) populations to be non-inferior to artemether-lumefantrine and mefloquine + artesunate in the treatment of acute uncomplicated *P. falciparum* in 2280 children and adults for the primary endpoint of polymerase chain reaction (PCR)-adjusted adequate clinical and parasitological response (ACPR) at 28 days. In addition, Pyramax was also found to be non-inferior to the comparator agents for the secondary endpoints of parasite PCR-adjusted ACPR at 42 days. Pyramax was rapidly effective, with more than 90% of subjects clearing parasites and fever within 48 hours. Parasite count (*P. falciparum* asexual forms) decreased rapidly (during the first 16 hours) in both the Pyramax and comparator groups. Time to parasite clearance was statistically significantly shorter in the Pyramax group compared with artemether-lumefantrine group based on the log-rank test. In the integrated analysis of all Phase III studies with *P. falciparum*, no clinically important differences in PCR-adjusted ACPR were observed by region, age, gender, race, weight, previous malaria episode, baseline parasitaemia, or formulation. Crude cure rate results were also similar. The median time to fever clearance was 15.5 hours.

In all studies conducted in *P. falciparum* malaria, there was a marginal but consistently longer gametocytes clearance time in the Pyramax groups as compared to mefloquine plus artesunate or artemether-lumefantrine groups. Further trials are awaited to address the mosquito infectivity.

In an analysis of a longitudinal study of 1342 patients treated with Pyramax tablets and granules for oral suspension, examining safety and efficacy of repeat dosing; 572 patients were between 5 and under 20 kg. Three hundred and ninety-three (393) patients received treatment with Pyramax for more than one malaria episode (68.7%) and 277 (48.4%) were treated for 3 or more malaria episodes. To date 43 patients under 10 kg received Pyramax granules for oral suspension in the longitudinal study. Reasons for non-inclusion into the study or non re-treatment were complicated malaria or hyperparasitaemia or significantly raised liver enzymes as well as comorbidities such as HIV, hepatitis, or severe malnutrition. Efficacy findings were similar to those in pivotal trials and were

maintained with repeated treatment episodes. Patients previously excluded or poorly represented in the clinical studies will be included in a pharmacovigilance study being conducted in endemic areas.

For patients weighing < 20 kg the PCR-adjusted ACPR in the ITT population at Day 28 for the four treatment arms were 94% Pyramax, 83.1% artemether-lumefantrine, 93.1% artesunate-amodiaquine, 95% DHA-piperaquine respectively and at Day 42 were 77.3%, 63%, 78.3%, 89.4% respectively.

Plasmodium vivax malaria:
In the studies in subjects with *P. vivax* malaria, non-inferiority of Pyramax compared with chloroquine was demonstrated with respect to the crude cure rate on Day 14 in the efficacy evaluable population (in children and adults), which was the primary end point in that study. Results were maintained in the intent-to-treat population. A high crude cure rate (95.5%) was still observed at Day 42. Times to fever and parasite clearance were significantly shorter for Pyramax than chloroquine in this study. Only 13 patients less than 12 years old (no patient less than 7 years) were treated with Pyramax for *P vivax* malaria. At the time the study was conducted, the areas where the studies were performed had low chloroquine resistance to *P vivax*.

5.2 Pharmacokinetic properties

Information on the pharmacokinetic of pyronaridine tetraphosphate and artesunate is mainly derived from the use of the tablet formulation.

There is no pharmacokinetic interaction between pyronaridine tetraphosphate and artesunate at the recommended dose.

In clinical trials trough levels of pyronaridine and artesunate in children were generally within the range observed in adults. Pyramax produces a uniform exposure across the weight ranges for the proposed labelled dosing of granules with no increased exposure seen in the younger patient range.

Absorption

Following administration of Pyramax tablets to healthy volunteers and patients with malaria, peak plasma concentrations are generally reached between 0.5 and 1.0 hours post-dose for artesunate, between 1 and 2 hours post-dose for DHA and between 2 and 8 hours post-dose for pyronaridine. Exposure to artesunate and pyronaridine was increased by 34% and 20% respectively when Pyramax was administered with a high fat meal, however these effects were not judged clinically significant and patients can take Pyramax tablets without regard to meals (see section 4.2).

Distribution

Pyronaridine and its metabolites are extensively distributed into tissues, with highest concentrations achieved in the liver, spleen, adrenal gland, kidney and thyroid gland in the rat. There is evidence that pyronaridine binds to melanin in the eye. In the dog, approximately 6% of a single dose of pyronaridine remained in the liver 24 months after administration. The potential extrapolation to human is not elucidated but the very slow elimination of pyronaridine-related material from the body means that accumulation, with possible hepatotoxicity, cannot be ruled out if pyronaridine is readministered too early.

Pyronaridine preferentially associates with blood cells, exhibiting a whole blood/plasma concentration ratio of approximately 1.5:1. Pyronaridine is highly bound to human serum proteins *in vitro* (92 to 95%). Pyronaridine displays two-compartment pharmacokinetic characteristics with a blood level profile that has a distinct distribution phase.

Artesunate and its metabolites are primarily associated in the rat with tissues involved in absorption and excretion and high levels were also found in the spleen.

Plasma protein binding of artesunate and DHA is moderate (62 to 93%) and albumin is the principal binding protein for DHA in human plasma.

Biotransformation

Pyronaridine appears to have a large number of potential metabolites, with no clear major metabolic route. Human *in vivo* metabolic profiling was conducted in blood, urine, and faecal samples from six healthy male volunteers in a microdose radioactivity mass balance study. Pyronaridine (unchanged) and a total of thirteen metabolites were identified in one or more sample matrices. Proposed metabolic pathways include: *N*-dearylation, oxidation, de-methylation, glucuronidation, cysteine conjugation, acetylation and reduction.

In vitro experiments indicate that CYP1A2, CYP2D6 and CYP3A4 could be involved in the metabolism of pyronaridine. *In vitro,* pyronaridine inhibits the activity of CYP2D6 at therapeutic plasma concentrations.

Artesunate is very rapidly metabolised by esterases to the active metabolite dihydroartemisinin (DHA). DHA is subsequently conjugated with glucuronic acid via UGT1A9 and UGT2B7.

Elimination

Pyronaridine is eliminated slowly from blood, with an elimination half-life in adults of between 14 and 18 days for parent compound, and a mean of 33.5 days for total drug-related material. The mean elimination half-life for paediatric malaria patients is 12.3 days. Urinary excretion of unchanged pyronaridine is <2% in healthy human subjects. Data from the mass balance study with pyronaridine in healthy volunteers indicates that faeces excretion is the main route of elimination of drug-related material. In this study, pyronaridine-related material was excreted both via faeces (47.8%) and urine (23.7%) after oral dosing of pyronaridine to healthy human subjects. Elimination occurred very slowly, the mean recovery of 71.5% (range 60.3%-82.2%) was achieved by 86 days after dosing.

In patients with uncomplicated malaria, artesunate and DHA are cleared from plasma with an elimination half-life of about 0.5 and 0.8 hours, respectively. No urinary excretion data are available for humans.

Hepatic and Renal Impairment

Pyramax has not been studied for efficacy and safety in patients with severe hepatic and/or severe renal impairment (see section 4.2).

Elderly Patients

Pyramax granules are intended for patients weighing less than 20 kg only.

5.3 Preclinical safety data

Repeat-dose toxicity studies with pyronaridine tetraphosphate:artesunate (3:1) in rats and dogs produced similar effects to those seen with each component individually.

The predominant feature in animals receiving repeated higher doses of pyronaridine tetraphosphate:artesunate (3:1) was related to the accumulation of pyronaridine.

Microscopically, after repeated dosing, this was seen as a widespread accumulation of basophilic material in many tissues and organs, sometimes present without associated inflammatory change (as for bone marrow and eye) but more often associated with dose-related inflammatory changes (as for liver, lung, spleen, gall bladder and kidney). It should be noted that, following a single 3-day cycle of treatment in dog, inflammatory changes were confined to liver and brain.

These inflammatory changes are considered secondary to the body's attempt to clear the accumulated material, and an increase in white blood cell count, predominantly in neutrophils and monocytes, is also considered a sequela of these changes. In more reactive tissues, notably rat liver, inflammatory and degenerate changes worsened over time in response to the prolonged presence of material, and this was correlated with increasing transaminase levels. This increase in severity was not evident following a single cycle of treatment.

Minimal to mild perivasculitis of the brain was noted in all repeat dose dog studies, including the single cycle study. This finding occurred with dose-related incidence, was not associated with relevant neurobehavioural changes and was not fully reversible.

Thymus atrophy was observed after administration of pyronaridine and artesunate to rats and dogs.

HERG studies were performed with pyronaridine, artesunate and dihydroartemisinin (DHA). Those studies showed that artesunate seldom had an effect on hERG tail current up to 300 µM (115.3 µg/mL) and that DHA and pyronaridine both inhibited hERG tail current with IC50s of 282.7 µM and 0.65 µM, respectively.

Pyronaridine was clastogenic in *in vitro* chromosome aberration tests and mouse lymphoma assays. The positive findings *in vitro* with mammalian cells are consistent likely related to the potential of pyronaridine for topoisomerase II inhibition. Pyronaridine was negative in the *in vivo* mouse bone marrow micronucleus test and rat liver *in vivo/in vitro* Unscheduled DNA Synthesis assay. In the rat liver comet assay negative results were obtained at liver concentrations 45-fold higher than the estimated liver concentrations reached in humans. Overall, the genotoxic risk associated with the proposed treatment cycle using pyronaridine should be no greater than that associated with other current therapies. Artesunate was not genotoxic in a standard package of genotoxicity assays. Carcinogenicity studies were not conducted since the treatment is limited to 3 days.

Neither pyronaridine tetraphosphate nor artesunate have effects on rat fertility. Artesunate is embryolethal at varying maternal dose levels and dosing regimens, depending on the nonclinical species. In fact, together with most other artemisinins (dihydroartemisinin, arteether, artemether) artesunate acts by depleting embryonic erythroblasts leading to severe anaemia. In cynomolgus monkeys, embryo lethality was observed in monkeys treated with artesunate for 30 days during the period of organogenesis, whereas no embryo lethality was observed in monkeys treated for a 3- or 7-day period during organogenesis, at comparable dose (which is 3 times the human dose based on mg/kg). In rats and rabbits, artesunate also caused embryolethality and foetotoxicity (decreased foetal body weight and increased skeletal and visceral variations).

Pyronaridine was shown to cross the placenta in rats. At maternally toxic doses, it caused early resorptions and abortions in rabbits, and decreased foetal body weight in rats and rabbits. There was no evidence of teratogenicity in both species.

6. PHARMACEUTICAL PARTICULARS

6.1 List of excipients

Pyramax granules for oral suspension

Artesunate
Pyronaridine tetraphosphate
Mannitol
Talc
Ethyl cellulose
Macrogol 6000
Hypromellose 2910
Tartrazine (E102)

Sunset Yellow FCF (E110)
Acesulfame potassium

6.2 Incompatibilities

Not applicable.

6.3 Shelf life

2 years.

6.4 Special precautions for storage

Do not store above 30°C.
Store in the original package.

6.5 Nature and contents of container

Pyramax granules for oral suspension

Sachets consisting of layers of polyester, aluminium and polyethylene/Surlyn, containing granules.
Each carton contains 90 sachets.

6.6 Special precautions for disposal

No special requirements.

Patients should be advised not to throw away any medicines via wastewater or household waste and ask their health provider how to dispose of unused medication.

7. SUPPLIER

Shin Poong Pharmaceutical Co., Ltd
161, Yeoksam-ro
Gangnam-gu
Seoul
South Korea

8. MARKETING AUTHORISATION NUMBER(S)

Not applicable.

9. DATE OF FIRST AUTHORISATION/RENEWAL OF THE AUTHORISATION

Not applicable.

10. DATE OF REVISION OF THE TEXT

Detailed information on this product is available on the website of the European Medicines Agency (EMA) http://www.ema.europa.eu

16.7.2 Artemether-lumefantrine

On the next pages, an example is given of a Summary of the product characteristics for Coartem® from Novartis, which is approved by a stringent regulator. Other WHO pre-qualified brands, such Lumartem from CIPLA may also be used.

Source of the summary of product characteristics for RiaMet/Coartem®: https://extranet.who.int/prequal/sites/default/files/documents/MA069part4v1.pdf

SUMMARY OF PRODUCT CHARACTERISTICS

(company authorized English translation of the “Fachinformation” approved by Swissmedic)

1. NAME OF THE MEDICINAL PRODUCT

Riamet Dispersible/ Coartem Dispersible 20 mg/120 mg dispersible tablets.

2. QUALITATIVE AND QUANTITATIVE COMPOSITION

Each dispersible tablet contains 20 mg artemether and 120 mg lumefantrine.

Excipients: Saccharin sodium, vanillin as flavouring agents, tableting excipients.

For a full list of excipients, see section 6.1.

3. PHARMACEUTICAL FORM

Dispersible tablets.

Yellow, round flat tablet with a beveled edge, debossed with “CD” on one side and “NVR” on the other side.

4. CLINICAL PARTICULARS

4.1 Therapeutic indications

This medicinal product is for pediatric use only.

Riamet Dispersible/ Coartem Dispersible is indicated for the treatment of children and infants with acute, uncomplicated infections due to *Plasmodium falciparum* or mixed infections including *P. falciparum*. Riamet Dispersible/ Coartem Dispersible may be used for self-administration, as stand-by emergency treatment in cases of suspected malarial infection, when no doctor can be reached within 24 hours or the medicinal product is not locally available.

Riamet Dispersible/ Coartem Dispersible is effective against both drug-sensitive and drug-resistant *P. falciparum*, and it is therefore also recommended for malaria infections acquired in areas where the parasites may be resistant to other antimalarials.

Consideration should be given to official guidelines and to local recommendations based on the prevalence of resistance to antimalarial agents. Official guidelines are those issued by the WHO and by health authorities.

4.2 Posology and method of administration

Riamet Dispersible/ Coartem Dispersible is not recommended for use in children below 5 kg body weight due to a lack of data on safety and efficacy.

Dispersible tablets for oral administration. The dispersible tablet(s) for one dose should be stirred in a small amount of water (approximately 10 ml per tablet) so that the active substance can be better dispersed before the suspension is drunk. Stir gently and administer immediately to the patient. Pour some more water (approximately 10 ml) into the glass and give immediately to the patient.

Food or drinks (such as milk) that are rich in fat should be consumed following ingestion of the dose even though patients with acute malaria are frequently averse to food. Patients should be encouraged to resume normal eating as soon as possible, since this improves absorption of artemether and lumefantrine.

In the event of vomiting within one hour of administration, a repeat dose should be taken. The dispersible tablet is indicated only for infants and children. A separate tablet formulation is available for adolescents and adults.

Treatment should be administered at the time of initial diagnosis or at the onset of symptoms.

Dosage for treatment and stand-by emergency treatment
A standard 3 day treatment schedule, with a total of 6 doses, is recommended as follows:

Dosage in infants and children ≤ 12 years of age weighing between 5 kg and < 35 kg
5 to < 15 kg body weight
One dispersible tablet at the time of initial diagnosis or as soon as symptoms appear, one dispersible tablet again after 8 hours and then one dispersible tablet twice daily (in the morning and evening) on each of the following two days (total course comprises 6 dispersible tablets).

15 to < 25 kg body weight
Two dispersible tablets as a single dose at the time of initial diagnosis or as soon as symptoms appear, two dispersible tablets again after 8 hours and then two dispersible tablets twice daily (in the morning and evening) on each of the following two days (total course comprises 12 dispersible tablets).

25 to < 35 kg body weight
Three dispersible tablets as a single dose at the time of initial diagnosis or as soon as symptoms appear, three dispersible tablets again after 8 hours and then three dispersible tablets twice daily (in the morning and evening) on each of the following two days (total course comprises 18 dispersible tablets).

Dosage in patients with impaired renal or hepatic function
No specific studies have been performed in these patient populations. No specific dose adjustment recommendations can be made for these patients (see sections 4.3 and 4.4).

Most patients with acute malaria present with some degree of hepatic impairment. In clinical trials the adverse event profile did not differ in patients with and those without hepatic impairment (see also section 4.4).

Moreover, baseline abnormalities in liver function tests improved in nearly all patients after treatment with Riamet.

New and recrudescent infections
Data for a limited number of patients show that new and recrudescent infections can be treated with a second course of Riamet Dispersible.

4.3 Contraindications

Hypersensitivity to the active substances or to any of the excipients.

Severe hepatic or renal impairment (see also section 4.4).

Patients with severe malaria according to the WHO definition.

First trimester of pregnancy in situations where other suitable and effective antimalarials are available (see also section 4.6).

Patients with a family history of congenital prolongation of the QTc interval or sudden death, or with any other clinical condition known to prolong the QTc interval, such as patients with a history of symptomatic cardiac arrhythmias, clinically relevant bradycardia or severe heart disease.

Patients taking drugs that prolong the QTc interval, such as class IA and III antiarrhythmics, neuroleptics, antidepressants, certain antibiotics (including some agents in the following classes: macrolides, fluoroquinolones, imidazoles and triazoles), antifungal agents, certain non-sedating antihistamines (terfenadine, astemizole) and cisapride.

Patients with known disturbances of electrolyte balance, e.g. hypokalaemia or hypomagnesaemia.

Patients taking drugs metabolized by cytochrome CYP2D6 (e.g. flecainide, metoprolol, imipramine, amitriptyline, clomipramine).

4.4 Special warnings and precautions for use

Riamet has not been evaluated for prophylaxis and is therefore not indicated for this use.

Riamet has not been investigated in the treatment of cerebral malaria or other severe manifestations of severe malaria, including pulmonary oedema or renal failure.

Severe malaria
In addition to the lack of clinical experience, use of Riamet in such cases is also inadvisable on pharmacokinetic grounds (the bioavailability of artemether and, in particular, of lumefantrine is uncertain in patients with high parasitaemia and little or no food intake).

Riamet has not been evaluated in, and is not indicated for, the treatment of malaria due to *P. vivax*, *P. malariae* or *P. ovale*, although some patients in clinical studies had co-infection with *P. falciparum* and *P. vivax* at baseline. Riamet is active against blood stages of *P. vivax*, but not against hypnozoites (= dormant form / dormant stage in hepatocytes).

Like other antimalarials (e.g. halofantrine, quinine, quinidine), Riamet may prolong the QTc interval, although no clinical adverse effect attributable to QTc prolongation (e.g. syncope, sudden death) has been reported (see section 5.1).

Riamet Dispersible/ Coartem Dispersible has not been studied for efficacy and safety in patients with severe hepatic or renal impairment, and therefore no recommendations can be made for these patient populations.
Patients who remain averse to food during treatment should be closely monitored. The risk of recurrence of disease may be greater.

If the patient's condition worsens during treatment with Riamet Dispersible, alternative antimalarial treatment should be started without delay. In such cases, ECG monitoring is recommended and steps should be taken to correct any electrolyte disturbances.

Following treatment of mixed infections including *P. vivax*, follow-up treatment must be given in order to eradicate the exoerythrocytic forms of *P. vivax*.

Caution is required if other medicinal products are given concomitantly.

Patients treated concomitantly with other antimalarials
Data on safety and efficacy are limited, and Riamet should therefore not be given concurrently with other antimalarials unless there is no other treatment option. The long elimination half-life of lumefantrine must be taken into account when administering quinine in patients previously treated with Riamet. The ECG should be closely monitored in this case, as well as when Riamet is administered following treatment with quinine, due to a possible additive prolongation of the QTc interval that has been observed in healthy subjects.

Patients previously treated with other antimalarials
Should Riamet be administered following treatment with mefloquine, it is particularly important to ensure that Riamet is taken together with food as lumefantrine levels may otherwise be insufficient.
In patients previously treated with halofantrine, Riamet should be administered no earlier than one month after the last halofantrine dose (see section 4.5).

Patients treated concomitantly with other medicinal products
Riamet Dispersible/ Coartem Dispersible should not be used concomitantly with drugs metabolized by CYP2D6 (see section 4.3). Additionally, caution is required when combining Riamet Dispersible/ Coartem Dispersible with substrates, inhibitors or inducers of CYP3A4 as the therapeutic effects of some drugs might be altered (see sections 4.5 and 5.2).

4.5 Interaction with other medicinal products and other forms of interaction

The mechanisms of the pharmacological and pharmacokinetic interactions are not all known.
Artemether and lumefantrine are substrates of CYP3A4. Administration of inducers or inhibitors of CYP3A4 may therefore lead to an increase or a reduction in exposure to lumefantrine and artemether.

Further interactions with CYP450 isoenzymes
Lumefantrine was found to inhibit CYP2D6 *in vitro*. This might be of particular clinical relevance for substances with a narrow therapeutic index. Co-administration of Riamet Dispersible/ Coartem Dispersible with drugs known to be metabolized by this isoenzyme (e.g. neuroleptics and tricyclic antidepressants) is contraindicated (see section 4.3).

Induction of CYP450 enzymes
Whereas *in vitro* studies with artemether at therapeutic concentrations revealed no significant inhibition with CYP450 enzymes, artemether and dihydroartemisinin (DHA) were reported to have a mild inducing effect on CYP3A4 activity. Although the changes have generally been slight and are unlikely to pose any problems in the general patient population, CYP3A4 induction might alter the therapeutic effects of drugs that are predominantly metabolized by this enzyme class.

Three specific pharmacokinetic and pharmacodynamic interaction studies with ketoconazole (a potent inhibitor of CYP3A4), mefloquine and quinine have been carried out in healthy volunteers.

Interactions with antimalarial drugs
Patients who are to receive Riamet Dispersible/ Coartem Dispersible may previously have been treated with other antimalarials. Interactions with mefloquine and quinine were therefore studied in healthy volunteers.
Sequential oral administration of mefloquine prior to Riamet Dispersible/ Coartem Dispersible had no effect on plasma concentrations of artemether or the artemether/dihydroartemisinin (DHA) ratio, but there was a significant (approximately 30–40%) reduction in plasma levels (C_{max} and AUC) of lumefantrine due to lower absorption, possibly secondary to a mefloquine-induced decrease in bile production.

Patients should be particularly advised to compensate for this decrease in bioavailability by eating something when taking Riamet Dispersible. As a rule, therefore, combined administration of Riamet Dispersible and mefloquine should be avoided.

In a drug interaction study in healthy subjects, administration of Riamet alone to 14 subjects had no effect on the QTc interval, while i.v. infusion of quinine alone in 14 other subjects caused a transient prolongation of the QTc interval, which was consistent with the known cardiotoxicity of quinine. This effect was slightly, but significantly, greater when quinine was infused after Riamet in 14 additional subjects. Prior administration of Riamet thus appears to increase the risk of QTc-prolongation associated with intravenous administration of quinine.

Concurrent intravenous administration of quinine (10 mg/kg body weight) with Riamet had no effect on plasma concentrations of lumefantrine or quinine. Plasma concentrations of artemether and DHA appear to be lower.

In a clinical study (carried out in Thailand), Riamet was given to some adult patients who had not responded to mefloquine or quinine. 121 patients received Riamet without any previous antimalarial treatment, whereas in 34 and 9 patients, respectively, blood levels of quinine or mefloquine were measurable at the start of the study. These patients showed safety and pharmacokinetic profiles for Riamet similar to those in patients who had no detectable levels of other antimalarials.

Interaction with a CYP450 3A4 inhibitor (ketoconazole)
Both artemether and lumefantrine are metabolized predominantly by CYP3A4, and at therapeutic concentrations do not inhibit this enzyme. In healthy adult subjects, concomitant oral administration of ketoconazole with Riamet leads to a modest increase (≤ 2-fold) in exposure: artemether (+130% in AUC and +116% in C_{max}), DHA (+51% and +37%, respectively), and lumefantrine (+61% and +28%, respectively). This increase in exposure to the antimalarial combination was not associated with increased adverse effects or changes in electrocardiographic parameters. Based on this study, dose adjustment of Riamet Dispersible/ Coartem Dispersible is not considered necessary in *P. falciparum* malaria patients given ketoconazole or other potent CYP3A4 inhibitors concomitantly.

Interaction with antiretroviral drugs
There have been no formal studies of interactions between Riamet and antiretroviral drugs.
Caution is required when using Riamet Dispersible/ Coartem Dispersible concomitantly with protease inhibitor antiretroviral drugs, especially fixed combinations thereof, due to variable patterns of inhibition, induction or competition for CYP3A4 with such drugs (see section 4.4 and 5.2).

4.6 Pregnancy and lactation

Pregnancy
There have been no controlled clinical studies of the safety of Riamet Dispersible/ Coartem Dispersible during pregnancy.
Data from animal studies suggest that Riamet Dispersible/ Coartem Dispersible may cause severe birth defects when administered during the first trimester of pregnancy (see sections 4.3 and 5.3).
In animals, reproductive toxicity studies with artemether have shown evidence of post-implantation losses and teratogenicity.

Other artemisinin derivatives have in addition demonstrated teratogenic potential, with increased risk during early gestation (see section 5.3).

Riamet Dispersible/ Coartem Dispersible is contraindicated during the first trimester of pregnancy if other effective antimalarials are available. However, it should not be withheld in life-threatening situations where no other effective antimalarials are available (see section 4.3).

During the second and the third trimesters, treatment should only be given if absolutely necessary.

Women of childbearing potential
Riamet Dispersible/ Coartem Dispersible is contraindicated during the first trimester of pregnancy, and women therefore should not conceive while undergoing malaria treatment with Riamet Dispersible. This includes women who are travelling, for whom Riamet Dispersible/ Coartem Dispersible has been prescribed as stand-by emergency treatment of malaria, should such treatment be required.

Women of childbearing potential undergoing treatment with Riamet Dispersible, including stand-by emergency treatment during travel, should be advised to practice contraception until the start of the next menstruation following the end of treatment.

Lactation
Animal data suggest that Riamet Dispersible/ Coartem Dispersible passes into the breast milk but no data are available in humans. Women who are breastfeeding should not take Riamet Dispersible. Due to the long elimination half-life of lumefantrine (4 to 6 days), it is recommended that breastfeeding should not resume before day 28 unless the potential benefits to both mother and child outweigh the risks of treatment with Riamet Dispersible.

4.7 Effects on ability to drive and use machines

Riamet Dispersible/ Coartem Dispersible has moderate influence on the ability to drive and use machines.

Patients receiving Riamet Dispersible/ Coartem Dispersible should be warned that dizziness, fatigue or asthenia may occur, in which case their ability to drive or use machines may be impaired.

4.8 Undesirable effects

Most of the reported events were mild to moderate in severity and of short to moderate duration. They were probably related more to the underlying malaria and/or to an inadequate response to treatment rather than to Riamet treatment, although a causal relationship with Riamet cannot be ruled out in some of the reported cases. In other reports, other factors (e.g. concomitant drug therapy, concurrent infections) were presumed to be the more likely cause of the events, or the available information was too meagre to allow any conclusions to be drawn.

Frequencies are defined as very common (≥1/10), common (≥1/100, <1/10), uncommon (≥1/1,000, <1/100), rare (≥1/10,000 to <1/1,000), very rare (<1/10,000), not known (cannot be estimated from the available data). Within each frequency grouping, undesirable effects are presented in order of decreasing seriousness.

Table 1 Adverse reactions reported in patients

**Immune system disorders**	
Rare	Hypersensitivity reactions
**Metabolism and nutrition disorders**	
Very common	Loss of appetite (10.8%)
**Psychiatric disorders**	
Uncommon	Sleep disturbances
**Nervous system disorders**	
Common	Headache
Common	Dizziness
Uncommon	Drowsiness
**Cardiac disorders**	
Common	QT interval prolonged in electrocardiogram (including QTc prolongations >60 msec and/or absolute QTc intervals >500 msec)
Uncommon	Palpitations
**Respiratory, thoracic and mediastinal disorders**	
Very common	Cough (23.5%)
**Gastrointestinal disorders**	
Very common	Vomiting (17.5%)
Common	Abdominal pain
Common	Diarrhoea
Common	Nausea
**Hepatobiliary disorders**	
Common	Elevated liver function values
**Skin and subcutaneous tissue disorders**	
Common	Rash
Uncommon	Pruritus
**Musculoskeletal and connective tissue disorders**	
Common	Arthralgia, myalgia
**General disorders and administration site conditions**	
Common	Asthenia, fatigue

In this pooled safety analysis, mood swings were reported in fewer than 1.2% of the paediatric patients treated with Riamet, but they were not considered drug-related by the investigators.

Adverse effects found in non-recommended regimens not included in this pooled safety analysis are: paraesthesia (1.2% of adolescents and adults, no cases in children); involuntary muscle contractions (1.3% of children); non-specific personality disorders which have been reported in 1.1% of children under 5 years of age who were treated with Riamet during clinical studies. This incidence is 2–3 times lower than that observed in children of the same age who were treated with the reference antimalarials used in these studies (mefloquine/artesunate, quinine or sulphadoxine/pyrimethamine).

There were uncommon reports of the following adverse effects in adults, but not in infants or children: hypoaesthesia, ataxia, and abnormal gait.

4.9 Overdose

No case of overdose has been reported.

If overdosage is suspected, symptomatic and supportive therapy should be initiated based on the clinical picture. The ECG and electrolytes (e.g. potassium) should be monitored.

5. PHARMACOLOGICAL PROPERTIES

5.1 Pharmacodynamic properties

Pharmacotherapeutic group: Artemisinin and derivatives, ATC code: P01BE52.

Riamet Dispersible/ Coartem Dispersible contains a fixed combination of artemether and lumefantrine, in the ratio of 1:6, which acts as an antimalarial agent against schizonts. Artemether is a semisynthetic chiral acetal derivative of artemisinin isolated from the plant *Artemisia annua*. Lumefantrine is a racemic mixture of a synthetic fluorene derivative. Like other antimalarials (quinine, mefloquine, halofantrine), lumefantrine belongs to the aryl-amino-alcohol family.

The site of antiparasitic action of both components is the food vacuole of the malaria parasite. Lumefantrine is thought to interfere with the polymerization process that brings about the conversion of haem, a toxic intermediate produced during haemoglobin breakdown, to the non-toxic haemozoin, malaria pigment. Artemether, on the other hand, may generate toxic, reactive metabolites as a result of the interaction between its endoperoxide bridge and haem iron. Both artemether and lumefantrine have a secondary action involving inhibition of nucleic acid and protein synthesis.

To date, data from *in vitro* and *in vivo* studies show that Riamet has not induced resistance.

The efficacy of the combination of lumefantrine and artemether in Riamet is greater than that of either substance alone. In a double-blind, comparative study in adults in China (n = 157), the cure rate for Riamet – given in 4 doses over a 28 day period – was 94%; it was 90% for lumefantrine monotherapy and 46% for artemether monotherapy (based on the intent-to-treat [ITT] population). For the evaluable population, the 28 day cure rates were 100% for Riamet, compared with 92% for lumefantrine monotherapy and 55% for artemether monotherapy.

In the resident population of areas where multi-drug-resistant strains of *P. falciparum* malaria are common, 28 day cure rates with the six-dose regimen (given over 60 or 96 hours) were 81% and 90% for Riamet versus 94% and 96% for mefloquine/artesunate (based on the ITT population). For the evaluable population, the 28 day cure rates were 97% and 95% for Riamet and 100% for mefloquine/artesunate.

In 319 adult patients in whom gametocytes were present, the average time to gametocyte clearance with Riamet was 96 hours. Riamet showed more rapid gametocyte clearance than any comparator except mefloquine/artesunate. Riamet is active against blood stages of *P. vivax*, but not against hypnozoites.

A similar efficacy and safety profile was shown in non-immune adult patients living in regions free of malaria but with malaria acquired when travelling in endemic regions. In an open-label study in adults (n = 165), the 28 day cure rate for Riamet given in the 6 dose regimen was 96% (119/124) in the evaluable population and 74.1% (120/162) in the ITT population. The difference between evaluable and ITT population cure rates was due to 38 patients who were excluded from the evaluable population for the following reasons: 33 patients were lost to follow up, of whom 19 had no evaluation and 14 had parasitic clearance at day 7 (but unknown efficacy status at day 28); 5 patients took concomitant medications that were not permitted by the protocol. All these patients were considered as treatment failures in the ITT analysis.

Efficacy data in infants and children
In a randomized, investigator-blinded, multicentre trial in sub-Saharan Africa comparing the efficacy of 6 dose Riamet Dispersible/ Coartem Dispersible dispersible tablets and (crushed) Riamet administered according to body weight in 899 children 12 years of age or younger with between 5 kg and 35 kg body weight, the 28 day parasitological (PCR-corrected) cure rate was 97.8% and 98.5%, respectively, in the primary analysis population and 95% and 96.2%, respectively, in the ITT population.

The mean 28 day parasitological (polymerase-chain-reaction [PCR]-corrected) cure rate was 93.9% in the ITT population and 96.7% in the evaluable population in an open, multicentre clinical study conducted in Africa in 310 children, weighing between 5 kg and 25 kg, who received a 6 dose Riamet regimen that varied according to body weight.

Children from non-endemic countries were not included in the clinical trials.

QT/QTc prolongation
The administration of the six dose regimen of Riamet was associated with QTcF prolongation in a parallel study in healthy adults that included placebo and moxifloxacin control groups (n = 42 per group). The mean change from baseline at 68, 72, 96, and 108 hours after the first dose were 7.45, 7.29, 6.12 and 6.84 milliseconds, respectively. The change from baseline QTcF was zero at 156 and 168 hours after the first dose. No subject had an increase from baseline > 30 milliseconds, nor an absolute value > 500 milliseconds. As compared with the placebo group, the moxifloxacin control was associated with a QTcF prolongation for 12 hours after the single dose, with the maximum change 1 hour after the dose amounting to 14.1 milliseconds.

QTcB prolongation > 500 milliseconds was reported in one patient (0.1%) in clinical trials in children. No patient had a QTcF interval > 500 milliseconds. In clinical studies in adults, QTcB prolongation > 500 milliseconds was reported in 0.9% of patients and QTcF prolongation > 500 milliseconds was reported in 0.3% of patients.

There have been no reports of clinical adverse effects attributable to QTc prolongation (e.g. syncope, sudden death).

5.2 Pharmacokinetic properties

Pharmacokinetic characterization of Riamet is limited by the lack of an intravenous formulation, and the very high inter- and intrasubject variability of artemether and lumefantrine plasma concentrations and derived pharmacokinetic parameters (AUC, C_{max}).

Absorption
Artemether is absorbed fairly rapidly, with peak plasma concentrations attained approx. 2 hours after administration. Absorption of lumefantrine, a highly lipophilic compound, starts after a lag-time of up to 2 hours, with peak plasma concentration about 6–8 hours after administration. Food enhances the absorption of both artemether and lumefantrine: In healthy volunteers given a high-fat meal, the relative bioavailability of artemether was increased more than two-fold, and that of lumefantrine sixteen-fold compared with fasted conditions. Food has also been shown to increase the absorption of lumefantrine in patients with malaria, although to a lesser extent (approximately two-fold), most probably due to the lower fat content of the food ingested by acutely ill patients. Food interaction data indicate that absorption of lumefantrine under fasted conditions is very poor (probably less than 10% of the dose). Patients should therefore be strongly encouraged to take the medication with a normal diet as soon as food can be tolerated.
In healthy (adult) volunteers, systemic exposure to artemether, its metabolite dihydroartemisinin (DHA) and lumefantrine was similar with dispersible and crushed tablets (see table 2).

Table 2: Pharmacokinetic parameters following a single dose (4 tablets) – containing 80 mg artemether / 480 mg lumefantrine – administered as either dispersible or crushed tablets

	Dispersible tablets	Crushed tablets
	Artemether	
	(n = 54)	(n = 50)
C_{max} (ng/ml)	73.3 ± 39.5	67.4 ± 35.5
t_{max} (hours)	2.02 [0.50-4.02]	2.05 [0.52-4.07]
AUC_{last} (ng×hours/ml)	263 ± 142	229 ± 136
	DHA	
	(n = 54)	(n = 50)
C_{max} (ng/ml)	48.6 ± 23.2	48.8 ± 26.0
t_{max} (hours)	2.98 [0.75-5.98]	2.54 [0.75-4.07]
AUC_{last} (ng×hours/ml)	171 ± 59.5	160 ± 68.0
	Lumefantrine	
	(n = 55)	(n = 52)
C_{max} (µg/ml)	10.2 ± 3.08	10.0 ± 2.57
t_{max} (hours)	8.00 [4.98-24.02]	8.00 [4.98-24.02]
AUC_{last} (µg×hours/ml)	295 ± 107	280 ± 93.2

Table shows mean ± standard deviation for C_{max} und AUC_{last}, median values and [min-max] ranges for t_{max}.

Distribution
Artemether and lumefantrine are both highly bound to human serum proteins *in vitro* (95.4% and 99.7%, respectively).
DHA is also bound to human serum proteins (47%–76%). Protein binding to human plasma protein is linear.

Biotransformation
Artemether is rapidly and extensively metabolized (substantial first-pass metabolism). *In vitro* data show that human liver microsomes metabolize artemether to the biologically active main metabolite DHA (demethylation), predominantly by way of CYP3A4/5.

The pharmacokinetics of this metabolite have also been described in humans *in vivo*.

The artemether/DHA AUC ratio is 1.2 after a single dose and 0.3 after the last of 6 doses given over 3 days. Artemether and DHA were reported to have a mild inducing effect on CYP3A4 activity that is not expected to pose a problem in the general patient population.

Plasma levels of artemether decreased markedly during repeated administration of Riamet, while levels of the active metabolite (DHA) increased, although not to a statistically significant degree. This confirms that there was induction of the enzyme responsible for the metabolism of artemether. The clinical evidence of induction is consistent with the *in vitro* data in the section 4.5.

In vitro, lumefantrine is N-debutylated, mainly by CYP3A4, in human liver microsomes. *In vivo* in animals (dogs and rats), glucuronidation of lumefantrine takes place directly and after oxidative biotransformation. In humans, systemic exposure to the desbutyl-lumefantrine metabolite – which has an *in vitro* antiparasitic effect 5 to 8 times higher than that of lumefantrine – amounted to less than 1% of the exposure to the parent compound.

In vitro, therapeutic plasma concentrations of lumefantrine significantly inhibit the activity of CYP2D6 (see sections 4.4, 4.5 and 4.3).

Elimination
Artemether and DHA are rapidly cleared from plasma with an elimination half-life of about 2 hours. Lumefantrine is eliminated very slowly, with a terminal half-life of 2–3 days in healthy volunteers and 4–6 days in patients with falciparum malaria. Demographic characteristics such as sex and weight appear to have no clinically relevant effects on the pharmacokinetics of Riamet.

No data are available on urinary excretion of artemether and lumefantrine in humans. In rats and dogs, unchanged artemether has not been detected in the faeces and the urine due to its rapid and high first-pass metabolism, but numerous metabolites (identified in part) have been detected in the faeces, the bile and the urine. Lumefantrine is eliminated into the bile in rats and dogs, with excretion primarily in the faeces. Metabolites (glucuronides of lumefantrine and of the desbutyl metabolite) were eliminated into the bile following oral administration in rats and dogs. Most of the dose was recovered in the faeces in the form of parent drug (this included unabsorbed drug components and drug components released from glucuronides).

Pharmacokinetics in special patient populations
No specific pharmacokinetic studies have been performed in patients with hepatic or renal impairment. Systemic exposure to artemether, DHA, and lumefantrine in paediatric malaria patients (≥ 5 to < 35 kg body weight) dosed on a mg/kg body weight basis is comparable to that measured in adult malaria patients on the recommended dosing regimen.

5.3 Preclinical safety data

Mutagenicity
There have been no reports of mutagenicity in *in vitro* and *in vivo* tests with an artemether:lumefantrine combination consisting of 1 part artemether : 6 parts lumefantrine. In the micronucleus test, myelotoxicity was seen at all dose levels (500, 1000 and 2000 mg/kg), but recovery was reported to be almost complete 48 hours after dosing.

Carcinogenicity
Due to the short period of treatment, carcinogenicity studies with the artemether:lumefantrine combination were not carried out.

Reproductive toxicity
Reproductive toxicity studies in rats given oral doses of the artemether:lumefantrine combination showed maternal toxicity and increased post-implantation loss at doses ≥ 50 mg/kg (corresponding to approximately 7 mg/kg artemether). The artemether:lumefantrine combination was not embryotoxic in rats at a dose of 25 mg/kg (corresponding to approximately 3.6 mg/kg artemether). Following oral administration of the artemether:lumefantrine combination in rabbits, maternal toxicity and increased post-implantation loss were seen at a dose of 175 mg/kg (corresponding to 25 mg/kg artemether), while the next lowest dose level of 105 mg/kg (corresponding to 15 mg/kg artemether) was free of treatment-induced effects.

Artemisinins are known to be embryotoxic in animals. Reproductive toxicity studies with artemisinin derivatives demonstrated increased post-implantation loss and teratogenicity (a low incidence of cardiovascular and skeletal malformations) in rats at a dose of 6 mg/kg artesunate and 19.4 mg/kg artemether. In rats, 3 mg/kg artemether was established as the non-toxic dose.

In rabbits, artemether produced maternal toxicity and an increase in post-implantation loss at a dose of 30 mg/kg, but no maternal toxicity, embryotoxicity or fetotoxicity at doses up to 25 mg/kg. The artemisinin derivative artesunate produced a low incidence of cardiovascular and skeletal malformations in rabbits at 5 mg/kg, the lowest dose used.

The embryotoxic artemether dose, 20 mg/kg/day in the rat, yields artemether and DHA exposures similar to those in humans.

Cardiovascular pharmacology

In toxicity studies in dogs, there was some evidence of QTc prolongation at doses higher than the therapeutic doses used in man (≥ 600 mg/kg/day). In an *in vitro* assay of HERG channels stably expressed in an HEK293 cell line, lumefantrine and the main metabolite desbutyl-lumefantrine showed some inhibitory potential on one of the ion channels responsible for cardiac repolarization. However, this potency was lower than that of the other antimalarial drugs tested. From the estimated IC_{50} values, the order of potency of HERG current block was: halofantrine (IC_{50} = 0.04 micromolar) > chloroquine (2.5 micromolar) > mefloquine (2.6 micromolar) > desbutyl-lumefantrine (5.5 micromolar) > lumefantrine (8.1 micromolar). A study in healthy adults shows that the QTcF interval may be prolonged by standard dosing of Riamet (see sections 4.3, 4.4 and 5.1).

6. PHARMACEUTICAL PARTICULARS

6.1 List of excipients

Microcrystalline cellulose
Hypromellose
Colloidal silicon dioxide
Polysorbate 80
Crospovidone
Cherry dry flavour
Saccharin sodium
Magnesium stearate

6.2 Incompatibilities

Not applicable.

6.3 Shelf life

2 years.

6.4 Special precautions for storage

Do not store above 30°C. Protect from heat and moisture, and store in the original packaging.
Keep out of the reach of children.

The physician or pharmacist should show parents or caregivers how to use the product in paediatric patients, and should inform them that a variable number of tablets (depending on the child's body weight) will be required for complete treatment. Depending on the required number of tablets, it may not be necessary to use the whole pack. Any extra tablets should be properly disposed of, or returned to a pharmacy, once treatment has been successfully completed.

6.5 Nature and contents of container

The tablets are packed in ALU and laminated transparent PVC/PE/PVDC blisters.
The blisters are glued into a carton wallet that protects the blisters from being destroyed. The wallets containing the blisters are then packed into a functional dispenser carton box.

Packs containing 30 blister strips x 6 dispersible tablets,
30 blister strips x 12 dispersible tablets or
30 blister strips x 18 dispersible tablets.

6.6 Special precautions for disposal

No special requirements.

7. MARKETING AUTHORISATION HOLDER

Novartis Pharma AG
Lichtstrasse 35,
CH-4002 Basel
Switzerland

8. MARKETING AUTHORISATION NUMBER(S):

58528 (Swissmedic)

9. DATE OF FIRST AUTHORISATION/RENEWAL OF AUTHORISATION:

10. DATE OF REVISION OF THE TEXT

10/2008

16.8 Appendix VIII. Budget and budget justification

16.8.1 Budget

Summary budget: Item Description	Kenya (USD)	Burkina Faso (USD)	Total cost (USD)
Personnel	548,037	500,189	1,048,226
Equipment			
Travel	74,656	36,700	111,356
Other Direct Costs	186,117	245,670	431,787
Consultants	$		-
**Total Direct Costs**	**808,810**	**782,559**	**1,591,369**
IDCs 15%	121,322	117,384	238,705
**TOTAL COSTS**	**930,132**	**899,943**	**1,830,074**
Total costs in local currency	KES 93,013,150	XOF 494,968,568	NA

Detailed budget: Item Description	Kenya (USD)	Burkina Faso (USD)	Total cost (USD)
(a) Personnel, salaries and benefits disbursement			-
i. Personnel salaries	499,037	464,189	963,226
ii. Local health staff compensation	49,000	36,000	85,000
(b) Patient costs, travel, food and/or supplies			-
i. Participants ancillary care	44,250	53,100	97,350
ii. Participants compensation	23,076	63,720	86,796
iii. Staff training & community engagement	7,330	11,000	18,330
(c) Major equipment itemized; minor aggregated	-		-
(d) Supplies			-
i. Material for data collection (Laptops, Tablets, etc.)	3,150	15,000	18,150
ii. File storage cabinets	21,540	5,000	26,540
iii. Personal Protection equipment & Lab supplies	132,686	72,850	205,536
(e) Travel and accommodation:			-
i. Local or field travel	3,136	36,700	39,836
ii International/Local conferences			-
(f) Transportation, vehicle repairs, insurance, etc.			-
i) vehicle and transportation costs, insurance, etc	17,730		17,730
(g) Operating expenses, postage, printing, etc.			-
i. Communication fees	4,575	20,000	24,575
ii. Ethical submission fees	3,300	5,000	8,300
(h) Animals: acquisition, food, cages, etc.	-		-
(i) Consultancy fees	-		-
(j) Contingency funds (15% including inflation)			-
**Total Direct Costs**	**808,810**	**782,559**	**1,591,369**
(k) Institutional administrative overheads: 15%	121,322	117,384	238,705
**TOTAL COSTS**	**930,132**	**899,943**	**1,830,074**
Total costs in local currency	KES 93,013,150	XOF 494,968,568	NA

16.8.2 Budget Justification

Funding has been approved for fieldwork starting in August 2020 for a period of approximately 18 months. The above budget estimates and specific costs will be subject to minimal variation. Centralised operating costs for running the project, plus costs for any assays in overseas laboratories are included in the budgets of the overseas partners and include funds for central sponsorship support, trial insurance and trial monitoring, a central safety register, data management support and for trial coordination, international travel and academic capacity training.

16.9 Appendix IX. Description of other clinical and laboratory methods

This section 'Appendix IX. Description of other clinical and laboratory methods' of the appendix, describes the clinical and laboratory-based assays for some endpoints conducted in subgroups of participants in specific study sites or otherwise in a random sample of participants in all sites. The volume and timing of these samples are described in the main text under section 2.2.4, Schedule of activities, page 19 and in section 9, Participants timeline and study encounters, page 40 onwards. The purpose of this appendix text is to describe the assays and measures of the procedures in more detail.

16.9.1 Novel diagnostics for COVID-19

Collaborating institutions and contact persons: Dr Emily Adams, LSTM, Pembroke Place, Liverpool L3 5QA, UK. Dr David Allen, Dr Tegwen Marlais, LSHTM, Keppel St, Bloomsbury, London WC1E 7HT, UK.

Objective: To determine the diagnostic accuracy of novel molecular diagnostics, point of care antigen tests and rapid diagnostics for antibody response for COVID-19.

Rationale: Swabbing of the nasopharynx, RNA extraction and qPCR is the standard diagnostic for COVID-19. This is a complex process, requiring invasive sampling, extensive laboratory infrastructure, expertise, and expensive consumables with limited availability during this pandemic. In most countries, there is a diagnostic delay of 2-3 days due to this complexity.

We propose to evaluate three different diagnostics approaches for COVID-19:

- The novel molecular test LAMP (by Optigene) which will use a non-invasive saliva sample for rapid molecular diagnosis with and without RNA extraction. Saliva has been shown to be a non-invasive sample type, that can be self-collected, and has a viral load of approximately 90% as compared with nasopharyngeal swabs. LAMP is quick (20 minutes), simple and performed directly on protocols are currently being optimised for working directly with a saliva sample.
- Rapid diagnostic antigen tests: SD-BioSensor Standard Q COVID-19 Ag Test is a rapid chromatographic immunoassay for the qualitative detection of specific antigens to SARS-CoV-2 present in human nasopharynx (30 min), and DiaTropix/Mologic antigen tests: DiaTropix/Mologic have partnered with Biosure to deliver a rapid, lateral flow-based antigen test on saliva samples. This test will take 10 minutes and directly detect spike proteins on the surface of the virus, alongside the N protein with lysis on the lateral flow device. Other antigen tests may also be considered when they become available.
- Rapid diagnostic antibody tests such as thepid Diagnostic Tests by DiaTropix/Mologic for early antibody response. Data collected in London (St Georges Hospital) and at LSTM, Liverpool indicates that antibody response to SARS-CoV-2 rise rapidly from symptom onset and can be indicative of early infection in moderately severe patients.

If these tests perform with high accuracy (WHO Target Product Profile [TPP] to be published in July) in sub-Saharan Africa, they could be implemented for diagnostic purposes, to speed time to diagnosis for triage, treatment, isolation and in clinical trials to speed time to recruitment. For this study, all these tests will be considered experimental and will not be used for patient care but may trigger expedited confirmation by diagnostic tests that are approved in each country, such as qPCR. This may change during the study when more tests are approved in each country.

Sample size: Approximately, 1000 suspected or confirmed COVID-19 cases from the screening study (cross-sectional survey, source population) and COVID-19 cohort study.

Methods:

Molecular diagnostics: Saliva samples (250 µL minimum), will be collected and half extracted for RNA, the other half to be used directly on the LAMP reaction. Time to results is 20 minutes using this direct method of amplification.

Antigen testing: Direct sampling of saliva from the patient's mouth with a Biosure/Mologic antigen test (like HIV self-test) device. The device will sample approximately 100 µL, and results read on a lateral flow device within 15 minutes.

Antibody testing at the point of contact: 10ul of serum will be applied to the test pad and results read after 10 minutes for IgA, IgM and IgG.

16.9.2 Kinetics of SARS-CoV-2 and malaria antibodies

Collaborating institutions and contact persons: Dr Tegwen Marlais, Dr Kevin Tetteh, LSHTM, Keppel St, Bloomsbury, London WC1E 7HT, UK Dr Emily Adams, LSTM, Pembroke Place, Liverpool L3 5QA, UK.

Objective: To determine the antibody profiles to SARS-CoV-2 and malaria-specific antigens in COVID19 infected individuals with and without malaria.

Rationale: Antibody responses to SARS-CoV-2 are implicated as a key part of the protective immune response. Understanding if and how they are affected by other infections such as malaria is vital in predicting disease severity and future spread of infection.

We propose to evaluate antibody responses to SARS-CoV-2 and malaria using multiplex bead-based assays. Assays will also describe the type (IgG, M and A) and strength (avidity) of antibody response. Assays will be conducted in paired serum and saliva to investigate the utility of saliva as a non-invasive sample source for future studies. These assays will also provide comparator metrics for diagnostic evaluation described above.

Sample size: All 708 confirmed COVID-19 cases from the COVID-19 cohort study and a subset of samples from the screening study (cross-sectional survey, source population) (~500).

Methods: Serum samples (100ul) and, where available, saliva samples (50 µL minimum), will be incubated with microsphere beads coated with ~ 5 SARS-CoV-2 antigens including spike protein and nuecleocapsid proteins and two seasonal coronavirus proteins and ~10 malaria antigens including those associated with longterm exposure (PfAMA-1, PfMSP-1 and PfGLURP) and short term exposure (Pf ETRAMP5, PfGEXP1, PfHSP40).[54] After washing samples are incubated with secondary antibody detecting IgG, M or A and responses detected by reading on a Luminex MAGPix and recorded as mean fluorescence index. In a subset of samples with avidity will be assessed by preincubation with Guanidine Isothiocyanate (GuICN) as an anti-chelation agent and the ratio of MFI with and without GuICN calculated as an avidity index.

16.9.3 Evaluation of immune biomarkers

Collaborating institutions and contact persons: Dr Tegwen Marlais, Prof Chris Drakeley, Dr Kevin Tetteh & Dr Chrissy Roberts, LSHTM, Keppel St, Bloomsbury, London WC1E 7HT, UK.

Objective: To determine the association of immune and endothelial markers with severity of disease outcome in COVID-19 patients, with and without malaria and other known co-infections.

Rationale: Markers of immune and endothelial activation are released into the bloodstream during immune responses. These include cytokines and other proteins involved in both immune activation and endothelial integrity. Some of these markers are raised in response to particular aetiologies, i.e.

bacterial, viral or parasitic, for example, procalcitonin (PCT) and myxovirus resistance protein A (MxA). Others are indicative of a poor outcome due to sepsis, e.g. angiopoietin-2 and IL-6. In contrast, some markers are protective; these include angiopoietin-1, which maintains vascular integrity and regulatory cytokine IL-10.

By measuring these markers in the cohort of COVID-19 patients, both with and without malaria co-infection, we will reveal whether any marker or combination of markers can differentiate patients that go on to have mild disease from those requiring medical intervention or suffering from severe disease. We will also investigate the effect of malaria treatment on the immune response to SARS-CoV-2.

Such knowledge could inform the future development and deployment of a rapid diagnostic test to detect relevant markers that could indicate COVID-19 risk in the early stages of infection before an individual becomes critically ill.

Sample size: All confirmed COVID-19 cases from the COVID-19 cohort study.

Methods: A multiplex bead-based immunoassay, in advanced stages of development at LSHTM, will be used to measure up to 15 different markers in dried blood spot (DBS), serum or plasma samples. Samples are incubated with microsphere beads coated with antibodies against the panel of markers. After washing, a mixture of secondary antibodies against each of the markers is added and incubated further to bind to any captured markers. Finally, a fluorescent label is added, which binds to the secondary antibody. Median fluorescence intensity is measured and indicates the level of each marker in each sample.

The assay is currently developed to use blood eluted from DBS, but through the use of paired DBS, serum and plasma samples in this study, assay performance can be assessed and adapted to use all sample types, making it more widely applicable as a research tool.

16.9.4 Immunological CyTOF B/Tcell assays & and OLINK screen

Collaborating institutions and contact persons: Dr Gerlinde Obermoser and Prof Holden Maecker, The Maecker laboratory, Department of Microbiology and Immunology, The Global Health Vaccine Accelerator Platform (GH-VAP) program, Systems Biology/Systems Immunology Consortium, Fairchild Science Building, 299 Campus Drive, Stanford University, California 94305-5124, USA.

Objective: To determine differences in cell phenotypes and protein markers in individuals with COVID19 with and without malaria, and healthy controls with no active COVID19 or malaria, and if vaccinated, at least 4 weeks previously against COVID19.

Rationale: Cellular activation can be profound in both COVID19 and malaria infections, leading to specific expansion or downregulation of cell populations which can have major impacts on disease progression and resolution. New technology such as CYTOF and OLINK allows simultaneous assessment of multiple cellular and protein products from small quantities of whole blood. Recent studies using CYTOF analysis from COVID19 patients described specific downregulation and immunosuppression of cellular responses, specifically those associated with CDC45+ T cells.[55] Analysis of cellular response to malaria with CYTOF has shown effects on B cell populations, specifically CD11c B cell subsets.[56] This study provides a unique opportunity to examine the effect of co-infection on B and T cell profiles and relate these with downstream protein biomarkers which have been shown to be affected by COVID19 infection.[57]

Sample size: Approximately 180 patients and 100 healthy controls

Methods:

Whole Blood CyTOF Assay of Immune Phenotypes: A small volume (270 µL) of whole blood to which a stabilizing fixative (PROT1, Smart Tube Inc., San Carlos, CA, USA) is added, to perform single-cell Mass Cytometry (CyTOF) to determine cell phenotypes and activation states in a highly comprehensive fashion, with up to 40 antibodies per sample. The Maecker lab has recently developed a CyTOF panel compatible with such fixed whole blood samples and containing a number of markers of specific interest in COVID-19 research (see Table 4: CyTOF panel).

Fixed whole blood samples will be shipped on dry ice to Stanford, where they will be batched for CyTOF runs. For each batch run, samples will be thawed, washed with Lyse-Wash buffer (Smart Tube), barcoded using Pd sample barcodes (Fluidigm), and stained for cell-surface and then intracellular epitopes. They will then be treated with Ir-labeled DNA intercalator, fixed, and acquired on a Helios CyTOF instrument. At least 250,000 cell events per sample will be targeted for collection. The resulting FCS files will be normalized for intensity using EQ bead readings, de-barcoded, and analysed via both manual gating and automated methods such as FlowSOM or Citrus.

Table 4: CyTOF panel

89Y	CD66b	HIMC
113Id	CD57	HIMC
141Pr	HLA-DR	HIMC
142Nd	CD19	DVS
143Nd	CD66a/c/e	HIMC
144Nd	CD31	DVS
145Nd	CD4	DVS
146Nd	BPI	HIMC
147Sm	CD20	DVS
148Nd	CD41	HIMC
149Sm	CD11c	HIMC
150Nd	CD123	HIMC
151Eu	CD107a	DVS
152Sm	Siglec-8	HIMC
153Eu	CD45RA	DVS
154Sm	CD3	DVS
155Gd	CD45	HIMC
156Gd	CD38	HIMC
157Gd	CD25	HIMC
158Gd	CD33	HIMC
159Tb	GMCSF	DVS
160Gd	CD14	DVS
161Dy	Ki-67	DVS
162Dy	CD69	DVS
163Dy	TCRgd	HIMC
164Dy	Arginase -1	DVS
165Ho	CD127	DVS
166Er	MS4A3	HIMC
167Er	CD27	DVS
168Er	CD8	DVS
169Tm	STOM	HIMC
170Er	PD1	HIMC
171Yb	Granzyme B	DVS
172Yb	CD15	DVS
173Yb	Perforin	HIMC
174Yb	CD21	HIMC
175Lu	IgD	HIMC
176Yb	CD56	DVS
209Bi	CD16	DVS

Olink Immunoassays: Olink (Upsala, Sweden) has developed standard immunoassay panels of 92 analytes each, using proximity extension assay technology to provide highly specific and sensitive readouts by qPCR on the Fluidigm Biomark system. Serum samples are incubated with capture antibody cocktail for the Olink Inflammation Panel (https://www.olink.com/products/inflammation/) and subjected to qPCR according to the manufacturer's protocol. They are transferred to a 96.96 Fluidigm array and read in the Biomark instrument. NPX values (indicative of cytokine level) are calculated by the Olink software and normalized across batches. Group-level comparisons will then be made with the normalized NPX values using univariate and multivariate regression and other possible analytical techniques.

16.9.5 Non-malaria serology and detection of other infection targets

Collaborating institutions and contact persons: *Dr Aaron Samuels, CDC-Kenya, Kisumu, Kenya; Dr Venkatachalam (Kumar) Udhayakumar, CDC, Atlanta, GA, USA.*

Objective: To assess the influence of infection with and/or exposure to non-malarial coinfections on infection outcome on patients with COVID19 alone or with COVID19 and malaria.

Rationale: In both western Kenya and Burkina Faso, a large proportion of the population live with multiple active infections. It is well documented that the presence of coinfection alters immunological responses and disease outcomes. Examples include the association with increased viral load in HIV-infected individuals following malaria infection, and decreased HIV viral load and increased CD-4 counts following deworming. The overall objective of MALCOV is to characterize the immunological profiles in individuals with SARS-CoV-2 and malaria coinfection, and this will also allow analysis whether these profiles are further affected by the presence of other infections. By running an array of molecular and serological tests to detect other existing infections, we hope to:

1) Document common infections in individuals infected with SARS-CoV-2
2) Characterize immunological profiles in individuals infected with SARS-CoV-2, malaria and additional pathogens

3) Characterize immunological profiles coinfected with SARS-CoV-2 infection and a non-malaria pathogen

We propose to run TaqMan array cards (TAC) developed to detect viral, bacterial, and parasitic pathogens commonly infecting individuals in western Kenya & Burkina Faso. This will require 2mL of whole blood. Samples will also be analysed in serological assays for antibody responses to hepatitis, respiratory pathogens such as respiratory syncytial virus, parainfluenza, *Klebsiella pneumoniae* and parasitic infections such as schistosomiasis and soil-transmitted helminths using the same technology as described in 16.9.2, Kinetics of SARS-CoV-2 and malaria antibodies, page 119. Additionally, stool samples will be assayed using multiplex PCR for schistosomiasis and soil-transmitted helminths, and blood for QuantiFERON-TB Gold I-Tube assay. Results will be used to describe the type and avidity of the response. Additionally, the samples will help to further validate Tetracore's multiplex bead assay (MBA) that is being developed in conjunction with the CDC. This activity is subject to further funding.

16.9.6 Host genetics

Collaborating institutions and contact persons: Dr Lynn Grignard, Dr Tegwen Marlais, Prof Martin Hibberd LSHTM, Keppel St, Bloomsbury, London WC1E 7HT, UK and Dr Vanessa Sancho-Shimizu Imperial College, St Marys Campus, London W2 1PG

Objective: To determine the prevalence of SNP's and haplotypes associated with the risk for COVID19 in study populations in Kenya and Burkina Faso.

Rationale: Genetic markers may be plausibly associated with SARS-CoV-2 acquisition and severity. Recent studies indicate that angiotensin-converting enzyme 2 (ACE2) and transmembrane serine protease 2 (TRPMSS2) are the host receptor for SARS-CoV-2. Variants in these receptors could reduce the association between ACE2 or TRPMSS2 and SARS-CoV-2. Therefore, the expression of human ACE2 and TRPMSS2 might be important for the susceptibility, symptoms, and outcome of SARS-CoV-2 infection.[42] Other variants such as HbS, HbC, ABO blood group and immune-related genes, including those for chemokine receptors and monocyte and T-cell markers have also been implicated. The association between plausible genetic markers and SARS-CoV-2 infection and disease severity will be assessed in this study. Since genetic investigations underlying COVID-19 is an active area of research and any new genetic variants affecting susceptibility or severity of disease will also be included in this study. Whole blood pellet and dried blood spot samples collected in the study are viable for genetic studies. During the consent process, participants can opt-out of genetic testing.

Sample size: All consenting individuals in the screening study (cross-sectional survey, source population) and COVID-19 cohort study.

Methods: Nested multiplex PCR will be developed for all SNP's of interest and a bead-based detection assay for the resulting oligonucleotides will be used to assess their prevalence. In addition, we will use exome sequencing, also known as whole-exome sequencing (WES) may be carried out to investigate the presence of rare disease variants associated with SARS-CoV-2 severity including in a sub-sample of the most severe patients. Genome-wide screening is not planned.

16.9.7 Pharmacokinetic studies

Collaborating institutions and contact persons: Liverpool School of Tropical Medicine: Giancarlo Biagini, Ghaith Aljayyoussi and Steve Ward. University of Liverpool: Andrew Owen.

Objective: To characterise the pharmacokinetic parameters of pyronaridine in patients, assess variables that influence overall pharmacokinetic exposure and build a pharmacokinetic-pharmacodynamic relationship between pyronaridine exposure and SARS-CoV-2 burden.

Rationale: Pyronaridine has displayed efficacy against SARS-CoV-2 virus in preliminary studies with an IC50 of 0.73µM (Giancarlo Biagini lab, LSTM). The clinical efficacy of this compound hasn't been established yet, and a pharmacokinetic study will allow for studying the pharmacokinetic-pharmacodynamic drivers of pyronaridine efficacy in the clinic. The study will also allow for studying disease effects upon its pharmacokinetic profile, particularly in patients who are co-infected with malaria. With a drug that is widely used in malaria within Africa and COVID-19 likely to spread further in the future, it is important to characterise the possible interactions between the two. Furthermore, unlike other antimalarial compounds such as chloroquine, amodiaquine, mefloquine and doxycycline, the pharmacokinetics of pyronaridine has not previously been investigated in saliva. In the current study, saliva is being utilised as a surrogate for drug distribution into the tissues and fluids of the upper respiratory and digestive tracts which are a key site for SARS-CoV-2 transmission and ongoing viral replication. Studies with other antimalarials have also sought to utilise saliva as a readily accessible surrogate for plasma exposures to simplify downstream therapeutic drug monitoring. Therefore, the investigators propose to validate assays to determine conclusively whether substantive penetration of the drug into this matrix occurs in patients. If successful, these methods will be critically important to rationalise outcomes from the nested malaria treatment trial and to provide tools for longer-term evaluation of the exposure-response relationship of pyronaridine as it relates to malaria and SARS-CoV-2 coinfection.

Sample size: All consenting individuals in the pyronaridine arm of the nested malaria treatment trial (~70).

Methods: Blood, plasma and saliva samples will be collected from the patients for analysis using LC/MS-MS. Appropriate extraction methods will be chosen for pyronaridine as previously published depending on the analysed matrix. All analyses will involve at least one internal standard, precision tests and standard curves to characterise the lower and upper limits of quantitation for pyronaridine in each matrix.

The quantified levels of pyronaridine will then be fitted according to a compartmental pharmacokinetic model using Monolix 2018R2 through the IQRtools package in R. The pharmacokinetic models will aim at simultaneously fitting all data to characterise the PK properties of pyronaridine in patients infected with COVID-19 with or without malaria co-infection as well as calculating the drug's partitioning between plasma, blood and saliva. Using a pharmacometrics approach, the models will establish the pharmacokinetic profile of pyronaridine and assessing the effect of covariates including malaria-disease status and other administered compounds at the time upon drug exposure in the different analysed compartments (blood, plasma and saliva).

These pharmacokinetic models will aid in building pharmacokinetic-pharmacodynamic models to relate drug exposure to overall anti-viral activity through the simultaneous modelling of pharmacokinetic profile in blood, plasma and saliva and SARS-CoV-2 levels in swab and saliva samples.

16.10 Appendix X. KEMRI Proposal format checklist

KEMRI Proposal format

	Page	Sections
1. TITLE OF THE PROJECT	1	Cover page
2. INVESTIGATORS AND INSTITUTIONAL AFFILIATIONS:	11	2.1
3. ABSTRACT:	13	2.2.2
4. LAY SUMMARY:	12	2.2.1
5. INTRODUCTION /BACKGROUND:	22	3.1
6. PROBLEM STATEMENT:	23	3.2
7. JUSTIFICATION FOR THE STUDY:	23	3.2
8. STATE THE NULL HYPOTHESIS:	25	3.3
9. (a) GENERAL OBJECTIVES:	26	4
(b) SPECIFIC OBJECTIVES:	26-28	4.1-4.3
10. METHODOLOGY:		
(a) Study site (Geographical)	31	6.1
(b) Study design	28-30	5.1-5.2
(c) Study populations	31-32	6.2.1-6.2.3
(i) Criteria for inclusion of subjects	31-32	6.2.1.1, 6.2.2.1, 6.2.3.1
(ii) Criteria for exclusion of subjects	31-32	6.2.1.2, 6.2.2.2, 6.2.3.2
(iii) Rationale for animal use and justification for species (where applicable).	Not applicable	Not applicable
(d) Sampling	55	11
(i) Sample size determination	55	11.1
(ii) Sampling procedure	36	7.3
(e) Procedures	40-48	9, 10
(i) Description of the type of data to be collected and collection procedures.	40-48	9, 10
(ii) Provisions for data verification and validation in the field and laboratory.	49,53-54	10.8.2, 10.12, 10.13
11. DATA MANAGEMENT:	49-49	10.8
(a) Data collection:	49	10.8.1
(b) Data Storage.	49	10.8.3
(c) Data Analysis	56-60	11.2
12. ETHICAL CONSIDERATIONS	6062	12
13. EXPECTED APPLICATION OF THE RESULTS:	67	12.10
14. TIME FRAME /DURATION OF THE PROJECT	69	13
15. BUDGET :	70, 116	14.3, 16.8
16. JUSTIFICATION OF THE BUDGET:	117	16.8.2
17. ROLE OF INVESTIGATORS	75	16.1.3
18. REFERENCES	71	15

16.11 Appendix XI. Questionnaires

The questionnaires are provided as a separate document.

16.12 Appendix XII. Participant information sheets and informed consent and assent statements

The following participant information sheets and informed consent and assent statements are available as a separate document.

16.12.1 Adults

- MALCOV: Participant Information Sheet, cohort and malaria treatment study, adults
- MALCOV: Consent statement, screening study, adults
- MALCOV: Consent Statement, long term storage of samples and use for future research, adults
- MALCOV: Consent Statement, cohort study, adults
- MALCOV: Consent Statement, malaria treatment study (malaria patients only), adults

16.12.2 Parents/guardians

- MALCOV: Participant Information Sheet for parents/guardians, screening study
- MALCOV: Participant Information Sheet for parents/guardians, cohort study
- MALCOV: Consent statement for parents/guardians, screening study
- MALCOV: Consent Statement for parents/guardians for long term storage of samples and use for future research
- MALCOV: Consent Statement for parents/guardians, cohort study
- MALCOV: Consent Statement for parents/guardians, malaria treatment study (malaria patients only)

16.12.3 Minors

- MALCOV: Participant information sheet for minors
- MALCOV: Assent statement for minors

16.13 Patient information video

A video outlining the main goals and activities of the study is available for study participants to download and will be available to show in study clinics. The link to the video can be found here: https://www.youtube.com/playlist?list=PLguVgnd1OLk9FKRDdLGDVTJCjUoI9FdGz.
