# Supplementary material for: Self-reported COVID-19 severity among persons with tuberculosis infection in western Kenya, 2021
Source: PLOS Glob Public Health. 2025 Apr 30;5(4):e0004372. doi: 10.1371/journal.pgph.0004372 (PMC12043119; doi:10.1371/journal.pgph.0004372)
Supplement: S2 File — ”. (DOCX) [file pgph.0004372.s002.docx]

Malaria as a risk factor for COVID-19 in western Kenya and Burkina Faso (MALCOV): an observational cohort study

# Authors

1. Hellen C Barsosio, MD^1,2^,
2. Brian Tangara, MSc^1^,
3. Tegwen Marlais, PhD^4,5^,
4. Jean Moise T Kabore, PhD^3^,
5. Alfred B Tiono, PhD^3^,
6. Kephas Otieno, MSc^1^,
7. Miriam Wanjiku, BSc^1^,
8. Morine Achieng, BSc^1^,
9. Eric D Onyango, MSc^1^,
10. Everlyne D Ondieki, MPH^1^,
11. Henry Aura, PGDIP^1^,
12. Telesphorus Odawo, HND^1^,
13. David J Allen, PhD^5^,^8^
14. Luke Hannan, MSc^2^,
15. Kevin KA Tetteh, PhD^4, 5^,
16. Issiaka Soulama, PhD^3^,
17. Alphonse Ouedraogo, PhD^3^,
18. Samuel S. Serme, PhD^3^,
19. Ben I Soulama, MD^3^,
20. Aissata Barry, PhD^3^,
21. Emilie Badoum, PhD^3^,
22. Julian Matthewman, MSc^6^,
23. Helena Brazal-Monzó, MSc^5^,
24. Jennifer Canizales, PhD^5^,
25. Anna Drabko, MEng^7^,
26. William Wu, PhD^7^,
27. Simon Kariuki, PhD^1^
28. Prof Maia Lesosky, PhD^2^
29. Prof Sodiomon B Sirima, PhD^3^,
30. Prof Chris Drakeley*, PhD^5^,
31. Prof Feiko O ter Kuile*, PhD^1,2^

*Contributed equally

## Affiliations

1. Kenya Medical Research Institute, Centre for Global Health Research, Kisumu, Kenya
2. Department of Clinical Sciences, Liverpool School of Tropical Medicine, Liverpool, United Kingdom
3. Groupe de Recherche Action en Santé (GRAS), Ouagadougou, Burkina Faso.
4. Department of Clinical Research, Faculty of Infectious and Tropical Diseases, London School of Hygiene & Tropical Medicine, London, United Kingdom
5. Department of Infection Biology, Faculty of Infectious and Tropical Diseases, London School of Hygiene & Tropical Medicine, London, United Kingdom
6. Department of Non-Communicable Disease Epidemiology, Faculty of Epidemiology and Population Health, London School of Hygiene & Tropical Medicine, London, United Kingdom
7. Quantitative Engineering Design (QED.ai), Warsaw, Poland
8. Department of Comparative Biomedical Sciences, Faculty of Health and Medical Sciences, University of Surrey, Guildford, United Kingdom

## Corresponding author

Prof Feiko ter Kuile

Liverpool School of Tropical Medicine, Pembroke Place, Liverpool L3 5QA, United Kingdom

Mobile UK: +44 (0)7846 377 369

E-mail: [feiko.terkuile@lstmed.ac.uk](mailto:feiko.terkuile@lstmed.ac.uk)

# Word count

Main text: 3016x (out of 3,500)

Abstract: 369 (out of 300)

Reference: 25 (out of 30)

# Abstract

Background: We conducted a cohort study in newly diagnosed COVID-19 patients of all ages in western Kenya and Burkina Faso to determine whether coinfection with *Plasmodium falciparum* malaria affects COVID-19 severity or duration.

Methods: Participants were screened for SARS-CoV-2 using rapid antigen tests. Patients co-infected with malaria were treated with 3-day artemether-lumefantrine or pyronaridine-artesunate. COVID-19 disease progression was assessed daily by FLU-PRO-Plus questionnaires until day 14. Nasal swabs and blood samples were taken on enrolment, days 0, 3, 7, 14, and 28. SARS-CoV-2 viral load was assessed by RT-PCR. All analyses were adjusted for country, enrolment age, disease severity, and viral load.

Findings: From February 2021 to January 2022, 742 COVID-19 patients were enrolled; 151 (20%) had malaria. Malaria patients were younger: 49/515 (32%) were aged <15 years vs 35/591 (6%) without malaria (p<0.0001). SARS-CoV-2 clearance in the first week was slower among malaria patients (aHR=0.69 95% confidence interval 0.51-0.94, p=0.017), although by Day-7 similar numbers in the malaria and non-malaria groups had cleared SARS-CoV-2 (48% [58/120] vs 53% [253/477], adjusted risk ratio [RR]:0.79, 0.58-1.08, p=0.14) and this was 99% (116/117) vs 98% (375/381) by day-28 (aRR=0.99, 0.79-1.24, p=0.95). There were no differences in viral load at enrolment or Day-7. Patients with malaria were more likely to have moderate-to-severe disease at enrolment (all ages: 68% vs 60%, p<0.074; <15 years: p=0.13; ≥15 years: p=0.022), but the time to clearance of moderate-to-severe symptoms was similar (aHR=1.14, 0.91-1.42, p=0.26). Overall, 3 (2.0%) and 9 (1.5%) patients with and without malaria had to be hospitalised, and 2 (1.3%) and 3 (0.5%) died.

Interpretation: Malaria coinfection did not aggravate or reduce the severity or duration of COVID-19. The clearance rate of SARS-CoV-2 was slightly slower in the first week in patients co-infected with malaria than in those without. However, clinical recovery rates were similar, and there was no evidence that acute uncomplicated malaria affected COVID-19 disease progression or the duration of illness after successful treatment of malaria.

Funding: Bill and Melinda Gates Foundation

# Introduction

Coronavirus disease 2019 (COVID-19) due to severe acute respiratory syndrome coronavirus-2 (SARS-CoV-2) has caused significant morbidity and mortality globally, adversely affecting healthcare systems and the global economy. At the start of the COVID-19 pandemic, it was unclear whether and how malaria affects COVID-19 severity, immune responses to SARS-CoV-2, viral loads, and/or the duration of shedding and potentially its onward spread.^1^ The impact of such coinfections can range from harmful to inconsequential or even beneficial.^2^ Several ecological studies have shown that malaria-endemic areas have reported a significantly lower burden of COVID-19 than temperate zones.^3,4^ Although many environmental, genetic, sociocultural and health systems related factors may contribute to this actual or reported lower burden,^5,6^ combined with the finding that malaria induces trained immunity that may induce some non-specific cross-tolerance to other infections,^7-9^ led to the hypothesis that malaria may reduce COVID-19 severity.^10-15^

Individual level data remain scarce three years after the start of the pandemic. One large study of hospitalised adults from Uganda suggests that malaria may protect from progression to severe SARS-CoV-2 disease,^16^ while another retrospective study suggested an increased risk of mortality with coinfection,^17^ and one prospective cohort study in Mali showed no effect on symptomatic illness.^18^ Other studies have suggested faster SARS-CoV-2 clearance among patients co-infected with malaria,^19,20^ although these analyses were not adjusted for the effect of baseline viral load and age.

We have conducted a multisite observational cohort study in over 750 newly diagnosed COVID-19 patients of all ages and a nested malaria treatment trial among 143 malaria and SARS-CoV-2 co-infected participants in western Kenya and Burkina Faso to determine the effect of malaria and malaria treatment on SARS-CoV-2 viral clearance and COVID-19 disease progression.

# Methods

## Study design and participants

This observational cohort study was conducted in 6 sites, including 5 sites in Kenya in areas with perennial malaria transmission and 1 site in Burkina Faso with intense, highly seasonal malaria transmission. The cohort was nested within a larger cross-sectional screening study to identify patients with COVID-19 among suspected COVID-19 patients, their case contacts or other high-risk populations. Patients with a positive SARS-CoV-2 RT-PCR or rapid antigen test were invited to join the cohort study. Participants co-infected with rapid diagnostic tests or microscopy-confirmed malaria were invited to enrol in a nested, individually randomised, 2-arm, open-label controlled treatment trial reported elsewhere.^21^

Participants were eligible if they were aged >=6 months, had RT-PCR or rapid-antigen test confirmed COVID-19, were residents in the study area, agreed not to self-medicate with chloroquine or hydroxychloroquine, and agreed to be contacted by phone. Potential participants were excluded if it was anticipated that they could not adhere to the follow-up schedule. All study participants provided written informed consent. The study was approved by the ethics committees of the Kenya Medical Research Institute, the Ethics Committee for Health Research, the Ministry of Higher Education, Scientific Research and Innovation, the Ministry of Health, Burkina Faso, the London School of Hygiene and Tropical Medicine and the Liverpool School of Tropical Medicine (LSTM). The study is registered with ClinicalTrials.gov NCT04695197.

## Procedures

Daily FLU-PRO-Plus plus questionnaires were used to assess COVID-19 disease severity and symptom resolution until day 14. FLU-PRO is a patient-reported outcome data collection instrument developed to measure the intensity and frequency of symptoms of viral respiratory tract diseases.^22^ Questions on loss of taste and smell have been added to the original FLU-PRO to adapt it for COVID-19, resulting in the FLU-PRO Plus.^23^ Patients were then seen again on days 21 and 28. Patients enrolled in the nested malaria treatment trial were also seen on day 42 to assess the anti-malarial treatment response.

Mid-nasal swab samples for SARS-CoV-2 RT-PCR, stored in viral transport media (Biocomma), and blood samples for immunological assays were taken on days 0, 3, 7, 14, and 28. If patients remained SARS-CoV-2 positive on day 28, another SARS-CoV-2 PCR test was conducted on day 42. SARS-CoV-2 RNA was detected by real-time reverse transcription polymerase chain reaction (real-time RT-PCR) using primers and probes targeting the N gene^24^ and the Luna Universal Probe One-Step RT-qPCR Kit (New England Biolabs, Hitchin, UK) (appendix p 23).

## Outcomes

The primary endpoint was the severity of COVID-19 by day 28 using the WHO clinical progression scale and the FLU-PRO-Plus self-reported disease severity. Secondary endpoints included the cumulative proportions of patients that had cleared their symptoms by days 7 and 14, the total number of days and the time to resolve any or moderate-or-severe symptoms. Other secondary endpoints included the cumulative incidence of patients who had cleared SARS-CoV-2 by day 7, 14, and 28, as determined by RT-PCR, time to SARS-CoV-2 clearance, median viral load, the total number of days and the time to resolution of symptoms (any) or moderate-or-severe symptoms.

## Statistical analysis

The primary endpoint for the sample size calculations was the cumulative proportion of patients that progressed at least one category in disease severity from enrolment to day 28. At least one category increase in disease severity was defined as progressing from either asymptomatic or mild disease at enrolment to at least moderate disease, from moderate to severe disease, or from severe disease to death. The sample size calculations to determine the crude impact (i.e., unadjusted for potential confounders) of malaria on COVID-19 disease regression suggested that a pooled sample size of 708 (673 completers), of whom approximately 142 were malaria positive and 566 malaria negative (135 and 538 completers) would provide 90% power to detect a 50% difference in the primary endpoint from 30% in the malaria-negative group to 45% in the malaria positive group (alpha=0.05, 5% loss to follow-up by day 28). The true power was assumed to be lower when the confounding effect of baseline differences between malaria and non-malaria patients, such as age, were considered. However, the inflation in the sample size required to adjust for differences in potentially confounding factors at baseline was difficult to estimate *a priori* as similar studies had not been completed when the study was designed. Similar crude sample size calculations using 80% power suggested that 522 participants are required. Thus, overall, a sample size of 708 participants was anticipated to provide approximately 80% power to detect a 50% difference in the primary endpoint, allowing for a 35.6% inflation in sample size (708/522=1.356) to take adjustment for confounding into account in multivariate or stratified analysis. The sample size calculation for the COVID-19 cohort study also assumed that each country would be able to recruit 354 COVID-19 participants and that approximately 20% of them would be co-infected with malaria, representing a weighted average of 15% of 354 COVID-19 cases in Kenya and 25% of 354 cases in Burkina Faso.

Statistical analyses were done using R version 4.3.2. Cox regression was used for time-to-event endpoints to obtain hazard ratios (HR) and corresponding 95% confidence intervals (CIs). Wilkinson-Rank tests were used to compare viral load. Negative binomial regression with the follow-up time as an offset was used for count variables (e.g. the number of days participants were symptomatic) to obtain incidence rate ratios and 95% CIs.

The adjusted analysis was the primary analysis and included the co-variates country, age in years, presence or absence of moderate-to-severe symptoms, and SARS-CoV-2 viral load. Missing covariates were imputed using simple imputation. Two-sided p-values <0.05 were used to define statistical significance. The analyses of disease progression and clearance of symptoms included all enrolled participants, referred to as the intention-to-treat (ITT) population. The analysis of the clearance rate of SARS-CoV-2 includes all patients with a valid SARS-CoV-2 PCR test at enrolment and is referred to as the mITT population (appendix p-23).

## Role of the funding source

The funders had no role in the study design, collection, analysis, interpretation of the data, or report writing.

# Results

## Study population and baseline characteristics

From January 08, 2021, to January 24, 2022, 756 patients with COVID-19 were screened for inclusion, and 742 had valid malaria diagnostics results and were included in this analysis; 627 (85%) were recruited in Kenya and the remaining 115 (15%) in Burkina Faso (Table-1). Most participants were recruited during the wave from December 2021 to January 2022, when Omicron (BA.1) was the predominant variant of concern. This was associated with a major surge in COVID-19 cases, including in Kenya.

Of the 742 participants, 151 (20%) had microscopy or RDT-confirmed malaria, and 143 were enrolled in the nested malaria treatment trial, as reported elsewhere.^21^ Overall, 11 (1%) were aged <5 years, 73 (10%) 5-14 years, and the remaining 658 (89%) were 15 years or older; 333 (45%) were male. None of the patients were considered severely ill; 10 were recruited while admitted to the hospital; 8 for isolation purposes at the beginning of the pandemic, and 2 had moderate-to-severe disease but were in stable clinical condition. 109 (15%) of participants had comorbidities (cardiovascular disease, diabetes, HIV/AIDS) or were pregnant or in their post-partum period (5, (1%)). Overall, 32 (4%) participants had been vaccinated against COVID-19, with equal proportions in each group.

Almost all participants (707/742, 95%) reported being symptomatic, and 93% (673/721) said this affected their normal daily activities; 452 of 737 (61%) with valid FLU-PRO-Plus data at enrolment self-reported their symptoms to be of moderate-to-severe severity, and 285 (60%) reported to be in poor or fair health. The median (IQR) viral load among the 117 PCR test-positive participants was 2,862 (229, 34,332) copies.

## Demographic characteristics among participants with and without malaria

The 151 patients co-infected with malaria differed in many aspects from those without malaria (Table-1). As expected, the malaria co-infected patients were younger; only 35 out of 591 (6%) of COVID-19 patients without malaria were aged <15 years compared to 49 out of 151 (32%) among those with malaria (p<0.0001). Malaria patients were also less likely to be male (p=0.0069), or to have comorbidities, and more likely to be recruited during the later Delta and Omicron waves (P<0.0001). Coinfection with malaria was also more common in Kenya (23%, 144/627) than in Burkina Faso (6%, 7/115) (p<0.0001).

## Clinical characteristics among participants with and without malaria

There were no major differences between the clinical presentation of COVID-19 patients with and without malaria, other than that patients with malaria were more likely to have a history of fever in the previous 2 weeks (19% vs 6%, P<0.0001) (Table-2). They were equally likely to be symptomatic at enrolment (malaria 97% vs no-malaria 95%), to report being unable to conduct their normal daily activities (malaria 95% vs no-malaria 93%) and to consider themselves in poor or fair health (as opposed to good to excellent health) (malaria 62% vs no-malaria 60%). The average O2 saturation was the same (96.3% in both groups). Patients co-infected with malaria, however, were at slightly higher risk of self-reported moderate to severe symptoms than non-malaria patients (68% vs 60%) (p=0.074), and this was 74% vs 61% (p=0.022) in those aged >=15 years, and 56% vs 37% in children <15 years (p=0.13) (Table-2).

## Symptom clearance

The time to resolution of symptoms was similar in both groups (aHR=1.14, 95% CI 0.91-1.42, p=0.26 (Figure-2), and by day 7, a similar proportion of participants were still symptomatic (62% in the malaria group and 58% in the non-malaria group, p=0.86). This was 13% in both groups by day 14 (p=0.50). The total number of days they reported symptoms was also comparable (aIRR=0.95, 0.86-1.05, p=0.31) (Table-3). Similar observations were seen with moderate-to-severe symptoms (Figure-3). By day 14, <1% of patients in both groups still reported moderate-to-severe symptoms, and the proportion of days that patients reported moderate-to-severe symptoms was also similar (13% vs 12%, IRR=1.00, 0.84-1.20, p=0.96). There were no obvious differences in the effects of malaria on symptom resolution by age, and patterns overall were similar in children <15 years and older adolescents and adults aged >= 15 years (Figure-2 and Figure-3). However, the number of children with moderate-to-severe symptoms was too small to draw meaningful conclusions. Of note was that patients with malaria were less likely to report that their symptoms affected their daily activities: of the 93% who reported this at enrolment, 47% still reported this by day 7 compared to 63% among those without malaria at enrolment (p=0.13) (Table-3).

## Disease progression

Overall, 12 patients (1.6%) were hospitalised for severe disease after enrolment: 3/151 (2%) in the malaria group and 9/591 (1.5%) in the non-malaria group, leading to 2 (1.3%) and 3 (0.5%) deaths, respectively. Both deaths in the malaria group were from respiratory failure, one of whom also had severe malaria. Two deaths in the non-malaria group were from respiratory failure and one from perforated peptic ulcer with anaemia. 7 out of 9 hospital admissions in the non-malaria group were related to acute respiratory distress syndrome (ARDS) suggestive of COVID-19, which was 2 out of 3 for the malaria group.

## SARS-CoV-2 viral clearance

Overall, 590 of the 742 participants had valid SARS-CoV-2 PCR data at enrolment and contributed to viral clearance analyses. The median (IQR) SARS-CoV-2 viral load per reaction at enrolment was comparable between malaria and no-malaria patients: malaria 2,260 (241-18,073) vs no-malaria: 3,001 (225-39,782) (p=0.2), and this was observed in adults and children (Table-2).

Overall, the time to SARS-CoV-2 clearance was similar between the two groups by day 28 (adjusted hazard ratio [aHR]: 0.83, 0.67-1.04), p=0.11), and the median (IQR) time to SARS-CoV-2 clearance was 7 (7-14) days in both groups (Table-4). However, the rate of viral clearance was somewhat slower in the malaria group in the first week (aHR=0.69, 0.51-0.94, p=0.017); by day 3, 14% (17/122) in the malaria group had cleared SARS-CoV-2 versus 27% (126/473) in the non-malaria group, although by day 7 these numbers were not as dissimilar (48% [58/120] vs 53% [253/477]), and by day 14, these proportions were 86% (105/122) and 73% (347/473) (aRR=0.99, 0.78-1.25, p=0.92) (Table-4).

# Discussion

This cohort study of patients with COVID-19 of all ages suggests that malaria is not an aggravating or protective factor of COVID-19. As expected, there were clear differences in demographic characteristics, such as age, with malaria being more common in children, and thus age-related comorbidities, such as cardiovascular disease, diabetes and HIV/AIDS, less common in this group. The clearance rate of SARS-CoV-2 was slightly slower in patients with *P. falciparum* malaria than in those without malaria, but only in the first week. However, there was no evidence that this is associated with a greater COVID-19 disease severity or longer duration of illness. After adjustment for age, overall differences in clinical symptom resolution were unremarkable and of limited clinical relevance. We conclude that acute malaria is not a risk factor for COVID-19 disease severity, duration, or shedding of SARS-CoV-2.

Patients co-infected with malaria did not have higher or lower SARS-CoV-2 viral loads than those without malaria at enrolment, and there were no obvious differences in disease severity at presentation. The viral load at enrolment was also similar between the two groups. None of the patients was severely ill at enrolment, which reflected the inherent selection bias from the study design, primarily screening patients in outpatient departments and dedicated COVID-19 screening centres, including as part of COVID-19 contact tracing. However, almost all patients (96%) were symptomatic, and 93% reported that their illness affected their normal daily activities. Co-infected patients were slightly more likely to consider their symptoms moderate-to-severe (68% vs 60%) (p=0.074) (74% vs 61% in adults, p=0.022), but they recovered equally as fast following successful treatment of malaria compared to the COVID-19 patients without malaria.

Because fewer than 2% of patients developed severe disease requiring hospitalisation, the study was underpowered to determine whether coinfection with malaria enhances the risk of hospitalisation. Overall, 2% vs 1.5% of patients in the malaria and non-malaria groups were hospitalised, and 2 (1.3%) vs 0.5% died.

Few patients had received a COVID-19 vaccine, as most were enrolled before the roll-out of COVID-19 vaccines in Kenya around mid-2021, with the highest risk group, including uniformed services and health care workers, being targeted first, followed by those aged over 58 years. Burkina Faso also started rolling vaccination in mid-2021, approximately halfway through the study. High rates of vaccine hesitancy have been reported in both countries.^25^

Our study has several strengths. The follow-up success rate was excellent, with 94% of participants contributing to the daily follow-up using the FLU-PRO-Plus questionnaire, despite the challenges related to the national restrictions in both countries in people movement during the pandemic’s peak. This study also has several limitations: our study recruited patients with mostly mild to moderate symptoms and did not recruit hospitalised patients. Thus, our study was not designed to address whether coinfection with malaria is a risk factor or potential protective factor for fatal disease in this more severe hospitalised group.

Although our results suggest uncomplicated symptomatic malaria does not affect the risk of disease progression, our study was not designed to address whether lifetime exposure to previous malaria infections in uninfected or asymptomatic individuals, rather than acute malaria may be associated with any protective effect of malaria on COVID-19 severity.^18^ It remains possible that malaria-induced immunity/tolerance may have non-specific cross-tolerance effects on SARS-CoV-2 infection, potentially suppressing viral infection early and/or tempering excessive inflammatory responses to modify the clinical presentation of COVID-19.^6-9^

Conclusion: Coinfection with malaria did not aggravate or reduce the severity or duration of COVID-19. The clearance rate of SARS-CoV-2 was slightly slower in patients co-infected with *P. falciparum* malaria in the first week than among those without malaria, and adult patients co-infected with malaria were more likely to self-report moderate-to-severe rather than mild symptoms at enrolment. However, their clinical recovery rate was similar, and there was no evidence that malaria was associated with a greater COVID-19 disease severity or a longer duration of illness.

# Article information

## Contributors

FOtK and CD conceived the idea for the study design. HCB, ABT, DJA, ML, SBS, CD and FOtK designed the study with input from all other authors. All authors contributed to data acquisition. KO, IS, CD, KT, DA and TM coordinated the laboratory component. EDO, JM, AD and WW coordinated the data management. LH and ML did the statistical analysis. FOtK wrote the first draft of the manuscript. All authors interpreted the data and critically reviewed the manuscript. All authors had full access to all the data in the study and had final responsibility for the decision to submit for publication.

## Declaration of interests

All authors declare no competing interests.

## Data sharing

The protocol is available in this publication’s appendix. Individual participant data will be available from the Worldwide Anti-malarial Resistance Network (WWARN) repository approximately three months after publication.

## Acknowledgements

We thank all the study participants and the research assistants who conducted the study. We are very thankful to Tracy Seddon, Helen Wong, and Katie Davies in Liverpool, UK; Raquel Thalheimer, Anastasia Ioakeimidou, and Ramlat Jose in London, UK; Benta Kamire, Sheila Nyarinda, Josephine Owade and Mildred Okello in Kisumu, Kenya; Nadège Kinda, Ernestine Yougbare, and Lydia Dabre in Burkina Faso for their excellent managerial and administrative support. We are also grateful to the Directors of Health, County Health Management teams, and Medical Superintendent of Kisumu, Busia, and Siaya Counties for accommodating the study in Kenya, and the Directors of Health and staff of Health Region of Ouagadougou, Health District of Kossodo, and the Response Centre for Health Emergencies for accommodating the study in Burkina Faso. This study is published with the permission of the Director, KEMRI.

This study received financial support from the Bill and Melinda Gates Foundation (INV-017985 and INV-019400). The findings and conclusions contained within are those of the authors and do not necessarily reflect the positions or policies of the Bill & Melinda Gates Foundation. We are very grateful to Jean-Luc Bodmer and Scott Miller from the Bill and Melinda Gates Foundation for their continued support and encouragement throughout the study. We thank Chris Ockenhouse from PATH and Richard Steketee from USAID for supporting the study concept and design.

# References

1. Gutman JR, Lucchi NW, Cantey PT, Steinhardt LC, Samuels AM, Kamb ML, Kapella BK, McElroy PD, Udhayakumar V, Lindblade KA, 2020. Malaria and Parasitic Neglected Tropical Diseases: Potential Syndemics with COVID-19? Am J Trop Med Hyg 103: 572-577.

2. Fraga A, Mosca AF, Moita D, Simas JP, Nunes-Cabaco H, Prudencio M, 2023. SARS-CoV-2 decreases malaria severity in co-infected rodent models. Front Cell Infect Microbiol 13: 1307553.

3. Iesa MAM, Osman MEM, Hassan MA, Dirar AIA, Abuzeid N, Mancuso JJ, Pandey R, Mohammed AA, Borad MJ, Babiker HM, Konozy EHE, 2020. SARS-CoV-2 and Plasmodium falciparum common immunodominant regions may explain low COVID-19 incidence in the malaria-endemic belt. New Microbes New Infect 38: 100817.

4. Habibzadeh F, 2023. Malaria and the incidence of COVID-19 in Africa: an ecological study. BMC Infect Dis 23: 66.

5. Lalaoui R, Bakour S, Raoult D, Verger P, Sokhna C, Devaux C, Pradines B, Rolain JM, 2020. What could explain the late emergence of COVID-19 in Africa? New Microbes New Infect 38: 100760.

6. Mbow M, Lell B, Jochems SP, Cisse B, Mboup S, Dewals BG, Jaye A, Dieye A, Yazdanbakhsh M, 2020. COVID-19 in Africa: Dampening the storm? Science 369: 624-626.

7. Schrum JE, Crabtree JN, Dobbs KR, Kiritsy MC, Reed GW, Gazzinelli RT, Netea MG, Kazura JW, Dent AE, Fitzgerald KA, Golenbock DT, 2018. Cutting Edge: Plasmodium falciparum Induces Trained Innate Immunity. J Immunol 200: 1243-1248.

8. Guha R, Mathioudaki A, Doumbo S, Doumtabe D, Skinner J, Arora G, Siddiqui S, Li S, Kayentao K, Ongoiba A, Zaugg J, Traore B, Crompton PD, 2021. Plasmodium falciparum malaria drives epigenetic reprogramming of human monocytes toward a regulatory phenotype. PLoS Pathog 17: e1009430.

9. Rogers KJ, Shtanko O, Vijay R, Mallinger LN, Joyner CJ, Galinski MR, Butler NS, Maury W, 2020. Acute Plasmodium Infection Promotes Interferon-Gamma-Dependent Resistance to Ebola Virus Infection. Cell Rep 30: 4041-4051 e4.

10. Osei SA, Biney RP, Anning AS, Nortey LN, Ghartey-Kwansah G, 2022. Low incidence of COVID-19 case severity and mortality in Africa; Could malaria coinfection provide the missing link? BMC Infectious Diseases 22: 1-11.

11. Hussein MIH, Albashir AAD, Elawad O, Homeida A, 2020. Malaria and COVID-19: unmasking their ties. Malar J 19: 457.

12. Napoli PE, Nioi M, 2020. Global Spread of Coronavirus Disease 2019 and Malaria: An Epidemiological Paradox in the Early Stage of A Pandemic. J Clin Med 9.

13. Altable M, de la Serna JM, 2021. Protection against COVID-19 in African population: Immunology, genetics, and malaria clues for therapeutic targets. Virus Res 299: 198347.

14. Kalungi A, Kinyanda E, Akena DH, Kaleebu P, Bisangwa IM, 2021. Less Severe Cases of COVID-19 in Sub-Saharan Africa: Could Coinfection or a Recent History of Plasmodium falciparum Infection Be Protective? Front Immunol 12: 565625.

15. Orish VN, Boakye-Yiadom E, Ansah EK, Alhassan RK, Duedu K, Awuku YA, Owusu-Agyei S, Gyapong JO, 2021. Is malaria immunity a possible protection against severe symptoms and outcomes of COVID-19? Ghana Med J 55: 56-63.

16. Achan J, Serwanga A, Wanzira H, Kyagulanyi T, Nuwa A, Magumba G, Kusasira S, Sewanyana I, Tetteh K, Drakeley C, Nakwagala F, Aanyu H, Opigo J, Hamade P, Marasciulo M, Baterana B, Tibenderana JK, 2022. Current malaria infection, previous malaria exposure, and clinical profiles and outcomes of COVID-19 in a setting of high malaria transmission: an exploratory cohort study in Uganda. Lancet Microbe 3: e62-e71.

17. Hussein R, Guedes M, Ibraheim N, Ali MM, El-Tahir A, Allam N, Abuakar H, Pecoits-Filho R, Kotanko P, 2022. Impact of COVID-19 and malaria coinfection on clinical outcomes: a retrospective cohort study. Clin Microbiol Infect 28: 1152 e1-1152 e6.

18. Woodford J, Sagara I, Diawara H, Assadou MH, Katile A, Attaher O, Issiaka D, Santara G, Soumbounou IH, Traore S, Traore M, Dicko OM, Niambele SM, Mahamar A, Kamate B, Haidara B, Sissoko K, Sankare S, Diarra SDK, Zeguime A, Doritchamou JYA, Zaidi I, Dicko A, Duffy PE, 2022. Recent malaria does not substantially impact COVID-19 antibody response or rates of symptomatic illness in communities with high malaria and COVID-19 transmission in Mali, West Africa. Front Immunol 13: 959697.

19. Mahajan NN, Gajbhiye RK, Bahirat S, Lokhande PD, Mathe A, Rathi S, Warty N, Mahajan KN, Srivastava V, Kuppusamy P, Mohite SC, 2021. Coinfection of malaria and early clearance of SARS-CoV-2 in healthcare workers. J Med Virol 93: 2431-2438.

20. Rathi PM, Mahajan NN, Srivastava V, Junare PR, Bansal S, Kaushal N, Rathod DB, 2023. Early virus clearance of SARS-CoV-2 among coinfection with malaria. J Vector Borne Dis 60: 211-214.

21. Tangara B, Barsosio HC, Marlais T, Kabore JMT, Tiono AB, Otieno K, Wanjiku M, Achieng M, Onyango ED, Ondieki ED, Aura H, Odawo T, Allen DJ, Hannan L, Tetteh KK, Soulama I, Ouedraogo A, Serme SS, Soulama BI, Barry A, Badoum E, Matthewman J, Brazal-Monzó H, Canizales J, Drabko A, Wu W, Kariuki S, Lesosky M, Sirima SB, Drakeley C, ter Kuile FO, 2024 (in preparation). Artemether-lumefantrine versus pyronaridine-artesunate for the treatment of malaria in patients with mild to moderate COVID-19 in Kenya and Burkina Faso: a randomised open-label trial (MALCOV).

22. Powers JH, 3rd, Bacci ED, Leidy NK, Poon JL, Stringer S, Memoli MJ, Han A, Fairchok MP, Coles C, Owens J, Chen WJ, Arnold JC, Danaher PJ, Lalani T, Burgess TH, Millar EV, Ridore M, Hernandez A, Rodriguez-Zulueta P, Ortega-Gallegos H, Galindo-Fraga A, Ruiz-Palacios GM, Pett S, Fischer W, Gillor D, Moreno Macias L, DuVal A, Rothman R, Dugas A, Guerrero ML, 2018. Performance of the inFLUenza Patient-Reported Outcome (FLU-PRO) diary in patients with influenza-like illness (ILI). PLoS One 13: e0194180.

23. Richard SA, Epsi NJ, Pollett S, Lindholm DA, Malloy AMW, Maves R, Utz GC, Lalani T, Smith AG, Mody RM, Ganesan A, Colombo RE, Colombo CJ, Chi SW, Huprikar N, Larson DT, Bazan S, Madar C, Lanteri C, Rozman JS, English C, Mende K, Tribble DR, Agan BK, Burgess TH, Powers JH, 3rd, Epidemiology I, Clinical Characteristics of Pandemic Infectious Diseases C-CSG, 2021. Performance of the inFLUenza Patient-Reported Outcome Plus (FLU-PRO Plus) Instrument in Patients With Coronavirus Disease 2019. Open Forum Infect Dis 8: ofab517.

24. Lu X, Wang L, Sakthivel SK, Whitaker B, Murray J, Kamili S, Lynch B, Malapati L, Burke SA, Harcourt J, Tamin A, Thornburg NJ, Villanueva JM, Lindstrom S, 2020. US CDC Real-Time Reverse Transcription PCR Panel for Detection of Severe Acute Respiratory Syndrome Coronavirus 2. Emerg Infect Dis 26: 1654-65.

25. Abubakari SW, Workneh F, Asante KP, Hemler EC, Madzorera I, Wang D, Ismail A, Assefa N, Azemraw T, Lankoande B, Nuhu AR, Chukwu A, Mapendo F, Millogo O, Olufemi AA, Okpara D, Boudo V, Mwanyika-Sando M, Berhane Y, Baernighausen T, Oduola A, Vuai S, Sie A, Soura A, Killewo J, Tajudeen R, Fawzi WW, Smith ER, 2023. Determinants of COVID-19 vaccine readiness and hesitancy among adults in sub-Saharan Africa. PLOS Global Public Health 3: e0000713.

# Figures

## Figure-1: Study profile

| 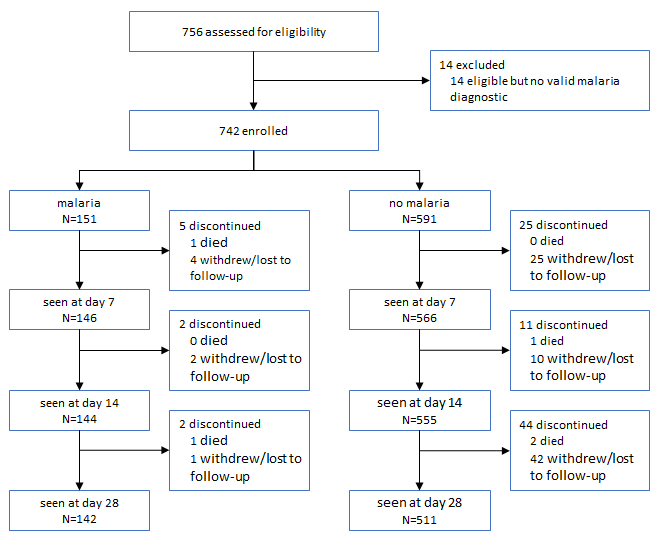 |
| --- |
|  |

## Figure-2: Resolution of all symptoms (FLU-PRO-Plus)

| Overall | ≥ 15 years |
| --- | --- |
| 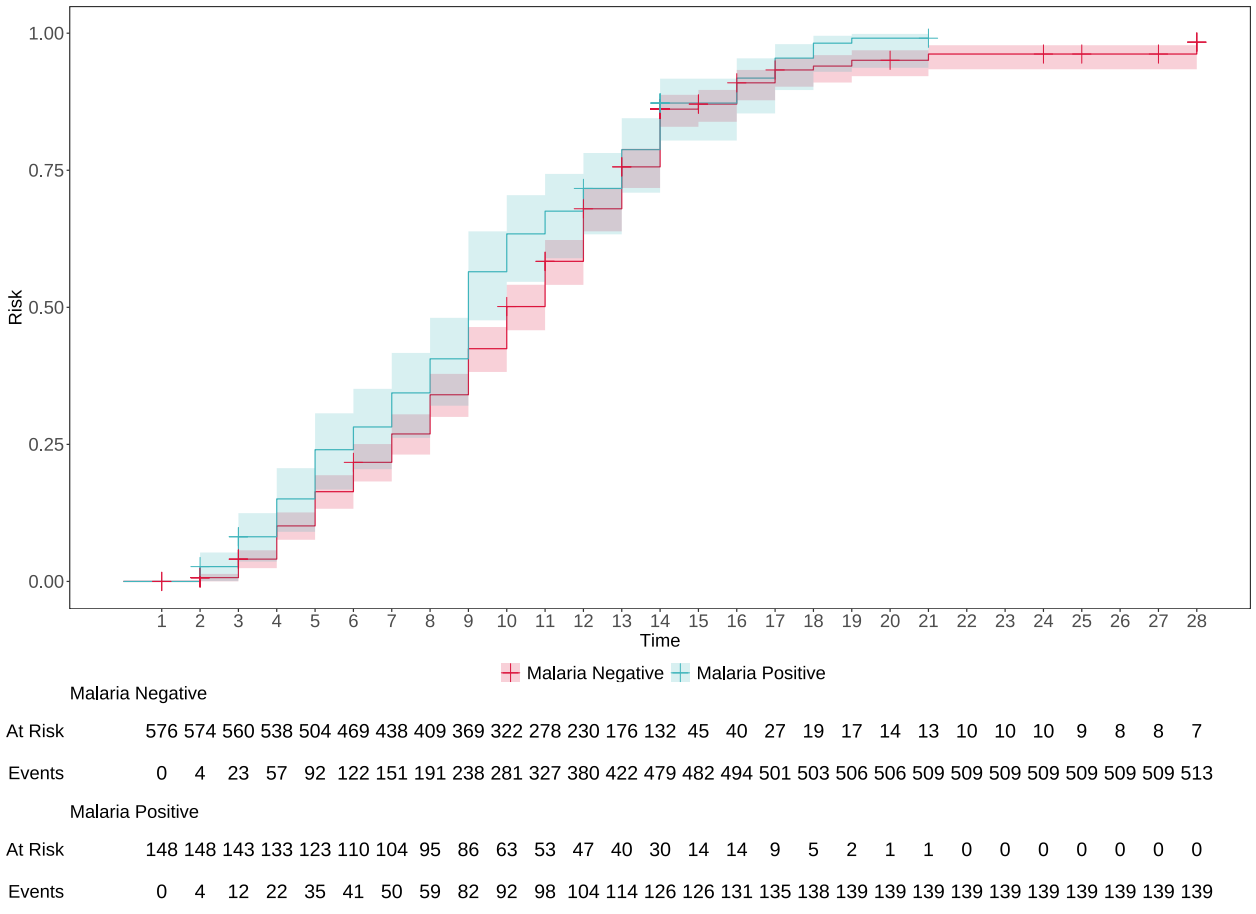 | 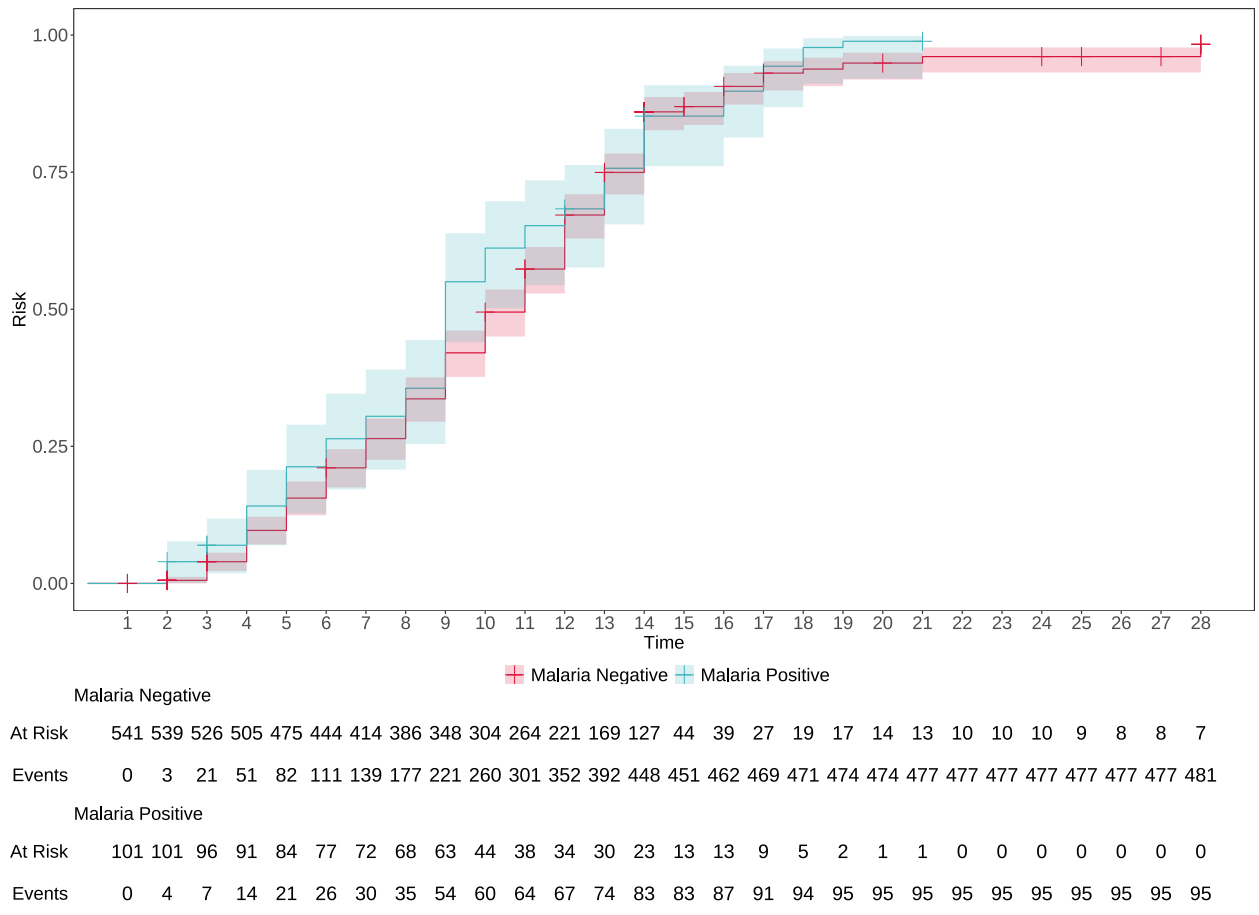 |
| **<15 years** | Notes |
| 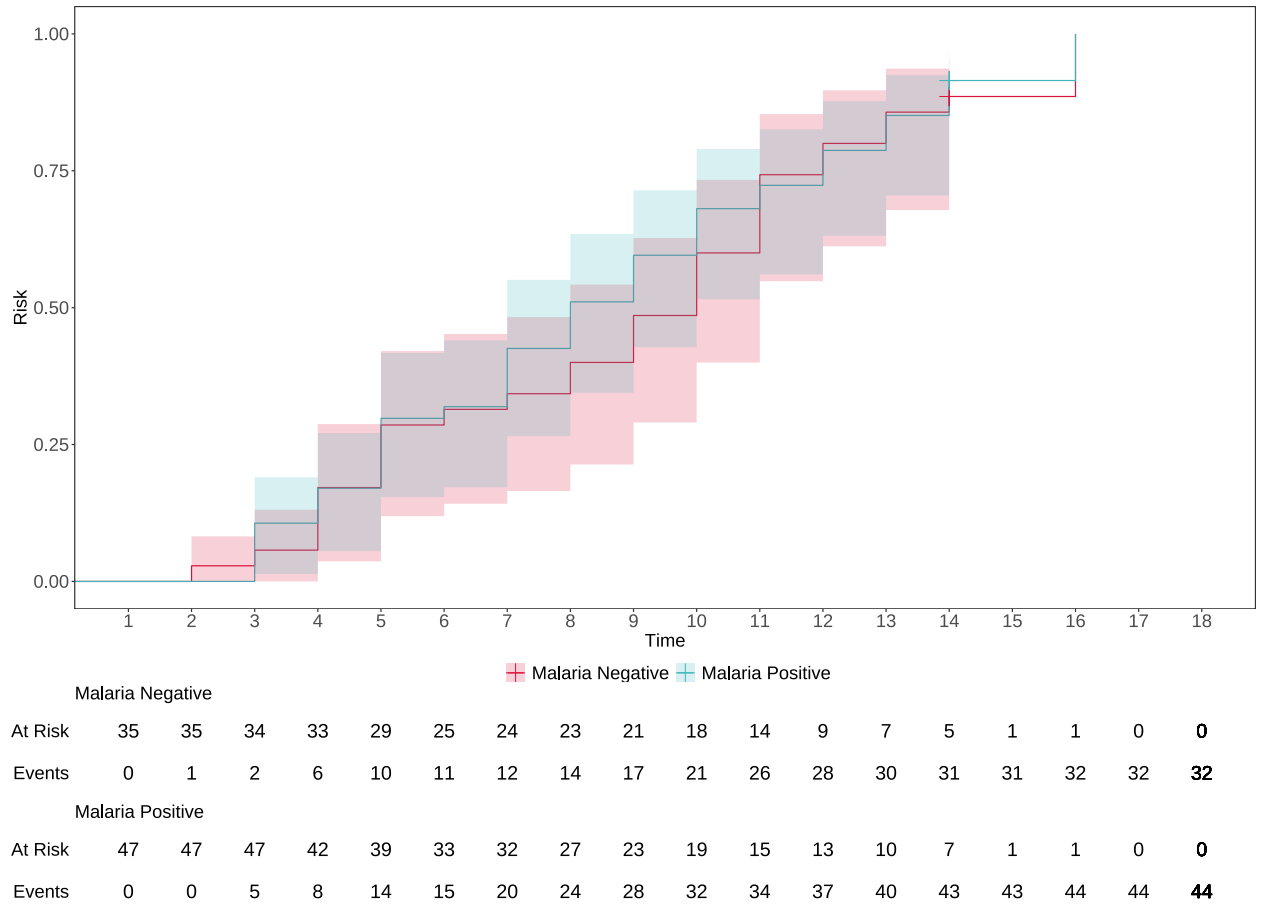 | Hazard ratio and 95% CI for time to symptom clearance obtained from cox-regression models adjusted for viral load, age in years, disease severity at enrolment, and country.  Overall: 1.14 (0.91-1.42), p=0.26  ≥ 15 years: 1.09 (0.85-1.41), p=0.48  <15 years: 1.36 (0.72-2.58), p=0.34 |

## Figure-3: Resolution of moderate to severe symptoms (FLU-PRO-Plus)

| Overall | ≥ 15 years |
| --- | --- |
| 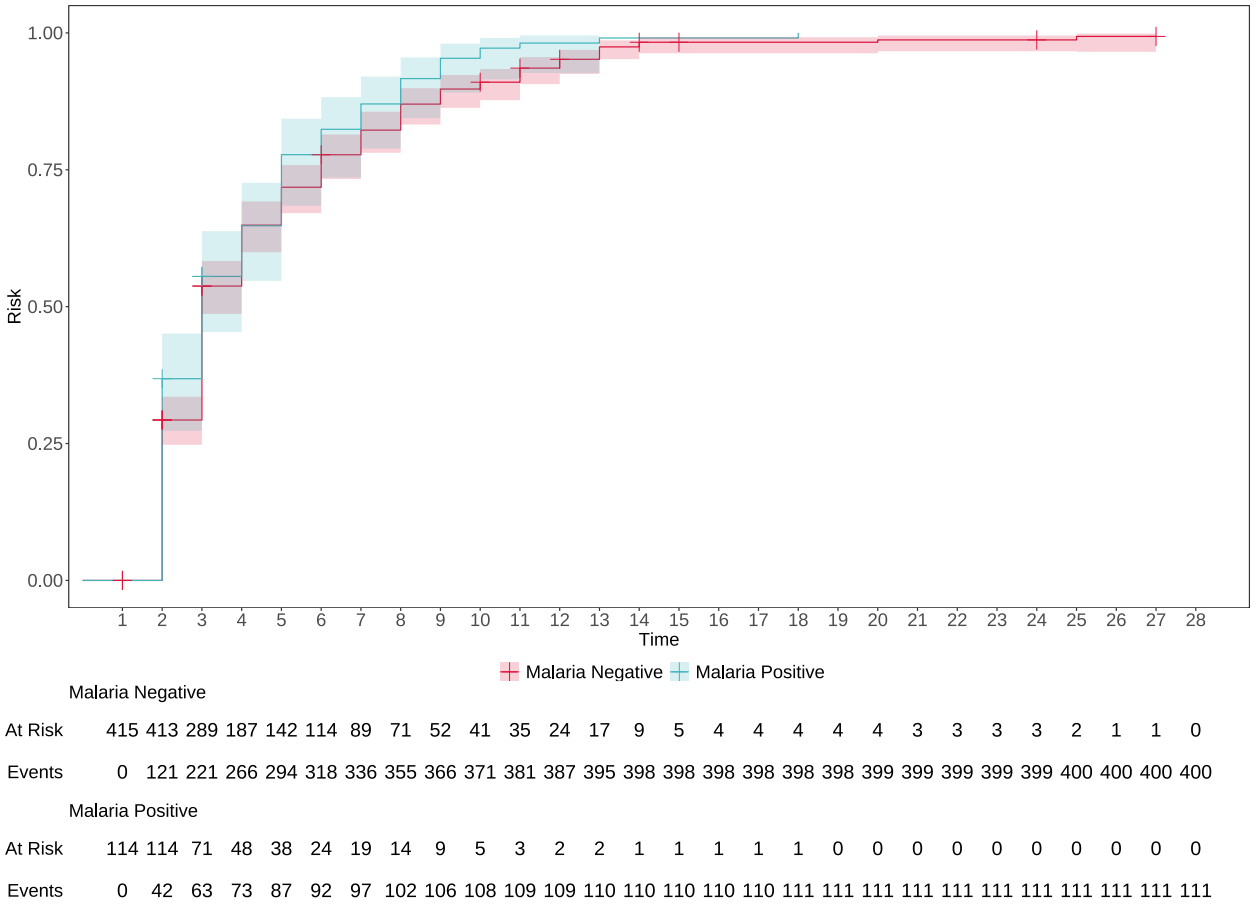 | 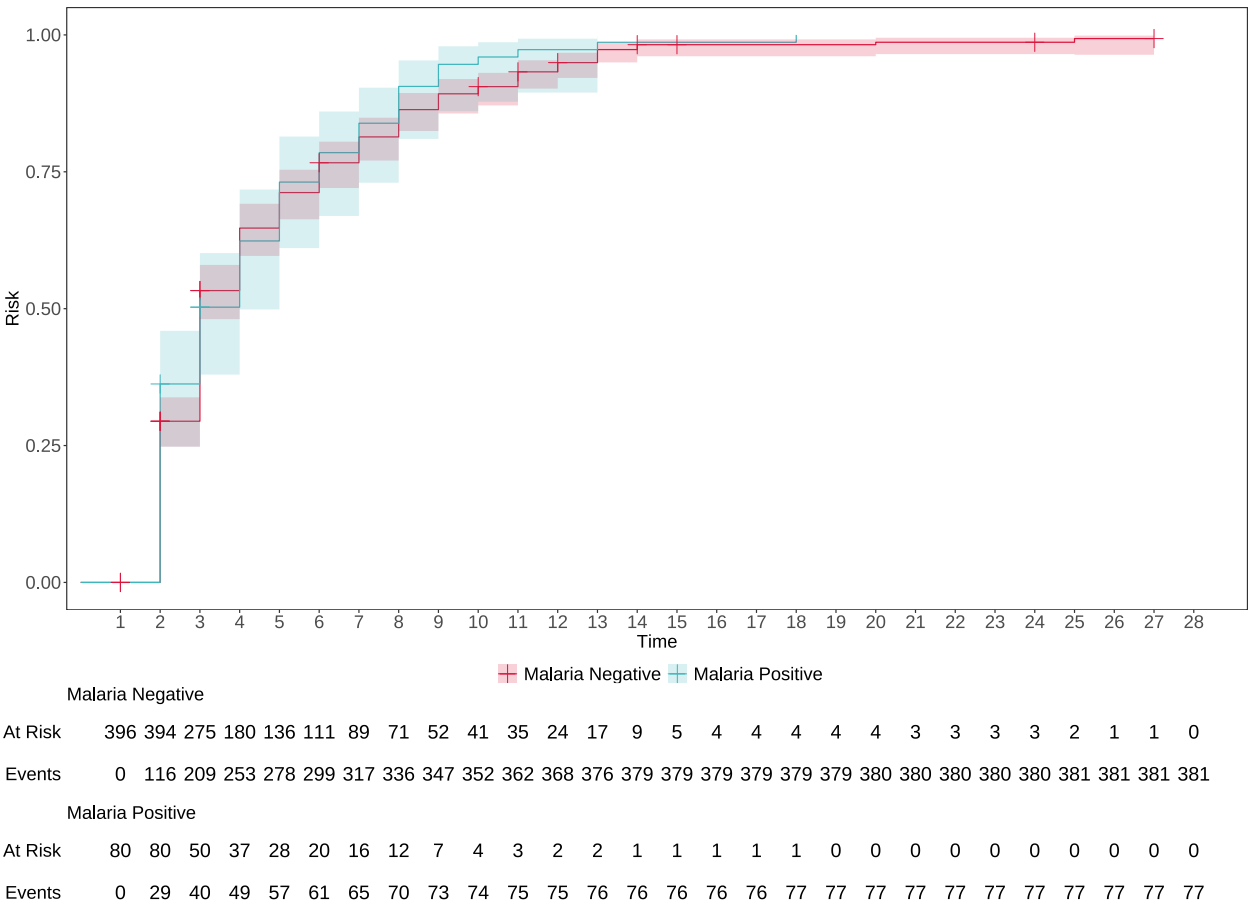 |
| **<15 years** | Notes |
| 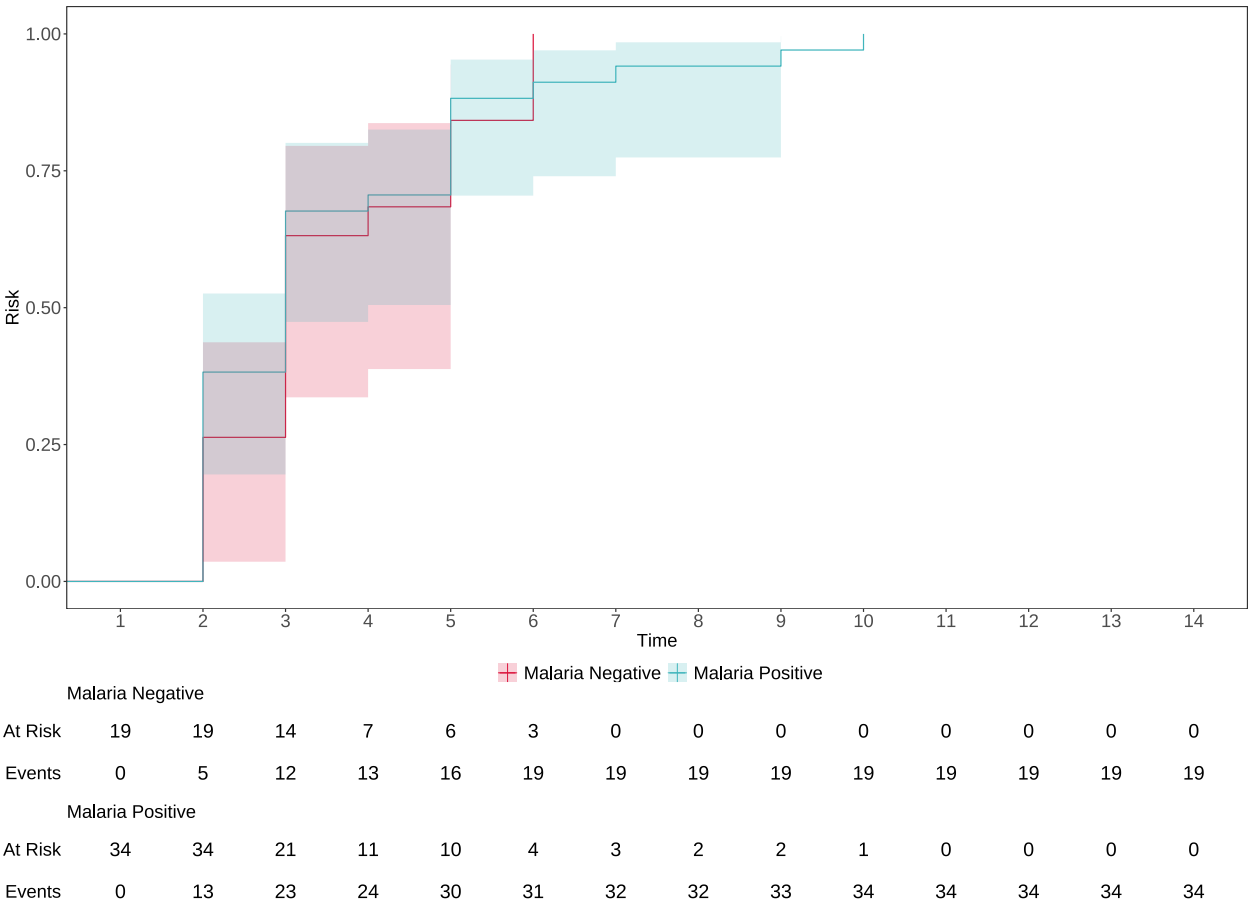 | Hazard ratio and 95% CI for time to clearance of moderate to severe symptoms obtained from cox-regression models adjusted for viral load, age in years, disease severity at enrolment, and country.  Overall: 1.11 (0.90-1.38), p=0.33  ≥ 15 years: 1.16 (0.91-1.48), p=0.24  <15 years: 0.98 (0.56-1.72), p=0.93 |

## Figure-4: Time to SARS-CoV-2 clearance by treatment arm (ITT population)

|  |
| --- |
| 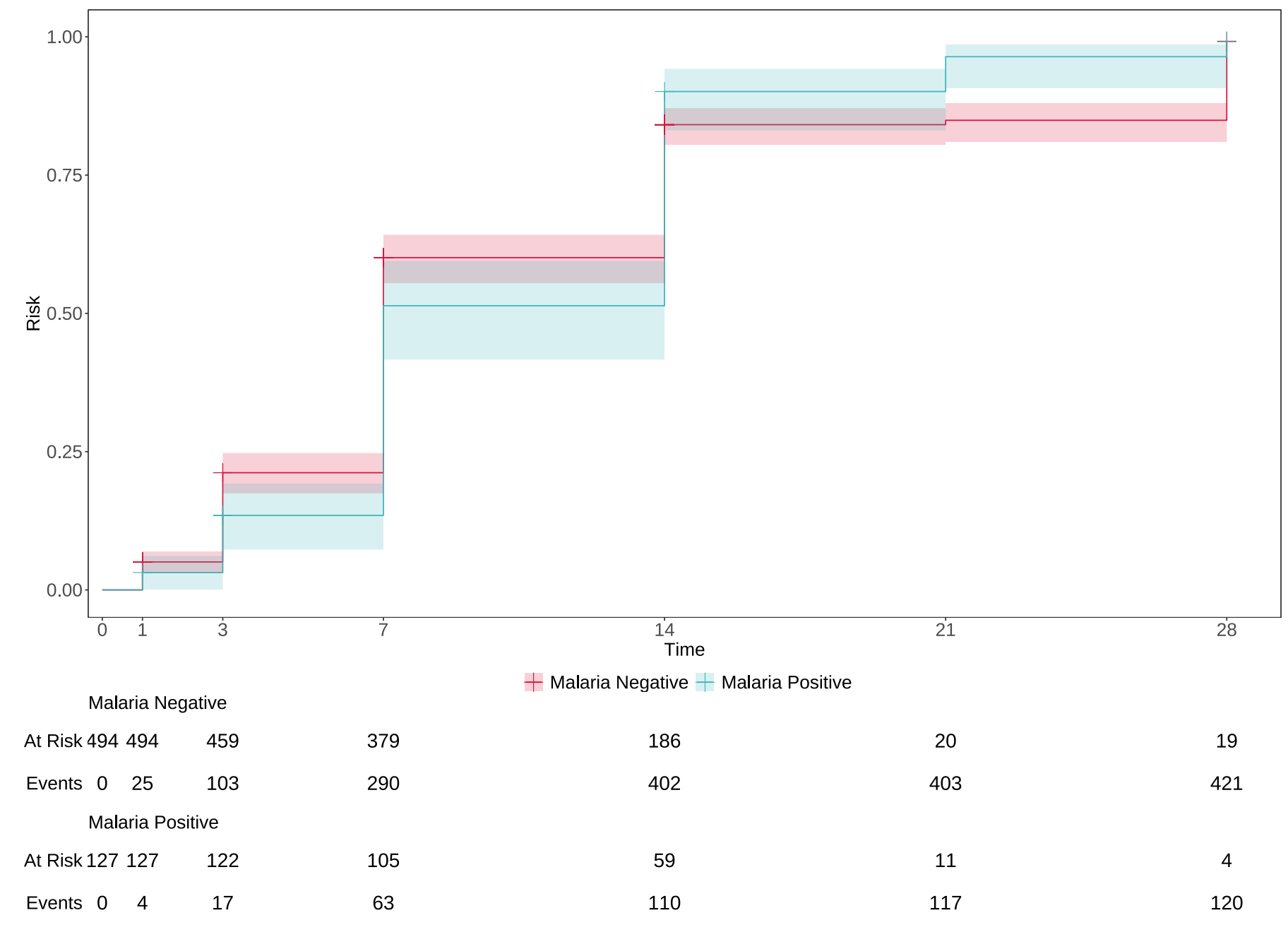 |
| The time to SARS-CoV-2 clearance was slower among malaria than non-malaria patients (adjusted hazard ratioby day 7: 0.71 (95% CI 0.53-0.96), p=0.024. By day 28, the HR was 0.88, 95% CI 0.70-1.09, p=0.24, obtained from cox-regression models adjusted for viral load, age in years, disease severity at enrolment, and country. |

# Tables

## Table-1: Baseline characteristics by malaria group (intention to treat population)

| **Characteristic** | | **Malaria***  **N = 151** | **No malaria**  **N = 591** | **Overall**  **N = 742** | **P-value**† |
| --- | --- | --- | --- | --- | --- |
| **Country** | |  |  |  |  |
|  | Kenya | 95% (144/151) | 82% (483/591) | 85% (627/742) | <0.0001 |
|  | Burkina | 5% (7/151) | 18% (108/591) | 15% (115/742) |  |
| **Age (years)** | | 25.9 (18.7) | 37.5 (17.7) | 35.2 (18.4) |  |
| **Age category** | |  |  |  |  |
|  | < 5 years | 5% (8/151) | <1% (3/591) | 1% (11/742) | <0.0001 |
|  | 5-14 years | 27% (41/151) | 5% (32/591) | 10% (73/742) |  |
|  | >= 15 years | 68% (102/151) | 94% (556/591) | 89% (658/742) |  |
| **Sex male** | | 55% (83/151) | 42% (250/591) | 45% (333/742) | 0.0069 |
| **Received COVID-19 vaccine** | | 4% (6/151) | 4% (26/579) | 4% (32/730) | 0.96 |
| **Years of schooling** | | 7.0 (4.0, 10.5) | 14.0 (9.0, 16.0) | 12.0 (8.0, 16.0) |  |
| **Education attainment** | |  |  |  |  |
|  | None | 57% (86/151) | 16% (93/591) | 24% (179/742) | <0.0001 |
|  | Primary School | 24% (36/151) | 16% (97/591) | 18% (133/742) |  |
|  | Secondary School | 11% (17/151) | 19% (114/591) | 18% (131/742) |  |
|  | Tertiary Training | 8% (12/151) | 49% (287/591) | 40% (299/742) |  |
| **COVID wave (dominant VOC)** | |  |  |  |  |
|  | Earlier variants | 5% (7/151) | 18% (109/591) | 16% (116/742) | <0.0001 |
|  | Alpha wave | 7% (10/151) | 30% (180/591) | 26% (190/742) |  |
|  | Delta wave | 34% (52/151) | 16% (97/591) | 20% (149/742) |  |
|  | Omicon | 54% (82/151) | 35% (205/591) | 39% (287/742) |  |
| **Hospitalised** | |  |  |  |  |
|  | Hospitalised, stable | 0% (0/151) | <1% (2/589) | 0% (2/740) | 0.27 |
|  | Hospitalised, severely ill | 0% (0/151) | 0% (0/589) | 0 (0%) |  |
|  | Hospitalised, to isolate | 0% (0/151) | 1% (8/589) | 1% (8/740) |  |
|  | Not hospitalised | 100% (151/151) | 98% (579/589) | 99% (730/740) |  |
| **Comorbidities** | |  |  |  |  |
|  | Cardiovascular disease | 1% (2/151) | 7% (39/591) | 6% (41/742) | 0.012 |
|  | Diabetes | 0% (0/151) | 2% (13/591) | 2% (13/742) |  |
|  | Immunodeficiency | 4% (6/151) | 5% (27/591) | 4% (33/742) |  |
|  | None | 94% (142/151) | 83% (491/591) | 85% (633/742) |  |
|  | Other | 1% (1/151) | 3% (16/591) | 2% (17/742) |  |
|  | Pregnant/post-partum | 0% (0/151) | 1% (5/591) | 1% (5/742) |  |
| **HIV status self report** | |  |  |  |  |
|  | Unknown | 59% (89/150) | 72% (416/580) | 69% (505/730) | 0.0009 |
|  | Positive | 5% (7/150) | 7% (40/580) | 6% (47/730) |  |
|  | Negative | 36% (54/150) | 21% (124/580) | 24% (178/730) |  |
| **Cigarette smoker** | | 3% (4/151) | 2% (10/591) | 2% (14/742) | 0.66 |
| Data are median (IQR), mean (SD) or n/N (%). Some percentages do not add up to 100 because of rounding. CVD=cardio-vascular disease. HIV=Human Immunodeficiency Syndrome. VOC=Variant of Concern.  * Microscopy or RDT (pLDH or HRP2 band) confirmed malaria. † p-value for difference between malaria and no-malaria groups. | | | | | |

## Table-2: Symptoms and laboratory characteristics by malaria group (intention to treat population)

| **Characteristic** | | **Malaria***  **N = 151** | **No malaria**  **N = 591** | **Overall**  **N = 742** | **P-value**† |
| --- | --- | --- | --- | --- | --- |
| **Documented fever** | | 19% (29/151) | 6% (35/590) | 9% (64/741) | <0.0001 |
| **FLU-PRO+ body system scores** | |  |  |  |  |
|  | Total score‡ | 16.3 (18.5) | 14.6 (15.2) | 14.9 (15.9) | 0.48 |
|  | Total score global | 20.7 (18.9) | 18.8 (15.7) | 19.1 (16.4) | 0.18 |
|  | Eyes | 0.3 (0.9) | 0.3 (0.9) | 0.3 (0.9) | 0.94 |
|  | Throat | 1.0 (2.4) | 1.1 (2.1) | 1.1 (2.1) | 0.082 |
|  | Nose | 2.3 (3.5) | 2.0 (2.9) | 2.0 (3.0) | 0.03 |
|  | Gastrointestinal | 1.0 (2.1) | 0.6 (1.3) | 0.6 (1.5) | 0.032 |
|  | Chest/respiratory | 3.5 (3.6) | 3.6 (3.4) | 3.6 (3.4) | 0.27 |
| **Normal daily activities affected** | | 95% (142/150) | 93% (531/571) | 93% (673/721) | 0.58 |
| **FLU-PRO+ self-reported physical health** | | |  |  |  |
|  | Poor | 9% (13/150) | 5% (27/586) | 5% (40/736) | 0.19 |
|  | Fair | 53% (79/150) | 55% (324/586) | 55% (403/736) |  |
|  | Good | 37% (56/150) | 36% (213/586) | 37% (269/736) |  |
|  | Very good | 1% (2/150) | 3% (19/586) | 3% (21/736) |  |
|  | Excellent | 0% (0/150) | 1% (3/586) | 0% (3/736) |  |
| **FLU-PRO+ symptom severity** | |  |  |  |  |
|  | No symptoms | 3% (5/150) | 4% (24/587) | 4% (29/737) | 0.42 |
|  | Mild | 29% (43/150) | 36% (213/587) | 35% (256/737) |  |
|  | Moderate | 64% (96/150) | 56% (330/587) | 58% (426/737) |  |
|  | Severe | 4% (6/150) | 3% (19/587) | 3% (25/737) |  |
|  | Very Severe | 0% (0/150) | 0% (1/587) | 0% (1/737) |  |
| **FLU-PRO+ severity category** | |  |  |  |  |
|  | Moderate or severe | 68% (102/150) | 60% (350/587) | 61% (452/737) | 0.074 |
|  | None-mild | 32% (48/150) | 40% (237/587) | 39% (285/737) |  |
| **O2 saturation** | | 96.3 (2.3) | 96.3 (4.1) | 96.3 (3.8) | 0.096 |
| **Malaria test positivity** | |  |  |  |  |
|  | Negative | 0% (0/151) | 100% (591/591) | 80% (591/742) | NA |
|  | Pos. by HRP2-band only | 37% (56/151) | 0% (0/591) | 8% (56/742) |  |
|  | Pos. by microscopy/pLDH | 63% (95/151) | 0% (0/591) | 13% (95/742) |  |
| **SARS-CoV-2 PCR*** | |  |  |  |  |
|  | Inconclusive | 0% (0/151) | 1% (6/584) | 1% (6/735) | 0.41 |
|  | Negative | 19% (28/151) | 20% (117/584) | 20% (145/735) |  |
|  | Positive | 81% (123/151) | 79% (461/584) | 79% (584/735) |  |
| **SARS-CoV-2 viral load, copies** | | |  |  |  |
|  | All | 2,260  (241-18,073) | 3,001  (225-39,782) | 2,862  (229-34,332) | 0.2 |
|  | < 15 years | 735  (78-11,462) | 2,576  (343-22,542) | 1,658  (82-14,503) | 0.3 |
|  | >= 15 years | 2,667  (365-27,102) | 3,013  (222-39,794) | 2,976  (248-38,589) | 0.6 |
| Data are median (IQR), mean (SD) or n/N (%). Some percentages do not add up to 100 because of rounding. CVD=cardio-vascular disease. FLU-PRO+=InFLUenza Patient-Reported Outcome (a self-administered patient-reported outcome measure [PRO]). HIV=Human Immunodefiency Syndrome. HRP2=Histidine-rich Protein 2. PCR=polymerase chain reaction. pLDH=*Plasmodium* Lactate Dehydrogenase. O2=Oxygen. SARS-CoV-2=severe acute respiratory syndrome coronavirus. VOC=Variant of Concern.  * Microscopy or RDT (pLDH or HRP2 band) confirmed malaria. † p-value for difference between malaria and no-malaria groups. ‡The FLU-PRO-plus total score is computed as a mean score across all 32 items comprising the FLU-PRO-Plus instrument. Total scores can range from 0 (symptom free) to 4 (very severe symptoms) . | | | | | |

## Table-3: Symptom resolution (intention to treat population) *

|  | | Malaria | No malaria | ES | Adjusted RR IRR, or HR (95% CI) | p-value |
| --- | --- | --- | --- | --- | --- | --- |
| **Symptoms (any)** | |  |  |  |  |  |
| All ages | Day 7 | 62% (90/146) | 58% (331/566) | RR | 1.02 (0.79-1.33) | 0.86 |
|  | Day 14 | 13% (18/144) | 13% (73/551) | RR | 1.19 (0.69-1.94) | 0.50 |
|  | days with symptoms**†** | 52% (1068/2063) | 55% (4380/7955) | IRR | 0.95 (0.86-1.05) | 0.31 |
|  | Time to clearance | 9 (5-13) | 10 (6-13) | HR | 1.14 (0.91-1.42) | 0.26 |
| ≥15 yrs | Day 7 | 68% (66/97) | 59% (313/531) | RR | 1.08 (0.80-1.44) | 0.63 |
|  | Day 14 | 15% (14/95) | 13% (69/516) | RR | 1.32 (0.73-2.19) | 0.32 |
|  | days with symptoms‡ | 55% (766/1381) | 56% (4150/7465) | IRR | 0.98 (0.88-1.09) | 0.66 |
|  | Time to clearance | 9 (6-13) | 10 (7-13)) | HR | 1.09 (0.85-1.41) | 0.48 |
| <15 yrs | Day 7 | 49% (24/49) | 51% (18/35) | RR | 0.82 (0.51-1.34) | 0.4 |
|  | Day 14 | 8% (4/49) | 11% (4/35) | RR | 0.48 (0.10-1.99) | 0.3 |
|  | days with symptoms**†** | 44% (302/682) | 47% (230/490) | IRR | 0.83 (0.62-1.11) | 0.21 |
|  | Time to clearance | 8 (5-12) | 10 (5-12) | HR | 1.36 (0.72-2.58) | 0.34 |
| **Moderate to severe symptoms (ITT)†** | | | | | | |
| All ages | Day 7 | 8% (11/146) | 7% (39/566) | RR | 1.53 (0.74-3.16) | 0.25 |
|  | Day 14 | <1% (1/144) | <1% (5/551) | RR | NA | NA |
|  | days with symptoms | 13% (267/2063) | 12% (975/7955) | IRR | 0.95 (0.86-1.05) | 0.31 |
|  | Time to clearance | 2 (2-5) | 2 (1-4) | HR | 1.11 (0.90-1.38) | 0.33 |
| ≥15 yrs | Day 7 | 10% (10/97) | 7% (39/531) | RR | 1.61 (0.76-3.42) | 0.22 |
|  | Day 14 | 1% (1/95) | <1% (5/516) | RR | 0.49 (0.01-41.71) | 0.75 |
|  | days with symptoms‡ | 15% (202/1381) | 13% (937/7465) | IRR | 0.98 (0.88-1.09) | 0.67 |
|  | Time to clearance | 2 (2-5) | 2 (1-4) | HR | 1.16 (0.91-1.48) | 0.24 |
| <15 yrs | Day 7 | 2% (1/49) | 0% (0/35) | RR | NA | 1.00 |
|  | Day 14 | 0% (0/49) | 0% (0/35) | RR | NA | 1.00 |
|  | days with symptoms‡ | 10% (65/682) | 8% (38/490) | IRR | 0.83 (0.62-1.11) | 0.21 |
|  | Time to clearance | 2 (1-3) | 2 (1-3) | HR | 0.98 (0.56-1.72) | 0.93 |
| **Illness affects normal activities (ITT)†** | | | | | | |
| All ages | Day 0 | 95% (142/150) | 93% (531/571) | RR | 1.01 (0.82-1.26) | 0.90 |
|  | Day 7 | 47% (69/146) | 63% (352/563) | RR | 0.80 (0.59-1.07) | 0.13 |
|  | Day 14 | 10% (14/144) | 17% (93/551) | RR | 1.03 (0.56-1.75) | 0.92 |
| ≥15 yrs | Day 0 | 96% (98/102) | 93% (498/536) | RR | 1.01 (0.79-1.29) | 0.92 |
|  | Day 7 | 48% (47/97) | 63% (331/528) | RR | 0.81 (0.58-1.14) | 0.22 |
|  | Day 14 | 12% (11/95) | 17% (89/516) | RR | 1.13 (0.59-2.20) | 0.71 |
| <15 yrs | Day 0 | 92% (44/48) | 94% (33/35) | RR | 0.97 (0.62-1.53) | 0.90 |
|  | Day 7 | 45% (22/49) | 60% (21/35) | RR | 0.75 (0.49-1.14) | 0.17 |
|  | Day 14 | 6% (3/49) | 11% (4/35) | RR | 0.54 (0.11-2.29) | 0.39 |
| **Poor or fair physical health (ITT)†** | | | | | | |
| All ages | Day 0 | 61% (92/150) | 60% (351/586) | RR | 0.92 (0.70-1.20) | 0.53 |
|  | Day 7 | 12% (18/146) | 15% (82/563) | RR | 0.74 (0.42-1.22) | 0.27 |
|  | Day 14 | <1% (1/144) | 1% (8/555) | RR | 0.73 (0.04-4.09) | 0.75 |
| ≥15 yrs | Day 0 | 59% (60/102) | 60% (330/551) | RR | 0.87 (0.64-1.18) | 0.36 |
|  | Day 7 | 14% (14/97) | 14% (75/528) | RR | 0.88 (0.47-1.49) | 0.65 |
|  | Day 14 | 1% (1/95) | 2% (8/520) | RR | 0.77 (0.04-4.34) | 0.80 |
| <15 yrs | Day 0 | 67% (32/48) | 60% (21/35) | RR | 1.11 (0.80-1.60) | 0.54 |
|  | Day 7 | 8% (4/49) | 20% (7/35) | RR | 0.41 (0.11-1.25) | 0.13 |
|  | Day 14 | 0% (0/49) | 0% (0/35) | RR | NA | >0.99 |
| CI=95% confidence interval. ES=effect size. HR=hazard ratio. IRR=rate ratio, IQR=interquartile range. MD=mean difference. NA=Not available. RR=risk ratio.  * Intention to treat population involving all enrolled participants. †All analyses were adjusted for country and enrolment age, disease severity, and viral load. ‡ Rate in person days calculated as the number of days with self-reported symptoms assessed by FLU-PRO-Plus divided by the total number of days with valid FLU-PRO-Plus data. § Not available because this could not be estimated because of the small number of events. All analyses were adjusted for country and enrolment age, disease severity, and viral load. | | | | | | |

## Table-4: SARS-CoV-2 clearance by malaria status) (modified intention to treat population)*

| SARS-CoV-2 | | Malaria | No malaria | ES | Adjusted HR or RR (95% CI) † | p-value |
| --- | --- | --- | --- | --- | --- | --- |
| All ages | % cleared by D3 | 14% (17/122) | 27% (126/473) | RR | 0.82 (0.45-1.49) | 0.52 |
|  | % cleared by D7 | 48% (58/120) | 53% (253/477) | RR | 0.79 (0.58-1.08) | 0.14 |
|  | % cleared by D14 | 86% (105/122) | 73% (347/473) | RR | 0.99 (0.78-1.25) | 0.92 |
|  | % cleared by D28 | 99% (116/117) | 98% (375/381) | RR | 0.99 (0.79-1.24) | 0.95 |
|  | Time to viral clearance D7 | 7 (7-7) | 7 (7-7) | HR | 0.69 (0.51-0.94) | 0.017 |
|  | Time to viral clearance by D28 (median, IQR) | 7 (7-14) | 7 (7-14) | HR | 0.83 (0.67-1.04) | 0.11 |
|  | Viral load at D7 (median, IQR) | 125 (20-1,518) | 65 (12-1,031) |  |  | 0.55 |
| ≥ 15 yrs | % cleared by D3 | 14% (12/86) | 27% (121/450) | RR | 0.70, 0.33-1.46) | 0.34 |
|  | % cleared by D7 | 48% (40/84) | 53% (240/454) | RR | 1.01 (0.78-1.31) | 0.93 |
|  | % cleared by D14 | 86% (74/86) | 73% (329/450) | RR | 0.99 (0.77-1.28) | 0.95 |
|  | % cleared by D28 | 99% (82/83) | 99% (356/360) | RR | 1.08 (0.80-1.44) | 0.63 |
|  | Time to viral clearance D7 | 7 (7-7) | 7 (7-7) | HR | 0.91 (0.68-1.22) | 0.51 |
|  | Time to viral clearance by D28 (median, IQR) | 7 (7-14) | 7 (7-14) | HR | 0.88 (0.54-1.49) | 0.62 |
|  | Viral load at D7 (median, IQR) | 311 (36-1,834) | 74 (12-1,125) |  |  | 0.14 |
| <15 yrs | % cleared by D3 | 14% (5/36) | 22% (5/23) | RR | 0.64 (0.20-2.07) | 0.44 |
|  | % cleared by D7 | 50% (18/36) | 57% (13/23) | RR | 1.10 (0.87-1.49) | 0.46 |
|  | % cleared by D14 | 86% (31/36) | 78% (18/23) | RR | 1.00 (0.58-1.71) | >0.99 |
|  | % cleared by D28 | 100% (34/34) | 90% (19/21) | RR | 0.55 (0.27-1.14) | 0.11 |
|  | Time to viral clearance D7 | 7 (7-7) | 7 (7-7) | HR | 0.54 (0.30-0.99) | 0.046 |
|  | Time to viral clearance by D28 (median, IQR) | 7 (7-14) | 7 (7-14) | HR | 0.88 (0.54-1.49) | 0.62 |
|  | Viral load at D7 (median, IQR) | 45 (6-263) | 53 (21-481) |  |  | 0.65 |
| CI=95% confidence interval. ES=effect size. HR=hazard ratio. IQR=interquartile range. RR=risk ratio.  * Modified intention to treat population comprising those with valid SARS-CoV-2 PCR at enrolment. †All analyses were adjusted for country, enrolment age, disease severity, and viral load. | | | | | | |

# Supplementary appendix

Supplement to: Barsosio H, Tangara B, Marlais T, et al. Malaria as a risk factor for COVID-19 in western Kenya and Burkina Faso (MALCOV): an observational cohort study

Table of Contents

[Supplemental methods 23](#_Toc160275823)

[Supplement 1: Definition of endpoints 23](#_Toc160275824)

[Supplement 2: Laboratory procedures 23](#_Toc160275825)

[Supplement 3: Statistical methods 24](#_Toc160275826)

[Supplemental references 24](#_Toc160275827)

##### Supplemental methods

###### Supplement 1: Definition of endpoints

All outcome variables are assessed after enrolment and exclude the value at enrolment before randomisation.

Efficacy endpoints

Primary endpoints

Defined as the cumulative proportion (n/N, %) of participants in the mITT population (see Analysis Populations, page 24, below) who had evidence of SARS-CoV-2 clearance in the first 7 days. Cleared was defined as meeting the definition of SARS-CoV-2 negative by PCR (see SARS-CoV-2 positivity at enrolment, page 24, below) on or before day 7. If RT-PCR results were missing or inconclusive on day 7, then the individual had to meet the negative definition of SARS-CoV-2 by RT-PCR on day 3 with no subsequent positive results.

Secondary endpoints

Cumulative clearance of SARS-CoV-2 by days 14, 21 and 28.

Defined as per the primary outcome but considering days 3, 14, 21 and 28 with window periods.

FLU-PRO+: Total days with self-reported moderate-to-severe disease severity

The total number of days with moderate to severe disease by day 7 and day 14.

FLU-PRO+: Total days with any symptoms

The total number of days with any self-reported symptom by day 7 and day 14.

FLU-PRO+: Time to clearance of self-reported moderate-to-severe disease severity

Number of days until clearance of moderate to severe symptoms in those reporting moderate to severe symptoms at enrolment or beyond.

FLU-PRO+: Total days with any symptom

Number of days until clearance of any symptom in those reporting symptoms at enrolment or beyond.

FLU-PRO+: Mean daily total and domain scores in the first 7 days post enrolment^1^

The conceptual framework for pooling FLU-Pro symptoms into system-level symptoms to derive the domain is described by Richard et al.^1.^ Daily total scores are derived by calculating the mean score of the symptoms for each day in each of the 7 symptom domains (throat, nose, eyes, gastrointestinal, respiratory, systemic, senses).

###### Supplement 2: Laboratory procedures

Blood smears

Blood smears were stained with Giemsa and read in duplicate by certified microscopists. A blood smear was considered negative when the examination of 100 high-power fields did not reveal the presence of asexual parasites. A third microscopist would read any discrepancies between the first and second reader. Blood smear results were considered discrepant if one reader scored them as negative and another as positive.

RT-PCR for SARS-CoV-2

SARS-CoV-2 RNA was detected by real-time reverse transcription polymerase chain reaction (real-time RT-PCR) using primers and probes targeting the N gene^2^ and the Luna Universal Probe One-Step RT-qPCR Kit (New England Biolabs, Hitchin, UK). Each 20 µl reaction contained 400 nM each primer and 200 nM probe. Reverse transcription was performed at 55ºC for 10 minutes, followed by denaturation at 95ºC, 60 seconds, followed by thermal cycling for 40 cycles of 95ºC for 10 seconds and 55ºC for 55 seconds. A SARS-CoV-2 RNA-detected result was defined as an exponential fluorescent curve that crossed the threshold within 36 cycles (cycle threshold [Ct] < 36). Viral load was estimated as copies per reaction by interpolation of sample Ct values against those of a quantified in vitro transcript (IVT) RNA standard. Briefly, partial SARS-CoV-2 N gene sequence was cloned into a vector containing a T7 promoter, using which IVT RNAs were produced. Purified IVT RNA was quantified and used to prepare the standard, which was run on every assay plate. The data for the standard was compiled separately for each country to account for local variation, and viral load estimates were interpolated from these reference curves respectively by country.

PCR for malaria

Dried blood spots for PCR were taken at enrolment in all participants . DNA was extracted from dried blood spots (50 microL) using a commercial DNA extraction kit (QIAamp DNA Mini Kit, 51306, Qiagen, UK) eluted in 150 ul final volume. The resulting DNA extracts were tested for the presence of *P. falciparum* DNA by real-time PCR using the qPCR method and conditions as described by Hermsen et al. (2001)￼￼ targeting the 18S rRNA gene of *P. falciparum.*

Reaction conditions consited of primers at 300 nM each and the TaqMan probe at 150 nM, 2.5 uL of DNA sample and TaqMan universal mastermix, with a final volume of 20 uL. Standard curve from a 3D7 *P. falciparum* culture, and no-template negative controls were included in each run. Cycling conditions were 95°C for 1 min, followed by 40 cycles of 95°C for 20 s and 58°C for 1 min. ￼￼￼The method has a lower limit of detection of 20 parasites per m￼blood.[Hermsen 2001￼￼

###### Supplement 3: Statistical methods

SARS-CoV-2 positivity at enrolment

All patients were enrolled based on either a positive SARS-CoV-2 RT-PCR test or a positive rapid antigen test for SARS-CoV-2 from a mid-nasal swab.

SARS-CoV-2 positivity at enrolment was defined as either:

1. Positive SARS-CoV-2 RT-PCR
2. Negative SARS-CoV-2 RT-PCR at enrolment, but positive rapid antigen test.

SARS-CoV-2 RT-PCR positivity

SARS-CoV-2 RT-PCR positivity was defined based on the cycle threshold (CT) value as follows:

- Positive: CT value of <36
- Inconclusive: CT value 36 to 40
- Negative: no amplification
- Missing: No result

Analysis Populations

Intention to treat population (ITT)

All enrolled participants. The ITT population was the analysis population for the symptom resolution and disease progression endpoints.

Modified intention to treat population

All participants in the ITT population with a SARS-CoV-2 RT-PCR positive test at enrolment. The mITT population was the analysis population for the SARS-CoV-2 clearance endpoints.

##### Supplemental references

1. Richard SA, Epsi NJ, Lindholm DA, Malloy AM, Maves RC, Berjohn CM, Lalani T, Smith AG, Mody RM, Ganesan A, 2022. COVID-19 patient-reported symptoms using FLU-PRO Plus in a cohort study: Associations with infecting genotype, vaccine history, and return to health. Open forum infectious diseases: Oxford University Press, ofac275.

2. Lu X, Wang L, Sakthivel SK, Whitaker B, Murray J, Kamili S, Lynch B, Malapati L, Burke SA, Harcourt J, Tamin A, Thornburg NJ, Villanueva JM, Lindstrom S, 2020. US CDC Real-Time Reverse Transcription PCR Panel for Detection of Severe Acute Respiratory Syndrome Coronavirus 2. Emerg Infect Dis 26: 1654-65.

3. Saidi Q, Minja D, Njau J, Hansson H, Kavishe R, Alifrangis M, 2021. Ultrasensitive qPCR-Based Detection of Plasmodium falciparum in Pregnant Women Using Dried Blood or Whole Blood Pellet Samples Processed through Different DNA Extraction Methods. Am J Trop Med Hyg 106: 846-849.

4. Hofmann N, Mwingira F, Shekalaghe S, Robinson LJ, Mueller I, Felger I, 2015. Ultra-sensitive detection of Plasmodium falciparum by amplification of multi-copy subtelomeric targets. PLoS Med 12: e1001788.
