# Supplementary material for: Self-reported COVID-19 severity among persons with tuberculosis infection in western Kenya, 2021
Source: PLOS Glob Public Health. 2025 Apr 30;5(4):e0004372. doi: 10.1371/journal.pgph.0004372 (PMC12043119; doi:10.1371/journal.pgph.0004372)
Supplement: Attachment — Submitted filename: PLoS Glob Pub Health REBUTTAL LETTER.docx [file pgph.0004372.s004.docx]

Taraz Samandari

2520 Kimbolton Dr.

College Station, TX 77845

January 16, 2025

Dr. María Elvira Balcells, M.D., MSc
Academic Editor

PLOS Global Public Health

1875 Mission Street

Suite 103 #188 San Francisco, CA 94103

United States

Re. REBUTTAL LETTER, PGPH-D-24-02424 “Severity of COVID-19 among persons with tuberculosis infection in western Kenya, 2021PLOS Global Public Health”

Dear Dr. Balcells,

We thank you and the reviewers for your comments. They have strengthened the paper.

In addition to this rebuttal letter, we have provided the following items: a revised manuscript, a track changes version of the manuscript, a revised figure 1, the parent study protocol which includes the sub-study (version Oct 15, 2021) and a manuscript of the parent study which will be published as a preprint early in 2025.

Sincerely,

Taraz Samandari, MD PhD

AUTHORS’ RESPONSE TO THE REVIEW

1. The Editor stated “To accurately reflect this distinction and prevent potential misinterpretation, the title, abstract, and main text need revision and replacing of "COVID-19 severity" with "COVID-19 symptom severity." The Discussion section also requires addressing this important differentiation, emphasizing that while clinical severity scales measure the impact of the disease on health outcomes, symptom severity scales capture the patient's subjective experience of the illness.”

**RESPONSE**: We have modified the term “COVID-19 severity” with “self-reported COVID-19 severity” throughout the manuscript and highlighted the difference between the self-reported outcomes and objective severity of disease in the Limitations.

Additional Editor Comments (if provided):

1. Introduction: Provide a brief description of the parent MALCOV study design for context.

**RESPONSE:** In order not to distract from the Introduction, we describe the parent study, MALCOV, early in the Methods section.
2. Screening and Recruitment: The strategy used to screen and recruit participants for this cohort is unclear and requires elaboration.

**RESPONSE:** After notification of County health authorities and in communication with community focal persons in charge of community health workers, our study recruiters screened potential participants attending several types of facilities. In the Methods we state that participants were recruited from “(1) a patient attending COVID-19 test centers or clinics testing for COVID-19, or (2) admitted to the COVID-19 isolation or quarantine facilities, or (3) a contact of a confirmed or suspected case as part of contact tracing, or (4) among those targeted for testing due to the high-risk nature of their work (i.e., frontline healthcare workers).” We added a sentence to elaborate on our study recruiters’ strategy. In addition, due to Reviewer #2’s comments, we have shown the proportion of participants recruited from each of these venues.
3. Exclusion Criteria: Were there any exclusion criteria? If so, please specify. Additionally, clarify why active TB was not considered an exclusion criterion.
**RESPONSE:** The exclusion criteria were: if a potential participant was under 6 months of age, unwilling or unable to give consent, or anticipated to be unable to adhere to the follow-up schedule. These have been added to the manuscript. Active TB was not an exclusion criterion because the parent study was aimed at studying the intersection of malaria and COVID-19.

4. Follow-Up: What was the follow-up strategy employed for this study? Please provide details.
**RESPONSE:** Each participant was followed up for 12 months after enrolment (the study itself lasted 18 months). Study recruiters maintained contact with participants initially through daily phone calls in the first 2 weeks, then by using text messaging in order to remind them of scheduled visits at study clinics. Appointment cards were provided to participants to show them their upcoming visits, and a participant diary was provided for the participant to record his/her symptoms. We had added this information to the Methods.

5. SARS-CoV-2 PCR Follow-Up: Clarify the relevance of regular SARS-CoV-2 PCR follow-up for this cohort in relation to the study objectives. If it is not relevant, consider removing it from the Methods section.
**RESPONSE:** The SARS-CoV-2 PCR after initial confirmation of infection with the virus was not relevant to this sub-study and has been removed. Thank you.

6. Parent Study: The NCT04695197 identifier corresponds to the parent study, not this one. Ensure this is clearly stated.
**RESPONSE:** We have added a statement to clarify that the identifier refers to the parent study.

7. Sample Size Calculation: Include details of the sample size calculation to support the study's statistical validity.
**RESPONSE:** As per the forthcoming preprint of the main paper (Barsosio et al), the sample size calculation was aimed at the parent study’s objective to determine the effect of malaria and malaria treatment on SARS-CoV-2 viral clearance and COVID-19 disease progression. This led to a sample size of 708. The calculation is available in the protocol which is now added to this submission among supplementary materials. The TBI sub-study drew from a convenience sample of participants enrolled in the main study and so a separate sample size was not conducted.

8. Results: Please provide the mean age of participants along with the range, and also provide in outcomes the hospitalization proportion.
**RESPONSE:** The average age of the 373 participants included was 34.6 years (range 8-80). Five participants were hospitalized (days hospitalized: median 2, range 1-11). The one participant who died was among these five. These have been added to the manuscript.

9. Style: Avoid starting sentences with numbers to maintain formal scientific writing standards.
**RESPONSE:** We edited the manuscript accordingly.

Reviewer #1: The study is interesting, given that current evidence supports the adverse effects of TB/SARS-CoV-2 co-infection or a history of active TB and COVID-19. However, there needs to be more evidence regarding the effect of TBI on the development of severe COVID-19; despite this, some issues should be considered to improve the clarity of the study and its scope.

The study presents a possible selection bias due to excluding people from areas with limited access to the health system (n=231), which limited access to the QTF test. These individuals could have experienced severe COVID-19 or died without adequate diagnosis, affecting the representativeness of the sample. It would be helpful to compare the baseline characteristics of the included and excluded participants to detect possible systematic differences that could influence the findings.

**RESPONSE:** In order to address Reviewer 1’s concern, we compared the following demographics in the 387 included in the QFT sub-study and those who were excluded from the sub-study: sex, age<50, completion of secondary education, unemployed, hypertensive, HIV+, a history of TB, death during the course of the study, loss to follow up during the study, and withdrawing consent during the study. The following comparisons were statistically different among those included vs excluded. Included persons had a higher odds of being male (OR 1.59, p=0.004), higher odds of self-reporting HIV+ status (OR 2.59, p=0.0096), and lower odds of becoming lost to follow up over the full 12 months of the study (OR 0.29, p=0.018). It is not unexpected to find differences between participants included and excluded in a study, particularly in one that incorporated a clinical trial.

In addition, only 22 cases of severe COVID-19 were captured, which limits the statistical power and the representativeness of the study, and these aspects should be discussed in the limitations section.

**RESPONSE:** We have added the likelihood of lack of representativeness in our limitations which is an inherent feature of a convenience cohort. We further note that our question was not what proportion of persons with severe COVID-19 had TBI but the reverse. Our question was, “If they were QFT positive, were they more likely to develop severe COVID-19 than persons who were QFT negative?” For this reason, we did not – for example – recruit among persons hospitalized for COVID-19.

The possible loss to follow-up needs to be mentioned, which could introduce additional biases in the results, especially regarding mortality and development of active TB.

**RESPONSE:** Ten of 373 participants lacked daily follow-up during the initial 14 days of FLUPRO+ observation (average of 8.2 days missing, range 1-14). The first 14 days, the period during which a COVID-19 infected person is most likely to develop severe disease, were the primary focus of this analysis. Stated otherwise, we had 98.4% completion of follow up despite the challenges related to the national restrictions in the movement of citizens during the pandemic’s peak. We have added this to the manuscript as a strength of the study. As for the follow-up period relating to active TB (an incidental and secondary opportunistic objective of the study), we state in our Results that “Median follow-up of the cohort was 105 days (range 0-368).”

It is unusual that the only variable significantly associated with severe COVID-19 is age >50 years, as previous studies have also pointed to male sex and socioeconomic disadvantage as significant risk factors. This finding could be due to a selection bias derived from the convenience sampling described in the methodology. In addition, the reliance on self-reporting to assess comorbidities and outcomes increases the risk of misclassification bias.

**RESPONSE:** Regarding the risk factor of male sex as a risk factor for severe COVID-19, we observed a trend towards less severe COVID-19 in women with crude OR 0.87, P=0.76. This magnitude is similar to what has been observed in meta-analyses (see for example https://pubmed.ncbi.nlm.nih.gov/35514606/ or https://pubmed.ncbi.nlm.nih.gov/32860457/). However, our study was not powered to determine significance at this level of difference; studies that do show significant differences between the sexes typically required thousands of cases. Regarding socioeconomic status and the risk for severe disease, this has rarely been documented in the African continent but rather has been well documented in affluent countries (https://www.thelancet.com/journals/lanpub/article/PIIS2468-2667(22)00223-7/fulltext). Our participants who consisted almost exclusively of peri-urban, lower income persons in rural, western Kenya, were therefore unlikely to show differences in socioeconomic disadvantage.

In particular, self-reporting of HIV may be underestimated due to the associated stigma, and it is not specified whether participants were immunocompromised or on antiretroviral treatment with controlled immunity. This lack of detail limits the interpretation of the impact of HIV on the results, so it is recommended to clarify this variable to improve the precision of its assessment (The methodology states that information on medications received by participants was accessed).

**RESPONSE:** We agree with Reviewer #1. We have modified the limitations which now reads: “A third limitation is that while HIV co-infection is an established risk factor for severe COVID-19, HIV status was self-reported and unknown in 8.6% of our cohort, so we could not establish whether it was an independent risk factor for self-reported severe COVID-19. Our multivariable analysis that included two variables – HIV and age ≥50 years – showed that age was the main risk factor. A large study of hospitalized HIV-infected patients from around the world found that patients aged 45-75 years had increased odds of severe COVID-19 which again emphasizes the importance of age. Hypertension and diabetes were self-reported conditions and so – just as with HIV status – we cannot exclude a risk of a misclassification error for these variables.”

Since the pediatric population is at lower risk for severe COVID-19 and TBI, it would be beneficial to stratify the analysis by age groups (adults and children) to avoid a possible distortion in the magnitude of the association between TBI and severe COVID-19, as well as in the subsequent development of active TB after viral infection.

**RESPONSE:** We agree with Reviewer #1 that persons ≤15 years of age (children) in Africa typically have low prevalences of TBI and this prevalence increases greatly after the age of 15 in such communities. We also agree that rates of severe COVID-19 affect persons with advanced ages far more than children. We examined our data to assess the Reviewer’s concern. There were 40 persons in our cohort ≤15 years of age; 3 were QFT+ and 37 were QFT-. Only one of the 3 QFT+ children (33%) had severe COVID-19 and none of the 37 QFT- (0%) children experienced severe COVID-19. These low numbers do not permit a meaningful stratification. Perhaps the hypothesis being proposed is that if children were excluded from the stratification of TBI, the association with severe COVID-19 might be stronger. We found that the odds of severe COVID-19 in ≥50 year-olds increased slightly from 3.73 (95%CI 1.47,9.07) (Table 2) to 4.24 (95%CI 1.65, 10.9) by excluding ≤15 year old participants, with 95%CIs overlapping considerably. Overall, our cohort aimed at being inclusive of all participants, and our models included adjustment for both age group and TBI.

At first, it seems that only the association between TBI and severe COVID-19 as an outcome will be evaluated; however, given the prospective nature of the study, an attempt was made to evaluate the reactivation of TBI post-COVID-19, but there is no statistical power to support this analysis, therefore the conclusion that there is no evidence of the association between COVID-19 infection (at all levels of severity) and the risk of TB activation seems premature due to the substantial imbalance between the study groups: 369 cases of COVID-19 versus only four asymptomatic controls. This imbalance limits the statistical power of the analysis and could affect the robustness of the results, suggesting that it should be interpreted with greater caution.

**RESPONSE:** We agree with the Reviewer that we did not have the statistical power to make this evaluation. However, given our unique prospective cohort with documented TBI, we felt it was important to comment on the issue. We modified our statement in the Discussion to say “Additionally, although it must be interpreted cautiously, we did not find evidence to support an increased risk of active TB among persons with TBI who had almost exclusively had symptomatic COVID-19.” We had also stated that “the hypothesis that COVID-19 could trigger active TB in persons with TBI remains unproven and is not supported in our prospective cohort.”

As an ethical consideration, did the study evaluate the possibility of providing antibiotic prophylaxis to participants with evident risk factors for developing active TB, or were these participants referred to the health system for a medical decision regarding this prophylaxis?

**RESPONSE:** Our study followed the standard of care in Kenya which states that TB preventive therapy (prophylaxis) should be provided to select groups of persons including all persons living with HIV and household contacts of persons with bacteriologically confirmed pulmonary TB (see pages 243-244 of the National Guidelines: <https://chskenya.org/wp-content/uploads/2022/04/INTEGRATED-GUIDELINE-FOR-TUBERCULOSIS-LEPROSY-AND-LUNG-DISEASE-2021.pdf>). We referred all QFT+ participants for an evaluation for active TB. Persons living with HIV in our communities routinely receive TB Preventive Therapy (TPT) through their local HIV care clinics.

To support the hypotheses raised, it is recommended to include recent meta-analyses on TB/SARS-CoV-2 co-infection, as they would enrich the introduction and strengthen the study's scientific basis.

**RESPONSE:** Thank you. We replaced reference #4 in the Introduction with a recent meta-analysis by Wang et al. in *PLoS Negl Trop Dis* 2024 “Systematic review and meta-analysis of tuberculosis and COVID-19 co-infection: prevalence, fatality, and treatment considerations” which includes the previous reference #4 and examined outcomes of patients with both COVID-19 and active TB or a history of active TB disease and found that “across multiple studies indicate that individuals with TB-COVID co-infection face a higher risk of hospital admission, longer time-to-recovery, and shorter time-to-death compared to individuals with single COVID-19 infection.” This comment was based primarily on reports from three countries: South Africa, Pakistan and the Philippines.

In addition, reference 12 suggests that TBI predisposes to severe COVID-19; however, the cited article documents a case of reactivation of TBI to active TB in a patient with severe COVID-19, which should be clarified to avoid confusion in interpretation.

**RESPONSE:** Thank you for identifying this error. We have changed the statement in the Introduction referencing #11 and #12 to read: “Some TB researchers have argued that persons with TBI are predisposed to developing active TB after severe COVID-19”. The reference to #12 in the Discussion is accurate in this regard.

Finally, the statistical analysis section should be expanded to detail how the assumptions of the logistic model are verified and to explain better the methods used.

**RESPONSE:** More details have been added to the statistical analysis in the Methods and an addition has been made to the footnote of Table 2.

It would also be helpful to include diabetes and hypertension in the description of baseline characteristics since these variables were considered in the adjusted analyses and could influence the results.
**RESPONSE:** Hypertension and diabetes have been added to the baseline characteristics (Table 1).

It is necessary to include all additional information from the study in the supplementary material to clarify any doubts the reader may have and avoid misinterpretation of the evidence presented.

**RESPONSE:** We are adding as supplementary material the MALCOV study protocol and a preprint of the main study paper. This paper will be published in early 2025.

Reviewer #2: This manuscript reports results of a prospective study evaluating COVID-19 severity among people with and without TB infection (TBI) in western Kenya. The study’s results suggest that those with TBI are not at elevated risk of severe COVID-19. Study strengths include QFT testing to determine TBI status, use of a validated tool to assess COVID-19 severity, and follow-up of up to 18 months to assess longer-term outcomes. Key limitations include the non-random sample and high proportion of possible participants not tested via QFT. The manuscript currently lacks some detail that would help with interpretation of the results.

Major comments:
• The manuscript notably lacks information about COVID-19 vaccination in the study population, which would have an important impact on COVID-19 severity. Was data on vaccination collected?

**RESPONSE:** In Kenya, few patients had received a COVID-19 vaccine at the time of enrolment, as most were enrolled before the full national roll-out of COVID-19 vaccines in mid-2021 that prioritized highest risk groups, including uniformed services and health care workers, followed by those aged over 58 years in accordance with Ministry of Health guidelines at the time. In the parent study, 4% had been vaccinated at baseline (a pre-print version of this paper will be available in early 2025). Among the 387 TBI sub-study participants reported in this manuscript, only five participants were known to have received a COVID-19 vaccine by the time of enrolment, which was also at the time of SARS-CoV-2 diagnosis. Among these five, the highest levels of severity of symptoms during the first 14 days were: no symptoms (1), mild (1), moderate (2), severe (1).

• Because of the methods for recruitment, the study population may have been at increased risk of severe COVID-19 compared to the general population; the authors note this in the discussion. It may be helpful for the recruitment flow diagram to include details on how many participants were recruited via each approach (attending a COVID-19 test center; admitted to COVID-19 isolation/quarantine facility; contact of a case; tested due to high-risk occupation).

**RESPONSE:** Since CONSORT diagrams do not typically include how participants were recruited, we add this information in the Results section: 45.0% of participants were tested at local clinics because they had symptoms and were suspected to have COVID-19, 15.5% were contacts of known cases and were tested at local clinics, 7.2% had no symptoms and were screened at clinics and 32.0% were tested for a variety of other reasons (e.g., high-risk occupations, quarantine facilities, etc.).

• Additional details about the FLU-PRO tool would be useful (perhaps in supplementary information). What symptoms are assessed, and how is severity rated?

**RESPONSE:** The details of the FLUPRO+ tool are described in section 16.5 of the protocol in Appendix V. Modified FLUPRO+ scale. The protocol is now provided as a supplementary material.

• The demographic and clinical data presented are very limited. Were any other data collected? E.g., COVID-19 vaccination, COVID-19 history, TB history, ART status (for those with HIV), COVID-19 treatment received, etc.

**RESPONSE:** We have added hypertension, diabetes and a history of TB to the baseline characteristics (Table 1). By the time recruitment ended in the trial, receipt of the COVID-19 vaccination prior to enrolment was uncommon (4%). (Note that the time of SARS-CoV-2 diagnosis was very close to the date of enrolment.) Similarly, as testing for SARS-CoV-2 was limited in our geographic area, these patients were experiencing their first documented infections with the virus and knowledge of prior infection could not be excluded. As stated in the manuscript, Kenya was experiencing the third and fourth waves of the pandemic during the recruitment period. ART status was not collected systematically and so we do not report on it. Very few participants received COVID-19 treatment; the only medication that was available to this rural, lower-income population was steroid therapy which is described for seven participants in the Results section of our manuscript.

• Did the multivariable regression account for COVID-19 treatment?

**RESPONSE:** No. As mentioned above, seven individuals received COVID-19 specific therapy which consisted solely of dexamethasone or prednisone.

• The methods state that “additional follow-up occurred for up to 18 months,” however it is not clear how follow-up was performed, how frequently, or what data were collected.

**RESPONSE:** The duration of the study was 18 months. Each participant was followed for up to 12 months (this is now clarified). The schedule of activities in the protocol (p. 20 section 2.2.4) which is now provided in the supplementary materials shows that participants were seen or contacted by text message daily for the first 14 days and then contacted through day 20 as needed, after which they were contacted at days 28, 42, and months 3, 6 and 12.

What proportion were lost to follow-up?

**RESPONSE:** For most of the data presented in this paper (days 1-14), only 10 of 373 participants with a positive or negative QFT did not complete all 14 daily FLUPro+ questionnaires. Three were lost to follow-up and seven withdrew from the study. This represents a 1.6% loss to follow-up rate (a median of 2 days missing, average 5.8 days). For the overall study (i.e., to 12 months), the follow-up rate was 94% (see the preprint of the main study).

Did this [loss to follow-up] vary by TBI or COVID-19 severity?

**RESPONSE:** Of the 10 QFT sub-study participants with loss to follow-up, none had severe COVID-19, five were QFT positive and five were QFT negative. The odds of loss to follow-up by QFT status was not statistically significant (p=0.25). Please see also responses to Reviewer 1’s concerns about loss to follow-up.

Minor comments:
• Were participants with a positive QFT referred for TB preventive therapy?

**RESPONSE:** Our study followed the standard of care in Kenya which states that TB preventive therapy (prophylaxis) should be provided to select groups of persons such as all persons living with HIV and household contacts of persons with bacteriologically confirmed pulmonary TB (see pages 243-244 of the National Guidelines: <https://chskenya.org/wp-content/uploads/2022/04/INTEGRATED-GUIDELINE-FOR-TUBERCULOSIS-LEPROSY-AND-LUNG-DISEASE-2021.pdf>). We referred all QFT+ participants for an evaluation for active TB. Persons living with HIV in our communities routinely receive TPT through their local HIV care clinics.

• Figure 3 is a bit difficult to interpret – what does “proportion of daily self-reported COIVD-19 illness” mean? Is this just the proportion reporting symptoms?

**RESPONSE:** Thank you. We have shortened Figure 3’s title to “Self-reported COVID-19 illness by severity…” The fact that the graph shows proportions and is reported on a daily basis are inherent to the titles of the graphs axes.

Reviewer #3: The study is a useful addition the the literature highlighting the t\point that presence of latent tb infection is not associated with Covid-19 disease severity. The study has a considerable sample size. In future viral pandemics like covid, the results of these study will help us understand the relationship between TB infection and viral coinfection.

**RESPONSE:** Thank you.
