# Supplementary material for: Self-reported COVID-19 severity among persons with tuberculosis infection in western Kenya, 2021
Source: PLOS Glob Public Health. 2025 Apr 30;5(4):e0004372. doi: 10.1371/journal.pgph.0004372 (PMC12043119; doi:10.1371/journal.pgph.0004372)
Supplement: Attachment — Submitted filename: PLoS Glob Pub Health reviewer response 2.docx [file pgph.0004372.s005.docx]

Taraz Samandari

2520 Kimbolton Dr.

College Station, TX 77845

February 10, 2025

Dr. María Elvira Balcells, M.D., MSc
Academic Editor

PLOS Global Public Health

1875 Mission Street

Suite 103 #188 San Francisco, CA 94103

United States

Re. Second REBUTTAL LETTER, PGPH-D-24-02424 “Self-reported COVID-19 severity among persons with tuberculosis infection in western Kenya, 2021”

Dear Dr. Balcells,

Once again, we thank you for your comments and the opportunity to improve our manuscript.

In addition to this rebuttal letter we have provided a revised manuscript (v. 3), a track changes version of the manuscript and a revised Figure 2 (per your request). The response to the reviewers is shown below in blue in the rebuttal letter.

Sincerely,

Taraz Samandari, MD PhD

PGPH-D-24-02424R1
Self-reported COVID-19 severity among persons with tuberculosis infection in western Kenya, 2021
PLOS Global Public Health

Major comments:
• As already underlined by the reviewers, SARS-CoV-2 vaccine status is a key information regarding the interpretation of COVID-19 severity. The authors comment in their response that among the 387 sub-study participants reported in this manuscript, only five participants were known to have received a SARS-CoV-2 vaccine by the time of enrolment, which was also at the time of SARS-CoV-2 diagnosis. However, this information has not been incorporated in the revised manuscript results neither in the tables.

**RESPONSE**: This information has been incorporated in the second paragraph of the text of the Results section.

• As previously recommended in the first round of revisions. COVID-19 severity needs to be replaced by “COVID-19 symptoms severity or “self-reported severe COVID-19”. In the Abstract results paragraph, there are still three sentences that refer to “severe COVID-19” that need modified accordingly, as well as in the Abstract’s conclusion. This is particularly relevant as the terms are confusing when including in the same paragraph that a patient died from “severe COVID-19” (which is probably the only occasion in which this “severity” is not only a patient-reported symptom).

**RESPONSE**: The Abstract has been modified in four places and “self-reported” has been added to a few more places in the main text.

Methods:
• In the Methods section, inclusion criteria indicate age over 6 months. As this study evaluated patient reported outcomes, were the authors expecting to use a pediatric /adapted FLUPRO tool for young children? Or is there an error in the lower age limit for inclusion? Or is that the age-inclusion criteria for the parent study only? Please clarify.

**RESPONSE**: The FLUPRO tool was previously validated for a “wide age spectrum” (see Powers JH et al. (2016) “Development of the FluPRO: a patient-reported outcome (PRO) instrument to evaluate symptoms of influenza.” *BMC Infect Dis* 16: 1). Additionally, the FLUPRO developers confirmed that the tool “…was developed for studies of adults and children, with content validity shown in both groups” (Powers JH et al. (2018) “Performance of the inFLUenza Patient-Reported Outcome diary in patients with influenza-like illness”, *PLoS One* 13(3): e0194180.) Finally, they have also indicated that “The content validity of the FLU-PRO has been established in children and adolescents through qualitative research” (Powers et al. (2018) “Reliability, Validity, and Responsiveness of InFLUenza Patient-Reported Outcome Scores in Influenza-Positive Patients” *Value in Health* 21:210). In our cohort of 387 MALCOV QFT sub-study participants, the youngest was an 8 year-old female and so was able to self-report her symptoms. This information has been added to the Methods section of the manuscript.

Results:
• As previously recommended, please avoid starting sentences with numbers to maintain formal scientific writing (e.g. number “387” in first paragraph of Results section, and 57.6% in the third paragraph of Results section).

**RESPONSE**: We have modified those sentences accordingly as well as a handful of others.

• Revise whenever referring to “COVID-19 cases” regarding the whole cohort, as those participants with asymptomatic SARS-CoV-2 infection are not a COVID-19 case (if no disease) (e.g.in the third paragraph of Results section)

**RESPONSE**: We have corrected the phrase “COVID-19 cases” accordingly in five places of the manuscript, including one figure title.

• Results reporting for binary variables in Table 1 and Table 2 is a little bit redundant. I strongly recommend removing all unnecessary rows with duplicated information when reporting binary variables. This is the consensus for scientific journals, in general it is not necessary to include a row for female and another for male, HIV yes/not, hypertensive and not-hypertensive, etc (as the information is complementary), just choose one of them (usually the “yes”). Additionally, in Table 2, the p values column’s titles are missing.

**RESPONSE**: We have revised the Tables accordingly.

• The authors report two cases of incident active TB for the cohort. However, one of them had already been diagnosed and was under TB treatment 1 month before acquiring the SARS-CoV-2 infection. Therefore, it is not correct to include this second case as a “new” TB case developing during the prospective cohort follow-up and I recommend removing that case from the analysis.

**RESPONSE**: We concur. The reference to the person receiving anti-TB therapy at enrolment has been removed. Of note, the calculation of 0.7 cases per 100 person-years in the Discussion includes only the incident (“new”) TB case in our cohort.

Discussion:
• In the Discussion, first paragraph: 1) this is not a prospective cohort of “COVID-19 cases”, as there were asymptomatic SARS-CoV-2 infection cases included. I would recommend replacing by a prospective “cohort of SARS-CoV-2 infections”.

**RESPONSE**: The sentence has been modified although it is noted that only 98.9% had symptoms (1.1% of the cohort had no symptoms).

• I recommend revising the order of the sentences in the Discussion, as it is presently a little bit “jumpy” between one subject and another. As an example: The last sentence of the 1st paragraph of the discussion would better read if introduced after the 1st sentence, as both relate to COVID-19 symptoms severity.

**RESPONSE**: Thank you. The paragraph has been rearranged.

• As steroid use has a strong and well- established evidence as a risk factor to TB infection reactivation (https://pubmed.ncbi.nlm.nih.gov/16463407/) , I am little bit uncomfortable with the sentence related to reference °31 that may give the wrong idea that the risk is low, please rephrase.

**RESPONSE**: We agree with your point that there exists well-established evidence for an increase in the risk of active TB among glucocorticoid users and have modified our paragraph accordingly. It is worth noting that the types of studies conducted by Jick (the reference you provide above and which we have now used but was not available at the time of the ATS-CDC-IDSA in their guideline and now reference #31 in our paper) and the older Schatz paper we had cited (and have now removed) are both good quality studies but are different types of studies in different populations. We had previously chosen the Schatz paper to indicate the basis on which ATS-CDC-IDSA prepared their guideline and to emphasize the types of patients receiving long-term high dose steroids. We decided to use the Jick reference as it was more recent and a larger study.

• In the limitations paragraph, revise the second sentence which refers to “symptomatic” SARS-CoV-2 infection being common in Kenya. Shouldn’t’ this be “asymptomatic” SARS-CoV-2 infections?

**RESPONSE**: Thank you for identifying that typo.

• As the authors presently included in their manuscript capturing patient-centered outcomes in clinical trials has become increasingly recognized as very important, please add a reference.

**RESPONSE**: We have added a reference about the value of patient-centered outcomes as a strength of our study.

Figures:
• The proportions of participants having TBI (+) in each age-group presented in Figure 2B does not seem to agree with the proportion and numbers of participants with TBI for each age group presented in Table 1. Please revise in detail.

**RESPONSE**: Fig 2B and Table 1 are consistent. The table shows column percentages to match other characteristics. If one examines Table 1’s TBI numbers to calculate row percentages, the following calculations readily show the consistency with Figure 2B: <20 (7/67=10%) and >=50 (17/64=27%). The remaining 20-49 year olds are combined in the table but separated in the Figure; their overall % TBI+ is 41% (100/242) which falls within the three bars shown.

• Figure 2: I recommend including the absolute numbers for each column (instead of repeating the information for percentages both on Y axis and above each column)

**RESPONSE**: Absolute numbers have replaced the percentages per your recommendation. The percentage Y-axes are retained due to epidemiologic interest in trends by age for severe COVID-19, HIV prevalence and TBI prevalence.
